# A Quantitative RNAi Screen for JNK Modifiers Identifies Pvr as a Novel Regulator of *Drosophila* Immune Signaling

**DOI:** 10.1371/journal.ppat.1000655

**Published:** 2009-11-06

**Authors:** David Bond, Edan Foley

**Affiliations:** Department of Medical Microbiology and Immunology, University of Alberta, Alberta Institute for Viral Immunology, Edmonton, Alberta, Canada; Stanford University, United States of America

## Abstract

*Drosophila melanogaster* responds to gram-negative bacterial challenges through the IMD pathway, a signal transduction cassette that is driven by the coordinated activities of JNK, NF-κB and caspase modules. While many modifiers of NF-κB activity were identified in cell culture and *in vivo* assays, the regulatory apparatus that determines JNK inputs into the IMD pathway is relatively unexplored. In this manuscript, we present the first quantitative screen of the entire genome of *Drosophila* for novel regulators of JNK activity in the IMD pathway. We identified a large number of gene products that negatively or positively impact on JNK activation in the IMD pathway. In particular, we identified the Pvr receptor tyrosine kinase as a potent inhibitor of JNK activation. In a series of *in vivo* and cell culture assays, we demonstrated that activation of the IMD pathway drives JNK-dependent expression of the Pvr ligands, Pvf2 and Pvf3, which in turn act through the Pvr/ERK MAP kinase pathway to attenuate the JNK and NF-κB arms of the IMD pathway. Our data illuminate a poorly understood arm of a critical and evolutionarily conserved innate immune response. Furthermore, given the pleiotropic involvement of JNK in eukaryotic cell biology, we believe that many of the novel regulators identified in this screen are of interest beyond immune signaling.

## Author Summary

Innate immunity is the sole immune response in the overwhelming majority of multicellular organisms and drives the sophisticated antigen-specific adaptive defenses of vertebrates. Defective regulation of immune signal transduction pathways has disastrous consequences for affected individuals and can result in life-threatening conditions that include cancer, autoimmune and neurological conditions. Thus, there is a major need to identify the regulatory circuits that govern activation of critical innate immune response pathways. The genetically accessible model organism *Drosophila melanogaster* is an ideal springboard for such studies, as core aspects of innate immune pathways are evolutionarily conserved and novel discoveries in *Drosophila* often inspire subsequent developments in the characterization of biomedically relevant mammalian pathways. *Drosophila* responses to certain bacterial invaders proceed through the IMD pathway, which contains partially overlapping signal transduction JNK and NF-κB arms. While substantial efforts have illuminated much of the NF-κB arm, there is a considerable paucity of information on the regulation of the JNK arm. We conducted a survey of the entire *Drosophila* genome for novel regulators the Imd/dJNK pathway. In this study, we uncovered a novel link between the proliferative Pvr pathway and the IMD pathway.

## Introduction

The adaptive immune response is a recent evolutionary acquisition by vertebrates. In contrast, the innate immune response is highly conserved among metazoans and is the first line of defense against invading pathogens [1]. *Drosophila melanogaster* is a powerful model for the study of innate immune signaling events owing to the high degree of evolutionary conservation of signal transduction pathways [2]. For example, pioneering studies in *Drosophila* led to the characterization of Toll as an essential element of invertebrate immune armories, which prompted the search for and characterization of Toll homologs in humans [3],[4]. The identification of the mammalian Toll-like Receptor (TLR) family revolutionized the study of innate immunity in humans and continues to have a profound impact on our understanding of the complexities of vertebrate responses to infectious microbes.

Characterization of a mutation in the *immune deficiency* (*imd*) gene uncovered a distinct immune response to gram-negative bacterial infections in *Drosophila* [5]. Imd is a death-domain containing protein with similarity to the Receptor Interacting Protein (RIP) of the mammalian Tumor Necrosis Factor (TNF) pathway [6]. *Drosophila* immunity to gram-negative bacteria requires an intact IMD signaling pathway, which shares many other similarities with the TNF pathway. Engagement of the IMD pathway requires recognition of diaminopimelic acid-containing peptidoglycan (PGN) by the PGN Receptor Protein (PGRP-LC) [7],[8],[9],[10],[11]. PGRP-LC coordinately activates the *Drosophila* c-Jun N-terminal Kinase (dJNK) and the NF-κB transcription factor family member Relish (Rel). The Rel arm of the IMD pathway is well characterized thanks to a number of individual studies and complementary genetic and cell culture RNA interference (RNAi) screens. Essentially, Rel activation requires the activities of Imd, the caspase-8 ortholog Dredd, dFADD, dTAB2, dIAP2 and the MAP3 kinase dTAK1 [12],[13],[14],[15],[16],[17],[18],[19],[20],[21].

Active dTAK1 drives the subsequent activation of the I-Kappa Kinase (IKK) components Kenny (Key) and Ird5 [22],[23],[24],[25]. Rel is a p105 ortholog with an N-terminal Rel domain and a C-terminal ankyrin repeat domain [26],[27]. While the exact mechanism of Rel activation requires clarification, a recent report identified two distinct aspects to the generation of an active Rel [28]. Signal transduction through the IMD pathway results in the endoproteolytic cleavage of Rel of the N-terminal Rel domain from the inhibitory ankrin repeat domain. At the same time, activation of IKK activation drives the phosphorylation and transcriptional activation of Rel. The Rel domain translocates to the nucleus and initiates the transcription of a large number of genes, such as the antimicrobial peptides (AMPs) *attacin* (*att*) and *diptericin* (*dipt*).

IMD pathway activation of dTAK1 also stimulates a kinase cascade through the MAP2Ks dMAPKK4/7 that leads to dJNK phosphorylation [29],[30]. Phosphorylated dJNK typically activates the nuclear translocation of the AP-1 transcription factor subunits dJun and dFos, which initiate the transcription of dJNK depended gene products [31]. dJNK activation is a transitory event in the IMD pathway [30]. pJNK protein levels are downregulated through the combined activities of Rel and dJNK-responsive transcripts such as the phosphatase Puckered [30],[32],[33]. Mutations in *djnk* are lethal due to defective epithelial sheet sealing in the dorsolateral axis of the developing embryo [34],[35]. The developmental requirement for dJNK and other components of the dJNK arm of the IMD pathway has hampered the study of dJNK signaling events in innate immune signaling. Thus, the processes that regulate dJNK phosphorylation in the IMD pathway are poorly understood and many of the mechanisms that regulate dJNK signaling remain unknown.

*Drosophila* tissue culture cells provide an ideal environment to study these events, as PGN-induced activation of the IMD pathway induces a transient dJNK activation that is easily quantified. To understand the regulation of PGN-induced dJNK phosphorylation in the IMD pathway, we performed a high-throughput, quantitative RNAi screen for modulators of dJNK phosphorylation. To this end, we treated the embryonic macrophage-like S2 cell line with 15,683 individual dsRNAs that cover all annotated genes in the *Drosophila* genome. In contrast to previous RNAi screens of the IMD pathway, our assay did not rely on indirect reporter constructs. Instead, we used phospho-JNK specific monoclonal antibodies in a quantitative plate-based assay to directly quantify the impact of each dsRNA on the extent of PGN-induced dJNK phosphorylation. In this manner, we identified enhancers and suppressors of dJNK activation. As a testament to the accuracy of this screen, we unambiguously identified fifteen established IMD pathway components as modifiers of dJNK activation. In addition, we identified numerous novel regulators of dJNK activation. Given the involvement of dJNK in cellular events as diverse as development, cell migration, immune signaling and cell death, we believe that many of the regulators identified in this screen will be of broad interest to the study of metazoan cell biology.

We present a comprehensive analysis of a novel regulator of dJNK in IMD pathway signaling – the PDGFR and VEGFR receptor (Pvr) tyrosine kinase. Pvr is primarily known for its role in the guidance of cellular movements [36],[37],. We uncover a novel inhibitory circuit in the IMD pathway, where dJNK drives the expression of the Pvr ligands, Pvf2 and Pvf3, which subsequently contribute to the downregulation of dJNK activity via a Pvr/dERK signal transduction cassette. We also demonstrate that Pvr attenuates the expression of Rel-responsive transcripts by regulating the extent of Rel phosphorylation. We confirm a regulatory role for Pvr in the IMD pathway with data that loss of Pvr in adult *Drosophila* enhances the infection-induced expression of *att*. These data indicate that the Pvr/dERK signal transduction pathway constitutes a novel negative regulator of the *Drosophila* IMD pathway.

## Results

### In-Cell Western (ICW) Assay for PGN-induced dJNK Phosphorylation

**Figure 1:**
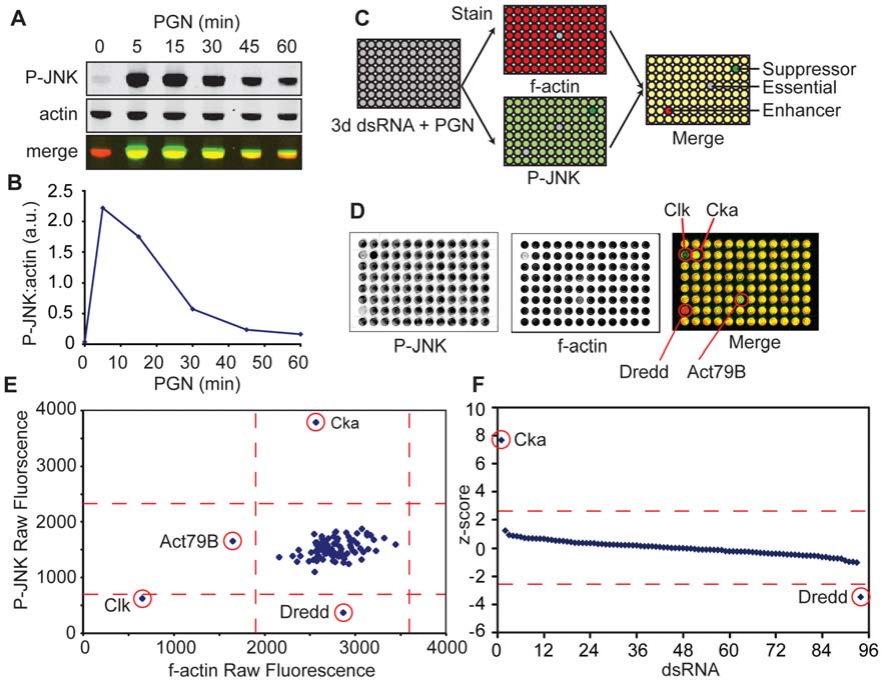
Quantitative high-throughput RNAi screen for modifiers of dJNK phosphorylation. (A) Representative Western blot analysis of S2 cell lysates treated with PGN for the indicated period. Lysates were probed with anti-P-JNK (top panel) and anti-actin (middle panel). To visualize relative dJNK phosphorylation P-JNK (green) and actin (red) channels were false colored and merged (bottom panel). (B) Quantification of relative dJNK phosphorylation in (A). dJNK phosphorylation levels were quantified and reported relative to actin levels for each of the indicated time points. (C) Schematic representation of a quantitative RNAi screen for modifiers of PGN-induced dJNK phosphorylation. S2 cells were incubated with dsRNA in 96 well plates prior to exposure to PGN. Cells were stained with an antibody specific for P-JNK and were counterstained with fluorescently labled phalloidin to visualize filamentous actin (f-actin). JNK phosphorylation levels were quantified relative to f-actin levels. Loss of activators (enhancers) of JNK phosphorylation decreases dJNK phosphorylation. In contrast, loss of inhibitory gene products (suppressors) increases dJNK phosphorylation. Essential gene products are visible as wells with no P-JNK or f-actin staining. (D) Representative plate from screen. S2 cells incubated with 96 distinct dsRNAs were treated with PGN for 15min. Cells were stained for P-JNK with monoclonal antibodies specific for P-JNK (left) and counterstained for f-actin (center). To visualize relative P-JNK levels, P-JNK (green) and f-actin (red) channels were false colored and merged (right). (E) Quantification of relative dJNK phosphorylation levels from panel D. Raw dJNK phosphorylation values were graphed against raw f-actin values. Red dashed lines indicate + or − 2.58 standard deviations from the median for both P-JNK and f-actin values. dsRNA targeting the established JNK modifiers Dredd and Cka decrease or increase dJNK phosphorylation levels respectively with no effect on f-actin levels. (F) Statistical analysis of PGN-induced dJNK phosphorylation relative to f-actin from panel E. P-JNK values were standardized to f-actin values for each of the 96 dsRNA treatments. The red dashed lines represent z-score values of + or − 2.58. dsRNA that targeted Cka and Dredd were identified as significant modifiers of PGN-induced dJNK phosphorylation.

Engagement of the IMD pathway leads to transient dJNK phosphorylation; PGN-induced dJNK phosphorylation peaks at 5min and returns to basal levels by 60min in S2 cells (e.g. Figure 1A, B). We developed a quantitative high-throughput dsRNA screen to identify novel regulators of dJNK signaling in the IMD pathway (Figure 1C). To this end, we treated *Drosophila* S2 cells with a library of 15,683 dsRNAs that cover all annotated genes in the *Drosophila melanogaster* genome and we monitored the subsequent extent of PGN-induced dJNK phosphorylation by In Cell Western (ICW) analysis. We used monoclonal antibodies specific for phosphorylated JNK (P-JNK) and fluorescently labeled secondary antibodies to directly visualize PGN-induced dJNK phosphorylation. We simultaneously monitored filamentous actin (f-actin) levels with fluorescently labeled phalloidin as a control measure of total cell numbers. We then quantified the ratio of P-JNK:f-actin for each well to determine the relative extent of dJNK phosphorylation in each sample. To identify genes that modulate the intensity and duration of dJNK phosphorylation, we screened the entire genome at fifteen and sixty minutes. We reasoned that depletion of gene products that are required for optimal PGN-induced dJNK phosphorylation will decrease dJNK phosphorylation at fifteen minutes and we defined such gene products as enhancers of dJNK phosphorylation. Likewise, we reasoned that depletion of gene products involved in dJNK dephosphorylation will increase the relative intensity and/or duration of dJNK phosphorylation at fifteen and/or sixty minutes and we defined such gene products as suppressors of dJNK phosphorylation.

A representative 96-well plate from the screen is shown in Figure 1D and the corresponding quantification of the P-JNK:f-actin levels are shown in Figure 1E. Consistent with a previous report [43], we identified Dredd as an enhancer of dJNK phosphorylation. In addition, we identified the dJNK signaling pathway element Cka as a suppressor of PGN-induced dJNK phosphorylation. As expected, we identified Act79B as a regulator of f-actin levels and the gene product Clk as essential for S2 cell viability. To eliminate dsRNAs that negatively affected cell viability or cell adherence, we excluded dsRNAs that greatly reduced cell numbers as determined by an absence of f-actin and P-JNK fluorescence from subsequent analyses. We then calculated the P-JNK:f-actin z-score for all remaining wells to determine the statistical significance of dsRNA-treatment on PGN-induced dJNK phosphorylation and to allow for inter-plate comparisons. By these criteria, we successfully identified Cka and Dredd as statistically significant modifiers of dJNK phosphorylation with z-scores of 7.70 and -3.48, respectively (Figure 1F). These data indicate that the ICW assay is an effective method to detect modifiers of PGN-induced dJNK phosphorylation in S2 cells.

### Quantitative High-thoughput dsRNA Screen for Regulators of PGN-induced dJNK Phosphorylation

**Figure 2:**
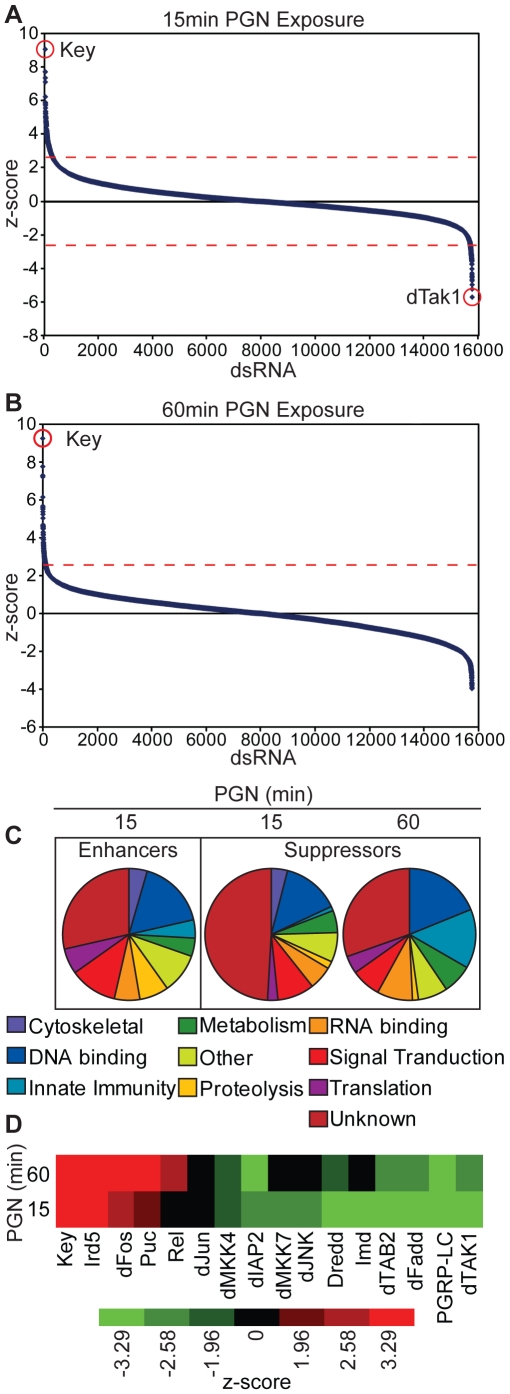
Whole genome dsRNA screen for modifiers of PGN-induced dJNK phosphorylation in the IMD pathway. (A,B) Quantification of relative dJNK phosphorylation in S2 cells incubated with 15,683 distinct dsRNAs prior to PGN exposure for 15min (A) or 60min (B). The relative P-JNK:f-actin z-score was determined for each dsRNA. The red dashed lines represent z-score values of + or − 2.58. Key was identified as the strongest suppressor of dJNK phosphorylation at 15min and 60min PGN exposure (A,B), whereas Tak1 was identified as the strongest enhancer of dJNK phoshorylation at 15min PGN exposure (B). (C) dsRNAs that modified dJNK phosphorylation in response to PGN were grouped according to biological functions. The biological functions for enhancers of dJNK phosphorylation with a z-score below −2.58 at 15min PGN exposure are presented (left panel). Additionally, the biological functions for suppressors of dJNK phosphorylation with a z-score above 2.58 at 15min and 60min PGN-exposure are presented (right panel). In addition to recognizable groups, the dsRNA screen identified a large number of *Drosophila* genes with unknown biological functions as modifiers of PGN-induced dJNK phosphorylation. (D) Heat map of z-score values for S2 cells depleted of known Imd pathway components and exposed to PGN for 15min or 60min. Fifteen core IMD pathway components were identified in the screen as either suppressors (z-scores above 1.96) or enhancers (z-scores below −1.96) of PGN-induced dJNK phosphorylation respectively.

We then measured the PGN-induced P-JNK:f-actin levels and determined the z-score for all non-lethal dsRNA treatments. We graphed all the z-scores from highest to lowest for both fifteen and sixty minutes PGN-exposures (Figure 2A,B). dsRNA-mediated depletion of enhancers or suppressors of PGN-dependent dJNK phosphorylation resulted in reduced or elevated P-JNK z-scores, respectively. The z-scores for all dsRNAs are available in Table S1. We disregarded the P-JNK enhancers at sixty minutes PGN-exposures because the level of PGN-induced dJNK phosphorylation was not sufficiently elevated over background P-JNK levels. We identified Key as the strongest suppressor of dJNK phosphorylation at both fifteen and sixty minutes with z-scores of 9.05 and 9.23, respectively. Conversely, we identified dTAK1 as the strongest enhancer of dJNK phosphorylation at fifteen minutes PGN-exposure with a z-score of −5.7. As the Key/Rel axis of the IMD pathway attenuates dJNK activation and dTAK1 is essential for dJNK phosphorylation, these data are consistent with the known roles of Key and dTAK1 in the IMD pathway. We grouped all suppressors of dJNK phosphorylation with z-scores above 2.58 at fifteen and sixty minutes and all enhancers of dJNK phosphorylation with z-scores below −2.58 at fifteen minutes according to their known biological functions (Figure 2C). We identified many genes involved in innate immune signaling, in addition to a large number of genes with previously uncharacterized functions (Tables S2, S3, S4). As a testament to the saturation of this screen, we identified fifteen IMD pathway components as modulators of PGN-induced dJNK phosphorylation with z-scores above 1.96 or below −1.96 (Figure 2D). We note that in each case the z-score is consistent with the established role of the fifteen genes as either suppressors or enhancers of dJNK phosphorylation.

### Validation of Pvr as a Suppressor of PGN-induced dJNK Phosphorylation

**Figure 3:**
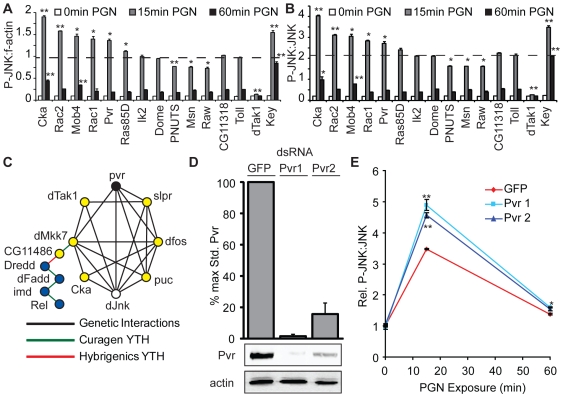
Pvr is a negative regulator of dJNK phosphorylation in the IMD pathway. (A,B) Quantification of PGN-induced dJNK phosphorylation relative to f-actin (A) or total JNK (B). S2 cells were incubated with the indicated dsRNAs and exposed to PGN at 0min, 15min or 60min as indicated. Key and dTak1 dsRNA were used as modifier dsRNA controls, whereas Toll and CG11318 dsRNA were used as non-modifier dsRNA controls. Cells were stained with anti-P-JNK antibody and dJNK phosphorylation was standardized to f-actin (A) and total dJNK (B). Data are presented as the mean of two independent experimental values and error bars indicate + SEM. The grey dashed line represents the mean dJNK phosphorylation value for Toll dsRNA and dsRNAs that significantly modulated dJNK phosphorylation from this value are indicated (* = p-value <0.05, ** = p-value <0.01). Secondary dsRNA analysis recapitulates the dJNK phosphorylation values from the primary screen. (C) A partial genetic and physical interaction network of dJNK phosphorylation modifiers. Modulators of dJNK phosphorylation with z-scores greater than 1.96 or less than −1.96 were grouped in an interaction network using known genetic and physical interaction databases. Within this network, *Pvr* (black circle) and *dJnk* (white circle) are connected directly and through a number of intermediate genes (yellow circles). The *Pvr* and *dJnk* interaction network also connects to IMD pathway (blue circles). (D) Quantitative Western blot analysis of lysates from S2 cells treated with either Pvr or GFP dsRNA. Lysates were probed with anti-Pvr (top blot) and anti-actin (bottom blot) antibodies. Pvr levels were quantified relative to actin (top graph). Data are presented as mean of three independent experiments and error bars indicate + SEM. Both Pvr dsRNA molecules deplete Pvr in S2 cells. (E) Quantification of PGN-induced dJNK phosphorylation. S2 cells were treated with GFP dsRNA as a control or two independent non-overlapping dsRNA targeting Pvr as indicated. Cells were exposed to PGN and dJNK phosphorylation was monitored relative to total dJNK. P-JNK:JNK values at 0h PGN exposure with GFP dsRNA were assigned a value of 1 and the remaining P-JNK:JNK values are reported relative to these data. Data is expressed as the mean of two independent experiments and the error bars represent +/− SEM. Significant differences in pJNK values are indicated (* = p-value<0.05, ** = p-value<0.01). Depletion of Pvr increases PGN-induced dJNK phosphorylation at 15min, indicating that Pvr negatively regulates dJNK activation in the IMD pathway.

To test the validity of the dsRNA screen, we selected a representative cohort of three enhancers and eight suppressors of PGN-induced dJNK phosphorylation for secondary analysis. We monitored the effect of dsRNA treatment for all genes in the cohort on dJNK phosphorylation relative to f-actin at zero, fifteen and sixty minutes PGN-exposure. We compared the eleven putative modifier dsRNAs to two dsRNAs (CG11318 and Toll) that had no effect on dJNK phosphorylation in the primary screen. Secondary dsRNA analysis was consistent with the screen results as nine of the eleven dsRNAs significantly modified dJNK phosphorylation relative to f-actin compared to control dsRNA (Figure 3A). Even though we excluded actin modifiers from our primary data analysis, we considered the possibility that a fraction of the phenotypes observed may be indirectly caused by effects on f-actin, as opposed to dJNK phosphorylation. To test this hypothesis, we depleted each gene in the cohort and monitored PGN-induced dJNK phosphorylation relative to total dJNK by ICW (Figure 3B). We observed that the P-JNK:JNK analysis essentially mirrored the P-JNK:f-actin analysis for each gene in the cohort. Thus, we have confidence that our screen primarily identified regulators of PGN-dependent dJNK phosphorylation. To map relationships between the identified modulators of PGN-induced dJNK phosphorylation, we mined known genetic and physical interaction databases to develop an interaction network for all hits in our primary screen. We restricted the interaction network to direct physical or genetic interactions between genes identified as modifiers of dJNK phosphorylation. Within this direct interaction network we identified a branch with a high density of interactions that spanned the IMD and the dJNK signaling pathways (Figure 3C). The *Drosophila* PDGF/VEGF Receptor (Pvr) homolog appeared as a major node within this branch.

To confirm Pvr as a suppressor of dJNK phosphorylation in the IMD pathway, we depleted S2 cells of Pvr with two independent non-overlapping dsRNAs and monitored relative dJNK phosphorylation upon exposure to PGN at zero, fifteen and sixty minutes. We confirmed that both dsRNAs deplete Pvr by monitoring Pvr protein levels relative to actin in S2 cell lysates using Pvr specific antibodies (Figure 3D). Treatment of S2 cells with Pvr dsRNA 1 or 2 reduced relative Pvr protein levels to 1.6% and 15.6% of the control, respectively. In addition, depletion of Pvr by either dsRNA significantly increased PGN-induced dJNK phosphorylation at fifteen minutes (Figure 3E). Thus, we conclude that Pvr suppresses PGN-dependent dJNK phosphorylation.

### *pvf2* and *pvf3* are IMD/dJNK-Responsive Transcripts

**Figure 4:**
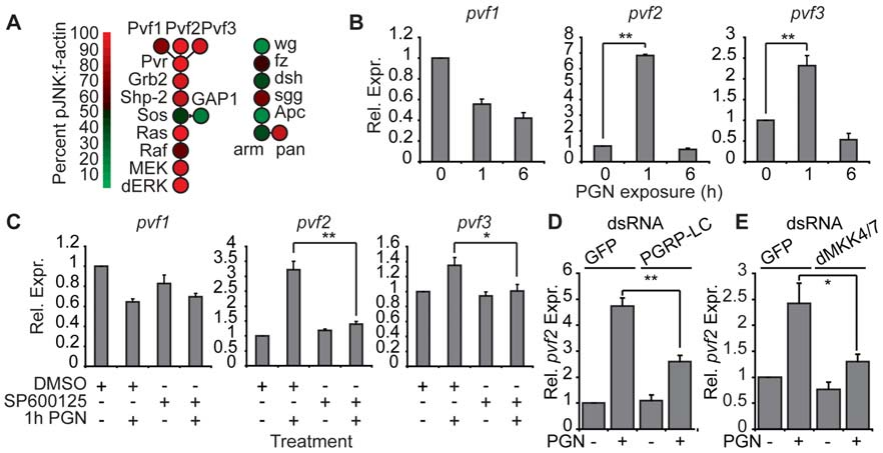
*pvf2* and *pvf3* are immune Induced dJNK-dependent transcripts. (A) Heat map analysis of known Pvr pathway genes compared to known wg pathway genes at 15 min PGN-exposure. 15min PGN-induced dJNK:f-actin z-scores were ordered from highest to lowest and organized according to percentile intervals. Genes colored more red indicate suppressors of PGN-induced dJNK phosphorylation while genes colored more green indicate enhancers of PGN-induced dJNK phosphorylation. Pvr pathway components were consistently identified as suppressors of PGN-induced dJNK phosphorylation. (B) Quantitative real-time PCR analysis of standardized *pvf1*,*2* and *3* expression in S2 cells treated with PGN for the indicated times. The uninduced expression levels for *pvf1*,*2* and *3* were given values of 1 and the remaining *pvf1*,*2* and *3* expression values are reported relative to these values. Data are presented as mean of three independent experiments and error bars indicate the + SEM. Significant differences in expression values are indicated (** = p-value <0.01). (C) Quantitative real-time PCR analysis of standardized *pvf1*,*2* and *3* expression in S2 cells or S2 cells treated with SP600125. S2 cells were incubated with SP600125 and PGN as indicated. The uninduced expression levels for *pvf1*,*2* and *3* were given values of 1 and the remaining *pvf1*,*2* and *3* expression values are reported relative to these values. Data are presented as mean of three independent experiments and error bars indicate the + SEM. Significant differences in P-JNK values are indicated (* = p-value <0.05, ** = p-value <0.01). (D) Quantitative real-time PCR analysis of standardized *pvf2* expression in S2 cells incubated with GFP or PGRP-LC dsRNA and treated with PGN as indicated. *pvf2* expression levels of unstimulated S2 cells treated with GFP dsRNA were assigned a value of 1 and the remaining *pvf2* expression values are reported relative to these values. Data are presented as mean of three independent experiments and error bars indicate the + SEM. Significant differences in P-JNK values are indicated ( ** = p-value <0.01). (E) Quantitative real-time PCR analysis of standardized *pvf2* expression in S2 cells incubated with GFP or a combination of dMKK4/7 dsRNA and treated with PGN as indicated. *pvf2* expression levels of unstimulated S2 cells treated with GFP dsRNA were assigned a value of 1 and the remaining *pvf2* expression values are reported relative to these values. Data are presented as mean of three independent experiments and error bars indicate the + SEM. Significant differences in P-JNK values are indicated ( * = p-value <0.05).

While a previous dsRNA screen hinted at a role for Pvr in the IMD pathway [14], Pvr is primarily known for its role in *Drosophila* ERK signaling and cell migration. To investigate the involvement of the Pvr pathway in attenuation of dJNK activation, we determined the dJNK:f-actin z-score for each member of the Pvr/dERK axis in the primary screen. As a comparison, we also determined the dJNK:f-actin z-scores for members of the wingless pathway – a signal transduction pathway with no know interaction with the IMD/dJNK module. As expected, our data do not indicate any major interactions between the wingless and IMD/dJNK pathways. In contrast, our data consistently indicate that the Pvr/dERK pathway negatively regulates dJNK activation (Figure 4A). Ablation of the Pvr ligands Pvf2 and Pvf3; Pvr; established dERK adaptors; Ras and dERK resulted in considerably increased PGN-mediated dJNK phosphorylation.

We then asked if IMD pathway activation results in expression of Pvr ligands. Treatment of S2 cells with PGN resulted in a minor decline in the expression of *pvf1* and significant increases in the levels of *pvf2* and *pvf3* expression (Figure 4B). Induction of *pvf2* and *pvf3* reached maximal levels within one hour of PGN treatment and reverted to basal levels by six hours. These expression patterns are reminiscent of other IMD/dJNK-responsive transcripts. To confirm that *pvf2* and *pvf3* are dJNK-responsive transcripts, we pre-incubated S2 cells with the dJNK inhibitor SP600125 and monitored the subsequent levels of *pvf2* and *pvf3* expression in response to PGN. Our data showed that SP600125 completely blocked the PGN-dependent expression of *pvf2* and *pvf3* (Figure 4C). Likewise, we observed a significant reduction in PGN-dependent *pvf2* induction in cells depleted of PGRP-LC (Figure 4D) or dMKK4/dMKK7 (Figure 4E), confirming a requirement for the IMD/dJNK cassette in *pvf2* induction by PGN. In summary, these data show that activation of the IMD pathway results in the dJNK-dependent expression of the Pvr ligands Pvf2 and Pvf3 and that the Pvr/dERK pathway attenuates dJNK activation.

### Pvr Suppresses PGN-induced Rel Signaling

**Figure 5:**
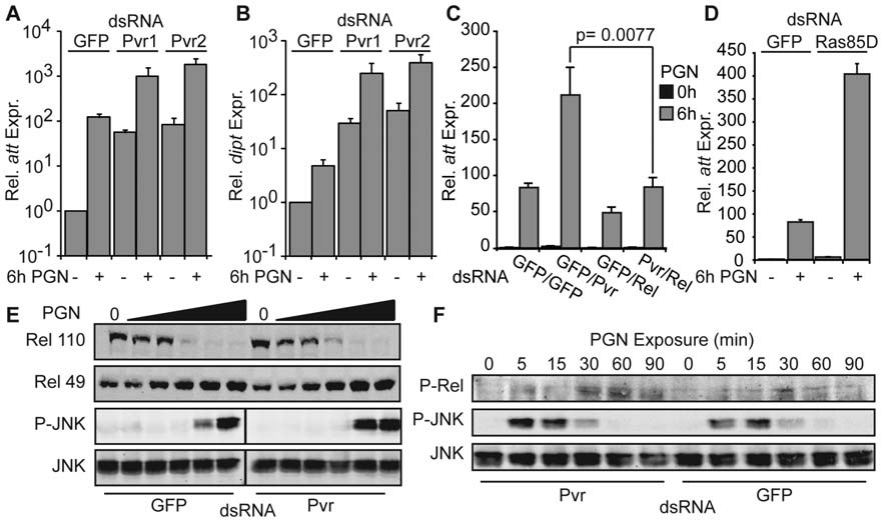
Pvr depletion increases antimicrobial peptide production in S2 cells. (A) Quantitative real-time PCR analysis of standardized *att* expression in S2 cells incubated with either GFP or two distinct Pvr dsRNA. S2 cells were treated with PGN where indicated. *att* expression levels of unstimulated S2 cells treated with GFP dsRNA were assigned a value of 1 and the remaining *att* expression values are reported relative to these values. Data are presented as mean of two independent experiments and error bars indicate the + SEM. (B) Quantitative real-time PCR analysis of standardized *dipt* expression in S2 cells incubated with either GFP or two distinct Pvr dsRNA. S2 cells were treated with PGN for 6h where indicated. *dipt* expression levels of unstimulated S2 cells treated with GFP dsRNA were assigned a value of 1 and the remaining *dipt* expression values are reported relative to these values. Data are presented as mean of two independent experiments and error bars indicate + SEM. (C) Quantitative real-time PCR analysis of standardized *att* expression in S2 cells incubated with GFP or Pvr dsRNA in combination with Rel dsRNA. S2 cells were treated with the indicated dsRNAs and unstimulated or stimulated with PGN for 6h as indicated. *att* expression levels of unstimulated S2 cells treated with GFP dsRNA were assigned a value of 1 and the remaining *att* expression values are reported relative to these values. Data are presented as mean of three independent experiments and error bars indicate the + SEM. Significant differences in *att* expression values are indicated with a p-value. (D) Quantitative real-time PCR analysis of standardized *att* expression in S2 cells incubated with GFP or Ras85D dsRNA and treated with PGN as indicated. *att* expression levels of unstimulated S2 cells treated with GFP dsRNA were assigned a value of 1 and the remaining *att* expression values are reported relative to these values. Data are presented as mean of two independent experiments and error bars indicate the + SEM. (E) Western blot analysis of lysates from S2 cells incubated with GFP dsRNA (lanes 1–6) or Pvr dsRNA (lanes 7–12). S2 cells were untreated or treated with PGN in increasing ten-fold gradations of LPS from 5×10^−4^µg/ml to 5µg/ml. Lysates were probed with anti-Rel (top panels), anti-P-JNK (middle) and anti-JNK (bottom panel). (F) Western blot analysis of lysates from S2 cells incubated with Pvr dsRNA (lane 1–6) or GFP dsRNA (lanes 7–12) and treated with PGN for the indicated period. Lysates were probed with anti-P-Rel (top panel), anti-P-JNK (middle) and anti-JNK (bottom panel).

Given that Pvr suppresses dJNK signaling in the IMD pathway, we asked if Pvr also modulates Rel signaling events. To determine if Pvr depletion affects Rel signaling in the IMD pathway, we depleted S2 cells of Pvr with two independent non-overlapping Pvr dsRNAs and monitored PGN-induced AMP expression. Specifically, we monitored expression of the Rel-responsive AMPs *dipt* and *att*. Depletion of Pvr by either dsRNA profoundly strengthened PGN-induced expression of *att* and *dipt* in comparison to control S2 cells (Figure 5A, B). Additionally, Pvr depletion significantly increased the basal expression levels of both *att* and *dipt*, in the absence of PGN stimulation. In fact, the basal levels of *att* or *dipt* expression in cells treated with Pvr dsRNA are approximately equal to the PGN-induced expression levels in cells treated with GFP control dsRNA. These data show that loss of Pvr in S2 cells results in an increase in both the uninduced and the PGN-induced expression of AMPs.

To confirm that the increased AMP expression observed upon Pvr loss proceeds through Rel, we then examined the expression of *att* in S2 cells that were simultaneously treated with Pvr and Rel dsRNA. As expected, depletion of Pvr increased the PGN-mediated expression of *att* (Figure 5C). In contrast, PGN-mediated expression of *att* was greatly reduced in cells treated with a combination of Rel and Pvr dsRNA. Thus, our data indicate that the bulk of Pvr RNAi-dependent increases in *att* expression proceed through the IMD/Rel module. In agreement with a role for the Pvr pathway in reducing *att* expression, we also observed increased *att* induction in cells treated with Ras85D dsRNA (Figure 5D). As Pvr loss leads to enhanced Rel-mediated AMP expression, we then asked if Pvr affects Rel cleavage or Rel phosphorylation. Whereas depletion of Pvr greatly sensitized S2 cells to PGN-dependent induction of dJNK phosphorylation (e.g. compare lanes 5 and 11, Figure 5E), we did not detect alterations in the pattern of PGN-induced Rel cleavage in S2 cells treated with Pvr dsRNA (Figure 5E). In contrast, we consistently detected prolonged and increased PGN-responsive phosphorylation Rel (P-Rel) in S2 cells treated with Pvr dsRNA (Figure 5F). These data indicate that Pvr negatively regulates the PGN-induced phosphorylation of both dJNK and Rel in the IMD pathway.

### Pvr Signaling Suppresses PGN-dependent IMD Pathway Activation

**Figure 6:**
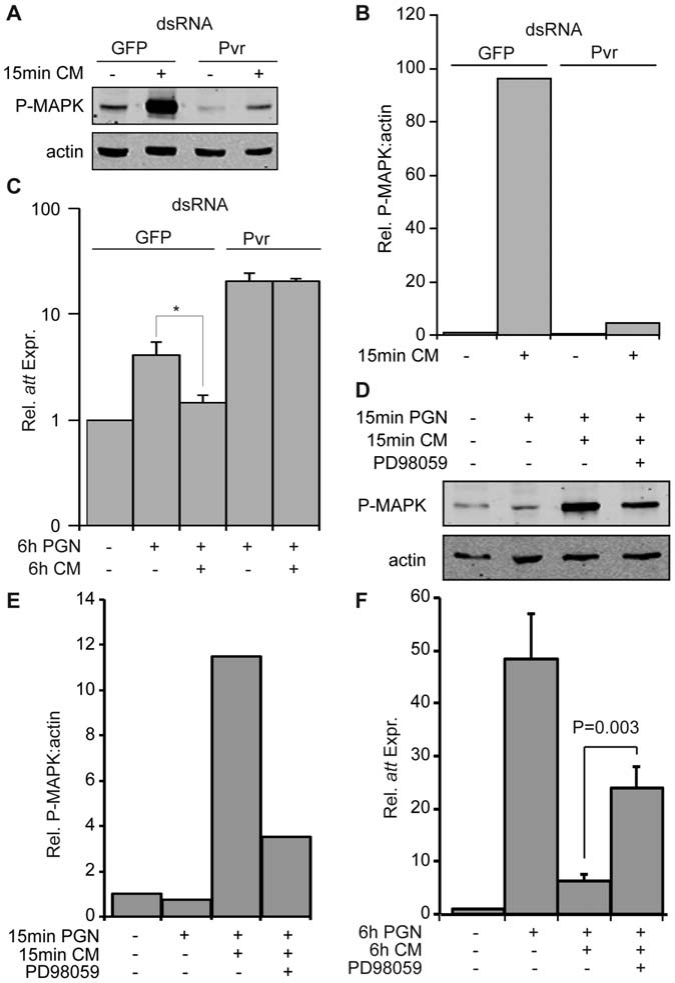
Pvr inhibits antimicrobial peptide production in S2 cells. (A) Western blot analysis of lysates from S2 cells incubated with GFP dsRNA (lane 1–2) or Pvr dsRNA (lanes 3–4). S2 cells were exposed to KC167 cell conditioned media (CM) where indicated to induce MAPK phosphorylation. Lysates were probed with antibodies specific for P-MAPK (top panel), and actin (bottom panel). Data is representative of three individual experiments. (B) Relative quantification of P-MAPK levels from (A). P-MAPK levels were standardized to actin levels for each treatment group. The unstimulated GFP dsRNA treated P-MAPK:actin value was given a value of 1 and the remaining P-MAPK:actin values are reported relative to this value. (C) Quantitative real-time PCR analysis of *att* expression in S2 cells incubated with GFP dsRNA (columns 1–3) or Pvr dsRNA (columns 4–5). S2 cells were treated with KC167 CM, and PGN as indicated. *att* expression levels in unstimulated S2 cells treated with GFP dsRNA were assigned a value of 1 and the remaining *att* expression values are reported relative to these values. Data are presented as the mean of three independent experiments and error bars indicate + SEM. The significance of CM treatment on *att* expression relative to the untreated samples is indicated (* = p-value <0.05). CM does not suppress *att* expression in the absence of Pvr. (D) Western blot analysis of lysates from S2 cells treated with the MEK1 inhibitor PD98059 followed by exposure to PGN, and KC167 CM as indicated. Lysates were probed with anti-P-MAPK (top panel), and anti-actin (bottom panel) antibodies. (E) Relative quantification of P-MAPK levels in panel D. P-MAPK levels were standardized to actin levels for each treatment group. The untreated P-MAPK:actin value was given a value of 1 and the remaining P-MAPK:actin values are reported relative to this value. (F) Quantitative real-time PCR analysis of *att* expression levels in S2 cells (column 1–3) or S2 cells treated with PD98059 (column 4). S2 cells were incubated with KC167 CM, and PGN as indicated. *att* expression levels in unstimulated S2 cells were assigned a value of 1 and the remaining *att* expression values are reported relative to these values. Data are presented as the mean of three independent experiments and error bars indicate + SEM. Inhibition of MEK1 activation with PD98059 significantly restored *att* expression in response to KC167 CM.

Given our findings that Pvr depletion increases AMP expression, we asked if activation of Pvr suppresses the IMD pathway. We monitored dERK phosphorylation to visualize Pvr signaling, as Pvr engagement results in activation of dERK in S2 cells. Previous reports demonstrated that Pvr ligands in conditioned medium (CM) from the *Drosophila* KC167 cell line activates Pvr signaling in S2 cells [44]. Likewise, we observed a requirement for Pvr in KC167 CM-induced dERK phosphorylation in S2 cells (Figure 6A). Quantification of relative dERK phosphorylation levels showed that Pvr dsRNA treatment decreased CM-induced dERK phosphorylation 21 fold (Figure 6B). To examine the effect of Pvr signaling on AMP expression, we treated S2 cells with GFP or Pvr dsRNA and monitored PGN-induced *att* expression levels 6h after exposure to CM (Figure 6C). Consistent with the role of Pvr as a suppressor of Rel signaling, we found that CM significantly decreased PGN-induced *att* expression. The phenotype is not an indirect effect of CM on PGN or other aspects of the IMD pathway, as dsRNA-mediated depletion of Pvr from S2 cells abrogated the suppressive effects of CM on *att* expression (Figure 6C). Thus, we conclude that activation of Pvr blocks PGN-responsiveness in S2 cells.

As Pvr signaling often proceeds through dERK and the bulk of the Ras/dERK pathway yielded Pvr-like phenotypes in our primary screen, we then tested if dERK phosphorylation is required for CM suppression of PGN-induced *att* expression. Treatment of S2 cells with the MEK1 inhibitor PD98059 decreased CM-induced dERK phosphorylation 3.2 fold relative to S2 cells treated with CM alone (Figure 6D, E). To test the effect of dERK inhibition on CM-mediated suppression of *att* expression, we pretreated S2 cells with PD98059 prior to exposure to PGN and CM (Figure 6F). CM suppressed the PGN-induced expression of *att* by 7.7 fold. However, we detected significant restoration of PGN-induced *att* expression in S2 cells treated with CM and PD98059. These data indicate that signal transduction through a Pvr/dERK axis attenuates activation of the IMD pathway.

### Pvr Suppresses AMP Production *In Vivo*

**Figure 7:**
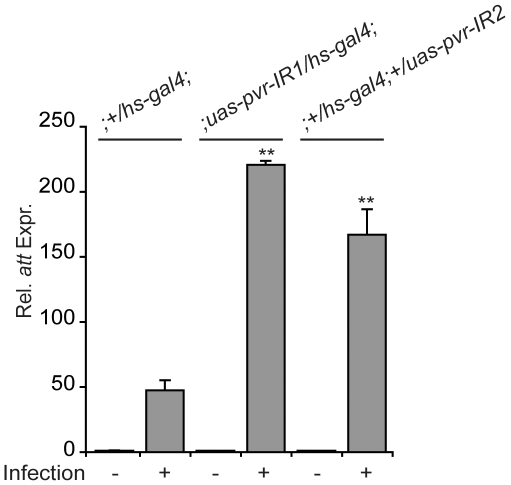
Pvr attenuates infection-induced antimicrobial peptide production *in vivo*. Quantitative real-time PCR analysis of *att* expression in *hs-gal4*/+ control flies (columns 1–2), *hs-gal4*/*uas-Pvr-IR1* flies (columns 3–4) and *hs-gal4*/+; *uas-Pvr-IR2*/+ flies (columns 5–6). Flies were uninfected or infected through septic injury with *E. coli* as indicated. *att* expression levels of uninfected controls were assigned a value of 1 and the remaining *att* expression values are reported relative to these value. Data are presented as mean of three independent experiments and error bars indicate the + SEM. Significant differences in *att* expression are indicated (** = p-value <0.01).

We then asked if Pvr suppresses IMD pathway activity *in vivo*. To reduce Pvr activity in whole animals, we expressed Pvr dsRNA hairpin constructs (Pvr-IR) in adult flies. We then compared the immune response of infected wild type flies to flies that express Pvr-IR. Specifically, we monitored the expression of the Rel-responsive transcript *att* in uninfected flies (control) and flies that were pricked with a needle coated in *E. coli* (infection). Strikingly, we noticed that *in vivo* depletion of Pvr significantly enhanced infection-mediated *att* expression in three separate experiments in two separate Pvr-IR fly lines (Figure 7). These data indicate that depletion of Pvr from adult flies results in increased IMD pathway activity *in vivo* and support a role for Pvr as a negative regulator of Imd pathway activity.

## Discussion

Signal transduction through the JNK family of MAP kinases is a central element of vertebrate and invertebrate innate immune responses to infectious microbes. In addition, JNK activation contributes to the regulation of essential cellular processes, such as differentiation, apoptosis and directed cell movements [45],[46],[47]. The pleiotropic developmental and homeostatic requirements for JNK activity combined with functional redundancies among JNK pathway member isoforms hampered large-scale evaluations of JNK in model systems. In this study, we present the first whole-genome RNAi screen for modifiers of JNK activation to be performed in any metazoan. We specifically addressed the regulation of JNK activation in the context of innate immunity. We believe that *Drosophila* S2 cells present an ideal system for the study of the JNK signal transduction pathway, as S2 cells are readily accessible to large-scale RNAi screens, reproduce key elements of the *Drosophila* innate immune response and serve as a convenient gateway for whole animal studies in the genetically tractable *Drosophila melanogaster*. Given the evolutionary conservation of the JNK signal transduction pathway, we believe that our studies are of direct relevance to JNK activity in the immune response of higher organisms.

We also consider it likely that we have serendipitously identified general regulators of the JNK pathway with roles that extend beyond immune signaling. For example, we identified core elements of the JNK activation cassette such as *misshapen* (*msn*, M4K ortholog), *hemipterous* (*hep*, MKK4 orthologs) and *dMKK7* (MKK7 ortholog) as required for activation of dJNK in the IMD pathway. A recent RNAi-based survey of four hundred eighty two *Drosophila* genes identified seventy seven core JNK pathway regulators [48]. Specifically, the authors detected gene products that modified basal dJNK phosphorylation levels in a number of genetically compromised backgrounds. In our assay, we excluded six of these JNK modifiers from analysis as they caused a significant depletion of f-actin. Of the remaining seventy one gene products, twenty three were significant modifiers of PGN-mediated dJNK phosphorylation (Figure S1). Thus, despite the large differences between both screens, we noticed a considerable overlap in our identification of dJNK modifiers.

We consider the false negative rate for IMD pathway members a more pertinent measure of the success of our screen. In contrast to previous RNAi screens of signal transduction pathways, our assay did not rely on indirect reporter assays. Instead, we measured the contribution of each annotated gene within the fly genome to the IMD-responsive phosphorylation of dJNK. We believe that the direct quantitative nature of our assay combined with the ease of RNAi in S2 cells greatly minimizes the likelihood of false negatives in the primary screen. Indeed, preliminary analysis of our primary screen data identified the bulk of the IMD signal transduction pathway (PGRP-LC, Imd, dFADD, Dredd, Pirk, dTAB2, dIAP2, dTAK1, dMKK4/7, dJNK, dFos, Key, Ird5 and Rel) as essential modifiers of JNK activation in the IMD pathway. In each case, the phenotype was consistent with the established molecular function of the respective IMD pathway element as either negative or positive modifiers of JNK activation. Thus, we are satisfied that false negatives do not obfuscate interpretation of our data in any meaningful manner. Ironically, the only anticipated hit we failed to identify was dJun [49].

The *Drosophila* receptor tyrosine kinase Pvr shows considerable similarity to members of the mammalian PDGF and VEGF receptor families and Pvr is considered an evolutionary ancestor of PDGF/VEGF receptors [38]. Pvr is activated in a partially redundant manner by three PDGF/VEGF-type ligands, Pvf1-3 [37],[38],[42],[50]. Initial studies implicated Pvr as a guidance receptor for cell migratory cues in embryonic hemocyte migration, oocyte border cell migration, thorax closure and dorsal closure of male terminalia [36],[37],[38],[39],[42]. The molecular basis for Pvr-mediated cell movements requires clarification. While functional redundancies appear to exist between individual Pvf ligands, several studies indicate a potential preference for Pvf-1 in the guidance of cell migration [40],[42]. In thorax closure and border cell migration, migratory cues proceed through the Pvr adaptor proteins Mbc, Ced-12 and Crk [36],[39]. In the case of thorax closure and dorsal closure of male genitalia it appears that Pvr induces the corresponding cell movements through the JNK pathway. Thus, Pvr appears to be a positive regulator of JNK activity in the context of cell movements. This is logical given the extensive involvement of JNK in the coordination of cell migration during development. However, our data strongly indicate that Pvr is a negative regulator of JNK activity during immune signaling. We did not detect any requirements for Mbc, Ced-12 or Ckr in the regulation of innate immune signaling. These data suggest that distinct adaptor molecule configurations may discriminate between the impacts of Pvr on immune responses and cell migration.

In addition to requirements for Pvr in cell migration, a parallel body of literature indicates a distinct function for Pvr in the regulation of hemocyte proliferation. The disruptions to embryonic hemocyte migration in *pvr* mutants were originally interpreted to indicate that Pvr detects migratory guidance cues in hemocytes [37]. More recent studies demonstrated that expression of the anti-apoptotic p35 molecule in the hemocytes of *pvr* mutants rescues the majority of the migratory phenoptye [51]. Further studies confirmed that the bulk of the *pvr* hemocyte phenotype is the result of cell death and that there are only minor guidance requirements for Pvr in hemocyte migration. Pvr activates the dERK pathway, which induces hemocyte proliferation [51],[52]. Consistent with a role for Pvr in hemocyte proliferation, overexpression of Pvf2 drives massive hemocyte proliferation *in vivo* and incubation of embryonic mbn-2 hemocytes with Pvr antibodies blocks cellular proliferation in a dose-dependent manner [50]. In contrast, overexpression of Pvf-1 did not substantially alter hemocyte proliferation *in vivo* and a recent study indicated that proliferative signals for hemocytes are preferentially provided by Pvf2 and Pvf3 [52]. In this context, we consider it particularly striking that our data reveal that signal transduction through the IMD pathway results in dJNK-mediated expression of Pvf2 and Pvf3.

Our study reveals a novel role for the Pvr/dERK pathway in the attenuation of the IMD pathway and illuminates our understanding of the network of regulatory checks and balances that fine tune the level of IMD/dJNK activity. Our data are most consistent with a model whereby activation of the IMD pathway results in dJNK-dependent expression of the Pvr ligands Pvf2 and Pvf3. Pvr then signals through dERK to negatively regulate the IMD pathway. On a molecular level, our data show that Pvr signaling dampens the dTAK1-dependent phosphorylation of dJNK and Rel. However, we believe that our data may also uncover an additional physiological role for Pvr. We speculate that the infection-driven production of Pvf2 and Pvf3 engages Pvr receptors on hemocytes and thereby stimulates the Ras/dERK-responsive proliferation of hemocytes. Such an increase in hemocytes numbers would provide a timely measure for the phagocytic elimination of invading extracellular microbes at early stages of infection.

We find it intriguing that proliferative signals inhibit activation of immune pathways. It may be that both processes require major metabolic commitments and that hemocytes preferentially reserve resources for proliferation. An alternative and non-exclusive hypothesis reflects the primary role of *Drosophila* hemocytes in immunity. Hemocytes are the major phagocytic cell type in *Drosophila* and are ideally suited for the engulfment of extracellular microbes. We consider it possible that induction of immune responses drives Pvr-mediated proliferation of hemocytes to facilitate rapid neutralization of extracellular microbes through phagocytosis. In this situation, it is advantageous for proliferative signals to suppress JNK activation, as hyper or prolonged activation of JNK in *Drosophila* often results in cell death. Preliminary data in our lab suggest that links between Pvr and immune signaling may be evolutionarily conserved, as we detected suppression of NF-κB activity through the PDGF receptor superfamily member c-Kit in human cell culture assays (Anja Schindler and Edan Foley, unpublished).

## Materials and Methods

### Cell Culture

*Drosophila* S2 cells and KC167 cells were cultured at 25°C in HyQ TNM-FH medium (HyClone) supplemented with 10% heat inactivated fetal bovine serum (Invitrogen), 50U/ml of penicillin and 5 µg/ml of streptomycin (GIBCO). Serum-free S2 cells were incubated in SFX-INSECT medium (HyClone) supplemeted with 50U/ml of penicillin and 5 µg/ml of streptomycin (GIBCO). PGN-dependent dJNK activation was inhibited in 10^6^ S2 cells in 1ml of culture media with the addition of 25µM SP600125 for 1h prior to PGN-exposure.

### RNAi

The dsRNA library employed in this screen is an extension of a partial-genome library described previously [53]. The remainder of the library was purchased from Open Biosystems. In-Cell Western quantitative analysis was carried out as described in [54]. Briefly, S2 cells were incubated at 1.5×10^5^ cells/well in 96 well plate in 20% conditioned media and 80% serum-free culture media with 10µg/ml dsRNAs at 25°C for three days. Cells were exposed to 50µg/ml LPS (Sigma) containing contaminating amounts of PGN for 15 or 60 min. Cell were washed with PBS, fixed in PBS + 3.7% formaldehyde, permeablized in PBS + 0.1% Triton-X 100 and blocked in blocking buffer (LI-COR Biosciences). Cells were probed with mouse anti-active-JNK (Cell Signaling) and washed with PBS + 0.1% Tween-20. P-JNK staining was detected with fluorescently labeled goat anti-mouse secondary antibodies and f-actin was stained with fluorescently labeled phalloidin (Invitrogen). Cells were washed in PBS + 0.1% Tween and P-JNK and f-actin levels were quantified with an Aerius automated imaging system (LI-COR Biosciences) following the manufacturers recommendations. In secondary ICW analyses P-JNK was monitored relative to JNK by replacing phalloidin staining with rabbit anti-JNK (Santa Cruz Biotechnology) and fluorescently labeled goat anti-rabbit antibodies.

### Data Analysis

For the RNAi screen, the raw fluorescent trimmed mean level was determined for P-JNK and f-actin channels in each well and the relative P-JNK:f-actin value was calculated. We applied z-score analysis to normalize P-JNK:f-actin values across the entire screen. Z-scores were calculated by subtracting the sample value by the plate median value and dividing by the plate standard deviation. The z-score assumes normal distribution and represents the standard deviation of every P-JNK:f-actin value from the plate median for each dsRNA treatment. Z-scores above 2.58 or below −2.58 represent the 99% confidence interval and z-scores above 1.96 or below −1.96 represent the 95% confidence interval. The f-actin z-scores were also calculated for every well on each plate and dsRNA treatments resulting in f-actin z-scores below −2.58 (99% CI) were excluded from further analysis to eliminate actin modifiers and lethal dsRNAs. We considered dsRNAs that modified P-JNK:actin z-scores outside the 95% confidence interval as hits in the screen. To identify genetic or physical interactions among hits from our screen, all hits were probed in the *Drosophila* interactions database [55] and visualized with the IM browser (http://www.droidb.org/IMBrowser.jsp). For analysis of *att* expression in the infection model, the ΔCt values were standardized to an internal control between qRt-PCR runs. The triplicate 0h ΔCt values were averaged and the ΔΔCt values were calculated relative to these values. The fold change was calculated for each sample and the 0h time point was set to one for each fly line. The SEM was calculated for each time point. Statistical significance of experimental values was expressed as p-values of less than .01 (**) or .05 (*), as calculated by a Student's t-test. We performed two-tailed Student's t-tests with two-samples of equal variance to calculate a p-value of experimental values relative to control values.

### Western Blot

Western blot analysis was performed on 10^6^ cells lysed in sample buffer, vortexted and incubated at 95°C for 5min. Proteins were separated by SDS-PAGE electrophoresis and were transfer to nitrocellulose membrane by semidry transfer. Membranes were blocked in blocking buffer (LI-COR Biosciences) and probed with mouse anti-active-JNK (Cell Signailing), rabbit anti-JNK (Santa Cruz Biotechnology), rabbit anti-pan-actin (Cell Signaling), mouse anti-actin (Sigma), rabbit anti-active MAPK1/2 (Upstate), mouse anti-HA (Sigma) or rat anti-Pvr. Western blot analysis of P-Rel and Rel cleveage was performed as described in [28]. All secondary antibodies were purchased from Invitrogen. Proteins levels were quantified with an Aerius automated imaging system (LI-COR Biosciences) following the manufacturers recommendations.

### Quantitative Real-Time PCR

Antimicrobial peptide production was monitored in S2 cells and flies by qRT-PCR. Total RNA was extracted from 10^6^ S2 cells or 10 adult flies using Trizol (Invitrogen) following the manufacturers instructions. cDNA was created from 2µg of RNA using Superscript III (Invitrogen) and oligo-dT primers (Invitrogen), according to the manufacturers instructions. We monitored transcript amplification with a Realplex 2 PCR machine (Eppendorf) using SYBR green as a detection reagent (Invitrogen). We used the following primers to monitor the expression of the corresponding gene products; *actin* forward 5′-TGCCTCATCGCCGACATAA-3′, *actin* reverse 5′-CACGTCACCAGGGCGTAAT-3′; *att* forward 5′-AGTCACAACTGGCGGAC-3′, *att* reverse 5′-TGTTGAATAAATTGGCATGG-3′; *dipt* forward 5′-ACCGCAGTACCCACTCAATC-3′, *dipt* reverse 5′-ACTTTCCAGCTCGGTTCTGA-3′; *pvf1* forward 5′-GCGCAGCATCATGAAATCAACCG-3′, *pvf1* reverse 5′-TGCACGCGGGCATATAGTAGTAG-3′; *pvf2* forward 5′-TCAGCGACGAAACGTGCAAGAG-3′, *pvf2* reverse 5′-TTTGAATGCGGCGTCGTTCC-3′; *pvf3* forward 5′-AGCCAAATTTGTGCCGCCAAG-3′, pvf3 reverse 5′- CTGCGATGCTTACTGCTCTTCACG-3′. All transcript expression values were normalized to actin and were quantified relative to a control using the ΔΔCt method.

### Inhibition/Activation of Pvr

We depleted Pvr from S2 cells with two non-overlapping dsRNAs. We designed the following primers to amplify the associated dsRNA template DNA in a two step PCR using 5′-GGGCGGT-3′ as an anchor sequence; Pvr1 forward 5′-GGGCGGGTGATGACTACATGGAGATGAGCC-3′, Pvr1 reverse 5′-GGGCGGGTATACCTTCGTTGCTCCTTCTCG-3′; Pvr2 forward 5′-GGGCGGGTCTCCTGATTTTGCGGATCTC-3′, reverse 5′-GGGCGGGTGTCTTGGGATCGGTTCTTGA-3′; GFP forward 5′-GGGCGGGTACGTAAACGGCCACAAG-3′, GFP reverse 5′-GGGCGGGTCTCAGGTAGTGGTTGTC-3′. We performed a second PCR amplification of anchor-tagged template DNA with the T7 promoter containing primer 5′-TAATACGACTCACTATAGGGAGACCACGGGCGGGT-3′. dsRNA was amplified from template DNA using T7 RNA polymerase at 25°C for 6h and annealed by cooling from 90°C to 30°C. S2 cells were depleted of Pvr using Pvr1 dsRNA unless stated otherwise. 10^6^ S2 cells were treated with dsRNA for 4 days in 1ml culture media to deplete Pvr. The Pvr pathway was activated in S2 cell using 1∶1 dilution of fresh culture media in conditioned media (CM) collected from 4 day cultures of KC167 cells. Pvr dependent dERK phosphorylation was inhibited in 10^6^ S2 cells in 1ml of culture media with the addition of 50µM PD98059 for 1h prior to CM exposure.

### Fly Husbandry

*Drosophila* strains were cultured on standard cornmeal medium (http://flystocks.bio.indiana.edu/Fly_Work/media-recipes/bloomfood.htm) at 25°C. *hs-gal4* flies were obtained from Dr. Sarah Hughes and *uas-PvrIR* flies were obtained from the Vienna *Drosophila* RNAi Center. For *in vivo* knock down of Pvr, *UAS-PvrIR* flies were crossed with *hs-gal4* flies or *w^118^* flies. 1 day old flies were heat-pulsed eight times at 37°C for 1h to initiate the expression of the RNAi construct and returned to 25°C for 5h over 48 hours. Infection was monitored in flies that were either uninjured (control), or pricked with a tungsten needle dipped in a pellet of DH5α *E. coli* bacteria (infection).

## Supporting Information

Figure S1 P-JNK screen comparative analysis. Comparative analysis of 15min and 60min PGN-induced P-JNK:f-actin screen results relative to Bakal C. et. al. 2008 [48]. 15min and 60min P-JNK:f-actin z-scores were ordered from highest to lowest and organized according to confidence intervals. Genes identified as modifiers of dJNK activity in Bakal C. et. al. 2008 were cross referenced with the PGN-induced percent P-JNK:f-actin at 15min and 60min. Genes indicated in more red demonstrated a stronger suppressive phenotype on PGN-induced JNK phosphorylation, while gene indicated in more green demonstrated a stronger enhancing phenotype on PGN-induced dJNK phosphorylation. Vertical lines indicate genes that suppress JNK phosphorylation in the top 95th percentile and genes that enhance JNK phosphorylation in the bottom 5th percentile.
(0.50 MB TIF)

Table S1 z-score analysis of dsRNA effects on 15 and 60 min PGN-induced dJNK phosphorylation. In-cell Western z-scores were calculated from P-JNK:f-actin values from S2 cells incubated with 15,683 dsRNAs and treated with PGN for 15 or 60 min. Z-scores are ordered from highest to lowest 15 min z-score. dsRNAs that modified f-actin levels below the 95% at 15 min PGN treatment were excluded. Each dsRNA is identified by its Celera Genome (CG) number or by its Heidelberg Drosophila Consortium identification number (HCDID) and general function.
(0.96 MB PDF)

Table S2 z-score analysis of dsRNA-mediated depletion of enhancers of PGN-induced dJNK phosphorylation. In-cell Western z-scores were calculated from P-JNK:f-actin values from S2 cells incubated with 15,683 dsRNAs and treated with PGN for 15 min. dsRNAs that modified P-JNK:f-actin z-scores below 1.96 (95% CI) are ordered from smallest to highest z-score. The fold change in dJNK phosphorylation relative to the plate median is shown alongside the z-score values. Each dsRNA is identified by its symbol and Celera Genome (CG) number or by its Heidelberg Drosophila Consortium identification number (HCDID).
(0.33 MB DOC)

Table S3 z-score analysis of dsRNA-mediated depletion of suppressors of 15 min PGN-induced dJNK phosphorylation. In-cell Western z-scores were calculated from P-JNK:f-actin values from S2 cells incubated with 15,683 dsRNAs and treated with PGN for 15 or 60 min. dsRNAs that modified 15 min PGN-induced P-JNK:f-actin z-scores above 1.96 (95% CI) are ordered from highest to lowest z-score. The fold change in dJNK phosphorylation relative to the plate median is shown alongside the z-score values for both 15 and 60 min time points. Each dsRNA is identified by its symbol and Celera Genome (CG) number or by its Heidelberg Drosophila Consortium identification number (HCDID).
(1.05 MB DOC)

Table S4 z-score analysis of dsRNA-mediated depletion of suppressors of 60 min PGN-induced dJNK phosphorylation. In-cell Western z-scores were calculated from P-JNK:f-actin values from S2 cells incubated with 15,683 dsRNAs and treated with PGN for 15 or 60 min. dsRNAs that modified 60 min PGN-induced P-JNK:f-actin z-scores above 1.96 (95% CI) are ordered from highest to lowest z-score. The fold change in dJNK phosphorylation relative to the plate median is shown alongside the z-score values for both 15 and 60 min time points. Each dsRNA is identified by its symbol, Celera Genome (CG) number, Heidelberg Drosophila Consortium identification number (HCDID) and general function.
(0.50 MB DOC)
