# Supplementary figures and images for: A Quantitative RNAi Screen for JNK Modifiers Identifies Pvr as a Novel Regulator of Drosophila Immune Signaling
Source: PLoS Pathog. 2009 Nov 6;5(11):e1000655. doi: 10.1371/journal.ppat.1000655 (PMC2766254; doi:10.1371/journal.ppat.1000655)

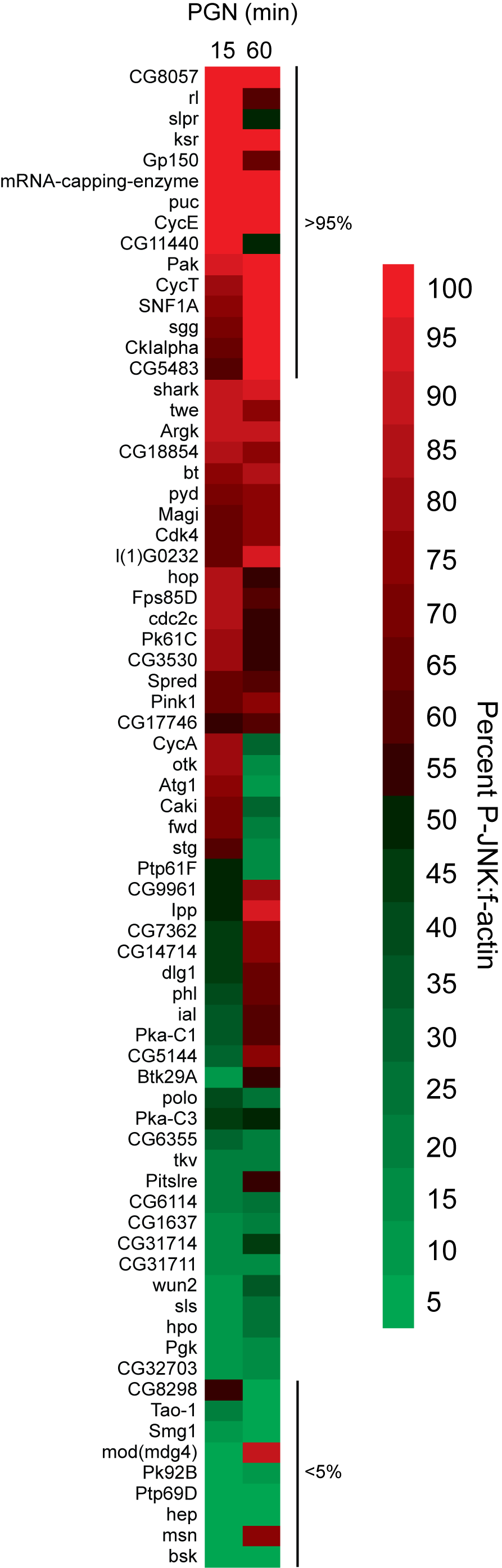

Supplement: Figure S1 — P-JNK screen comparative analysis. Comparative analysis of 15min and 60min PGN-induced P-JNK:f-actin screen results relative to Bakal C. et. al. 2008 [48]. 15min and 60min P-JNK:f-actin z-scores were ordered from highest to lowest and organized according to confidence intervals. Genes identified as modifiers of dJNK activity in Bakal C. et. al. 2008 were cross referenced with the PGN-induced percent P-JNK:f-actin at 15min and 60min. Genes indicated in more red demonstrated a stronger suppressive phenotype on PGN-induced JNK phosphorylation, while gene indicated in more green demonstrated a stronger enhancing phenotype on PGN-induced dJNK phosphorylation. Vertical lines indicate genes that suppress JNK phosphorylation in the top 95th percentile and genes that enhance JNK phosphorylation in the bottom 5th percentile. (0.50 MB TIF) [file ppat.1000655.s001.tif]
