# Supplementary material for: A Quantitative RNAi Screen for JNK Modifiers Identifies Pvr as a Novel Regulator of Drosophila Immune Signaling
Source: PLoS Pathog. 2009 Nov 6;5(11):e1000655. doi: 10.1371/journal.ppat.1000655 (PMC2766254; doi:10.1371/journal.ppat.1000655)
Supplement: Table S1 — z-score analysis of dsRNA effects on 15 and 60 min PGN-induced dJNK phosphorylation. In-cell Western z-scores were calculated from P-JNK:f-actin values from S2 cells incubated with 15,683 dsRNAs and treated with PGN for 15 or 60 min. Z-scores are ordered from highest to lowest 15 min z-score. dsRNAs that modified f-actin levels below the 95% at 15 min PGN treatment were excluded. Each dsRNA is identified by its Celera Genome (CG) number or by its Heidelberg Drosophila Consortium identification number (HCDID) and general function. (0.96 MB PDF) [file ppat.1000655.s002.pdf]

Table S1. P-JNK:f-actin z-score values for 15 and 60 minutes PGN-exposure.

CG	HDCID	15 min z-score	60 min z-score
16910		9.06	9.23
	3194	7.72	-0.33
7392		7.70	7.20
11526		7.35	4.67
	12197	7.11	-1.10
4201		6.23	7.74
11799		5.89	1.56
6647		5.88	5.50
	6860	5.85	-0.53
5461		5.75	2.05
15732		5.72	-0.74
2086		5.56	
8346		5.37	2.54
3180		5.37	2.74
17077		5.32	-1.34
14314		5.24	1.76
6556		5.20	3.63
17603		5.08	0.38
5575		5.07	3.57
	2525	5.04	0.61
12264		5.02	-0.49
8222		5.01	1.77
	2579	5.01	-0.59
3403		5.00	0.24
9038		4.96	6.13
10936		4.92	0.57
4027		4.90	4.28
4918		4.80	1.75
11848		4.78	-0.66
15321		4.76	0.71
	9403	4.73	1.68
31992		4.71	0.70
	12021	4.69	0.91
8828		4.69	1.46
9291		4.69	0.59
32681		4.66	0.25
15678		4.63	7.28
10798		4.62	1.06
	8340	4.61	-0.04
8954		4.59	2.12
9864		4.57	-0.08
13083		4.57	-0.89

4479		4.57	1.52
10158		4.57	3.88
1554		4.52	1.65
2175		4.47	-0.90
7873		4.31	2.30
	5820	4.24	-1.12
	10149	4.24	2.05
	113	4.24	-0.18
	2195	4.23	1.40
9709		4.23	1.57
8491		4.23	2.05
3477		4.23	0.64
13628		4.23	-0.82
	6790	4.20	-0.92
7481		4.20	3.12
4729		4.19	4.63
12263		4.17	1.96
12002		4.16	2.38
9493		4.12	1.54
15674		4.07	0.75
4320		4.07	3.48
5546		4.06	2.40
12663		4.06	-1.23
2086		4.03	2.58
14226		3.99	1.64
15899		3.96	-1.47
3176		3.94	-1.04
18285		3.93	1.43
8556		3.93	1.48
13363		3.92	4.00
7476		3.89	0.28
5954		3.88	0.03
8446		3.86	0.84
1163		3.86	1.16
14999		3.83	0.67
	5815	3.81	-1.31
	11835	3.81	0.06
4087		3.80	1.38
32000		3.76	3.14
32690		3.75	-1.10
3891		3.70	2.55
17233		3.70	-0.19
5360		3.67	1.26
31047		3.66	0.84
	17826	3.65	1.41

6280		3.65	0.27
18009		3.64	2.18
10189		3.63	-0.85
	10185	3.63	1.40
4119		3.62	2.24
14782		3.61	-0.03
11132		3.61	1.91
	17828	3.60	1.19
32211		3.59	1.61
17894		3.58	2.70
11984		3.57	2.69
31049		3.57	1.65
	4702	3.56	0.66
6500		3.56	2.96
	13104	3.51	0.22
5757		3.51	0.50
6840		3.49	1.28
32920		3.49	1.00
9663		3.48	0.36
	888	3.48	1.43
8771		3.48	2.99
1689		3.47	0.54
5838		3.47	0.72
	19487	3.46	1.61
31739		3.46	-0.08
10225		3.45	1.83
	403	3.45	-1.56
	897	3.45	-0.14
9375		3.44	0.72
	8321	3.44	0.25
12157		3.42	1.32
32180		3.42	2.51
15121		3.42	-0.76
10851		3.42	2.63
12341		3.41	0.04
14313		3.41	4.46
1560		3.40	1.59
6735		3.40	2.16
5057		3.39	0.72
	9508	3.38	0.65
9954		3.38	2.03
12923		3.36	0.14
9304		3.36	4.28
	8330	3.35	-0.16
3992		3.35	

8517		3.33	0.62
	14726	3.33	-0.19
	9514	3.33	-0.74
15119		3.32	-1.27
3998		3.31	-0.22
9983		3.31	0.98
8057		3.31	1.84
	2034	3.30	1.24
15324		3.28	-1.28
10067		3.28	4.28
	14831	3.27	1.35
17358		3.27	-0.24
1910		3.26	1.62
5785		3.25	-0.89
9394		3.24	1.66
10541		3.24	0.53
	3047	3.24	-0.61
7808		3.23	0.33
12050		3.22	3.17
16916		3.20	-1.13
1250		3.19	-0.29
	10130	3.19	1.92
32168		3.19	3.41
32817		3.18	-0.57
9623		3.18	0.59
3476		3.18	0.90
4631		3.17	-0.14
14160		3.16	1.55
16865		3.15	1.22
11245		3.15	2.01
6134		3.15	1.12
13609		3.14	-0.56
30217		3.13	1.20
13802		3.13	1.61
7177		3.12	2.33
	9397	3.12	1.38
	11912	3.12	-1.49
	497	3.12	1.09
11321		3.12	1.48
1244		3.10	2.40
13675		3.09	0.55
7773		3.09	-0.38
3731		3.08	1.00
5746		3.08	0.06
13780		3.06	-0.96

15509		3.06	3.56
9895		3.06	-0.84
18545		3.05	2.74
14722		3.04	1.71
	17852	3.04	1.35
7727		3.01	1.72
11227		3.01	0.85
12681		3.01	-0.62
5760		3.00	0.57
9523		3.00	2.69
2083		2.99	0.22
5502		2.99	1.98
8260		2.98	0.12
2652		2.96	-1.90
5479		2.96	3.05
	17815	2.95	0.33
	14028	2.95	1.23
13845		2.95	0.22
5837		2.95	-0.02
4896		2.94	2.69
31394		2.94	0.58
	19488	2.94	1.49
33296		2.94	1.47
32104		2.94	-0.21
1553		2.94	-0.74
15488		2.94	1.89
	14817	2.93	2.81
12051		2.92	4.13
30034		2.92	0.21
4636		2.90	1.50
31369		2.89	0.35
18166		2.88	-0.58
	9406	2.88	0.22
9122		2.87	0.64
1772		2.87	0.55
3777		2.87	0.63
	7441	2.87	-1.35
5409		2.87	1.57
4612		2.87	1.32
12254		2.85	
13519		2.85	0.95
	15864	2.85	0.90
	12184	2.84	1.07
3619		2.84	3.40
	13103	2.83	0.29

	8318	2.83	-0.35
12255		2.83	1.05
4798		2.82	2.44
	7043	2.82	0.54
13041		2.81	-1.28
9019		2.81	-0.69
16817		2.80	1.93
6369		2.80	1.54
6187		2.79	1.82
4913		2.78	0.99
	7585	2.78	0.57
11121		2.78	1.15
8509		2.78	1.76
	6322	2.78	2.10
15006		2.77	0.84
	17406	2.77	0.48
1276		2.76	2.90
9730		2.75	1.23
	14730	2.74	-1.37
6215		2.74	0.67
5800		2.73	0.06
9862		2.72	-0.04
4153		2.72	1.18
11843		2.72	0.78
2872		2.71	-3.14
5119		2.71	4.48
9324		2.71	5.65
15185		2.71	1.51
3416		2.71	1.92
18446		2.71	-0.11
	19486	2.70	1.30
10320		2.70	0.33
12175		2.70	1.07
4993		2.70	0.47
11260		2.70	-0.55
8885		2.70	-0.09
	17312	2.70	1.30
7366		2.69	-0.01
33346		2.69	1.00
1787		2.69	2.39
3838		2.68	-3.08
	10201	2.68	0.56
	13864	2.68	-0.05
	15843	2.68	1.03
6615		2.68	1.94

12744		2.67	0.86
	19511	2.67	0.01
	14841	2.67	0.39
15326		2.66	-1.17
12047		2.65	0.96
3849		2.65	0.40
17632		2.64	-0.32
	8316	2.63	-0.69
12582		2.62	0.59
13358		2.62	0.01
31696		2.61	1.11
30196		2.61	1.31
10990		2.60	0.60
32058		2.60	1.49
	7692	2.59	-0.81
11100		2.59	1.68
13047		2.59	2.01
	13099	2.59	0.06
15909		2.59	-0.96
7558		2.58	3.28
12091		2.58	0.03
9533		2.58	-0.61
6674		2.58	1.18
30084		2.58	1.48
12212		2.57	0.90
31712		2.56	0.13
32054		2.56	0.57
	14728	2.56	0.03
10630		2.56	0.80
2556		2.55	0.63
3329		2.55	1.08
11422		2.55	0.73
4931		2.54	1.90
	19504	2.54	0.58
14685		2.54	1.34
9293		2.52	0.51
8223		2.52	1.62
10370		2.51	0.17
8523		2.51	0.15
9973		2.50	1.30
6338		2.50	0.15
8042		2.50	-0.96
8066		2.50	1.30
15894		2.50	1.07
12559		2.50	0.13

5266		2.49	1.58
	17817	2.49	0.19
32755		2.49	-0.35
2013		2.48	1.08
6685		2.48	0.85
31607		2.48	0.88
5099		2.48	1.90
33203		2.47	1.69
33484		2.47	2.36
	12190	2.47	0.35
1973		2.46	1.46
5258		2.46	1.97
	6940	2.46	0.37
6577		2.46	0.63
33187		2.46	1.22
14194		2.46	0.71
32946		2.45	0.49
31298		2.45	0.84
	17821	2.45	-0.49
3069		2.45	1.52
	6591	2.44	0.52
12920		2.44	-0.85
	6795	2.44	-0.91
5263		2.43	2.15
12119		2.43	0.46
4396		2.43	0.71
	33	2.42	-2.48
2272		2.42	-0.09
	14799	2.42	-0.51
	14842	2.41	0.07
10752		2.41	2.20
7763		2.41	-1.23
7128		2.40	1.16
8501		2.40	0.51
10149		2.40	5.02
8937		2.40	0.97
6993		2.40	3.91
	16223	2.39	0.06
	11436	2.39	0.58
	13111	2.39	-0.31
13338		2.39	0.12
	6912	2.39	-1.56
4367		2.39	-0.17
2331		2.38	1.63
11575		2.38	0.87

32956		2.38	1.23
33297		2.38	-0.22
18266		2.38	0.71
2899		2.38	2.01
12069		2.37	0.12
11486		2.37	0.35
32368		2.36	-0.07
9603		2.36	-0.17
3528		2.36	1.46
8749		2.36	-0.54
15015		2.36	1.65
	19521	2.36	0.30
4615		2.36	0.57
12773		2.35	1.33
13530		2.35	1.76
13932		2.35	0.24
14801		2.35	0.41
	20230	2.35	0.01
7371		2.34	1.82
3524		2.34	0.02
17265		2.34	-2.31
30264		2.34	1.76
6009		2.33	1.02
6006		2.33	2.79
3733		2.33	0.23
11023		2.33	2.56
12237		2.33	-0.70
33150		2.32	1.74
16992		2.32	-0.01
9650		2.32	1.75
9346		2.32	0.08
7678		2.32	0.04
2788		2.32	1.25
6023		2.32	1.22
1746		2.31	0.77
	7586	2.31	1.08
31044		2.31	0.67
8710		2.31	0.54
14998		2.31	2.27
	19522	2.30	0.25
13198		2.30	-0.23
7876		2.30	-0.81
32217		2.29	2.74
13564		2.29	0.61
1793		2.29	2.59

	16885	2.29	1.50
14520		2.28	1.13
9350		2.28	0.48
3455		2.28	2.28
12598		2.28	-0.12
	9478	2.28	1.75
8290		2.27	1.19
32646		2.27	1.68
1906		2.26	0.48
	19473	2.26	0.96
	7589	2.26	1.29
3227		2.26	2.29
7349		2.26	-0.40
9757		2.25	0.45
1245		2.25	0.76
3806		2.24	-0.42
	14720	2.24	-0.49
1019		2.24	1.09
5330		2.24	-0.70
	2627	2.24	-0.37
8715		2.23	1.34
10079		2.23	1.67
30176		2.23	0.44
1810		2.23	4.60
14683		2.23	1.39
5820		2.23	0.23
12842		2.23	0.79
18397		2.22	0.19
5452		2.22	2.34
1458		2.22	0.85
	12145	2.22	1.33
2615		2.22	2.99
15263		2.22	1.74
14972		2.22	0.66
	13135	2.21	-0.29
8877		2.21	0.15
7181		2.21	1.31
10419		2.21	1.61
14853		2.20	0.74
13283		2.19	1.27
12584		2.19	0.44
32673		2.19	-1.04
32148		2.19	1.03
10658		2.19	0.81
3934		2.19	-0.21

13288		2.18	0.77
2082		2.18	0.13
14909		2.18	-0.73
32053		2.18	0.65
5375		2.18	1.66
5454		2.18	1.22
7851		2.18	5.58
4264		2.18	0.85
	17108	2.17	1.28
15403		2.17	1.09
3730		2.17	1.56
11971		2.17	1.81
3708		2.17	0.26
18748		2.17	0.69
6474		2.17	3.33
3250		2.17	0.77
3713		2.16	-0.01
33151		2.16	1.25
31238		2.16	1.19
7850		2.16	5.23
	5276	2.15	0.26
32638		2.15	1.50
5048		2.15	0.68
5927		2.15	0.27
	18862	2.15	1.17
	3592	2.14	0.92
3616		2.14	1.75
12493		2.13	-0.15
33339		2.13	0.54
	16673	2.13	-1.33
	13081	2.12	-0.82
32102		2.12	1.42
9496		2.12	0.11
32365		2.12	-0.44
15627		2.12	0.75
3017		2.11	1.32
32428		2.11	1.70
9098		2.11	0.04
6121		2.10	2.57
	5924	2.10	0.34
33309		2.10	0.53
30495		2.10	-0.92
	4697	2.10	1.54
7498		2.10	0.51
4207		2.10	-0.29

	15882	2.09	0.82
14305		2.09	1.15
4461		2.09	1.30
32745		2.09	-0.90
15792		2.09	1.13
18586		2.09	-1.11
	17872	2.09	0.27
16788		2.08	0.78
30270		2.08	0.97
31427		2.08	0.73
6671		2.08	0.13
	6022	2.08	1.35
	8262	2.08	-1.04
9131		2.07	2.45
13474		2.07	-0.22
7931		2.07	-0.50
1287		2.07	0.97
9900		2.07	-1.57
33260		2.07	0.12
8856		2.07	1.60
5742		2.07	0.63
9556		2.07	1.27
5107		2.06	-1.18
17855		2.06	0.33
14635		2.06	0.46
15256		2.06	1.30
8550		2.06	1.63
4097		2.06	1.74
	7058	2.06	-0.29
14545		2.06	0.52
9086		2.06	0.48
17666		2.06	1.11
32800		2.06	-1.07
13167		2.05	-1.47
31787		2.05	0.37
7762		2.05	1.09
	14836	2.05	0.13
11202		2.05	0.21
32464		2.05	0.57
3234		2.05	1.18
31423		2.05	0.77
12581		2.05	0.77
9822		2.05	0.55
3065		2.04	1.92
6479		2.04	1.18

13800		2.04	1.76
15444		2.04	0.00
5444		2.04	2.36
9641		2.04	1.03
8310		2.04	1.94
5289		2.03	1.01
3050		2.03	1.68
6124		2.03	1.17
31936		2.03	0.65
31638		2.03	1.24
33182		2.03	1.49
1449		2.02	0.38
9374		2.02	0.41
	7059	2.02	-0.58
2249		2.02	-0.65
14113		2.01	1.44
30265		2.01	1.94
14223		2.01	-0.06
31128		2.01	1.07
32016		2.01	-0.87
2079		2.01	-1.06
17612		2.01	-0.33
14384		2.00	-0.12
	9412	2.00	0.04
14469		2.00	2.02
3034		2.00	1.69
11872		2.00	0.28
	4700	2.00	-0.76
4629		2.00	-0.17
7885		2.00	1.68
7236		1.99	1.95
4945		1.99	-0.11
7071		1.99	1.18
	770	1.99	-0.27
5783		1.99	0.83
32937		1.99	1.37
	2900	1.99	-1.06
32297		1.99	-0.21
	12189	1.98	0.69
18505		1.98	0.73
5604		1.98	1.54
4399		1.98	1.25
10772		1.97	0.46
11110		1.97	1.66
31213		1.97	0.59

11050		1.97	0.81
32553		1.97	-0.11
14633		1.97	-0.17
8019		1.97	-0.65
13701		1.97	0.38
8120		1.97	2.12
31082		1.96	0.85
	20240	1.96	1.74
16941		1.96	0.89
5694		1.96	0.84
12031		1.96	1.35
30491		1.96	0.87
5922		1.95	-0.34
17611		1.95	0.38
7035		1.95	0.91
7903		1.95	-1.02
14651		1.95	1.66
31948		1.95	0.94
17922		1.95	-1.09
17470		1.95	0.40
5609		1.95	0.48
4059		1.95	1.73
8809		1.95	2.69
	7242	1.95	1.01
9863		1.94	-0.96
18591		1.94	0.53
12582		1.94	-0.14
	10263	1.94	-0.06
13126		1.94	-1.10
15675		1.94	0.35
14243		1.94	0.99
4374		1.94	1.19
13566		1.94	1.36
32788		1.94	1.26
5352		1.93	1.08
11628		1.93	1.06
	3160	1.93	0.93
4319		1.93	0.50
14551		1.93	0.70
18768		1.93	-1.28
17608		1.93	-0.81
10586		1.93	1.52
4257		1.93	0.60
8432		1.93	0.27
8674		1.92	0.49

8472		1.92	0.78
9207		1.92	1.09
	3444	1.92	1.25
7550		1.92	0.58
3923		1.92	1.41
12819		1.92	0.66
3801		1.92	1.71
11254		1.91	-0.04
18870		1.91	-0.36
	8561	1.91	0.01
6325		1.91	-0.28
30189		1.91	0.48
14271		1.91	1.14
13715		1.91	0.47
15863		1.90	0.43
17108		1.90	0.89
30118		1.90	0.81
5041		1.90	0.24
3189		1.90	0.56
30156		1.90	0.23
	11442	1.90	-0.10
13196		1.90	-1.08
13778		1.90	1.36
32535		1.90	-0.74
8151		1.89	-0.23
17078		1.89	-1.84
13158		1.89	0.96
	13306	1.89	1.59
13449		1.89	0.62
11073		1.89	1.83
1291		1.89	1.44
32364		1.88	-0.33
13794		1.88	0.07
14634		1.88	0.04
7704		1.88	0.10
11981		1.88	3.12
5179		1.88	1.25
8859		1.88	0.78
18581		1.87	1.13
31754		1.87	
8852		1.87	0.94
	6536	1.87	-0.08
31299		1.87	1.30
11504		1.87	-1.74
12022		1.87	-0.96

32754		1.87	-0.49
11505		1.86	2.24
2859		1.86	1.38
5755		1.86	0.38
33080		1.86	-0.31
13487		1.86	-0.14
13795		1.86	-0.91
3938		1.86	1.54
7242		1.86	0.74
11885		1.86	1.32
5468		1.85	0.98
	16899	1.85	0.82
31278		1.85	1.20
14206		1.85	0.57
5977		1.85	0.79
15877		1.85	1.59
15460		1.85	0.06
9525		1.85	1.31
7214		1.85	-2.12
	6238	1.84	0.54
30420		1.84	0.29
11979		1.84	1.32
3634		1.84	1.10
9883		1.84	0.84
5044		1.84	0.41
	20243	1.84	2.48
5282		1.84	0.55
	4665	1.84	1.79
3526		1.84	0.28
12169		1.84	2.29
4797		1.83	1.07
17885		1.83	0.80
3259		1.83	1.98
8816		1.83	-0.21
6044		1.83	0.84
11115		1.83	1.15
6684		1.83	0.90
9812		1.83	-0.10
6747		1.83	-0.06
10703		1.83	1.01
3883		1.83	1.90
9213		1.83	0.05
9297		1.83	3.32
3395		1.83	1.00
	13979	1.83	0.59

15160		1.83	0.80
2993		1.83	-0.42
10947		1.82	0.00
13461		1.82	0.56
12094		1.82	-1.11
10683		1.81	1.29
4539		1.81	1.56
1762		1.81	0.29
	16908	1.81	0.53
32885		1.81	1.11
31316		1.81	0.71
14106		1.81	-0.34
3246		1.81	1.65
11844		1.81	-0.05
3279		1.81	0.76
5433		1.81	0.86
30081		1.81	-0.31
12996		1.81	1.58
5930		1.81	0.51
	3688	1.81	-0.10
3214		1.81	0.59
18155		1.81	0.27
	3478	1.80	0.82
15673		1.80	0.45
31147		1.80	-0.38
	20237	1.80	1.18
31809		1.80	0.31
8713		1.79	1.45
	20215	1.79	0.25
1299		1.79	1.09
8539		1.79	-0.30
8084		1.79	-0.31
9750		1.79	2.88
8038		1.79	0.42
30275		1.79	0.92
5537		1.79	0.07
	7597	1.79	0.09
31324		1.79	1.80
8522		1.78	0.65
3068		1.78	2.49
	11440	1.78	0.00
33335		1.78	1.05
15056		1.78	0.60
17050		1.78	-0.91
14741		1.77	2.00

13867		1.77	1.35
13298		1.77	-0.35
8712		1.77	1.48
11436		1.77	2.77
	18422	1.77	1.09
4651		1.77	0.88
7980		1.77	1.34
3746		1.77	1.03
6792		1.77	0.02
11584		1.76	0.71
30021		1.76	-0.36
10315		1.76	0.46
2371		1.76	1.82
4766		1.76	0.73
10616		1.76	0.71
18455		1.76	2.01
	17098	1.76	1.97
	14021	1.76	0.73
2065		1.75	0.91
2637		1.75	2.62
13813		1.75	-0.12
5725		1.75	0.32
10624		1.74	0.59
2901		1.74	2.14
	13130	1.74	-0.55
33130		1.74	0.78
5215		1.74	0.73
33351		1.74	1.63
13415		1.74	-1.07
1903		1.73	0.53
	6796	1.73	-0.44
9381		1.73	0.66
	14029	1.73	0.83
10610		1.73	1.45
12679		1.73	0.24
12225		1.72	4.58
12936		1.72	0.46
17784		1.72	0.89
12117		1.72	0.14
4166		1.72	3.42
32298		1.72	1.51
10295		1.72	1.83
9259		1.71	0.52
5780		1.71	1.10
13090		1.71	-1.60

6518		1.71	0.50
7895		1.71	0.11
9310		1.71	1.70
31089		1.71	0.39
13060		1.71	-0.95
	6939	1.71	0.89
5603		1.71	0.60
7791		1.71	-0.24
5844		1.71	0.90
	2590	1.70	0.49
31390		1.70	1.87
2248		1.70	-0.96
17941		1.70	1.27
31781		1.70	0.69
31297		1.70	-0.43
2555		1.70	0.34
4076		1.70	1.19
10572		1.70	1.74
17952		1.70	2.59
11000		1.70	-0.93
4986		1.70	-0.24
2709		1.70	1.49
4300		1.69	0.81
9598		1.69	2.07
5885		1.69	0.74
17964		1.69	1.72
4422		1.69	1.49
6709		1.69	0.31
	6974	1.69	0.30
7875		1.69	-0.77
12070		1.69	-0.91
32086		1.69	-0.01
10211		1.68	-0.56
9074		1.68	0.34
13840		1.68	0.32
8180		1.68	-0.88
15450		1.68	-0.08
6329		1.68	-1.23
9642		1.68	0.39
6118		1.68	0.77
4769		1.68	-0.09
5660		1.67	0.86
5096		1.67	1.01
7251		1.67	0.93
18108		1.67	-1.01

12787		1.67	1.62
6331		1.67	0.25
1854		1.67	-1.42
3181		1.67	0.87
33234		1.66	1.30
	20222	1.66	-0.20
	9414	1.66	-0.30
15027		1.66	0.40
13939		1.66	0.34
17299		1.66	3.62
4448		1.66	1.89
14977		1.66	0.32
14984		1.66	-0.56
7911		1.66	-0.84
4402		1.66	0.11
9096		1.66	-0.94
2009		1.66	1.31
10042		1.66	0.65
	11441	1.66	0.06
14323		1.65	0.38
5569		1.65	0.41
18178		1.65	-1.75
8982		1.65	1.89
	4655	1.65	2.10
8483		1.65	-0.28
5778		1.65	1.78
10287		1.65	-0.05
18669		1.65	0.01
32310		1.65	1.38
10436		1.65	0.43
32534		1.65	-1.33
31151		1.64	-0.95
4815		1.64	0.60
5431		1.64	1.59
18525		1.64	1.10
1744		1.64	1.06
11111		1.64	1.28
10264		1.64	1.08
2715		1.64	0.48
7756		1.64	-0.24
17797		1.64	0.58
3705		1.63	-0.59
12611		1.63	-0.01
11620		1.63	-0.43
11786		1.63	1.17

8963		1.63	0.03
31484		1.63	1.24
9132		1.63	0.48
9024		1.63	1.16
31544		1.63	-1.01
15503		1.63	-0.21
32130		1.63	0.37
4272		1.63	0.88
8857		1.63	1.84
	8314	1.62	-0.10
15484		1.62	1.42
1303		1.62	0.39
13741		1.62	0.57
32590		1.62	0.77
9947		1.62	1.55
	10065	1.62	0.50
	7779	1.62	0.08
4420		1.62	1.08
10639		1.61	1.24
14216		1.61	
13605		1.61	-1.51
6584		1.61	2.14
	15889	1.61	1.37
9704		1.61	-0.38
16894		1.61	0.32
3284		1.61	0.33
31407		1.61	-0.13
6812		1.61	0.05
	8265	1.61	-0.17
10988		1.61	-2.47
10080		1.61	0.04
11339		1.61	1.08
8006		1.61	-0.79
9941		1.61	0.84
10667		1.61	0.61
31466		1.61	-0.02
8524		1.61	-1.55
31955		1.60	1.49
	15809	1.60	-1.68
17604		1.60	-0.47
14060		1.60	-0.23
9901		1.60	-0.13
14107		1.60	-0.75
9322		1.60	0.92
9866		1.60	-1.87

11421		1.60	1.22
13610		1.60	-1.09
	13700	1.60	-1.70
2321		1.60	-0.97
18048		1.60	0.57
31155		1.60	0.24
13623		1.59	-0.66
12754		1.59	1.31
5613		1.59	1.19
	4663	1.59	0.40
14581		1.59	1.70
2867		1.59	0.89
9215		1.59	0.04
13037		1.59	0.63
13604		1.59	-1.21
11198		1.59	0.39
11190		1.59	0.02
	17813	1.59	-0.80
4060		1.58	0.76
11295		1.58	0.31
5012		1.58	0.28
31264		1.58	0.91
32319		1.58	1.62
4480		1.58	1.11
18210		1.58	-1.71
6203		1.58	-0.16
1295		1.58	0.38
18341		1.58	1.47
32825		1.58	-1.13
12538		1.57	1.27
9416		1.57	1.02
11666		1.57	2.11
13151		1.57	1.35
11263		1.57	1.72
9397		1.57	1.89
	784	1.57	0.16
15784		1.57	1.20
7069		1.57	2.35
10806		1.57	1.01
	14528	1.57	-0.83
7031		1.57	0.63
6033		1.57	2.44
4593		1.57	0.12
3650		1.57	0.00
10113		1.57	-1.68

2674		1.57	1.11
31039		1.57	2.95
11142		1.57	0.85
15064		1.57	0.05
3187		1.56	1.81
5904		1.56	0.02
6919		1.56	0.61
34378		1.56	1.41
	10645	1.56	1.60
13631		1.56	1.32
7203		1.56	-2.79
7325		1.56	0.29
32073		1.56	2.80
13850		1.56	0.37
32120		1.56	0.77
	3686	1.56	-1.36
8615		1.56	2.75
	2045	1.56	1.73
17369		1.55	1.46
5864		1.55	0.50
1954		1.55	1.24
14770		1.55	1.79
3560		1.55	0.57
10812		1.55	-2.38
16869		1.55	1.06
3033		1.55	1.87
7341		1.55	0.50
14979		1.55	0.67
32060		1.55	1.32
	14054	1.55	-0.10
	7622	1.55	1.81
3869		1.55	-1.42
17947		1.55	2.39
8435		1.55	-0.45
	12718	1.55	0.71
	8264	1.55	-0.18
4008		1.55	0.28
16807		1.55	0.05
14674		1.54	1.67
2222		1.54	0.13
1975		1.54	1.09
8819		1.54	-0.29
	2648	1.54	0.39
5829		1.54	0.83
31358		1.54	-0.40

14990		1.54	-0.98
1768		1.54	2.00
9815		1.54	0.39
7728		1.54	0.07
	566	1.54	-2.15
10642		1.54	0.32
1315		1.54	0.56
8759		1.53	0.65
9935		1.53	-0.03
	9417	1.53	-0.46
4109		1.53	2.10
10467		1.53	0.39
33170		1.53	1.10
31689		1.53	-0.36
6729		1.53	1.00
31513		1.53	-0.41
	16881	1.53	1.46
13016		1.53	-1.75
8247		1.53	1.02
32267		1.53	0.26
9497		1.53	1.01
3709		1.53	0.01
18764		1.52	-0.19
3707		1.52	0.00
9393		1.52	0.33
17871		1.52	-1.16
7327		1.52	0.67
1249		1.52	0.07
18528		1.52	0.31
10592		1.52	0.51
4464		1.51	0.27
3240		1.51	0.30
1856		1.51	0.51
	19573	1.51	1.18
14667		1.51	-1.58
	468	1.51	-2.18
4012		1.51	-0.77
	12125	1.51	-1.38
	2380	1.51	-1.73
1264		1.51	-1.77
5069		1.51	1.23
14379		1.51	0.54
7014		1.51	0.33
33180		1.51	1.15
33471		1.51	-1.03

	17346	1.51	0.00
11205		1.51	0.37
2819		1.51	0.20
30128		1.50	0.09
4975		1.50	-1.63
13359		1.50	0.99
8950		1.50	-2.24
32083		1.50	1.00
15240		1.50	0.00
31237		1.50	0.16
30285		1.49	-1.22
9535		1.49	0.21
8981		1.49	0.37
9879		1.49	-0.38
33459		1.49	0.81
10107		1.49	0.88
5602		1.49	0.98
30044		1.49	0.65
17142		1.49	1.44
8695		1.49	-0.25
3242		1.49	0.12
30182		1.49	0.73
14540		1.49	-0.58
14854		1.49	-0.29
13679		1.49	-0.54
	27	1.49	-1.25
17283		1.49	0.22
11596		1.48	1.68
13128		1.48	1.02
17664		1.48	-0.37
32265		1.48	0.63
3612		1.48	0.86
	6804	1.48	0.65
15800		1.48	1.48
5219		1.48	0.20
30109		1.48	1.13
17508		1.48	0.56
1214		1.48	1.06
4877		1.48	-0.45
	7147	1.47	0.85
8629		1.47	-0.26
10757		1.47	1.90
11128		1.47	0.40
7675		1.47	0.35
	12161	1.47	-2.66

12772		1.47	-1.22
9850		1.47	1.34
31805		1.47	0.25
	12662	1.47	-1.04
4613		1.47	-0.13
5591		1.47	0.35
17533		1.47	0.03
9227		1.47	-0.25
1537		1.47	1.57
7139		1.47	0.85
7629		1.47	0.25
3075		1.47	1.75
32408		1.46	0.04
	7114	1.46	1.32
5905		1.46	0.00
9611		1.46	1.96
	19414	1.46	1.02
3399		1.46	1.15
32666		1.46	0.37
3474		1.46	1.99
	20209	1.46	-0.28
4291		1.46	1.30
	15019	1.46	0.43
	8349	1.46	-1.06
13493		1.46	0.31
6123		1.46	1.41
	6063	1.46	0.86
14980		1.46	-0.84
17958		1.45	2.04
8190		1.45	-0.26
	14069	1.45	-0.09
5799		1.45	3.25
5850		1.45	-0.95
8431		1.45	1.45
3615		1.45	1.94
5902		1.45	0.39
14422		1.45	1.64
8252		1.45	-0.58
4446		1.45	1.03
11094		1.44	1.17
5535		1.44	-1.02
14937		1.44	0.92
4772		1.44	1.04
7138		1.44	0.83
5484		1.44	1.60

	17877	1.44	0.18
13776		1.44	0.82
13499		1.44	0.33
5363		1.44	-1.36
10263		1.44	1.39
1919		1.44	1.43
17534		1.44	0.64
5620		1.44	1.56
	7045	1.43	0.22
	17310	1.43	0.26
7776		1.43	0.81
2843		1.43	1.05
31525		1.43	2.10
8427		1.43	0.60
	15018	1.43	0.42
3624		1.43	1.19
12729		1.43	1.92
8609		1.43	0.51
8067		1.43	-1.16
18468		1.43	1.19
	15841	1.43	0.65
33281		1.43	-0.88
13524		1.43	0.92
30092		1.43	0.74
3874		1.43	0.35
1404		1.43	0.60
7957		1.43	1.97
15094		1.43	1.06
	14807	1.43	1.11
17472		1.42	0.90
5594		1.42	0.19
14324		1.42	1.61
5488		1.42	
15432		1.42	0.58
17650		1.42	0.32
1489		1.42	1.63
17203		1.42	1.38
15756		1.42	1.23
6498		1.42	0.72
	9404	1.42	0.23
14173		1.42	-0.27
14207		1.42	0.68
1709		1.42	0.76
7890		1.41	0.11
6301		1.41	0.81

9425		1.41	1.86
14291		1.41	0.80
1157		1.41	0.76
5793		1.41	-0.23
	7189	1.41	0.77
17149		1.41	1.47
4478		1.41	0.96
	14703	1.41	-0.78
6287		1.41	-0.64
32628		1.41	2.87
18754		1.41	-0.15
31266		1.40	0.84
	2041	1.40	-0.19
11718		1.40	0.56
6499		1.40	0.61
15576		1.40	-0.19
5941		1.40	1.50
13810		1.40	1.54
30383		1.40	-0.09
6470		1.40	0.89
	17962	1.40	0.52
10839		1.40	-0.10
10043		1.40	0.43
13471		1.40	-0.65
6789		1.40	1.25
	8341	1.40	0.17
8416		1.40	1.55
9854		1.39	0.90
4537		1.39	0.65
33204		1.39	1.30
13838		1.39	0.63
10938		1.39	0.61
10972		1.39	0.89
13777		1.39	1.50
32953		1.39	0.25
	7552	1.39	-0.61
5791		1.39	1.64
13139		1.39	-0.52
32138		1.39	1.12
	12192	1.39	-0.02
13847		1.39	-1.45
9782		1.39	1.86
33290		1.39	1.16
32832		1.39	1.15
7363		1.39	1.45

8024		1.39	-0.88
4821		1.39	0.28
5599		1.39	1.47
4760		1.38	0.39
1124		1.38	1.56
10302		1.38	0.44
3843		1.38	-0.38
9706		1.38	0.74
30469		1.38	-1.86
15729		1.38	-0.38
1677		1.38	0.76
3066		1.38	1.46
11785		1.38	0.05
30478		1.38	0.16
3213		1.38	1.61
8625		1.38	0.77
31789		1.38	0.92
13138		1.38	0.47
5987		1.38	0.56
6593		1.38	-0.13
12267		1.38	-0.72
	14680	1.38	-0.70
9913		1.37	1.88
2087		1.37	-0.07
5582		1.37	0.55
31676		1.37	0.87
7324		1.37	1.17
10999		1.37	-1.00
4933		1.37	-0.30
2102		1.37	1.99
32254		1.37	1.63
12857		1.37	0.54
5237		1.37	0.98
7928		1.37	0.51
32853		1.37	1.24
3210		1.37	1.46
4424		1.37	1.24
17204		1.37	0.97
7930		1.37	0.15
6262		1.37	0.75
10212		1.37	-0.56
13348		1.37	-0.41
8091		1.37	1.03
14546		1.37	-0.01
31738		1.37	2.03

	14788	1.37	-2.06
15455		1.37	-0.55
10840		1.36	0.05
4849		1.36	0.41
7231		1.36	2.37
6811		1.36	1.25
8129		1.36	1.01
31424		1.36	1.30
32782		1.36	0.83
7197		1.36	-1.82
	4656	1.36	0.44
3520		1.36	-0.08
	15037	1.36	0.49
4336		1.36	1.40
10593		1.36	0.16
3263		1.36	1.98
13102		1.36	0.59
10134		1.36	-0.06
30425		1.36	0.69
6758		1.36	1.57
5909		1.36	2.86
4784		1.36	0.02
4968		1.35	0.71
15209		1.35	1.47
3527		1.35	-0.32
	18857	1.35	0.98
10060		1.35	-1.40
3019		1.35	1.01
	6618	1.35	-0.21
31127		1.35	-0.04
18091		1.35	1.79
15581		1.35	0.18
13548		1.35	0.29
31974		1.35	0.71
10466		1.35	1.43
9526		1.35	1.08
10039		1.35	0.13
7837		1.35	-1.05
8502		1.35	1.41
4966		1.34	1.19
10587		1.34	0.95
13272		1.34	0.63
9918		1.34	1.03
31210		1.34	1.02
16991		1.34	0.61

15043		1.34	1.42
9354		1.34	0.68
	6102	1.34	0.75
14380		1.34	-0.23
10664		1.34	0.70
1316		1.34	0.60
5577		1.34	1.28
3014		1.34	1.12
15009		1.33	0.16
10444		1.33	-0.10
8044		1.33	-0.90
11788		1.33	0.20
11192		1.33	0.38
5277		1.33	-0.94
31746		1.33	0.03
7626		1.33	1.55
	5638	1.33	0.41
12602		1.33	-0.49
1667		1.33	-0.36
15048		1.33	0.99
4912		1.33	1.41
8087		1.32	0.47
4644		1.32	-0.49
3944		1.32	0.13
5942		1.32	0.55
	3201	1.32	-0.26
10555		1.32	-1.26
14011		1.32	0.56
12511		1.32	-0.04
7053		1.32	1.49
11798		1.32	0.03
7221		1.32	0.45
10590		1.32	0.37
10440		1.32	2.26
9015		1.32	0.11
15793		1.32	0.65
13502		1.32	1.31
2890		1.32	-0.19
15544		1.32	1.09
1098		1.32	0.12
32796		1.32	1.90
32423		1.32	1.39
10977		1.32	-0.19
	762	1.31	0.42
6757		1.31	1.59

1603		1.31	1.45
5411		1.31	0.32
15218		1.31	0.76
9890		1.31	-0.49
2915		1.31	1.78
10317		1.31	0.51
13804		1.31	1.00
3011		1.31	0.09
4788		1.31	-2.75
1922		1.31	0.81
2905		1.31	0.14
7889		1.31	0.75
31106		1.31	0.76
	19094	1.31	0.66
13422		1.31	0.57
31606		1.31	0.54
	4675	1.31	1.03
6744		1.31	-0.22
10595		1.31	1.00
9368		1.31	0.51
5089		1.30	0.72
10948		1.30	0.29
	19527	1.30	-0.15
5482		1.30	0.84
16972		1.30	0.65
1438		1.30	1.10
6759		1.30	1.12
6640		1.30	0.32
13032		1.30	-1.68
31386		1.30	4.18
4187		1.30	1.24
15168		1.30	0.39
10206		1.30	-1.01
12272		1.30	-1.40
	3182	1.30	0.13
4575		1.30	0.73
5923		1.30	0.34
17446		1.30	0.52
	15820	1.30	-0.05
30413		1.30	-0.85
4630		1.30	-0.27
14989		1.30	0.72
8707		1.30	-0.16
31935		1.30	1.15
17274		1.30	-0.09

6906		1.30	-0.59
18662		1.30	0.13
31957		1.29	0.16
32489		1.29	-1.65
31900		1.29	1.10
32195		1.29	-0.09
5627		1.29	1.49
	7666	1.29	1.85
2909		1.29	-2.97
33334		1.29	0.44
14985		1.29	-1.01
30323		1.29	-0.53
4685		1.29	1.49
13550		1.29	-1.95
4818		1.29	0.28
31766		1.29	0.57
9880		1.29	-0.60
30157		1.29	0.43
5045		1.29	0.32
32400		1.29	0.41
1924		1.29	1.07
2048		1.29	-0.72
10688		1.28	-0.53
6900		1.28	0.74
13457		1.28	0.80
5068		1.28	1.26
14390		1.28	-0.17
	11433	1.28	1.34
32416		1.28	
5261		1.28	1.07
14724		1.28	0.41
10543		1.28	-0.68
7537		1.28	1.28
32642		1.28	1.08
12178		1.28	0.57
1629		1.28	0.36
11378		1.28	0.62
1984		1.28	1.08
7958		1.28	0.25
12290		1.28	1.84
3525		1.28	0.20
32415		1.28	-1.03
3744		1.28	0.46
6959		1.28	0.59
4829		1.28	0.73

5278		1.28	0.94
9211		1.28	-0.64
33108		1.28	-0.03
10680		1.28	1.19
31558		1.28	-0.05
8009		1.28	-0.78
	14527	1.28	0.17
3262		1.28	0.91
31304		1.28	0.75
9829		1.27	1.16
	16683	1.27	-0.49
	20190	1.27	-0.91
11208		1.27	1.19
12324		1.27	1.40
31861		1.27	1.25
	19265	1.27	-0.47
18124		1.27	-0.29
18171		1.27	-0.76
6995		1.27	1.12
	6936	1.27	0.11
4000		1.27	1.41
14417		1.27	0.65
12811		1.27	0.78
6860		1.27	1.66
17680		1.27	0.89
	12191	1.27	-0.28
10843		1.26	1.40
31323		1.26	0.16
14448		1.26	-0.28
2811		1.26	0.53
5580		1.26	0.16
5450		1.26	-0.12
7764		1.26	1.21
	7596	1.26	0.40
5514		1.26	-0.15
9396		1.26	0.17
15582		1.26	-0.06
3622		1.26	2.03
6808		1.26	0.61
	15892	1.26	-0.17
31898		1.26	1.02
32308		1.26	1.12
13608		1.26	0.53
5970		1.26	0.37
14117		1.26	0.99

10243		1.26	0.31
4609		1.26	1.48
15198		1.26	1.70
13647		1.26	2.58
8209		1.26	0.15
	7633	1.26	-0.07
14222		1.26	0.74
9522		1.25	2.29
13624		1.25	-0.02
10268		1.25	-0.11
12714		1.25	1.12
9388		1.25	0.35
15134		1.25	0.67
8997		1.25	-1.16
7446		1.25	0.70
10934		1.25	-1.21
11711		1.25	
5972		1.25	1.59
31549		1.25	1.68
13592		1.25	-0.10
6908		1.25	1.12
12794		1.25	1.30
30494		1.25	-0.07
9754		1.25	0.68
7001		1.25	0.65
8639		1.24	0.98
8793		1.24	2.80
1867		1.24	0.49
18466		1.24	1.42
4744		1.24	-1.09
10278		1.24	1.62
5235		1.24	1.45
	15403	1.24	0.14
	10038	1.24	-0.27
5621		1.24	1.11
1532		1.24	1.02
	2460	1.24	2.36
	7659	1.24	0.83
1490		1.24	1.09
10207		1.24	-0.85
13667		1.24	0.41
11266		1.24	1.22
2471		1.24	0.64
15415		1.23	-1.57
3637		1.23	1.44

6164		1.23	-1.31
9547		1.23	0.85
14100		1.23	-1.15
9433		1.23	0.79
5385		1.23	0.56
18553		1.23	0.25
7173		1.23	0.13
9333		1.23	-0.80
5610		1.23	1.47
9384		1.23	0.77
33467		1.23	0.34
13776		1.23	0.28
18368		1.23	0.06
6546		1.23	0.84
12854		1.23	0.60
15373		1.23	0.46
1462		1.23	0.62
8817		1.23	3.01
4589		1.22	1.22
	1575	1.22	0.92
13922		1.22	0.65
31680		1.22	0.90
5189		1.22	-1.52
14165		1.22	
7463		1.22	1.21
30158		1.22	1.18
8281		1.22	-1.33
4428		1.22	0.75
1835		1.22	0.94
15044		1.22	1.30
31188		1.22	-0.79
3238		1.22	1.12
6944		1.22	1.38
11672		1.21	0.47
9248		1.21	-0.05
3064		1.21	-2.02
9193		1.21	0.21
33012		1.21	-1.35
5284		1.21	2.12
	17422	1.21	0.22
	16061	1.21	-0.22
15912		1.21	-0.77
8012		1.21	-0.80
	18959	1.21	0.48
3751		1.21	0.70

32564		1.21	2.29
10605		1.21	2.64
15922		1.21	0.74
31184		1.21	0.56
9108		1.21	-0.23
12699		1.21	0.15
33472		1.21	-0.43
13500		1.21	0.98
5592		1.21	1.15
4184		1.21	1.33
5708		1.21	1.76
9779		1.21	0.76
10620		1.21	0.72
12972		1.21	2.17
13134		1.21	0.65
10808		1.21	0.15
16740		1.21	0.91
13793		1.21	-0.33
4616		1.21	-0.05
10081		1.21	0.72
32112		1.20	-0.66
12835		1.20	0.17
32582		1.20	1.65
	9513	1.20	-0.17
15302		1.20	-0.38
9201		1.20	-0.45
14642		1.20	-0.64
1516		1.20	0.90
	14243	1.20	0.05
6125		1.20	0.68
14332		1.20	1.27
30048		1.20	1.14
6920		1.20	-0.51
30283		1.20	1.12
	5821	1.20	2.12
6098		1.20	0.62
5116		1.20	2.04
4157		1.20	3.19
15629		1.20	1.44
1571		1.20	1.51
9126		1.20	1.09
10104		1.20	0.54
6302		1.20	0.89
10478		1.20	0.97
10842		1.20	0.57

14991		1.19	-1.15
13463		1.19	0.23
11816		1.19	0.38
15655		1.19	-1.58
2168		1.19	3.26
7620		1.19	0.34
2374		1.19	-0.98
2941		1.19	-0.31
17362		1.19	0.44
	20183	1.19	-0.37
17147		1.19	-0.68
10384		1.19	0.54
10539		1.19	-0.48
14383		1.19	-0.60
12820		1.19	-0.55
33480		1.19	-0.30
11629		1.19	0.50
9147		1.19	1.92
8542		1.19	-0.87
9032		1.19	1.30
3979		1.19	-0.03
18539		1.19	1.64
3184		1.19	0.89
	13074	1.19	-1.10
	216	1.18	0.97
30416		1.18	-1.61
4592		1.18	1.09
	4671	1.18	0.40
16959		1.18	0.84
17019		1.18	-0.46
	14735	1.18	-1.45
8540		1.18	-0.69
7288		1.18	0.23
18619		1.18	0.06
32346		1.18	-0.82
32762		1.18	0.77
	8464	1.18	1.30
6763		1.18	0.00
	19206	1.18	-0.53
14495		1.18	-0.84
15333		1.18	0.70
9749		1.18	1.43
32071		1.18	1.04
	20200	1.18	-1.54
15239		1.18	0.69

2103		1.18	1.32
8801		1.18	0.68
11029		1.18	-1.00
9376		1.18	0.85
13814		1.17	-0.74
4710		1.17	1.12
13676		1.17	-0.36
2503		1.17	1.61
	10113	1.17	1.31
17975		1.17	0.79
15445		1.17	-0.03
8531		1.17	0.81
4712		1.17	0.76
8197		1.17	-0.69
5308		1.17	1.52
4466		1.17	1.57
2101		1.17	0.58
9528		1.17	-0.16
6932		1.17	2.16
3016		1.17	1.11
9897		1.17	-1.46
7795		1.17	0.73
1274		1.17	0.85
12092		1.17	-0.11
13949		1.17	1.14
31284		1.17	1.25
31839		1.17	-0.30
9805		1.17	-0.85
10888		1.17	0.24
17734		1.17	-0.02
30337		1.17	1.79
11748		1.17	-0.56
7945		1.17	0.57
5804		1.17	2.12
13089		1.17	-1.45
4838		1.17	0.65
2789		1.17	1.83
11901		1.17	-0.14
18347		1.17	1.75
2081		1.17	0.60
17645		1.17	-0.88
8818		1.17	0.75
32711		1.17	1.72
10217		1.17	0.35
8046		1.17	-1.42

7555		1.16	1.29
2937		1.16	1.77
14994		1.16	-0.13
9266		1.16	0.93
14326		1.16	1.04
11219		1.16	1.22
17723		1.16	1.45
5162		1.16	0.68
14451		1.16	1.06
7164		1.16	0.47
31998		1.16	0.42
10094		1.16	1.21
31360		1.16	-1.01
5741		1.16	1.71
15690		1.16	-1.27
12784		1.16	0.53
15876		1.16	0.89
6559		1.16	1.16
7123		1.16	0.90
7574		1.16	-2.36
6353		1.16	-0.46
32777		1.15	2.35
	5451	1.15	0.61
3183		1.15	-0.28
18069		1.15	-0.58
10917		1.15	0.39
30438		1.15	0.00
	19012	1.15	0.52
8821		1.15	0.41
10984		1.15	-0.42
14969		1.15	-0.22
6145		1.15	0.58
12388		1.15	0.83
14462		1.15	1.31
14932		1.15	0.28
14570		1.15	1.79
6099		1.15	0.24
32751		1.15	-1.00
5412		1.15	-0.39
	11401	1.15	0.47
7228		1.15	1.43
1484		1.15	0.55
33341		1.15	0.67
9739		1.15	0.33
	6142	1.15	0.62

13129		1.15	0.75
	14804	1.15	0.48
14391		1.15	-0.35
1740		1.15	-0.88
31120		1.15	-1.12
4454		1.15	-0.39
11719		1.15	0.31
14111		1.15	0.66
	7660	1.15	1.06
12914		1.14	1.14
15595		1.14	2.24
3633		1.14	1.05
11525		1.14	0.54
7235		1.14	1.44
30377		1.14	-0.38
3811		1.14	0.11
15196		1.14	0.92
33285		1.14	1.12
9453		1.14	0.29
2863		1.14	1.63
18340		1.14	0.94
13935		1.14	1.26
2862		1.14	1.85
30378		1.14	1.03
4057		1.14	-0.20
18420		1.14	0.37
1963		1.14	0.58
11761		1.14	1.16
4450		1.14	-0.01
	1274	1.14	-0.59
14410		1.14	1.13
10159		1.14	0.25
	17317	1.14	0.85
1539		1.14	1.39
31713		1.14	-0.35
4406		1.13	0.11
11837		1.13	0.49
32236		1.13	-0.05
17950		1.13	3.25
11517		1.13	0.85
15396		1.13	-0.86
17218		1.13	1.82
7054		1.13	-0.38
3003		1.13	-0.11
31526		1.13	0.96

7953		1.13	-0.74
31336		1.13	-1.55
8922		1.13	2.23
6760		1.13	0.63
12035		1.13	-0.43
	16800	1.13	-0.26
3774		1.13	1.33
	7100	1.13	1.42
9498		1.13	1.76
33123		1.13	1.11
18247		1.13	1.14
	16915	1.13	0.82
2469		1.13	0.30
	19711	1.13	0.02
8694		1.13	0.20
2918		1.13	1.34
3215		1.13	0.23
	15749	1.13	-1.54
6303		1.13	1.11
4071		1.12	1.38
	7634	1.12	0.24
31116		1.12	0.50
12656		1.12	-1.65
31692		1.12	0.90
6650		1.12	0.80
7210		1.12	0.55
15570		1.12	0.47
3523		1.12	-0.31
	15099	1.12	1.05
7925		1.12	0.51
14810		1.12	0.81
12960		1.12	-1.82
4753		1.12	1.89
	8161	1.12	3.54
31810		1.12	-0.03
18128		1.12	0.01
16974		1.12	-2.10
32536		1.12	0.87
12734		1.12	-1.29
12111		1.12	1.03
4965		1.12	0.57
7055		1.12	-0.52
1385		1.12	-0.35
4983		1.12	-0.68
31806		1.12	-0.93

14628		1.11	0.90
33257		1.11	0.40
6904		1.11	-0.57
13028		1.11	1.19
10231		1.11	1.36
4741		1.11	-0.91
14069		1.11	0.26
12000		1.11	2.08
	16744	1.11	0.18
33005		1.11	1.73
4488		1.11	1.53
6788		1.11	-0.11
5744		1.11	0.46
8167		1.11	-0.39
2100		1.11	0.15
8029		1.11	0.31
12799		1.11	-1.26
6605		1.11	0.92
5098		1.11	1.85
4627		1.11	0.54
32459		1.11	-0.39
6983		1.11	-0.19
8390		1.11	-0.09
17285		1.11	1.40
15427		1.11	0.69
8498		1.10	0.60
33119		1.10	0.29
32259		1.10	0.43
10237		1.10	1.41
	871	1.10	-1.16
5931		1.10	-0.26
3585		1.10	1.47
1257		1.10	-1.81
5134		1.10	1.32
11560		1.10	-0.01
	20201	1.10	-1.68
6284		1.10	0.03
11055		1.10	-1.10
4835		1.10	-1.04
10743		1.10	-0.50
8865		1.10	-0.38
8127		1.10	-1.83
33171		1.10	-0.05
17786		1.10	-1.62
15226		1.10	0.69

30038		1.10	1.46
16985		1.10	0.61
	17320	1.10	0.84
5407		1.10	1.12
1575		1.10	1.60
17063		1.10	0.96
6949		1.10	-0.68
16728		1.10	0.44
6669		1.10	0.22
7962		1.10	1.04
	11866	1.10	1.34
7765		1.10	1.02
30281		1.10	1.20
	13011	1.10	-1.27
14087		1.10	1.26
6409		1.10	0.76
7864		1.10	0.66
11790		1.10	2.15
	19246	1.10	-0.26
13076		1.10	-1.80
10573		1.10	-0.19
31270		1.09	-0.46
32809		1.09	1.49
33062		1.09	1.79
15631		1.09	-0.91
5924		1.09	-0.17
14164		1.09	-0.33
32504		1.09	0.70
15498		1.09	0.09
33306		1.09	-1.25
9932		1.09	1.15
33338		1.09	0.19
18063		1.09	1.65
15157		1.09	1.79
9742		1.09	0.83
5114		1.09	3.04
	16879	1.09	1.38
32631		1.09	-1.23
12295		1.09	0.67
	16682	1.09	-0.43
10861		1.09	0.59
33221		1.09	-2.46
8023		1.09	-2.38
32971		1.08	-0.60
11896		1.08	-1.06

13695		1.08	-0.98
17868		1.08	0.72
3895		1.08	1.35
	17452	1.08	0.12
8025		1.08	-2.43
10348		1.08	0.31
16901		1.08	0.40
32653		1.08	0.76
1749		1.08	1.35
4252		1.08	-0.41
14544		1.08	1.57
6841		1.08	0.15
	18092	1.08	1.76
7768		1.08	0.65
14629		1.08	0.88
5674		1.08	1.07
10334		1.08	0.69
7906		1.08	1.09
12964		1.08	-1.62
7336		1.08	0.05
4576		1.07	-0.58
1082		1.07	0.94
6569		1.07	-1.41
9737		1.07	0.52
16979		1.07	-1.12
12424		1.07	-1.66
5585		1.07	0.03
15211		1.07	2.09
13613		1.07	0.72
6874		1.07	0.92
13837		1.07	0.13
32496		1.07	0.49
33198		1.07	0.84
14643		1.07	1.26
3910		1.07	1.04
1063		1.07	0.66
13407		1.07	1.73
5065		1.06	-0.02
30171		1.06	-0.79
	7680	1.06	1.16
	17253	1.06	1.20
8735		1.06	1.27
14125		1.06	-0.60
6583		1.06	0.83
13976		1.06	-0.85

13900		1.06	0.73
32442		1.06	0.31
8900		1.06	2.51
1507		1.06	-1.27
5313		1.06	1.40
3412		1.06	0.61
12116		1.06	0.83
10920		1.06	-0.35
13603		1.06	-1.25
33126		1.06	0.92
1275		1.06	1.05
13594		1.06	-0.26
	17524	1.06	0.28
	6207	1.06	1.41
3937		1.06	0.72
	10582	1.06	1.01
3360		1.06	0.24
9837		1.06	1.13
13247		1.06	-1.79
9188		1.06	0.66
14341		1.06	-0.77
31164		1.06	1.87
9873		1.06	0.03
15459		1.06	0.20
31133		1.06	0.13
11411		1.06	0.66
5771		1.06	0.29
6455		1.06	0.73
30460		1.06	0.14
10407		1.06	0.97
	11267	1.05	-0.63
9335		1.05	0.80
7343		1.05	1.00
4611		1.05	0.15
7238		1.05	0.14
	20188	1.05	-0.85
11053		1.05	0.55
17068		1.05	1.70
3954		1.05	0.53
13615		1.05	1.07
15096		1.05	-1.70
31012		1.05	0.19
9548		1.05	1.68
16937		1.05	-1.28
	2327	1.05	-0.07

9989		1.05	1.56
13091		1.05	-1.77
31462		1.05	0.55
5772		1.05	0.65
6781		1.05	0.88
11241		1.05	-0.27
8405		1.05	1.64
10898		1.05	0.34
6494		1.04	0.67
	3158	1.04	0.16
5467		1.04	0.50
33009		1.04	0.16
4847		1.04	-0.07
18405		1.04	1.30
9723		1.04	-0.27
31913		1.04	-0.90
15449		1.04	0.87
	8578	1.04	0.86
	2538	1.04	0.39
13207		1.04	1.88
30203		1.04	1.19
6567		1.04	1.04
4633		1.04	0.76
	13009	1.04	-0.84
8536		1.04	0.00
1324		1.04	0.22
31893		1.04	0.35
	17379	1.04	1.05
13699		1.04	-2.02
13597		1.04	-1.48
14511		1.04	0.63
9252		1.04	0.24
	11254	1.04	-0.79
7400		1.04	0.38
4843		1.04	-1.07
	5909	1.04	-0.88
17367		1.04	1.46
18476		1.04	-0.50
12419		1.04	-1.78
17686		1.04	0.93
	17958	1.04	-0.82
12073		1.03	-0.75
32829		1.03	1.54
5142		1.03	0.77
6951		1.03	1.05

32921		1.03	1.11
1258		1.03	-0.93
13030		1.03	-2.02
15561		1.03	1.07
1519		1.03	1.78
9277		1.03	0.41
	17228	1.03	1.77
15104		1.03	0.28
12918		1.03	-0.12
1136		1.03	0.76
32056		1.03	0.49
8578		1.03	0.29
10497		1.03	0.14
5071		1.03	0.61
9564		1.03	-0.17
	14802	1.03	0.74
6649		1.03	0.83
7973		1.03	0.08
32158		1.03	1.02
4813		1.03	0.53
31771		1.03	1.49
32625		1.03	0.91
10364		1.03	0.29
10415		1.03	0.59
12478		1.03	-0.03
	15939	1.03	-1.12
13620		1.03	-1.12
2488		1.03	1.08
10810		1.03	0.04
31757		1.03	0.51
12753		1.03	1.68
12856		1.03	0.01
15122		1.03	-0.52
2614		1.03	2.02
16873		1.03	-0.13
16863		1.03	0.12
6348		1.03	0.89
32712		1.02	0.92
15366		1.02	1.02
32537		1.02	1.29
10970		1.02	0.76
13366		1.02	0.36
30349		1.02	-0.20
31614		1.02	2.12
	18124	1.02	-0.06

7916		1.02	-0.75
17642		1.02	-0.07
6185		1.02	0.77
32105		1.02	1.21
16712		1.02	0.93
30001		1.02	-1.66
7106		1.02	0.46
9214		1.02	0.09
11635		1.02	1.15
10992		1.02	-0.33
15095		1.02	0.69
12586		1.02	-1.07
14122		1.02	0.78
6632		1.02	0.42
	10343	1.02	1.18
8961		1.02	0.34
33494		1.02	-0.66
2091		1.02	-0.27
6958		1.02	0.91
31912		1.02	-1.32
31198		1.02	0.49
8389		1.02	0.01
12816		1.02	0.66
5966		1.02	0.38
30472		1.02	-1.69
5207		1.02	0.81
2144		1.01	1.62
31930		1.01	0.34
1494		1.01	0.94
12730		1.01	0.04
14418		1.01	-0.10
8166		1.01	0.15
17930		1.01	-0.05
32031		1.01	0.95
17924		1.01	0.25
13459		1.01	-0.01
8719		1.01	0.62
	16736	1.01	0.21
9870		1.01	0.91
32346		1.01	0.22
	7601	1.01	-0.94
	7611	1.01	-0.58
5392		1.01	0.03
2857		1.01	0.55
6067		1.01	-1.04

4244		1.01	0.81
	14223	1.01	-0.79
8253		1.01	2.11
7908		1.01	0.30
2917		1.01	2.09
8357		1.01	0.89
17337		1.01	-0.21
	759	1.01	-0.30
4290		1.01	0.37
	20208	1.01	-2.07
18426		1.01	1.64
18144		1.01	0.93
2116		1.00	1.20
6501		1.00	-0.39
	971	1.00	-2.98
5823		1.00	2.67
10242		1.00	0.62
18778		1.00	1.28
31335		1.00	-1.42
14892		1.00	0.73
	10041	1.00	-0.99
32687		1.00	1.37
8436		1.00	2.85
4475		1.00	1.91
	12410	1.00	1.48
18780		1.00	-0.66
31542		1.00	0.97
30352		1.00	2.39
17324		1.00	0.09
30287		1.00	0.96
30466		1.00	-0.32
12024		1.00	0.94
12154		1.00	1.42
32670		1.00	0.77
3772		1.00	0.71
5255		1.00	1.17
2670		1.00	0.61
11242		1.00	0.54
31830		1.00	0.02
5094		1.00	0.96
13379		1.00	1.06
15385		1.00	0.02
	14013	1.00	2.58
	12132	1.00	-1.88
3907		1.00	-0.64

1602		1.00	1.34
4447		1.00	0.18
14441		1.00	0.80
30096		1.00	0.63
32511		1.00	1.03
10353		0.99	-0.01
8560		0.99	-0.56
12203		0.99	0.64
2914		0.99	1.59
12273		0.99	-0.47
4318		0.99	1.13
12120		0.99	1.21
8882		0.99	2.10
14269		0.99	0.09
3466		0.99	2.45
	3756	0.99	-0.59
30105		0.99	-0.64
32085		0.99	-0.20
12594		0.99	0.32
33316		0.99	-1.47
11853		0.99	0.33
14277		0.99	-0.29
14458		0.99	0.00
5629		0.99	0.18
3273		0.99	0.72
5058		0.99	0.25
31132		0.99	1.95
10712		0.99	1.33
9025		0.99	-1.90
18747		0.99	-0.01
	17435	0.99	0.46
7863		0.99	0.09
5939		0.99	0.29
13201		0.99	1.48
	18352	0.99	0.41
5430		0.99	1.17
32856		0.99	1.65
10833		0.99	1.99
32592		0.99	-0.04
2233		0.99	0.10
30411		0.99	0.56
5558		0.98	1.00
10311		0.98	-0.01
33047		0.98	0.67
	13171	0.98	0.59

14973		0.98	-0.55
18471		0.98	0.87
9777		0.98	1.07
5853		0.98	-0.07
5986		0.98	0.08
10631		0.98	0.84
9996		0.98	1.03
5964		0.98	-0.09
30222		0.98	-0.20
1937		0.98	0.96
3662		0.98	1.21
14589		0.98	-1.51
13281		0.98	-0.18
2078		0.98	-1.22
8529		0.98	0.14
31863		0.98	1.96
8725		0.98	-0.07
8606		0.98	0.60
4472		0.98	1.73
11852		0.98	0.37
17271		0.98	-0.86
11298		0.97	-0.01
33166		0.97	0.23
4421		0.97	0.35
8098		0.97	-0.68
30016		0.97	0.01
32696		0.97	1.12
3165		0.97	0.64
	2445	0.97	0.60
7220		0.97	-0.02
6138		0.97	0.67
	7417	0.97	-0.69
	14948	0.97	0.62
12902		0.97	-0.68
12516		0.97	-0.05
31422		0.97	0.97
12077		0.97	-0.55
8414		0.97	0.52
17111		0.97	-1.41
11399		0.97	-0.34
5805		0.97	
9820		0.97	0.05
8552		0.97	0.40
5093		0.97	0.89
12625		0.97	1.45

2839		0.97	1.33
7654		0.97	-1.03
17051		0.97	-0.32
2864		0.97	1.97
4301		0.97	0.78
8596		0.97	-1.67
1089		0.97	0.13
6921		0.97	-0.11
	8342	0.97	-0.89
1690		0.97	0.00
11289		0.97	1.02
6146		0.97	1.04
	18076	0.97	1.43
14481		0.96	0.99
8786		0.96	0.28
6372		0.96	-0.25
1472		0.96	1.09
10901		0.96	0.98
17322		0.96	0.02
18214		0.96	0.05
4649		0.96	-0.01
3795		0.96	1.55
10534		0.96	1.31
9570		0.96	-1.26
16880		0.96	-0.49
6457		0.96	0.12
12557		0.96	1.39
6790		0.96	0.56
32299		0.96	0.99
	17232	0.96	0.73
18729		0.96	0.74
15203		0.96	0.38
13542		0.96	1.07
14654		0.96	1.44
17631		0.96	0.44
14360		0.96	1.04
11750		0.96	0.86
	3363	0.96	0.50
15439		0.96	-1.16
14015		0.96	0.37
31897		0.96	0.66
6456		0.96	0.49
5393		0.96	0.95
5690		0.96	0.46
1344		0.96	0.49

	11603	0.95	-1.23
	19846	0.95	-0.58
12130		0.95	1.12
5946		0.95	2.11
	19398	0.95	-0.10
10660		0.95	-0.31
6259		0.95	0.07
11824		0.95	0.00
1822		0.95	1.32
	16008	0.95	0.70
16792		0.95	-1.67
14535		0.95	1.41
18854		0.95	0.54
7229		0.95	1.20
11491		0.95	0.94
13616		0.95	0.96
8394		0.95	-0.89
	6051	0.95	0.43
	16120	0.95	1.99
	16123	0.95	1.78
9073		0.95	1.28
1271		0.95	0.19
12159		0.95	-0.41
10128		0.95	-0.49
31246		0.94	0.07
4098		0.94	1.22
10625		0.94	0.29
16721		0.94	-0.21
6159		0.94	0.87
13688		0.94	0.32
9769		0.94	3.72
	16556	0.94	-1.36
15133		0.94	-0.10
8788		0.94	-0.67
4443		0.94	0.46
31141		0.94	0.69
10689		0.94	-0.51
3212		0.94	1.23
12292		0.94	0.33
32568		0.94	0.70
	19541	0.94	0.19
	8294	0.94	-0.42
4944		0.94	0.78
8092		0.94	0.79
6607		0.94	0.29

	14023	0.94	0.39
8722		0.94	0.07
5170		0.94	-0.36
5796		0.94	2.41
10588		0.94	-0.02
4254		0.94	0.22
	9367	0.94	-0.64
31305		0.94	0.80
4495		0.94	0.57
3995		0.94	1.24
11940		0.94	1.18
7382		0.94	1.46
1976		0.94	1.22
17758		0.94	1.23
5469		0.94	-1.13
	15815	0.93	-0.31
30480		0.93	0.17
13488		0.93	-0.61
13852		0.93	0.87
	12180	0.93	0.10
32410		0.93	-1.11
15878		0.93	1.12
33492		0.93	-0.61
31148		0.93	-1.38
12133		0.93	0.95
5798		0.93	0.62
1938		0.93	1.32
30332		0.93	1.37
2135		0.93	0.26
6170		0.93	0.30
	7604	0.93	-0.63
	14712	0.93	-1.77
7634		0.93	0.07
13862		0.93	-0.18
14579		0.93	1.12
9917		0.93	0.92
13117		0.93	-0.84
15434		0.93	0.43
9210		0.93	-0.56
13394		0.93	0.67
8208		0.93	-0.85
10679		0.93	-0.01
7254		0.93	0.99
	5146	0.93	1.90
8534		0.93	-0.63

4091		0.93	0.21
	9424	0.93	-0.41
13476		0.92	-0.55
33121		0.92	-0.23
11027		0.92	0.12
	14169	0.92	1.26
10228		0.92	0.11
	11470	0.92	-0.84
31016		0.92	1.13
31005		0.92	0.62
4665		0.92	1.18
3593		0.92	0.98
8273		0.92	0.35
3915		0.92	0.24
8750		0.92	-1.29
32107		0.92	0.46
6333		0.92	0.89
11049		0.92	0.78
8188		0.92	-0.34
14468		0.92	1.16
31265		0.92	0.36
32228		0.92	-1.50
12321		0.92	0.35
7166		0.92	-0.49
13653		0.92	0.68
18537		0.92	0.89
14096		0.92	1.04
6666		0.92	0.00
2345		0.92	1.63
5907		0.92	1.07
	20244	0.92	0.84
	14444	0.92	0.28
31337		0.92	1.13
1514		0.92	0.51
4714		0.92	0.41
3529		0.92	-0.27
13578		0.92	0.45
	4828	0.92	1.79
6779		0.92	1.10
7560		0.92	1.34
31161		0.92	0.76
12608		0.92	-1.21
15779		0.91	1.79
1150		0.91	1.43
5830		0.91	-0.17

6913		0.91	-0.91
7208		0.91	0.62
8021		0.91	1.24
14867		0.91	1.65
13280		0.91	0.72
6126		0.91	0.79
15283		0.91	1.28
1896		0.91	1.00
5596		0.91	0.59
3704		0.91	-0.98
14416		0.91	1.18
4293		0.91	1.38
30019		0.91	0.73
11207		0.91	0.02
3814		0.91	1.13
	11850	0.91	-1.53
3870		0.91	-1.79
2275		0.91	0.18
3411		0.91	0.92
14045		0.91	-1.02
14825		0.91	0.87
3609		0.91	0.33
7729		0.91	1.23
	7157	0.91	0.80
12975		0.91	-0.77
32672		0.91	1.49
4321		0.91	0.79
13856		0.91	0.81
13607		0.90	-1.27
12530		0.90	1.40
9774		0.90	1.11
10913		0.90	0.19
7996		0.90	-0.81
9034		0.90	-0.86
8635		0.90	-1.56
	4818	0.90	0.66
	7600	0.90	-0.66
31317		0.90	0.32
31859		0.90	1.23
1594		0.90	0.05
2286		0.90	0.28
14982		0.90	-0.46
8060		0.90	1.26
13575		0.90	1.13
8760		0.90	1.81

1333		0.90	0.49
12112		0.90	1.96
14357		0.90	0.79
4405		0.90	-0.36
30036		0.90	1.08
6764		0.90	-1.13
17342		0.90	-0.85
18330		0.90	0.94
11407		0.90	-0.75
17359		0.90	0.07
	4681	0.90	0.66
	14234	0.90	1.54
30430		0.90	0.49
10585		0.90	0.41
17143		0.90	1.28
11732		0.90	0.62
4553		0.90	-1.46
32796		0.90	-0.45
12716		0.90	0.86
1399		0.90	0.12
13745		0.90	1.06
14181		0.89	1.00
17654		0.89	0.90
	16436	0.89	0.00
1320		0.89	0.23
5625		0.89	0.99
	14466	0.89	0.30
15010		0.89	1.22
32556		0.89	-0.24
6412		0.89	0.14
14638		0.89	2.09
	7791	0.89	2.37
	6540	0.89	-0.82
9023		0.89	0.24
32521		0.89	1.42
14006		0.89	-0.83
31091		0.89	1.20
1625		0.89	1.55
4634		0.89	1.04
1674		0.89	0.53
	14659	0.89	-0.67
15335		0.89	0.64
14331		0.89	0.71
8043		0.89	1.06
5279		0.89	1.46

17174		0.89	0.54
4266		0.89	0.80
31469		0.89	0.06
7051		0.89	0.27
5648		0.89	0.75
32018		0.89	-0.49
13749		0.89	2.11
4740		0.89	-0.79
15819		0.89	0.39
6495		0.89	0.58
6144		0.88	0.80
6454		0.88	1.12
13213		0.88	1.43
8170		0.88	-0.75
18617		0.88	1.13
33337		0.88	0.63
33230		0.88	0.49
9578		0.88	1.54
	14560	0.88	-1.96
14434		0.88	0.98
14005		0.88	-1.16
1721		0.88	-0.06
31622		0.88	-0.68
12303		0.88	0.49
13337		0.88	0.22
1901		0.88	0.28
11614		0.88	0.87
13792		0.88	-0.99
15590		0.88	0.45
13725		0.88	0.92
1088		0.88	1.63
	4678	0.88	0.29
32584		0.88	-1.20
13766		0.88	0.49
3143		0.88	-0.13
7427		0.88	0.73
31694		0.88	0.20
3957		0.88	0.33
11024		0.88	-0.06
15233		0.88	1.01
17269		0.88	-0.48
4988		0.88	0.81
1599		0.88	1.34
5825		0.87	-0.54
9762		0.87	-0.04

32773		0.87	1.10
5728		0.87	3.02
32234		0.87	1.21
4476		0.87	1.14
7892		0.87	0.19
15735		0.87	0.80
	3373	0.87	0.64
5087		0.87	0.38
	8528	0.87	1.48
9107		0.87	-0.20
4610		0.87	1.36
17026		0.87	-0.46
31289		0.87	-0.18
32240		0.87	0.41
3948		0.87	-0.44
	804	0.87	-0.34
5091		0.87	0.62
9731		0.87	1.88
	14015	0.87	0.41
4079		0.87	0.48
	10342	0.87	1.16
5906		0.87	1.55
6122		0.87	0.97
15807		0.87	-1.46
10241		0.87	1.04
8978		0.87	0.02
17104		0.87	1.16
2669		0.87	0.57
15778		0.87	1.97
3710		0.87	-0.29
9527		0.86	0.23
3635		0.86	0.98
15453		0.86	0.91
14757		0.86	0.57
3542		0.86	0.11
5381		0.86	0.98
17625		0.86	0.20
4485		0.86	0.22
	10482	0.86	0.76
12268		0.86	-1.04
8016		0.86	0.32
	5380	0.86	1.09
32637		0.86	1.32
14062		0.86	1.26
	13095	0.86	-0.59

3321		0.86	0.31
8392		0.86	-0.12
17461		0.86	-0.14
4404		0.86	2.12
5399		0.86	1.27
30427		0.86	0.83
1492		0.86	0.96
8321		0.86	-0.58
1394		0.86	0.25
32088		0.86	-2.04
7342		0.86	-0.13
7211		0.86	1.44
3045		0.86	1.39
9355		0.86	0.10
31389		0.86	0.87
6133		0.86	-0.64
4770		0.86	1.37
3653		0.85	0.50
32441		0.85	-0.98
31104		0.85	0.74
	5937	0.85	0.35
13860		0.85	0.17
	18149	0.85	1.95
1764		0.85	1.03
6746		0.85	-0.29
17689		0.85	1.30
14363		0.85	1.64
7202		0.85	0.40
	10628	0.85	-0.87
	11616	0.85	0.21
	10046	0.85	-1.40
6181		0.85	1.00
12233		0.85	0.75
9447		0.85	-0.28
1956		0.85	0.83
5765		0.85	0.53
13263		0.85	0.48
14569		0.85	0.58
12637		0.85	0.51
12664		0.85	1.18
32487		0.85	0.45
7888		0.85	0.77
7499		0.85	-0.01
7542		0.85	0.21
	3136	0.85	-0.08

8232		0.85	-0.06
4151		0.85	0.60
	3719	0.85	1.53
16988		0.85	-1.01
8261		0.85	-0.48
3345		0.85	-0.13
9802		0.85	0.57
10195		0.84	0.28
10013		0.84	-0.61
6142		0.84	0.83
32662		0.84	0.65
2604		0.84	0.37
4916		0.84	0.00
15769		0.84	0.45
4792		0.84	0.39
8561		0.84	-0.60
1028		0.84	0.51
5776		0.84	0.71
11335		0.84	0.21
11286		0.84	-0.28
14749		0.84	0.27
	1517	0.84	1.17
10117		0.84	1.25
31311		0.84	-0.14
6327		0.84	0.09
18649		0.84	0.63
9579		0.84	2.88
7148		0.84	0.63
8274		0.84	-0.02
2991		0.84	1.12
5001		0.84	0.23
1658		0.84	0.39
15710		0.84	0.76
7818		0.84	0.34
16983		0.84	-1.28
2671		0.84	1.20
32277		0.84	0.60
4005		0.84	0.73
1913		0.84	-1.42
	13247	0.84	1.74
4070		0.84	0.88
5185		0.84	-2.12
13350		0.84	0.69
18783		0.84	1.66
13663		0.83	0.68

10484		0.83	1.14
13125		0.83	-1.18
7443		0.83	-0.58
13674		0.83	-1.30
5873		0.83	-0.95
4619		0.83	1.32
8134		0.83	0.81
13874		0.83	-0.86
17034		0.83	0.05
7225		0.83	1.54
2986		0.83	0.43
30387		0.83	0.24
3338		0.83	0.02
4752		0.83	1.03
14718		0.83	-0.43
	12196	0.83	0.02
	10779	0.83	-0.94
3793		0.83	0.58
10092		0.83	1.16
1957		0.83	1.27
3032		0.83	1.12
3558		0.83	0.56
	11460	0.83	-0.34
18096		0.83	0.18
2919		0.83	0.61
6767		0.83	0.29
7883		0.83	0.76
8908		0.83	-1.20
	6109	0.83	0.59
	8285	0.83	-1.02
31884		0.83	-1.25
6277		0.83	1.09
11122		0.83	0.99
11451		0.83	1.83
16723		0.83	-0.21
9020		0.83	0.16
	4684	0.83	1.08
4218		0.83	1.32
3096		0.82	-0.87
11182		0.82	0.71
30427		0.82	-0.46
3889		0.82	0.81
31557		0.82	0.00
31999		0.82	0.43
30463		0.82	1.31

3769		0.82	0.46
	16557	0.82	-1.17
14237		0.82	1.34
33002		0.82	-0.20
15323		0.82	-1.60
6431		0.82	0.79
12837		0.82	0.76
16976		0.82	-1.14
6069		0.82	0.76
17744		0.82	1.29
11267		0.82	0.58
	7602	0.82	0.49
33310		0.82	0.51
4204		0.82	0.24
12665		0.82	1.04
13096		0.82	-2.05
32196		0.82	0.18
13494		0.82	-0.24
2530		0.82	-0.12
7088		0.82	1.20
30437		0.82	1.13
31120		0.82	-0.97
	10341	0.82	1.44
1543		0.82	0.98
14792		0.82	0.65
32985		0.82	0.26
	18952	0.82	1.27
5899		0.82	0.08
	3379	0.82	0.16
11726		0.82	0.58
	2265	0.82	-1.34
11373		0.82	0.02
9945		0.82	1.15
1531		0.82	0.38
	10221	0.82	-0.10
7770		0.82	0.54
14059		0.82	0.59
14104		0.81	0.91
17687		0.81	1.33
9022		0.81	-0.09
18316		0.81	1.28
14950		0.81	1.19
15345		0.81	0.78
32743		0.81	0.39
10112		0.81	1.08

4082		0.81	0.23
8774		0.81	-1.70
32648		0.81	1.47
32663		0.81	0.01
14031		0.81	1.68
32702		0.81	0.58
8591		0.81	0.57
1800		0.81	-0.26
12607		0.81	1.00
	11370	0.81	0.73
31674		0.81	0.20
32834		0.81	-1.46
	13477	0.81	1.12
7194		0.81	0.13
5272		0.81	0.00
6163		0.81	1.31
3320		0.81	-0.02
7605		0.81	0.67
	954	0.81	1.20
1121		0.81	0.71
7926		0.81	0.74
	10048	0.81	-1.58
6749		0.81	-0.63
18398		0.81	1.15
8233		0.81	0.54
31976		0.81	0.08
14563		0.81	0.73
31406		0.81	2.92
32425		0.81	1.36
17982		0.81	0.18
18473		0.81	1.00
31068		0.81	0.18
10262		0.81	1.31
2934		0.80	-1.21
9778		0.80	-0.31
32210		0.80	1.08
3083		0.80	0.78
32072		0.80	0.94
7948		0.80	1.01
31732		0.80	0.94
8388		0.80	0.21
3780		0.80	0.57
18577		0.80	0.62
31731		0.80	0.53
	11444	0.80	1.29

9541		0.80	0.38
6476		0.80	0.44
2835		0.80	1.73
5106		0.80	0.26
9027		0.80	0.73
1443		0.80	0.32
1225		0.80	0.12
9856		0.80	-0.91
13713		0.80	0.64
10413		0.80	0.18
12507		0.80	0.54
8854		0.80	1.82
12176		0.80	0.74
5693		0.80	0.86
	19406	0.80	0.20
7643		0.80	0.11
15835		0.80	1.19
32025		0.80	1.30
7058		0.80	0.60
14183		0.80	0.29
31786		0.80	1.05
	13159	0.80	-0.63
3767		0.80	0.82
2098		0.80	-0.14
31662		0.80	-1.40
2453		0.80	-0.29
14884		0.80	0.56
6461		0.80	0.00
9445		0.80	1.18
10433		0.80	-0.08
15098		0.80	1.10
1559		0.80	0.18
6912		0.80	0.85
31770		0.79	1.16
8036		0.79	1.01
5727		0.79	-2.62
	7167	0.79	0.29
14827		0.79	0.80
7332		0.79	-1.65
7580		0.79	0.75
8874		0.79	0.19
4805		0.79	0.44
12347		0.79	0.66
9423		0.79	1.19
18469		0.79	0.05

15462		0.79	-0.23
	20320	0.79	1.09
32115		0.79	0.83
4969		0.79	1.11
6730		0.79	-0.21
5102		0.79	0.02
31901		0.79	0.87
7752		0.79	0.60
17098		0.79	1.26
	10511	0.79	1.15
15605		0.79	0.27
3922		0.79	1.12
18412		0.79	0.10
	7605	0.79	-0.13
31001		0.79	0.18
17181		0.79	0.11
2964		0.79	0.54
4816		0.79	0.64
1971		0.79	-0.36
	1289	0.79	-0.05
9316		0.79	-1.07
4328		0.79	-0.24
7073		0.79	1.81
	17241	0.79	0.63
	19532	0.79	2.08
33251		0.79	2.21
15118		0.79	0.38
10493		0.79	0.75
	12157	0.79	-2.50
13445		0.79	-0.20
5252		0.79	0.41
17778		0.79	-0.68
5455		0.79	0.60
16975		0.79	-1.79
	6881	0.79	0.02
14637		0.79	-0.90
3327		0.78	-0.09
1318		0.78	0.56
4960		0.78	0.61
9377		0.78	0.88
8823		0.78	-0.38
	18073	0.78	0.92
	14538	0.78	-3.43
18600		0.78	-0.19
17945		0.78	-1.06

14680		0.78	0.77
10230		0.78	0.72
4827		0.78	0.17
6127		0.78	0.18
	7564	0.78	0.05
	7102	0.78	0.41
6627		0.78	0.10
13124		0.78	-0.70
5410		0.78	0.66
10229		0.78	0.51
2816		0.78	0.40
15730		0.78	0.45
33483		0.78	0.83
1104		0.78	0.06
	12128	0.78	-1.35
13941		0.78	1.21
10531		0.78	-0.13
3048		0.78	1.15
13029		0.78	0.64
5661		0.78	0.72
4558		0.78	0.36
12521		0.78	0.24
32171		0.78	0.09
	6893	0.78	-1.80
7218		0.78	0.32
2346		0.77	0.85
30115		0.77	0.31
32186		0.77	1.68
7188		0.77	-0.44
9920		0.77	0.41
18636		0.77	-0.30
4723		0.77	-0.01
31063		0.77	0.30
	9567	0.77	1.25
9997		0.77	0.36
	18075	0.77	-0.19
6745		0.77	-0.09
7172		0.77	-0.52
5199		0.77	1.46
4066		0.77	0.27
10699		0.77	0.56
13110		0.77	0.47
	8965	0.77	-0.18
10763		0.77	-0.56
13630		0.77	-1.45

6429		0.77	0.95
7067		0.77	1.33
2257		0.77	0.07
	17117	0.77	0.92
3917		0.77	0.72
30375		0.77	0.45
	7244	0.77	1.30
	2734	0.77	-0.16
6852		0.77	0.06
33144		0.77	0.68
15902		0.77	0.80
14499		0.77	0.17
13559		0.77	-0.80
31744		0.77	0.52
30085		0.77	2.23
1743		0.77	0.07
15083		0.77	0.08
	12906	0.77	0.97
12732		0.77	1.06
4078		0.77	0.52
8118		0.77	1.09
9391		0.77	-0.29
1763		0.76	0.78
8441		0.76	0.85
4917		0.76	0.31
9733		0.76	-0.08
14744		0.76	0.27
2022		0.76	0.44
8860		0.76	-0.37
	16023	0.76	1.44
31066		0.76	0.38
5092		0.76	1.46
14024		0.76	0.53
6838		0.76	-0.37
8238		0.76	1.28
30476		0.76	-0.24
18559		0.76	0.68
4163		0.76	0.69
12952		0.76	1.15
1338		0.76	0.54
10498		0.76	0.01
3590		0.76	2.08
2186		0.76	0.58
9728		0.76	0.59
11143		0.76	0.88

12486		0.76	0.33
10804		0.76	0.71
30153		0.76	1.30
3162		0.76	0.36
	13476	0.76	0.55
13841		0.76	-0.12
	18297	0.76	1.17
31718		0.76	0.76
30499		0.76	0.26
13279		0.76	-0.90
30259		0.76	1.54
11324		0.76	1.23
5446		0.76	0.57
	13464	0.76	-0.34
10226		0.76	0.55
	912	0.76	0.39
10133		0.76	0.22
	14600	0.76	0.43
9813		0.76	1.23
	6110	0.75	-0.14
5397		0.75	0.04
	7554	0.75	-1.07
12177		0.75	0.49
	14857	0.75	-0.94
	7565	0.75	-0.57
14640		0.75	1.77
17819		0.75	0.65
4579		0.75	-0.40
1567		0.75	0.94
33264		0.75	0.37
	14682	0.75	-0.67
10604		0.75	1.27
12725		0.75	0.70
32057		0.75	-0.12
	4774	0.75	1.03
13732		0.75	0.12
11294		0.75	3.75
33197		0.75	0.29
12090		0.75	-0.31
14936		0.75	0.53
15395		0.75	0.06
14971		0.75	-1.13
10214		0.75	-0.13
9638		0.75	0.56
3887		0.75	0.98

30043		0.75	-0.57
3886		0.75	0.52
1210		0.75	0.03
	19535	0.75	2.15
31080		0.75	1.56
6379		0.75	-1.48
	17874	0.75	-0.60
12234		0.75	0.43
1311		0.75	0.91
18410		0.75	1.00
32155		0.75	1.09
11664		0.75	-0.06
7454		0.75	-1.62
4672		0.75	1.51
3960		0.75	2.23
31241		0.75	-0.28
14098		0.75	0.58
14472		0.75	1.88
32781		0.75	0.97
32135		0.75	-0.72
	13964	0.75	-1.27
12209		0.74	1.91
2110		0.74	-0.22
15616		0.74	-1.30
18259		0.74	0.40
14646		0.74	-0.60
33095		0.74	0.38
16892		0.74	0.29
4899		0.74	0.23
31773		0.74	0.65
15309		0.74	0.39
	14523	0.74	1.17
3956		0.74	0.54
10640		0.74	0.72
15412		0.74	0.98
4927		0.74	0.74
2174		0.74	1.22
8841		0.74	-0.57
6976		0.74	0.81
9552		0.74	0.21
17894		0.74	0.76
30385		0.74	-0.37
6337		0.74	-0.60
1970		0.74	0.05
2330		0.74	0.01

17999		0.74	0.51
	15431	0.74	0.78
17118		0.74	0.86
2217		0.74	-1.92
	7654	0.74	0.51
1962		0.74	1.64
9637		0.74	0.19
7291		0.74	0.41
10527		0.74	0.39
10246		0.74	0.12
	8267	0.74	0.78
	11811	0.74	0.26
5285		0.74	1.34
6682		0.74	0.23
18767		0.73	-0.53
2813		0.73	1.03
	13141	0.73	-0.97
5220		0.73	-0.31
	217	0.73	0.63
4317		0.73	0.73
14500		0.73	-0.82
10352		0.73	-0.06
11775		0.73	-0.41
	15595	0.73	-1.26
	15095	0.73	0.55
	14780	0.73	-1.72
	16691	0.73	-0.50
3217		0.73	0.13
32863		0.73	-1.77
1898		0.73	0.82
32843		0.73	0.53
2162		0.73	1.93
17304		0.73	0.83
15014		0.73	1.89
13384		0.73	-1.79
32719		0.73	0.71
31040		0.73	0.74
6292		0.73	2.87
11742		0.73	-1.21
14459		0.73	-0.97
9569		0.73	-1.63
31296		0.73	1.48
	18344	0.73	0.13
4820		0.73	0.32
	20257	0.73	-0.11

31257		0.73	0.12
3940		0.73	2.23
14420		0.73	1.41
32108		0.73	-0.49
	18571	0.73	0.85
17213		0.73	0.57
15060		0.73	-0.39
7597		0.73	-0.68
6217		0.73	-0.67
33145		0.72	-0.89
	72	0.72	-1.79
9911		0.72	0.43
13680		0.72	-1.11
	11656	0.72	-0.04
30484		0.72	0.35
7066		0.72	-0.63
9463		0.72	1.02
7383		0.72	0.39
1517		0.72	0.86
6046		0.72	0.19
16725		0.72	1.84
13886		0.72	1.01
	15807	0.72	-1.35
14105		0.72	-0.74
5498		0.72	-0.32
15393		0.72	0.61
7747		0.72	0.39
10192		0.72	0.08
33057		0.72	0.25
4299		0.72	0.61
8210		0.72	-0.04
1341		0.72	1.23
5175		0.72	1.37
17250		0.72	0.27
9902		0.72	0.84
16753		0.72	0.77
18317		0.72	-1.04
30129		0.72	0.08
14480		0.72	1.12
9389		0.72	0.79
30404		0.72	-1.12
12251		0.72	0.16
12684		0.72	1.31
7693		0.72	-0.33
2528		0.72	0.34

13972		0.72	-0.27
8873		0.72	0.82
31535		0.72	1.07
5216		0.72	1.00
6103		0.72	-0.14
11849		0.72	0.98
	16730	0.72	-1.00
17246		0.72	-1.15
	15810	0.72	-0.52
3950		0.72	-0.16
13602		0.71	0.59
8863		0.71	0.48
4852		0.71	0.58
14701		0.71	0.37
13984		0.71	1.39
8579		0.71	-0.18
	6363	0.71	1.46
1530		0.71	0.55
10753		0.71	0.92
10805		0.71	0.25
11656		0.71	-0.03
13869		0.71	0.82
30092		0.71	0.97
3975		0.71	0.38
9220		0.71	-1.31
12535		0.71	1.17
7276		0.71	1.03
14168		0.71	1.64
	10264	0.71	-0.51
6147		0.71	0.57
7323		0.71	1.11
7672		0.71	-0.12
	2275	0.71	-0.67
	2478	0.71	-0.48
3530		0.71	0.05
14616		0.71	-1.05
17287		0.71	-0.61
5355		0.71	0.34
33217		0.71	-1.17
4140		0.71	0.83
15883		0.71	1.19
	14429	0.71	-0.03
7297		0.71	0.32
11679		0.71	-0.23
15930		0.71	0.22

17292		0.71	0.50
17012		0.71	-0.73
	7418	0.71	-1.12
13556		0.71	-0.90
11246		0.71	0.46
12439		0.71	0.83
11755		0.71	1.30
11864		0.71	0.50
	20160	0.71	-0.91
13438		0.71	0.39
	12428	0.71	-0.47
33214		0.70	-0.34
3747		0.70	0.98
8996		0.70	0.39
13140		0.70	0.83
15267		0.70	0.76
6630		0.70	0.17
12283		0.70	-0.68
5651		0.70	-0.83
	11547	0.70	1.64
18510		0.70	-0.21
6897		0.70	1.90
11301		0.70	0.72
13948		0.70	0.54
11258		0.70	0.82
11865		0.70	0.60
7999		0.70	-1.27
8121		0.70	0.40
	943	0.70	1.06
	14918	0.70	1.50
13552		0.70	0.10
14871		0.70	1.79
14471		0.70	0.22
11692		0.70	0.92
30288		0.70	0.61
13458		0.70	0.69
6876		0.70	0.16
1895		0.70	-0.80
5921		0.70	0.03
8975		0.70	0.02
31536		0.70	1.62
7439		0.70	0.12
8005		0.70	-0.13
4946		0.70	0.12
17272		0.70	-0.71

30141		0.70	0.63
7441		0.70	0.36
4626		0.70	1.23
32750		0.70	0.11
11414		0.70	0.66
12640		0.70	-0.32
	12287	0.70	0.61
14817		0.70	0.93
10371		0.70	-1.30
13511		0.70	0.71
14889		0.70	-0.46
32104		0.70	-0.84
9187		0.70	-0.34
32797		0.70	-0.91
14439		0.69	1.01
13801		0.69	0.37
33485		0.69	2.90
18135		0.69	0.41
14248		0.69	1.16
10139		0.69	0.12
30487		0.69	0.90
15770		0.69	-0.04
10301		0.69	0.14
11379		0.69	0.07
13011		0.69	1.27
8628		0.69	-1.04
17024		0.69	-1.16
	18724	0.69	1.20
4497		0.69	0.57
15818		0.69	1.05
	5896	0.69	-1.25
	14364	0.69	1.11
1113		0.69	-0.10
	6835	0.69	-1.00
	17466	0.69	-1.29
17280		0.69	0.14
	14863	0.69	-0.38
8620		0.69	-1.12
14408		0.69	1.59
12789		0.69	0.22
	18157	0.69	0.93
8291		0.69	0.46
5940		0.69	-0.60
	7563	0.69	0.32
	9600	0.69	1.27

	14580	0.69	-0.98
6463		0.69	-0.46
	17210	0.69	0.87
13340		0.69	1.39
1515		0.69	0.97
	11404	0.69	0.68
6370		0.69	0.08
13495		0.69	-0.77
5085		0.69	-0.11
11715		0.69	0.62
11320		0.69	0.36
4928		0.69	0.31
15717		0.69	0.58
	17301	0.69	1.66
1049		0.69	-1.01
10838		0.69	0.21
2380		0.69	0.38
4955		0.69	-0.65
11839		0.68	0.73
30101		0.68	1.02
6536		0.68	0.29
4670		0.68	0.99
7887		0.68	0.70
18622		0.68	-0.66
5841		0.68	-0.16
15499		0.68	-0.20
1378		0.68	0.03
3911		0.68	0.48
5936		0.68	-0.32
4074		0.68	0.94
	8769	0.68	0.85
13396		0.68	0.54
7099		0.68	-0.46
15293		0.68	0.36
15753		0.68	0.99
17622		0.68	0.71
6736		0.68	-0.22
18449		0.68	-1.57
12236		0.68	1.38
3552		0.68	1.11
5010		0.68	0.59
9726		0.68	0.22
	17369	0.68	-0.23
6283		0.68	0.20
11604		0.68	-0.58

32732		0.68	1.32
4221		0.68	-1.42
13216		0.68	1.65
13116		0.68	-1.93
11767		0.68	0.80
15191		0.68	-1.29
33460		0.68	-0.64
33070		0.68	1.26
12441		0.68	0.27
2826		0.68	0.74
2114		0.68	0.24
18369		0.68	0.80
33100		0.68	
7490		0.68	0.60
14219		0.68	
10602		0.68	0.60
	8759	0.67	1.85
6686		0.67	-0.38
	15952	0.67	0.27
4774		0.67	1.49
17974		0.67	-0.01
17404		0.67	1.62
	7431	0.67	1.28
	3159	0.67	0.24
31251		0.67	0.33
3959		0.67	1.29
7787		0.67	0.36
11752		0.67	0.07
15440		0.67	0.00
14340		0.67	1.05
31709		0.67	-2.68
4897		0.67	1.05
11319		0.67	0.08
30026		0.67	-0.99
11146		0.67	0.29
31941		0.67	-0.43
14082		0.67	0.84
11676		0.67	0.91
32300		0.67	0.03
12655		0.67	-1.45
10018		0.67	0.62
11282		0.67	-0.02
30099		0.67	0.34
6980		0.67	-0.23
	10927	0.67	-1.12

	17526	0.67	1.26
32919		0.67	0.90
11106		0.67	0.64
11753		0.67	1.70
18643		0.67	-0.20
18156		0.67	-0.83
32263		0.67	0.68
	14592	0.67	0.35
2995		0.67	0.84
15399		0.67	0.63
	320	0.67	-0.99
7910		0.67	1.12
10347		0.67	0.34
33262		0.67	0.16
	10037	0.67	-0.77
	1179	0.67	1.90
15370		0.67	1.13
5638		0.67	-0.79
8417		0.67	0.87
7432		0.67	0.06
15924		0.67	1.00
10326		0.66	-0.21
3949		0.66	-0.41
15845		0.66	1.23
12726		0.66	1.00
7919		0.66	0.81
8203		0.66	-0.50
14537		0.66	0.07
3576		0.66	-1.82
8262		0.66	0.20
11274		0.66	1.27
5973		0.66	0.11
32385		0.66	0.63
	8507	0.66	-0.17
13239		0.66	-0.28
9045		0.66	0.84
1873		0.66	-1.35
12736		0.66	0.49
30169		0.66	0.26
8204		0.66	0.48
	2704	0.66	1.17
32388		0.66	1.18
3715		0.66	-0.49
	6009	0.66	0.73
10510		0.66	0.13

3440		0.66	1.08
2005		0.66	1.45
33332		0.66	0.29
1009		0.66	1.15
6352		0.66	-0.33
9256		0.66	-0.50
12026		0.66	0.64
	8434	0.66	0.89
8141		0.66	-1.01
	2776	0.66	-0.77
2016		0.66	0.13
1529		0.66	-0.30
7007		0.66	0.19
3413		0.66	0.98
17673		0.66	1.05
32540		0.66	0.00
12102		0.66	0.50
7364		0.66	-0.37
32886		0.66	0.48
12010		0.66	0.45
1542		0.66	1.39
4122		0.66	1.35
8008		0.66	-0.87
1440		0.66	0.46
9740		0.66	1.51
1618		0.66	-0.15
32172		0.66	-0.79
31006		0.66	1.02
1951		0.66	0.01
10393		0.66	-0.06
33293		0.66	-0.28
7169		0.66	-0.22
	7172	0.66	-1.13
6186		0.66	1.19
14359		0.65	-0.68
7350		0.65	-0.33
	17741	0.65	-0.31
17025		0.65	1.31
14850		0.65	0.11
	16067	0.65	0.35
	16780	0.65	-0.16
8881		0.65	-0.65
9783		0.65	0.04
1495		0.65	1.47
31222		0.65	0.43

7607		0.65	0.59
1304		0.65	0.53
12269		0.65	0.03
6435		0.65	0.40
9473		0.65	0.35
17556		0.65	0.98
30027		0.65	1.12
4910		0.65	-0.84
4934		0.65	-0.01
	17652	0.65	0.93
12110		0.65	1.12
9125		0.65	-0.51
17302		0.65	0.10
8364		0.65	-0.66
8603		0.65	2.02
15523		0.65	0.87
5333		0.65	0.38
13214		0.65	1.48
	14237	0.65	1.09
2956		0.65	-0.10
6603		0.65	0.16
7893		0.65	0.07
5241		0.65	0.88
11251		0.65	0.38
	19650	0.65	1.40
8358		0.65	0.66
5118		0.65	-0.78
8979		0.65	0.11
14450		0.65	0.47
18109		0.65	0.02
31156		0.65	0.70
30177		0.65	0.61
8673		0.65	0.29
5682		0.65	0.09
9170		0.65	-0.07
5394		0.65	0.17
30415		0.65	-0.44
2902		0.65	1.97
12217		0.65	-0.84
10076		0.65	-0.18
6444		0.65	-0.31
17623		0.64	0.25
12624		0.64	0.49
13397		0.64	0.40
18174		0.64	0.77

6271		0.64	1.74
	3388	0.64	-1.37
4271		0.64	1.15
9969		0.64	0.30
7092		0.64	0.66
10219		0.64	-0.44
9359		0.64	-1.03
8701		0.64	0.58
4211		0.64	0.35
14886		0.64	1.19
8830		0.64	0.71
31293		0.64	0.44
	7065	0.64	-1.22
32091		0.64	-0.81
12763		0.64	0.46
3280		0.64	1.10
14550		0.64	0.57
12220		0.64	0.30
7933		0.64	0.34
3027		0.64	1.51
5386		0.64	0.10
1076		0.64	0.39
8086		0.64	-0.16
14743		0.64	1.14
5262		0.64	1.66
13724		0.64	0.51
10868		0.64	2.02
8171		0.64	4.54
17816		0.64	0.94
11043		0.64	-0.41
30482		0.64	0.88
7219		0.64	-0.12
5497		0.64	0.18
1678		0.64	0.46
4194		0.64	-0.63
31295		0.64	-0.09
11334		0.64	-0.12
	2542	0.64	0.65
33220		0.64	-0.78
18631		0.64	1.72
9550		0.64	-1.58
13563		0.64	1.24
	8075	0.63	-0.25
	13381	0.63	0.62
5619		0.63	0.65

1597		0.63	0.64
14299		0.63	1.24
9648		0.63	-0.24
14214		0.63	
30058		0.63	-0.08
14690		0.63	0.07
14669		0.63	0.12
14728		0.63	0.02
4467		0.63	0.17
31925		0.63	0.53
31826		0.63	0.71
5388		0.63	0.41
33104		0.63	2.07
3672		0.63	1.00
13188		0.63	0.34
12045		0.63	1.82
13742		0.63	0.31
5557		0.63	0.42
8964		0.63	-0.44
	6247	0.63	0.50
17258		0.63	-0.26
7077		0.63	-0.08
1108		0.63	0.37
1722		0.63	0.69
14265		0.63	0.37
10369		0.63	-0.93
4980		0.63	-0.02
	19678	0.63	1.35
14330		0.63	0.97
5911		0.63	-0.22
	14087	0.63	-0.15
14254		0.63	1.57
4433		0.63	-0.58
12628		0.63	0.69
9312		0.63	0.92
32434		0.63	0.84
3639		0.63	0.84
11140		0.63	0.18
8562		0.63	-1.67
4128		0.62	0.73
7997		0.62	-1.54
1888		0.62	-0.13
32695		0.62	0.07
31605		0.62	0.75
13010		0.62	0.64

4924		0.62	1.02
15489		0.62	1.04
18193		0.62	-0.06
6335		0.62	-0.40
32509		0.62	0.86
16918		0.62	0.87
4141		0.62	0.80
4322		0.62	0.38
32578		0.62	0.65
15824		0.62	0.58
18363		0.62	2.37
2916		0.62	-0.33
1371		0.62	0.47
	2476	0.62	-0.90
2608		0.62	1.05
5148		0.62	1.24
33227		0.62	0.14
18125		0.62	0.52
6304		0.62	-1.79
31708		0.62	0.00
32453		0.62	0.04
1107		0.62	0.67
13796		0.62	0.53
	14973	0.62	1.09
9768		0.62	0.25
10286		0.62	0.05
32651		0.62	0.62
13077		0.62	1.62
1972		0.62	0.48
14536		0.62	1.10
5559		0.62	-0.13
6588		0.62	0.13
12892		0.62	0.86
31132		0.62	1.13
3931		0.62	-0.35
11347		0.61	0.11
15278		0.61	0.05
10031		0.61	0.56
13240		0.61	-0.13
1783		0.61	0.40
6903		0.61	-0.43
33090		0.61	-0.27
33154		0.61	0.05
33350		0.61	0.54
14508		0.61	0.41

10739		0.61	0.13
3539		0.61	0.58
	7557	0.61	-0.39
6634		0.61	-1.23
13775		0.61	-1.24
1331		0.61	1.60
31414		0.61	0.09
3996		0.61	-0.25
6856		0.61	0.43
14688		0.61	0.64
8967		0.61	-1.30
15712		0.61	0.56
32530		0.61	0.96
13970		0.61	0.79
7126		0.61	0.85
5640		0.61	-0.22
31659		0.61	0.79
5115		0.61	1.43
	19536	0.61	1.27
	17373	0.61	-0.12
4739		0.61	0.09
1829		0.61	0.46
3916		0.61	0.80
9232		0.61	0.00
13101		0.61	-0.49
1502		0.61	0.35
6813		0.61	1.02
1631		0.61	-0.05
31110		0.61	-0.06
1826		0.61	-0.38
17049		0.61	-0.78
7960		0.61	0.14
13958		0.61	-0.82
3363		0.61	-2.00
15429		0.60	0.35
7576		0.60	1.05
17259		0.60	0.94
31345		0.60	0.96
3394		0.60	0.48
	13072	0.60	-2.32
	12386	0.60	1.38
1307		0.60	0.09
12817		0.60	-0.33
31742		0.60	-3.76
15532		0.60	2.02

	2527	0.60	-2.48
5121		0.60	0.63
11888		0.60	1.00
3581		0.60	-1.90
7943		0.60	0.24
7192		0.60	0.68
18090		0.60	0.26
15170		0.60	0.75
14645		0.60	0.94
17177		0.60	1.12
10594		0.60	-0.34
	16114	0.60	1.41
16779		0.60	-0.59
	3721	0.60	0.88
6585		0.60	1.04
8770		0.60	0.54
4035		0.60	-1.10
	6252	0.60	1.70
13189		0.60	0.40
2006		0.60	1.34
	7637	0.60	0.37
32167		0.60	0.67
6586		0.60	-0.92
3820		0.60	0.90
5611		0.60	0.91
6625		0.60	1.29
12038		0.60	-0.08
2540		0.60	1.56
1578		0.60	1.23
31149		0.60	0.64
18635		0.60	0.02
33298		0.60	0.77
	7843	0.60	1.29
6611		0.60	-1.61
7938		0.60	0.98
	12285	0.60	1.76
33302		0.60	0.01
17935		0.60	-1.57
9537		0.60	-0.22
13148		0.60	0.80
32774		0.60	0.12
1227		0.60	-0.17
	19222	0.60	-1.79
7390		0.60	0.29
	13008	0.60	-0.65

17141		0.59	0.61
6756		0.59	0.17
6072		0.59	0.31
15159		0.59	0.92
31308		0.59	0.89
7052		0.59	-0.03
8728		0.59	-0.62
16952		0.59	0.95
32592		0.59	-0.24
15400		0.59	-1.38
32573		0.59	0.30
6734		0.59	-1.11
32523		0.59	0.45
13255		0.59	0.45
	6801	0.59	-1.21
7222		0.59	-0.50
6652		0.59	0.60
1598		0.59	1.28
	11622	0.59	0.33
6188		0.59	-0.22
10915		0.59	-0.21
6496		0.59	0.94
16726		0.59	-0.38
1555		0.59	-0.13
1691		0.59	0.05
31283		0.59	0.20
9968		0.59	0.90
13289		0.59	-0.29
6928		0.59	-0.60
13335		0.59	-1.13
11427		0.59	0.38
3450		0.59	-0.25
13933		0.59	-0.12
9635		0.59	1.12
10284		0.59	1.31
5184		0.59	0.15
9021		0.59	0.38
17475		0.59	0.57
14000		0.59	0.59
9916		0.59	0.78
10669		0.59	0.32
	17641	0.59	0.43
4351		0.59	0.51
33493		0.59	0.80
	16820	0.58	0.06

	6257	0.58	2.01
6701		0.58	0.24
11337		0.58	-0.17
7414		0.58	1.05
13720		0.58	1.28
14686		0.58	1.28
	12959	0.58	-2.58
8746		0.58	0.62
14626		0.58	0.01
	16133	0.58	0.00
12448		0.58	-0.40
15475		0.58	0.48
30104		0.58	1.34
3594		0.58	0.00
33312		0.58	0.52
4548		0.58	0.97
4414		0.58	-0.36
10574		0.58	0.38
	3353	0.58	1.96
15236		0.58	1.10
10349		0.58	-1.11
14949		0.58	0.75
6392		0.58	-0.01
8931		0.58	-0.97
15214		0.58	-0.51
	8474	0.58	1.50
3513		0.58	0.36
8149		0.58	-0.29
3077		0.58	1.96
7206		0.58	-0.96
	8032	0.58	-0.34
13133		0.58	0.38
6962		0.58	-0.61
18540		0.58	0.95
31790		0.58	-0.96
8062		0.58	0.09
	6622	0.58	-0.60
4249		0.58	-0.61
3119		0.58	0.76
12156		0.58	0.51
8348		0.58	0.65
18777		0.58	0.32
14487		0.58	-0.94
31856		0.58	0.07
32033		0.58	2.23

3595		0.58	1.47
32973		0.58	0.81
9784		0.58	-0.22
7052		0.58	-1.02
7593		0.58	0.45
31213		0.58	1.45
7394		0.58	-0.24
12372		0.58	0.78
	2554	0.58	-0.13
17328		0.58	0.42
3161		0.58	0.22
11964		0.58	0.59
9003		0.58	-0.48
5896		0.58	0.03
	8366	0.58	-0.18
6323		0.58	0.49
2781		0.58	0.73
3605		0.58	-1.26
31204		0.57	0.64
3396		0.57	0.05
14496		0.57	0.46
8727		0.57	0.15
13633		0.57	1.47
11236		0.57	-0.61
7714		0.57	-0.85
32715		0.57	0.91
	8766	0.57	0.84
13848		0.57	0.30
5064		0.57	0.13
11678		0.57	-0.79
3281		0.57	0.88
4648		0.57	0.97
10240		0.57	0.26
13889		0.57	1.01
9928		0.57	0.37
13614		0.57	0.54
5588		0.57	-0.85
30296		0.57	1.10
32975		0.57	0.87
	20098	0.57	0.48
12068		0.57	-0.17
7289		0.57	1.00
10910		0.57	0.02
14614		0.57	-1.23
6977		0.57	0.29

17227		0.57	0.66
	2633	0.57	1.06
	9787	0.57	-1.16
13446		0.57	0.66
31381		0.57	0.23
	5601	0.57	0.23
31183		0.57	-0.70
31223		0.57	0.47
12153		0.57	1.12
17991		0.57	0.25
6606		0.57	-0.03
7108		0.57	0.97
16766		0.57	-1.39
	11537	0.57	0.92
7162		0.57	0.02
9964		0.57	0.26
3632		0.57	0.62
6449		0.57	-1.01
9001		0.57	-0.47
8641		0.56	1.07
	6176	0.56	-0.68
6917		0.56	0.58
3209		0.56	1.83
32432		0.56	-1.17
9899		0.56	-0.68
1799		0.56	-0.62
	17109	0.56	1.22
12612		0.56	-0.23
13170		0.56	0.81
15149		0.56	0.81
32174		0.56	1.01
7992		0.56	0.71
	10357	0.56	0.44
9761		0.56	0.45
33336		0.56	-0.39
13079		0.56	0.24
5932		0.56	-0.21
11500		0.56	0.34
33146		0.56	-0.66
33481		0.56	0.41
1480		0.56	0.81
32343		0.56	-1.24
13996		0.56	1.06
2277		0.56	-0.26
30356		0.56	1.18

5740		0.56	0.20
3332		0.56	-0.04
15436		0.56	-2.15
14959		0.56	-0.16
7326		0.56	0.76
12005		0.56	-0.15
12214		0.56	-0.28
10250		0.56	0.73
7538		0.56	1.48
16848		0.56	-1.80
6425		0.56	-1.70
11148		0.56	-0.28
12230		0.56	0.70
11585		0.56	-0.43
9216		0.56	1.42
32688		0.56	0.78
30440		0.56	0.05
4111		0.56	0.56
6115		0.56	0.17
10346		0.56	0.06
14506		0.56	0.64
30278		0.56	0.75
2958		0.56	0.45
	9558	0.56	1.04
7260		0.56	0.11
	19232	0.56	-1.12
17173		0.56	0.62
6199		0.56	-0.24
4943		0.55	-0.11
4806		0.55	0.00
3775		0.55	1.81
	9396	0.55	-0.90
3644		0.55	-1.09
12289		0.55	-0.02
6011		0.55	-0.09
5264		0.55	0.62
10354		0.55	0.05
5276		0.55	0.43
6014		0.55	-1.25
	19389	0.55	-2.84
30142		0.55	0.23
13391		0.55	0.24
17763		0.55	1.05
6318		0.55	0.80
14704		0.55	-0.02

7076		0.55	0.12
6258		0.55	0.59
31043		0.55	-0.71
12242		0.55	-0.92
3018		0.55	0.21
	5595	0.55	-0.55
4148		0.55	0.23
32732		0.55	-2.14
	18638	0.55	-0.15
7786		0.55	0.50
3941		0.55	2.12
4647		0.55	0.62
	15091	0.55	1.02
13662		0.55	0.63
32513		0.55	1.05
13137		0.55	0.48
12425		0.55	1.78
33205		0.55	-0.75
2107		0.55	0.25
1939		0.55	0.30
3921		0.55	-0.25
1155		0.55	0.92
1785		0.54	1.17
17570		0.54	1.99
4926		0.54	0.12
1748		0.54	1.09
6502		0.54	-0.68
30496		0.54	1.06
15081		0.54	1.10
4303		0.54	1.25
3073		0.54	1.09
14789		0.54	1.15
8711		0.54	0.79
17652		0.54	-0.05
4527		0.54	-0.47
5802		0.54	0.22
31858		0.54	0.48
33174		0.54	-0.98
15314		0.54	0.27
32676		0.54	-1.20
11589		0.54	0.01
5890		0.54	-0.21
7927		0.54	0.54
10142		0.54	-0.02
32019		0.54	0.84

13206		0.54	0.69
7671		0.54	0.16
10844		0.54	0.53
7540		0.54	0.47
4679		0.54	0.91
8395		0.54	-0.43
10197		0.54	0.61
6483		0.54	-0.14
14142		0.54	0.83
15093		0.54	0.65
4213		0.54	0.63
18806		0.54	0.58
17510		0.54	-2.94
3288		0.54	-0.07
33041		0.54	-0.48
9594		0.54	0.42
31774		0.54	0.83
31371		0.54	1.40
11400		0.54	-0.61
	10999	0.54	0.33
2957		0.54	0.12
7029		0.54	0.69
14149		0.54	0.38
15443		0.54	-0.21
13344		0.54	0.52
11768		0.54	0.91
32603		0.54	0.12
30389		0.54	-0.56
10116		0.54	0.50
7586		0.54	0.86
6462		0.54	-0.31
32187		0.54	0.27
8905		0.54	0.22
4521		0.54	0.81
31004		0.54	-0.16
13923		0.54	0.09
3277		0.54	0.65
10244		0.54	-0.11
1771		0.54	1.04
11556		0.53	-1.74
	9506	0.53	1.84
8593		0.53	0.82
31397		0.53	1.69
31020		0.53	0.78
7319		0.53	1.29

12749		0.53	1.49
12410		0.53	-0.48
31902		0.53	1.17
17531		0.53	1.02
3579		0.53	-0.23
14322		0.53	0.01
7458		0.53	0.63
14843		0.53	-1.12
31883		0.53	0.49
11621		0.53	-0.01
31315		0.53	0.78
4684		0.53	0.27
14839		0.53	0.21
	197	0.53	-0.32
	10095	0.53	0.79
3461		0.53	1.90
5818		0.53	-0.04
	4683	0.53	-0.01
8544		0.53	-2.00
	18309	0.53	0.61
7564		0.53	0.65
8862		0.53	-0.35
6668		0.53	-0.26
32813		0.53	0.29
10261		0.53	0.71
9630		0.53	-1.98
30371		0.53	-0.43
6589		0.53	-0.20
15728		0.53	-0.28
5002		0.53	0.50
6238		0.53	-0.05
	11643	0.53	-0.02
31189		0.53	-0.25
13842		0.53	-0.28
32023		0.53	-0.57
4095		0.53	0.82
1139		0.53	0.94
16997		0.53	-2.10
9908		0.53	0.59
30443		0.53	-1.02
17903		0.53	-2.57
10406		0.53	0.24
	19645	0.53	1.75
14812		0.53	0.71
3448		0.53	-0.19

14521		0.53	-0.35
3061		0.53	1.05
6523		0.53	0.24
7772		0.53	-0.11
1004		0.53	-0.37
	6337	0.53	0.02
11797		0.53	-0.16
2118		0.53	1.77
3809		0.53	0.17
12470		0.53	-0.28
	6286	0.53	0.93
31187		0.53	0.65
10981		0.53	-1.43
13421		0.52	0.42
1705		0.52	0.14
33156		0.52	-0.67
5295		0.52	0.54
15611		0.52	1.01
13670		0.52	0.34
33291		0.52	0.34
4073		0.52	0.19
3278		0.52	0.59
6629		0.52	0.26
14981		0.52	0.98
15406		0.52	-1.26
5373		0.52	-0.65
16836		0.52	0.29
15260		0.52	-0.06
8300		0.52	0.05
5391		0.52	-0.47
30358		0.52	0.38
31632		0.52	0.17
	12176	0.52	-0.80
9766		0.52	-0.51
14766		0.52	-0.99
7252		0.52	0.52
10423		0.52	1.04
6750		0.52	-1.09
7496		0.52	0.08
	4964	0.52	0.45
3808		0.52	-0.37
14400		0.52	0.61
17764		0.52	0.67
5451		0.52	0.00
32275		0.52	-0.16

17867		0.52	0.37
	8822	0.52	0.54
10344		0.52	0.41
12701		0.52	0.37
6095		0.52	2.33
30456		0.52	0.72
17596		0.52	-1.36
9952		0.52	0.63
11042		0.52	0.11
4181		0.52	0.04
14885		0.52	0.66
3430		0.52	0.66
18518		0.52	0.18
11069		0.52	0.91
9474		0.52	1.57
2998		0.52	0.51
3999		0.52	0.16
15422		0.51	0.82
6608		0.51	-0.38
33477		0.51	1.47
	5203	0.51	-0.14
3926		0.51	0.34
4789		0.51	0.35
4312		0.51	-0.84
4759		0.51	0.30
2150		0.51	0.88
10761		0.51	-0.13
11144		0.51	0.38
8571		0.51	0.70
6784		0.51	0.73
14199		0.51	0.79
1977		0.51	0.90
8822		0.51	-0.21
12132		0.51	0.48
11235		0.51	-0.75
1600		0.51	0.84
9044		0.51	-0.42
3905		0.51	-0.31
3726		0.51	-0.34
	14917	0.51	1.04
1527		0.51	1.24
18418		0.51	0.28
17737		0.51	2.05
7075		0.51	0.76
8768		0.51	0.39

8013		0.51	-0.28
9269		0.51	-0.75
7941		0.51	1.52
12201		0.51	1.03
13217		0.51	1.03
13284		0.51	0.82
13978		0.51	0.26
12934		0.51	0.74
	8818	0.51	0.92
32188		0.51	-0.02
32512		0.51	-0.63
7384		0.51	-0.36
32812		0.51	-0.31
7304		0.51	-0.39
9581		0.51	1.46
17075		0.51	1.21
14402		0.51	0.31
14786		0.51	-0.37
18445		0.51	0.44
	14631	0.51	-0.66
18104		0.51	0.14
7467		0.51	-0.53
	9480	0.51	1.09
18315		0.51	1.23
30370		0.51	0.56
1780		0.51	-0.73
	20286	0.51	-0.18
31840		0.51	0.83
3397		0.51	0.68
6291		0.50	-0.57
6030		0.50	0.18
8837		0.50	0.67
15626		0.50	0.12
4362		0.50	0.57
17462		0.50	0.87
4214		0.50	-0.55
7614		0.50	0.54
9331		0.50	-0.02
4407		0.50	-0.46
	10700	0.50	1.12
11665		0.50	0.27
4026		0.50	0.18
4552		0.50	-0.70
18095		0.50	-0.02
4083		0.50	-0.28

3347		0.50	0.55
2493		0.50	1.93
16717		0.50	-0.48
15007		0.50	0.96
31846		0.50	-0.44
4288		0.50	0.91
13577		0.50	0.65
17923		0.50	-2.27
32775		0.50	-1.36
9084		0.50	-0.82
12509		0.50	-1.24
11727		0.50	2.08
14585		0.50	0.24
	910	0.50	0.25
3099		0.50	1.03
17657		0.50	0.54
8654		0.50	-1.62
7338		0.50	-0.21
8039		0.50	-0.06
5641		0.50	0.98
4650		0.50	0.40
	12579	0.50	0.68
9224		0.50	-0.84
16987		0.50	-1.69
17048		0.50	-1.18
6300		0.50	0.52
33286		0.50	0.94
31919		0.50	2.49
30055		0.50	-0.94
14375		0.50	-0.01
12261		0.50	-2.23
	15708	0.50	0.66
13716		0.50	-0.26
7103		0.50	0.68
9081		0.50	-0.77
7201		0.50	0.67
31869		0.50	1.09
5618		0.50	0.78
17030		0.50	-1.15
8611		0.49	-2.02
	2384	0.49	0.42
	19643	0.49	1.83
	18414	0.49	0.86
4116		0.49	0.44
6244		0.49	0.16

33340		0.49	0.88
12327		0.49	1.03
12974		0.49	-0.02
6197		0.49	0.00
31679		0.49	-0.25
32544		0.49	1.07
9684		0.49	0.45
	2468	0.49	1.54
11010		0.49	0.92
30436		0.49	1.39
7535		0.49	0.53
9643		0.49	0.81
32351		0.49	1.36
18358		0.49	2.04
10137		0.49	0.63
32955		0.49	0.45
10903		0.49	-0.42
7577		0.49	0.47
4316		0.49	0.62
31857		0.49	-0.02
5929		0.49	-0.84
5109		0.49	-0.16
6349		0.49	0.23
5034		0.49	-0.33
15545		0.49	0.75
9716		0.49	1.06
16931		0.49	0.01
31269		0.49	0.42
4810		0.49	0.37
	16025	0.49	0.98
8485		0.49	-0.53
13087		0.49	-0.20
8007		0.49	1.00
4496		0.49	-0.28
31262		0.49	0.44
6604		0.49	0.00
11474		0.49	-0.16
14791		0.49	1.02
17780		0.49	0.53
11781		0.49	0.06
13576		0.49	-0.26
7628		0.49	0.81
4159		0.49	0.55
14823		0.49	1.15
32944		0.49	0.03

10790		0.48	1.64
9356		0.48	-0.86
8361		0.48	0.74
33071		0.48	-0.51
2852		0.48	-1.07
1048		0.48	0.14
7802		0.48	0.65
1279		0.48	-2.24
	17718	0.48	-0.03
8202		0.48	-0.11
14405		0.48	0.55
17294		0.48	0.84
33095		0.48	0.79
6966		0.48	-0.73
9119		0.48	-0.30
	2634	0.48	1.30
2092		0.48	-0.26
	8259	0.48	-0.74
30473		0.48	-0.57
32957		0.48	1.44
11473		0.48	0.67
14167		0.48	-0.88
4520		0.48	0.84
7049		0.48	-0.90
	966	0.48	0.80
	12089	0.48	-0.73
32811		0.48	0.65
14519		0.48	0.97
4892		0.48	-1.94
1471		0.48	0.60
14568		0.48	0.00
3054		0.48	0.99
15162		0.48	0.08
3109		0.48	-0.28
	9177	0.48	-0.76
7676		0.48	0.77
8806		0.48	-0.46
	16921	0.48	-2.12
14795		0.48	0.75
	18678	0.48	-0.22
12520		0.48	1.73
3082		0.48	0.54
	8755	0.48	-0.95
4439		0.48	0.23
	15094	0.48	1.33

14030		0.48	-1.80
1106		0.48	-1.43
13321		0.48	1.57
6936		0.48	0.35
31678		0.48	1.07
10694		0.48	-1.59
4120		0.48	0.63
5971		0.48	0.55
6743		0.48	-0.16
17230		0.48	0.09
	12022	0.48	1.34
	3439	0.48	1.91
7272		0.48	-1.88
3994		0.48	-0.61
5402		0.48	-1.03
8814		0.48	0.61
10140		0.47	0.99
	11627	0.47	0.05
14215		0.47	0.08
13389		0.47	0.27
	6310	0.47	1.56
	16773	0.47	-0.66
31829		0.47	0.80
14244		0.47	0.05
12387		0.47	-0.82
31446		0.47	-0.40
	6314	0.47	0.79
31461		0.47	0.53
	5911	0.47	-1.07
17769		0.47	0.44
	10929	0.47	-0.01
6515		0.47	0.11
	16976	0.47	0.86
30441		0.47	-2.07
33120		0.47	0.47
3035		0.47	1.24
13618		0.47	0.88
2616		0.47	2.38
17932		0.47	0.36
11762		0.47	0.51
8646		0.47	-0.02
13178		0.47	-2.93
15676		0.47	0.84
	15349	0.47	-0.68
4597		0.47	0.29

31563		0.47	-1.38
30098		0.47	0.34
31717		0.47	0.41
32026		0.47	0.50
	2742	0.47	0.71
3936		0.47	0.03
33303		0.47	-0.37
10281		0.47	2.11
6803		0.47	0.09
15916		0.47	0.94
2505		0.47	1.28
30083		0.47	0.29
11814		0.47	0.96
7480		0.47	0.00
	13934	0.47	0.73
4692		0.47	-1.26
30268		0.47	1.01
12797		0.47	-0.68
31363		0.47	1.14
1622		0.47	0.71
10449		0.47	1.52
9165		0.47	0.86
12551		0.47	0.82
17336		0.47	-0.01
13685		0.47	-1.67
5882		0.47	0.23
4169		0.47	-0.20
4040		0.47	0.67
12316		0.46	0.71
	7614	0.46	0.24
14021		0.46	-1.45
10138		0.46	0.18
10597		0.46	0.26
8960		0.46	0.92
6265		0.46	-0.68
5475		0.46	0.41
11230		0.46	0.75
3274		0.46	0.14
32306		0.46	1.85
1059		0.46	0.14
5532		0.46	0.30
17826		0.46	0.14
31126		0.46	-0.46
15390		0.46	1.19
3557		0.46	-0.64

12723		0.46	0.28
7745		0.46	0.03
6543		0.46	-0.55
32384		0.46	-0.63
15011		0.46	0.13
15865		0.46	0.43
3333		0.46	0.09
11409		0.46	1.72
12127		0.46	1.02
	8399	0.46	0.82
15414		0.46	-0.47
8871		0.46	1.26
9245		0.46	-0.12
4096		0.46	1.08
13671		0.46	0.22
8201		0.46	0.57
30289		0.46	0.14
15371		0.46	0.79
8332		0.46	0.10
6768		0.46	0.95
33226		0.46	-1.68
2113		0.46	0.15
10651		0.46	1.03
8366		0.46	1.10
12540		0.46	-0.24
8994		0.46	0.13
13728		0.46	0.62
11131		0.46	1.15
10967		0.46	-1.52
14436		0.46	0.42
	14008	0.46	-2.99
11567		0.46	0.98
17721		0.46	0.91
7766		0.46	0.71
30477		0.46	0.58
7646		0.46	0.04
7660		0.46	-0.02
4217		0.46	-1.87
5123		0.46	-1.83
9166		0.46	0.48
5434		0.46	0.02
	4604	0.46	0.78
	1001	0.46	-0.15
14644		0.46	-0.31
31672		0.46	0.19

3319		0.46	-0.55
	11502	0.45	1.06
6769		0.45	2.00
9499		0.45	-0.38
32858		0.45	1.02
32451		0.45	0.20
14213		0.45	
3669		0.45	-1.14
30171		0.45	0.70
8114		0.45	0.50
	11731	0.45	1.31
1742		0.45	-0.62
31395		0.45	-0.17
4063		0.45	-0.55
13758		0.45	1.06
8726		0.45	0.14
30120		0.45	0.43
32183		0.45	-0.61
6987		0.45	-0.32
8374		0.45	1.51
6432		0.45	1.16
	9590	0.45	-0.01
	8855	0.45	0.03
10687		0.45	-0.46
32444		0.45	0.30
	7110	0.45	0.80
7959		0.45	-0.06
9936		0.45	0.36
4829		0.45	0.67
15764		0.45	0.87
6330		0.45	-0.17
7348		0.45	0.36
17208		0.45	1.77
30111		0.45	-0.10
17633		0.45	-0.73
8231		0.45	2.23
7878		0.45	-0.14
2930		0.45	0.15
	19167	0.45	-1.73
7068		0.45	2.10
4395		0.45	-0.22
7872		0.45	-0.22
12836		0.45	0.57
3216		0.45	0.50
11661		0.45	-0.42

2846		0.45	0.30
3091		0.45	0.25
3051		0.45	3.24
32561		0.45	1.07
1591		0.44	-0.43
	16941	0.44	0.91
3502		0.44	-1.15
	2490	0.44	0.28
	16455	0.44	-0.32
14010		0.44	-0.48
11694		0.44	-0.32
10188		0.44	-0.03
11582		0.44	0.64
10853		0.44	0.05
8249		0.44	0.90
18549		0.44	-0.13
9764		0.44	0.06
	5913	0.44	0.10
9717		0.44	1.20
18186		0.44	-1.10
5413		0.44	0.19
31173		0.44	-1.83
18371		0.44	-0.23
3297		0.44	0.04
10674		0.44	0.59
	15089	0.44	0.72
4599		0.44	0.01
31684		0.44	0.84
6733		0.44	-0.99
10943		0.44	0.59
10989		0.44	0.00
9104		0.44	-0.05
6885		0.44	0.87
13252		0.44	-0.11
7856		0.44	-1.71
10986		0.44	-0.53
15893		0.44	0.49
14772		0.44	-0.99
2381		0.44	0.25
1072		0.44	0.44
3182		0.44	0.63
	5178	0.44	0.07
2790		0.44	0.68
33319		0.44	0.78
11184		0.44	0.58

3314		0.44	-1.26
31075		0.44	-0.76
8772		0.44	0.34
7070		0.44	0.60
13318		0.44	1.17
6209		0.44	-0.38
1397		0.44	0.38
17598		0.44	-1.66
16935		0.44	1.19
4323		0.44	0.66
	10365	0.44	0.25
12004		0.43	0.59
13209		0.43	0.27
14678		0.43	-0.57
14128		0.43	0.40
18268		0.43	-0.47
	7658	0.43	-1.63
15553		0.43	1.44
12703		0.43	0.25
10619		0.43	1.09
14074		0.43	0.47
	8878	0.43	-1.31
9523		0.43	0.57
33160		0.43	0.54
15468		0.43	-1.49
5076		0.43	1.14
32394		0.43	1.27
17988		0.43	-0.50
7440		0.43	0.09
32593		0.43	-0.19
	6871	0.43	-1.85
1935		0.43	-0.65
	2532	0.43	-0.04
	3020	0.43	-0.60
2621		0.43	2.03
30450		0.43	-0.44
31101		0.43	0.35
	19780	0.43	1.05
31624		0.43	1.00
6772		0.43	-0.75
3348		0.43	-2.28
3533		0.43	0.16
	14011	0.43	0.28
13924		0.43	0.97
3842		0.43	-1.08

17226		0.43	-0.34
	19023	0.43	-1.96
2097		0.43	-0.96
12129		0.43	0.82
12276		0.43	-1.37
7367		0.43	-0.33
11663		0.43	-1.14
5110		0.43	-0.93
31322		0.43	-1.95
	7628	0.43	0.69
9512		0.43	-0.91
3419		0.43	0.58
7255		0.43	0.65
31728		0.43	1.01
3434		0.43	0.52
32868		0.43	0.44
3504		0.43	-1.17
5073		0.43	1.13
12017		0.43	0.89
14820		0.43	0.99
12420		0.43	0.12
14760		0.43	0.18
17064		0.43	0.17
3971		0.43	-0.23
6602		0.43	-0.90
33209		0.43	0.92
17770		0.43	0.65
14913		0.43	-0.86
6859		0.43	0.68
6426		0.43	0.33
32446		0.43	-0.36
12020		0.43	0.65
6633		0.43	-1.37
1373		0.42	0.14
10447		0.42	0.55
12402		0.42	0.69
	3018	0.42	-1.35
6384		0.42	1.41
32209		0.42	-0.48
8962		0.42	-0.55
9707		0.42	0.09
9169		0.42	1.12
31209		0.42	0.56
31962		0.42	0.18
14556		0.42	0.30

5748		0.42	-0.13
	13963	0.42	-1.45
12013		0.42	-0.21
4125		0.42	-0.26
9771		0.42	-0.22
7595		0.42	0.86
3223		0.42	-0.02
9861		0.42	-1.38
12164		0.42	0.05
9828		0.42	-1.02
10508		0.42	-1.28
4715		0.42	1.26
30393		0.42	-0.49
3972		0.42	-0.05
	16501	0.42	0.50
31817		0.42	0.87
1804		0.42	0.84
9286		0.42	-0.08
	10029	0.42	-1.43
3295		0.42	0.69
32799		0.42	-0.91
14028		0.42	-1.62
11280		0.42	-0.18
	8644	0.42	1.58
13062		0.42	-1.39
4978		0.42	0.06
	16996	0.42	0.91
7736		0.42	0.58
7707		0.42	-0.46
14855		0.42	0.64
6129		0.42	-0.64
16978		0.42	0.27
10895		0.42	0.00
32119		0.42	-0.06
1512		0.42	-0.51
14696		0.42	0.43
14816		0.42	0.54
	6710	0.42	0.25
9791		0.42	-0.32
15097		0.42	0.31
13227		0.42	1.17
8986		0.41	-0.16
8359		0.41	0.50
1342		0.41	-0.13
2041		0.41	-1.62

12872		0.41	1.14
11802		0.41	0.82
7907		0.41	0.71
13155		0.41	0.95
14437		0.41	0.03
33085		0.41	-0.68
15738		0.41	0.41
4903		0.41	0.52
7668		0.41	0.51
3423		0.41	-0.63
	19641	0.41	0.82
33282		0.41	-0.06
	19808	0.41	-1.02
31638		0.41	1.59
15757		0.41	0.83
3431		0.41	0.56
14485		0.41	0.09
17580		0.41	0.53
13314		0.41	0.81
6081		0.41	0.50
30052		0.41	0.50
7145		0.41	0.17
10958		0.41	0.65
31871		0.41	0.87
33088		0.41	0.87
9016		0.41	0.05
6367		0.41	0.17
	13188	0.41	0.72
	3621	0.41	2.00
2925		0.41	-1.31
15441		0.41	-0.12
6168		0.41	0.31
32692		0.41	-0.24
8697		0.41	1.07
5539		0.41	-0.19
9436		0.41	0.14
16964		0.41	1.00
6198		0.41	0.82
14438		0.41	0.00
6978		0.41	0.04
	13181	0.41	1.25
31150		0.41	-1.12
	20207	0.41	-2.19
32488		0.41	-1.87
12357		0.41	-1.21

	10899	0.41	-0.01
13190		0.41	0.80
12173		0.41	0.88
31205		0.41	-1.48
11007		0.41	0.83
8739		0.41	0.73
2034		0.41	1.26
	10902	0.41	0.49
7709		0.41	-0.72
	18819	0.41	0.70
4830		0.41	0.18
	20293	0.41	0.10
	15791	0.41	-0.19
8072		0.41	-0.46
10210		0.41	-0.53
	13976	0.41	-0.87
4954		0.41	0.19
5250		0.41	-1.26
18773		0.41	0.40
31349		0.41	0.56
9290		0.41	0.96
32418		0.41	-1.43
4265		0.41	0.79
11475		0.41	1.26
	17711	0.41	0.92
32643		0.41	0.55
31878		0.40	-0.26
11963		0.40	0.59
10764		0.40	-0.14
5062		0.40	-0.64
33225		0.40	0.79
	20150	0.40	-0.99
5683		0.40	-0.03
30095		0.40	0.25
	8802	0.40	-0.45
11297		0.40	-0.93
11574		0.40	-0.31
17295		0.40	0.65
	2744	0.40	0.19
15020		0.40	0.22
13809		0.40	1.26
	2097	0.40	-1.13
31028		0.40	1.58
9921		0.40	0.22
3723		0.40	-0.80

	2675	0.40	-0.33
3483		0.40	-1.12
17754		0.40	1.44
6281		0.40	1.00
32041		0.40	0.14
8869		0.40	0.84
16800		0.40	0.00
9572		0.40	2.52
	8496	0.40	-0.26
1427		0.40	0.26
6414		0.40	0.40
11821		0.40	0.98
12325		0.40	1.16
1736		0.40	-1.35
10702		0.40	0.53
4094		0.40	-0.54
13271		0.40	-0.11
	20110	0.40	-0.25
14242		0.40	0.94
15482		0.40	0.01
13834		0.40	0.50
	7350	0.40	-1.15
3307		0.40	-0.62
9273		0.40	-0.37
9012		0.40	-1.15
31791		0.40	0.37
1883		0.40	0.94
7552		0.40	1.09
3402		0.40	-1.24
11229		0.40	1.17
8740		0.40	2.03
5756		0.40	0.52
17834		0.40	-0.28
	13006	0.40	-1.20
13931		0.40	-0.24
11255		0.40	-0.38
15715		0.40	0.10
12848		0.40	-0.03
1058		0.40	0.43
12016		0.40	1.12
6279		0.40	0.16
1909		0.40	0.24
3725		0.40	0.13
6340		0.39	0.20
7940		0.39	0.91

2048		0.39	2.27
	223	0.39	-0.28
11092		0.39	1.24
10026		0.39	-0.12
30125		0.39	1.02
11141		0.39	0.37
17577		0.39	-0.23
	16858	0.39	0.65
8800		0.39	1.24
31385		0.39	0.02
	6206	0.39	0.82
12358		0.39	-0.33
	1389	0.39	1.02
31174		0.39	-0.72
5826		0.39	-0.08
1403		0.39	0.38
4814		0.39	0.03
7340		0.39	-0.03
3126		0.39	0.46
33179		0.39	-0.29
15465		0.39	-0.79
2104		0.39	0.49
31088		0.39	1.13
17273		0.39	-0.94
18538		0.39	1.04
3239		0.39	0.67
32230		0.39	-1.90
6579		0.39	1.18
31991		0.39	0.95
1132		0.39	0.25
6703		0.39	-0.59
11659		0.39	-0.74
4285		0.39	0.90
	3415	0.39	-0.32
12953		0.39	-0.60
5645		0.39	0.67
10520		0.39	1.00
4525		0.39	0.68
11163		0.39	-0.17
14527		0.39	1.29
4914		0.39	-0.16
14518		0.39	1.79
8334		0.39	-1.90
	10711	0.39	0.20
8236		0.39	0.59

1759		0.39	-0.62
12467		0.39	0.01
32954		0.39	-0.93
8866		0.39	-0.43
5675		0.39	-0.14
32140		0.39	-0.24
4695		0.39	-0.32
31814		0.39	-0.22
9727		0.39	2.09
4099		0.39	-0.09
18356		0.39	0.98
10383		0.38	0.10
7233		0.38	0.14
5137		0.38	0.82
12012		0.38	0.85
11444		0.38	-0.10
11509		0.38	1.42
1865		0.38	0.88
1148		0.38	0.61
1007		0.38	0.46
5247		0.38	-0.30
5083		0.38	-0.33
4594		0.38	0.71
	17998	0.38	1.64
30363		0.38	0.16
30009		0.38	0.96
8097		0.38	-0.01
1747		0.38	0.94
15880		0.38	1.05
3441		0.38	0.12
30485		0.38	0.26
8958		0.38	0.55
	2581	0.38	-0.16
31065		0.38	0.01
3140		0.38	-0.79
	12270	0.38	1.56
	8293	0.38	-0.26
14541		0.38	-0.32
3460		0.38	1.78
31410		0.38	0.02
8956		0.38	1.04
3522		0.38	-0.86
7460		0.38	0.46
4666		0.38	1.01
3074		0.38	0.80

10387		0.38	-0.02
14798		0.38	0.26
10198		0.38	0.58
31736		0.38	0.07
1119		0.38	0.83
12219		0.38	0.49
15758		0.38	-0.21
6545		0.38	0.69
12350		0.38	0.29
	15395	0.38	0.71
7303		0.38	0.51
	11632	0.38	0.41
6688		0.38	0.33
4259		0.38	-0.23
9196		0.38	-0.05
	18761	0.38	0.60
10428		0.38	-0.09
16868		0.38	0.81
7036		0.38	-2.21
9083		0.38	0.44
17985		0.38	0.97
7503		0.38	0.06
13884		0.38	0.19
3589		0.38	-0.08
	8287	0.38	-1.15
13036		0.38	-0.13
7800		0.37	0.50
	12083	0.37	-0.70
	3406	0.37	-0.72
	19217	0.37	-0.81
6794		0.37	-0.95
17564		0.37	0.49
31002		0.37	-0.11
32822		0.37	1.49
	3469	0.37	0.58
18482		0.37	1.23
14715		0.37	-1.45
	18382	0.37	0.65
7748		0.37	0.86
13004		0.37	1.24
12524		0.37	0.00
18269		0.37	0.06
32212		0.37	0.74
	12175	0.37	-0.55
1809		0.37	1.02

30266		0.37	0.67
31745		0.37	-0.07
2163		0.37	1.37
32221		0.37	0.12
11426		0.37	0.23
9475		0.37	0.89
31081		0.37	1.32
9988		0.37	0.55
13617		0.37	0.10
10922		0.37	0.32
31556		0.37	0.01
9705		0.37	-0.25
1031		0.37	0.23
8328		0.37	0.93
5499		0.37	0.60
	20137	0.37	-1.49
10051		0.37	-1.39
	13300	0.37	1.37
	10043	0.37	-1.42
15013		0.37	1.06
1464		0.37	0.69
12274		0.37	0.92
12344		0.37	0.57
8490		0.37	-0.05
31281		0.37	-1.02
17807		0.37	0.66
5920		0.37	-0.05
17084		0.37	0.16
	6722	0.37	-0.96
31954		0.37	0.49
32671		0.37	1.15
5292		0.37	-0.07
1693		0.37	0.45
8065		0.37	-0.43
7402		0.37	0.48
3041		0.37	0.55
	5185	0.37	
14851		0.37	0.08
	6105	0.37	0.56
33135		0.37	-0.70
2258		0.37	0.00
8169		0.36	-0.57
	5034	0.36	-0.14
15343		0.36	0.50
16827		0.36	0.83

1801		0.36	0.18
13403		0.36	0.02
	2065	0.36	0.83
4394		0.36	0.24
5302		0.36	0.63
7855		0.36	0.61
15743		0.36	-1.62
	14260	0.36	0.67
17599		0.36	-0.96
17061		0.36	0.39
14964		0.36	-1.85
9519		0.36	-0.80
1380		0.36	-0.05
32207		0.36	0.70
12310		0.36	0.41
30403		0.36	-0.13
6406		0.36	0.74
10300		0.36	0.33
17333		0.36	0.86
6413		0.36	-0.24
	17409	0.36	-0.20
10524		0.36	-2.30
2194		0.36	0.82
14238		0.36	0.96
17027		0.36	-0.89
	8489	0.36	-0.19
6680		0.36	-0.04
33259		0.36	0.09
8972		0.36	-1.12
10424		0.36	-0.14
14282		0.36	-1.48
2706		0.36	-0.12
4787		0.36	-0.23
7381		0.36	0.51
	6119	0.36	-0.41
7275		0.36	0.34
2302		0.36	0.08
7977		0.36	0.54
10969		0.36	1.23
7806		0.36	0.16
31510		0.36	-1.29
2668		0.36	0.53
	7653	0.36	0.23
31034		0.36	-1.64
10617		0.36	0.40

12622		0.36	0.89
	3384	0.36	0.46
2534		0.36	1.39
5659		0.35	0.55
4618		0.35	0.97
31821		0.35	0.65
31033		0.35	1.18
9042		0.35	-0.84
17192		0.35	0.19
5664		0.35	-0.15
15882		0.35	0.51
10944		0.35	1.26
7519		0.35	-0.23
	18338	0.35	0.25
8977		0.35	-0.50
11840		0.35	0.05
	19531	0.35	1.27
2822		0.35	-0.24
31619		0.35	1.23
6937		0.35	0.77
6678		0.35	0.18
30184		0.35	0.87
	11380	0.35	0.20
	8854	0.35	0.31
7125		0.35	1.08
6311		0.35	0.15
7093		0.35	-0.16
15725		0.35	-0.94
5735		0.35	-0.34
	20328	0.35	0.18
11371		0.35	-0.78
31050		0.35	1.11
9672		0.35	-0.15
9018		0.35	-1.47
	10434	0.35	-0.75
	5374	0.35	1.32
	8476	0.35	0.66
5414		0.35	-0.26
	12883	0.35	0.31
3837		0.35	-1.17
2003		0.35	0.54
	7568	0.35	-0.72
7479		0.35	-0.14
5745		0.35	1.61
15667		0.35	0.60

32717		0.35	-0.27
2841		0.35	-1.61
11710		0.35	0.20
31547		0.35	-0.44
13601		0.35	-0.87
32150		0.35	0.66
32810		0.35	0.31
12143		0.35	0.18
13648		0.35	0.15
12929		0.35	-0.31
8399		0.35	-0.22
10795		0.35	1.98
8363		0.35	-0.46
14955		0.35	1.37
3712		0.35	0.27
13501		0.35	0.14
15829		0.35	-1.10
31747		0.35	0.96
6391		0.35	-1.51
	8846	0.35	0.67
6522		0.35	-0.60
10186		0.35	0.45
33106		0.35	-0.47
	9510	0.35	1.07
	19330	0.35	-1.75
33284		0.35	0.81
12008		0.34	0.36
	8279	0.34	-0.30
11323		0.34	1.62
3421		0.34	0.39
4900		0.34	1.45
33113		0.34	0.46
	11514	0.34	-0.95
31561		0.34	1.16
6354		0.34	-0.29
7004		0.34	-0.90
12231		0.34	0.72
11722		0.34	0.63
8644		0.34	1.04
10425		0.34	0.88
30412		0.34	-1.43
11759		0.34	1.25
32543		0.34	0.85
11980		0.34	0.40
12750		0.34	0.70

17364		0.34	1.19
	7243	0.34	1.35
31118		0.34	-0.35
	18303	0.34	0.64
12323		0.34	0.61
4836		0.34	0.52
8462		0.34	-0.44
7961		0.34	0.39
	10505	0.34	0.97
8415		0.34	3.27
6202		0.34	-0.01
	17338	0.34	-1.26
8910		0.34	0.22
7738		0.34	-0.89
14829		0.34	0.32
12855		0.34	0.98
	4083	0.34	-1.59
14013		0.34	0.40
	5289	0.34	1.26
10711		0.34	0.98
31534		0.34	-0.13
31445		0.34	0.58
31030		0.34	2.15
18416		0.34	0.59
13405		0.34	-0.57
5300		0.34	0.24
32103		0.34	0.31
12632		0.34	-2.02
32591		0.34	-0.05
6416		0.33	0.49
7533		0.33	0.16
10374		0.33	0.02
8693		0.33	-0.29
	11676	0.33	-0.72
30321		0.33	-1.22
4158		0.33	-0.25
15773		0.33	0.53
	4713	0.33	1.41
14103		0.33	1.08
13097		0.33	-2.69
7998		0.33	-0.87
6424		0.33	
1792		0.33	1.12
15571		0.33	0.50
8327		0.33	0.10

3981		0.33	1.00
9582		0.33	-0.68
10641		0.33	0.02
1463		0.33	0.11
8594		0.33	-0.35
9919		0.33	0.19
15234		0.33	1.20
14830		0.33	0.45
12993		0.33	0.86
18404		0.33	-0.51
7581		0.33	0.59
4699		0.33	1.47
12875		0.33	0.35
3608		0.33	0.57
	16453	0.33	-0.26
6064		0.33	-0.10
6194		0.33	2.05
33096		0.33	-0.28
10964		0.33	-0.54
15148		0.33	0.60
15362		0.33	-0.47
13936		0.33	0.58
9380		0.33	0.29
3566		0.33	0.44
9078		0.33	0.77
6024		0.33	0.61
3367		0.33	0.66
11275		0.33	1.59
7311		0.33	-1.43
3764		0.33	0.16
10738		0.33	-0.99
7360		0.33	0.31
30365		0.33	0.75
17569		0.33	-0.56
1332		0.33	0.08
	2524	0.33	0.51
30355		0.33	0.45
	18183	0.33	0.34
8400		0.33	-0.16
32095		0.33	0.73
3365		0.33	0.40
6927		0.33	-0.71
13374		0.33	0.49
	3383	0.33	0.92
5753		0.33	0.08

	2369	0.33	-2.59
7625		0.33	-0.23
8250		0.33	-0.31
13490		0.33	-0.33
1718		0.33	0.62
2040		0.32	0.00
11261		0.32	0.39
1242		0.32	0.90
11696		0.32	1.61
6298		0.32	0.16
16914		0.32	0.07
13535		0.32	0.95
4950		0.32	-0.54
5390		0.32	0.32
4276		0.32	-0.49
6653		0.32	0.55
3186		0.32	0.62
2254		0.32	-0.32
3871		0.32	-1.51
9710		0.32	-0.19
6802		0.32	0.94
17856		0.32	-0.25
	12488	0.32	0.43
12086		0.32	-0.63
6506		0.32	-0.08
18555		0.32	-0.70
2052		0.32	1.05
	8516	0.32	-0.46
13154		0.32	0.22
13026		0.32	0.99
11188		0.32	1.01
1422		0.32	-0.25
	13007	0.32	-1.07
	5320	0.32	0.96
14723		0.32	0.39
4645		0.32	-0.97
9712		0.32	0.06
12106		0.32	0.82
33061		0.32	-0.20
3228		0.32	1.06
8813		0.32	0.95
30087		0.32	-0.46
16995		0.32	-0.05
17060		0.32	-0.42
4132		0.32	-0.73

18437		0.32	0.43
7330		0.32	-1.48
8331		0.32	-1.38
7549		0.32	0.02
	5334	0.32	0.29
32983		0.32	0.09
12843		0.32	0.56
16787		0.32	0.86
	16124	0.32	1.21
10016		0.32	1.51
5935		0.32	-0.41
12301		0.32	-0.04
1041		0.32	0.52
6933		0.31	-0.73
10627		0.31	1.21
31096		0.31	-0.11
31000		0.31	0.70
6974		0.31	1.43
14802		0.31	0.31
32064		0.31	-0.10
	3087	0.31	1.06
7622		0.31	0.54
	11137	0.31	-0.85
15141		0.31	-0.01
10522		0.31	0.17
5933		0.31	-0.33
12505		0.31	1.35
1231		0.31	-0.87
31969		0.31	-0.31
4953		0.31	0.64
10859		0.31	1.02
10414		0.31	0.47
	17200	0.31	0.28
30388		0.31	0.53
5378		0.31	0.41
14598		0.31	-1.80
13871		0.31	-0.99
13859		0.31	1.12
8884		0.31	-0.43
6172		0.31	0.17
31464		0.31	0.32
8781		0.31	1.01
7562		0.31	0.75
30170		0.31	0.47
12959		0.31	-1.47

14440		0.31	0.84
2045		0.31	0.31
14574		0.31	-1.11
9366		0.31	0.69
1079		0.31	-0.32
15485		0.31	-1.52
10333		0.31	-0.20
14801		0.31	0.37
10260		0.31	0.88
31207		0.31	0.02
	7231	0.31	0.58
9013		0.31	0.43
15130		0.31	0.80
4608		0.31	1.91
6898		0.31	0.00
9976		0.31	-1.97
2028		0.31	1.87
5560		0.31	0.23
14177		0.30	0.68
31013		0.30	-0.19
6998		0.30	0.37
5140		0.30	0.26
31351		0.30	0.31
11380		0.30	-0.82
3427		0.30	0.08
4660		0.30	-0.92
3167		0.30	-0.29
	16016	0.30	1.46
1524		0.30	0.56
2533		0.30	1.81
11929		0.30	0.09
5493		0.30	-0.64
10072		0.30	-1.36
	4285	0.30	-0.74
10155		0.30	0.15
10928		0.30	0.73
2931		0.30	1.10
16755		0.30	0.25
14063		0.30	0.84
8276		0.30	-1.55
15528		0.30	0.92
2225		0.30	-0.01
	11776	0.30	0.34
9938		0.30	0.87
11570		0.30	-2.18

	11911	0.30	-1.88
31516		0.30	0.67
32113		0.30	0.01
	9441	0.30	-1.09
4147		0.30	0.47
	10030	0.30	-0.85
9926		0.30	-0.72
8285		0.30	-0.20
4848		0.30	-0.12
	10421	0.30	-1.36
	10663	0.30	0.06
8449		0.30	-0.10
31507		0.30	0.36
12346		0.30	0.05
	5868	0.30	0.55
14252		0.30	-1.32
	17627	0.30	-0.19
	6266	0.30	1.04
6477		0.30	-0.01
17221		0.30	0.44
31004		0.30	0.58
9887		0.30	-0.65
30464		0.30	-2.55
14788		0.30	1.32
14567		0.30	-0.07
11456		0.30	1.04
6961		0.30	0.54
	3337	0.30	0.90
9422		0.30	0.05
33487		0.30	-0.43
11674		0.30	0.70
10516		0.30	0.18
5006		0.30	-0.02
1803		0.30	0.50
16899		0.30	-0.54
17921		0.30	-0.02
9993		0.30	0.88
	13337	0.30	0.65
7399		0.30	0.84
31048		0.30	1.96
32507		0.30	0.01
11233		0.30	0.79
	11583	0.30	0.48
18467		0.30	0.58
7097		0.30	-0.06

	10294	0.30	-0.63
7395		0.30	1.00
3569		0.30	-0.05
31079		0.30	1.14
	17114	0.30	-1.05
2656		0.30	-0.71
30195		0.29	1.63
1246		0.29	1.02
14652		0.29	0.11
32720		0.29	0.26
14661		0.29	0.75
1406		0.29	0.09
9235		0.29	-0.75
16912		0.29	0.19
14670		0.29	0.23
7769		0.29	0.65
11173		0.29	-1.40
1908		0.29	-0.02
13672		0.29	0.35
	13293	0.29	0.95
	12170	0.29	-1.32
	20441	0.29	-0.19
4445		0.29	-0.98
7191		0.29	0.10
	6476	0.29	1.81
32986		0.29	1.13
9231		0.29	0.55
8799		0.29	1.29
1044		0.29	0.88
8105		0.29	2.34
9793		0.29	0.13
31441		0.29	0.56
1916		0.29	-0.80
15102		0.29	0.29
15394		0.29	0.26
9483		0.29	0.43
12128		0.29	1.22
11887		0.29	-0.71
6251		0.29	0.85
	8824	0.29	0.50
	9356	0.29	-0.91
1327		0.29	0.55
	1474	0.29	1.15
4935		0.29	-0.99
10834		0.29	-0.05

2043		0.29	0.16
1745		0.29	0.57
2127		0.29	0.73
11573		0.29	0.80
4337		0.29	-0.11
33474		0.29	0.74
8928		0.29	-0.15
15697		0.29	1.15
13887		0.29	0.82
	20177	0.29	-0.94
4942		0.29	0.70
14888		0.29	-0.72
	6561	0.29	-0.42
15550		0.29	-0.56
8717		0.29	-0.24
31908		0.29	0.68
18801		0.29	-0.19
7143		0.29	0.56
11327		0.29	0.73
7609		0.29	0.38
16756		0.29	-1.40
17321		0.29	0.19
9441		0.29	0.66
	19652	0.29	1.32
	12856	0.28	-1.60
2093		0.28	0.10
	2646	0.28	0.15
1499		0.28	0.08
9314		0.28	0.49
31750		0.28	0.14
33038		0.28	0.76
	7875	0.28	0.34
10177		0.28	0.54
	3016	0.28	0.88
	18283	0.28	0.78
10816		0.28	1.22
11212		0.28	0.51
30119		0.28	0.74
12698		0.28	0.53
17376		0.28	-1.62
11160		0.28	0.29
15100		0.28	-0.37
12030		0.28	-0.08
12131		0.28	0.93
3400		0.28	-0.02

11374		0.28	-0.81
1561		0.28	1.37
10742		0.28	-0.57
7287		0.28	1.28
8878		0.28	-0.17
33229		0.28	0.26
4415		0.28	-1.01
1668		0.28	-0.95
7588		0.28	0.96
	195	0.28	0.32
31357		0.28	-1.37
15124		0.28	-1.76
5724		0.28	1.69
	13475	0.28	0.86
3935		0.28	-0.13
8430		0.28	-0.39
4468		0.28	-1.64
12066		0.28	-0.88
5812		0.28	0.41
18012		0.28	0.05
11695		0.28	0.69
15615		0.28	0.57
33311		0.28	0.67
10365		0.28	-0.90
30093		0.28	0.32
	6383	0.28	-0.82
33491		0.28	-0.69
	14969	0.28	0.86
15480		0.28	1.35
3815		0.28	1.34
30011		0.28	1.51
	11474	0.28	-1.08
10093		0.27	0.81
6762		0.27	0.08
8831		0.27	0.90
3071		0.27	-0.37
5180		0.27	0.11
13245		0.27	0.40
	13711	0.27	0.21
11458		0.27	-0.95
10082		0.27	-0.30
7788		0.27	0.20
14529		0.27	0.51
	7528	0.27	-0.84
15252		0.27	-0.29

7942		0.27	0.89
5704		0.27	0.35
4341		0.27	1.60
11462		0.27	1.38
11048		0.27	1.13
32350		0.27	-1.95
7820		0.27	0.15
31894		0.27	0.63
3578		0.27	-0.12
	7063	0.27	-2.39
12259		0.27	-1.48
3649		0.27	-0.12
7120		0.27	0.57
	2710	0.27	0.95
10512		0.27	-1.41
9065		0.27	-1.02
8189		0.27	0.74
17572		0.27	0.41
12462		0.27	-0.25
	13480	0.27	0.91
10247		0.27	-0.35
8246		0.27	0.42
8577		0.27	0.21
18233		0.27	-0.59
16713		0.27	0.57
15207		0.27	1.07
6320		0.27	0.88
15433		0.27	0.44
11796		0.27	1.23
14632		0.27	-1.39
14983		0.27	0.23
32219		0.27	0.33
8245		0.27	-0.45
13316		0.27	0.44
14463		0.27	0.21
	20288	0.27	-0.07
5403		0.27	-2.55
7978		0.27	0.95
7144		0.27	0.16
7385		0.27	0.19
17738		0.27	0.02
	10340	0.27	1.51
17835		0.27	0.69
17260		0.27	0.84
31294		0.26	0.45

12617		0.26	0.97
31099		0.26	-0.46
1828		0.26	-0.32
6833		0.26	0.04
17853		0.26	-1.33
13431		0.26	1.05
14734		0.26	1.20
13043		0.26	-2.66
13078		0.26	1.29
14411		0.26	0.11
31933		0.26	0.32
15308		0.26	-0.96
8032		0.26	0.43
11842		0.26	0.38
14191		0.26	0.80
5322		0.26	-0.60
13306		0.26	1.38
1712		0.26	0.52
7044		0.26	0.97
33128		0.26	0.07
9311		0.26	0.83
	12475	0.26	0.07
17450		0.26	0.46
6646		0.26	0.83
3264		0.26	0.24
14597		0.26	1.45
	8587	0.26	-0.60
33255		0.26	0.65
7267		0.26	1.42
13726		0.26	1.10
10477		0.26	0.34
6910		0.26	0.80
17196		0.26	0.59
7104		0.26	-0.55
6857		0.26	-0.16
1927		0.26	-0.30
13995		0.26	0.75
7079		0.26	-0.40
12523		0.26	0.51
	12018	0.26	-0.51
30130		0.26	-1.44
11430		0.26	1.14
33265		0.26	1.31
15457		0.26	-1.33
10670		0.26	-0.49

7111		0.26	1.12
31475		0.26	0.68
3458		0.26	1.64
7536		0.26	0.77
6819		0.26	-0.27
9186		0.26	0.02
30170		0.26	-0.04
9253		0.26	0.76
15418		0.26	0.14
17781		0.26	-0.79
10883		0.26	-0.90
3658		0.26	0.29
2112		0.26	-0.91
5008		0.26	1.46
7532		0.26	0.00
18213		0.26	0.28
32659		0.26	-0.63
10725		0.26	-0.49
4357		0.26	0.84
32068		0.26	1.83
15551		0.26	0.87
9296		0.25	-0.01
17124		0.25	0.48
11175		0.25	0.77
	6237	0.25	0.06
31700		0.25	-1.04
32463		0.25	-0.42
14586		0.25	-0.23
	14666	0.25	-0.93
12082		0.25	0.09
7669		0.25	-0.19
13111		0.25	0.58
30079		0.25	0.34
12688		0.25	-0.23
7431		0.25	0.16
	9475	0.25	2.27
32713		0.25	0.99
10659		0.25	0.10
8505		0.25	0.89
12390		0.25	0.62
8764		0.25	0.73
5047		0.25	0.19
8314		0.25	-1.71
14204		0.25	0.58
8386		0.25	-0.17

6051		0.25	0.00
9150		0.25	-0.07
	3427	0.25	0.78
	7098	0.25	0.16
1513		0.25	-0.22
5494		0.25	1.50
10673		0.25	-0.89
30492		0.25	-0.19
1105		0.25	0.18
10713		0.25	1.07
32198		0.25	-0.53
14747		0.25	-1.15
32747		0.25	-0.60
	16100	0.25	0.56
31822		0.25	-0.18
17919		0.25	-0.80
	5555	0.25	-1.54
4463		0.25	0.84
1416		0.25	0.21
30050		0.25	-1.11
7430		0.25	0.51
	7638	0.25	-0.61
5389		0.25	-0.51
13454		0.25	0.26
33473		0.25	-0.57
10005		0.25	1.66
10118		0.25	0.65
31975		0.25	0.06
31792		0.25	-0.23
10328		0.25	0.05
	20092	0.25	-0.10
17648		0.25	0.04
4080		0.25	0.54
3630		0.25	-0.49
16716		0.25	-0.57
	3523	0.25	-2.03
9029		0.25	-0.20
31875		0.25	-0.30
6486		0.25	-0.59
4637		0.25	0.32
15573		0.25	0.44
1921		0.25	-0.42
7262		0.25	1.55
30479		0.25	0.87
12151		0.25	1.14

6362		0.25	-0.33
6805		0.25	0.34
32445		0.25	0.11
5786		0.25	-0.01
	16188	0.25	2.32
31087		0.25	-0.01
31723		0.25	0.01
1165		0.25	0.45
4591		0.24	0.11
3893		0.24	0.44
17284		0.24	1.08
13228		0.24	0.04
12262		0.24	-0.09
	5653	0.24	0.33
12250		0.24	-1.29
9299		0.24	0.75
32604		0.24	0.60
10045		0.24	-0.91
1660		0.24	0.91
30277		0.24	0.63
14916		0.24	0.55
13200		0.24	-1.63
3668		0.24	-0.69
31062		0.24	-1.29
32206		0.24	0.71
3735		0.24	0.28
13902		0.24	0.48
	18546	0.24	-0.81
9636		0.24	0.86
13334		0.24	0.61
	10778	0.24	-1.42
10257		0.24	-0.77
12054		0.24	0.84
7755		0.24	0.27
12767		0.24	1.21
13423		0.24	-0.23
11466		0.24	0.76
11448		0.24	-0.40
2139		0.24	0.79
8678		0.24	-1.48
1074		0.24	-0.48
11838		0.24	1.40
4133		0.24	0.55
	8768	0.24	0.37
4523		0.24	0.56

12172		0.24	0.18
13738		0.24	1.28
17256		0.24	0.47
15438		0.24	0.63
13192		0.24	-0.53
	14589	0.24	0.11
14512		0.24	0.60
11018		0.24	-1.26
13136		0.24	0.87
12373		0.24	0.35
4817		0.24	-0.20
14225		0.24	0.19
33161		0.24	-0.06
9076		0.24	0.56
12001		0.24	-0.29
7749		0.24	1.66
9688		0.24	-0.45
4065		0.24	-0.99
9501		0.23	-0.86
7397		0.23	0.92
13310		0.23	1.57
13012		0.23	0.86
	16562	0.23	-0.57
31932		0.23	1.10
2157		0.23	0.77
9327		0.23	-0.08
14838		0.23	0.69
8481		0.23	-0.98
14235		0.23	-0.05
	10022	0.23	-0.77
5773		0.23	0.42
32918		0.23	0.42
8776		0.23	-1.12
6617		0.23	-0.19
12405		0.23	0.72
9580		0.23	0.87
7013		0.23	0.32
5556		0.23	0.03
12374		0.23	0.09
8581		0.23	-1.99
14796		0.23	0.41
10481		0.23	2.29
3292		0.23	-0.23
13658		0.23	0.37
13956		0.23	1.17

31086		0.23	0.35
11958		0.23	0.63
1309		0.23	0.43
31758		0.23	0.34
9007		0.23	0.57
9330		0.23	0.41
7904		0.23	2.04
	3657	0.23	-0.11
18787		0.23	0.33
8671		0.23	-0.29
14280		0.23	-1.61
	3690	0.23	0.75
	2650	0.23	0.31
14316		0.23	0.07
14759		0.23	-1.30
7492		0.23	-2.57
8325		0.23	-0.46
18493		0.23	0.29
9759		0.23	-0.12
32356		0.23	0.58
32040		0.23	1.04
6364		0.23	-0.61
7785		0.23	0.65
14778		0.23	0.03
4465		0.23	-0.39
5072		0.23	0.58
6575		0.23	-0.88
32226		0.23	1.38
	2821	0.23	-0.78
13185		0.23	-0.48
14900		0.23	-1.72
18335		0.23	-1.35
5819		0.23	3.69
	15803	0.23	-1.29
14899		0.23	-2.20
12052		0.23	-0.96
3337		0.23	0.02
32836		0.23	0.07
4145		0.23	-0.06
33482		0.22	0.18
31157		0.22	0.22
4783		0.22	0.19
	19233	0.22	-0.54
16890		0.22	-0.47
6761		0.22	0.25

8808		0.22	-1.93
10123		0.22	0.17
6438		0.22	0.65
11259		0.22	-1.27
16857		0.22	-0.63
7636		0.22	-0.82
33013		0.22	0.20
14505		0.22	0.71
5336		0.22	0.34
	5709	0.22	-1.92
8487		0.22	-0.79
30192		0.22	-1.08
15454		0.22	0.12
8401		0.22	1.05
30409		0.22	-1.57
17122		0.22	0.71
3980		0.22	0.80
	983	0.22	0.17
5984		0.22	-0.08
15073		0.22	0.14
12338		0.22	0.91
3301		0.22	-0.52
32588		0.22	-0.78
	19552	0.22	-0.18
13937		0.22	0.88
33140		0.22	-0.12
33263		0.22	0.19
8557		0.22	-1.13
17153		0.22	-0.42
9006		0.22	-1.85
7494		0.22	-0.09
4267		0.22	0.81
	7730	0.22	0.67
14627		0.22	-0.43
32118		0.22	0.40
15804		0.22	-0.71
	12962	0.22	-1.44
17752		0.22	1.69
17191		0.22	-0.21
13270		0.22	0.01
10961		0.22	-1.62
	11797	0.22	0.94
4225		0.22	0.65
6324		0.22	-0.04
1268		0.22	0.44

7839		0.22	-0.02
7309		0.22	-1.04
12609		0.22	0.80
5614		0.22	-0.56
	19367	0.22	0.53
17928		0.22	0.11
31873		0.22	0.15
16719		0.22	-0.34
1824		0.22	-0.68
15364		0.22	1.11
10845		0.22	0.31
	2819	0.22	0.61
17660		0.22	-0.05
15155		0.22	0.29
12674		0.22	-2.33
10249		0.22	-0.99
3991		0.22	-0.84
15554		0.22	0.30
2955		0.22	-0.45
	19895	0.22	0.37
11470		0.22	-1.49
8938		0.22	0.15
30279		0.22	0.60
	6648	0.21	0.17
5554		0.21	-0.08
10523		0.21	0.49
3002		0.21	0.17
3851		0.21	-0.36
2525		0.21	-0.01
30446		0.21	-0.56
3335		0.21	-0.11
15110		0.21	-0.03
	9916	0.21	-0.04
7437		0.21	0.24
12487		0.21	-1.84
18042		0.21	-0.53
	7103	0.21	0.82
13317		0.21	0.69
8858		0.21	1.16
7157		0.21	-0.71
31926		0.21	-0.31
4590		0.21	0.40
12355		0.21	-0.41
14273		0.21	0.47
16778		0.21	0.21

10154		0.21	0.44
	11619	0.21	-0.31
18190		0.21	0.22
5866		0.21	1.26
32250		0.21	-0.22
3153		0.21	0.09
7524		0.21	-0.60
5938		0.21	0.04
10253		0.21	0.26
5842		0.21	0.27
6415		0.21	0.72
4625		0.21	0.88
	5747	0.21	0.41
12643		0.21	-1.69
4662		0.21	0.75
32508		0.21	-0.36
8736		0.21	0.49
15473		0.21	-0.18
30282		0.21	-0.50
18506		0.21	0.03
4020		0.21	0.45
30364		0.21	0.37
33328		0.21	1.60
4203		0.21	0.20
33250		0.21	0.18
7725		0.21	0.36
	3637	0.21	2.10
13223		0.21	0.33
5436		0.21	0.25
6702		0.21	-0.29
33273		0.21	-1.12
7740		0.21	0.53
13023		0.21	1.06
6233		0.21	0.61
14257		0.21	0.49
11126		0.21	0.75
3782		0.21	-1.21
	11084	0.21	-1.39
7831		0.21	0.40
32070		0.21	-1.31
5686		0.21	1.17
	5351	0.21	0.68
	756	0.21	-0.49
32389		0.21	0.16
3776		0.21	0.15

6096		0.21	0.58
5654		0.21	0.03
	12483	0.21	-0.10
14184		0.21	-0.77
3564		0.21	1.37
9345		0.21	1.11
8418		0.21	0.43
14190		0.21	0.19
17803		0.21	-0.16
13135		0.21	0.21
32136		0.21	-0.96
15253		0.21	0.31
11399		0.21	0.79
17029		0.21	-0.47
7664		0.20	1.24
3029		0.20	-0.17
	18598	0.20	-0.03
32352		0.20	0.82
5912		0.20	0.74
	2990	0.20	-1.00
18734		0.20	0.22
11859		0.20	0.21
15641		0.20	-0.29
12860		0.20	0.53
8590		0.20	0.01
2677		0.20	-0.04
13385		0.20	0.47
9033		0.20	-0.45
1620		0.20	0.71
32919		0.20	0.29
7196		0.20	-0.63
7744		0.20	0.43
9631		0.20	0.94
7627		0.20	0.38
8254		0.20	-1.51
31876		0.20	-0.53
10628		0.20	1.11
	14315	0.20	-0.30
2665		0.20	0.15
2219		0.20	1.08
8362		0.20	0.53
2199		0.20	-0.27
11811		0.20	2.65
3920		0.20	0.62
4653		0.20	0.15

32697		0.20	1.16
1587		0.20	-0.58
2913		0.20	0.21
9459		0.20	-0.28
4621		0.20	-1.02
7462		0.20	0.24
13061		0.20	-1.13
12905		0.20	1.15
33333		0.20	1.41
	12688	0.20	0.86
9853		0.20	-0.28
3060		0.20	0.41
13168		0.20	0.22
11956		0.20	0.77
18496		0.20	1.82
3679		0.20	0.00
7912		0.20	0.33
15169		0.20	0.07
13545		0.20	-0.92
	631	0.20	-2.00
3541		0.20	-0.24
9283		0.20	0.80
8330		0.20	-0.78
9116		0.20	-0.55
10109		0.20	0.70
6664		0.20	0.49
7715		0.20	-0.31
14470		0.20	-0.62
8652		0.20	0.01
12075		0.20	-0.48
4761		0.20	0.35
7442		0.20	0.86
10632		0.20	-0.48
5561		0.20	0.10
	16265	0.20	1.26
32398		0.20	0.16
4901		0.20	0.70
13811		0.20	0.05
7814		0.20	0.45
18678		0.20	-1.34
8516		0.20	-0.40
11913		0.20	0.33
	3715	0.20	0.02
6289		0.19	0.24
5342		0.19	-1.73

18249		0.19	0.36
1545		0.19	0.17
12861		0.19	0.59
10862		0.19	0.54
	12487	0.19	0.20
30386		0.19	0.67
15082		0.19	0.72
4812		0.19	-0.44
9735		0.19	0.07
	430	0.19	-1.51
9191		0.19	0.08
	13938	0.19	-2.03
2137		0.19	1.19
12275		0.19	-1.71
	15303	0.19	-1.27
5848		0.19	-0.23
3724		0.19	-0.46
13035		0.19	0.14
4429		0.19	-0.58
5348		0.19	-0.17
10417		0.19	0.93
31693		0.19	0.69
15151		0.19	0.75
17904		0.19	-1.17
4381		0.19	-1.04
31217		0.19	0.42
4747		0.19	0.88
3204		0.19	0.73
14343		0.19	0.95
14176		0.19	-0.13
17332		0.19	0.02
14672		0.19	0.70
	3437	0.19	1.25
9163		0.19	0.70
31119		0.19	-0.64
	20280	0.19	-0.07
30121		0.19	0.16
	13364	0.19	1.30
14442		0.19	0.18
14085		0.19	-0.59
	4272	0.19	-2.02
9028		0.19	0.96
14824		0.19	-0.78
	13415	0.19	0.50
11982		0.19	0.55

	14612	0.19	0.00
5807		0.19	0.07
11155		0.19	-1.09
3773		0.19	-1.29
5242		0.19	0.57
6322		0.19	0.43
2120		0.19	0.86
3044		0.19	1.08
6321		0.19	0.65
12296		0.19	-0.59
12352		0.19	1.08
31748		0.19	0.66
5961		0.19	0.14
8766		0.19	1.56
	7866	0.19	-1.03
5480		0.19	0.64
	3378	0.19	0.31
	9922	0.19	0.28
30359		0.19	-0.87
10993		0.19	-0.69
14367		0.19	-0.11
16993		0.19	-0.12
8320		0.19	1.68
15378		0.19	0.66
31085		0.19	-0.19
1832		0.19	1.05
31763		0.19	-0.13
33131		0.19	0.61
9307		0.19	0.94
	20263	0.19	-0.31
30197		0.19	0.58
1474		0.19	0.19
9962		0.19	0.81
15208		0.19	-0.01
	6241	0.19	-1.38
13906		0.18	0.05
11949		0.18	-0.11
	10646	0.18	0.76
10019		0.18	-0.03
7834		0.18	-0.17
	5200	0.18	1.05
8533		0.18	0.67
13014		0.18	0.38
14476		0.18	1.11
5214		0.18	0.00

	3347	0.18	2.02
6514		0.18	0.75
32541		0.18	0.01
18578		0.18	0.28
10882		0.18	0.47
1395		0.18	-1.29
	16199	0.18	0.82
15354		0.18	0.28
8948		0.18	-1.71
7804		0.18	0.64
5913		0.18	-0.60
9448		0.18	1.84
3362		0.18	0.65
8983		0.18	-1.89
13876		0.18	-1.03
9521		0.18	-0.35
11064		0.18	0.62
15138		0.18	0.07
1688		0.18	-0.46
9047		0.18	-0.02
9484		0.18	-0.10
8308		0.18	0.76
15224		0.18	-0.11
18296		0.18	1.06
	12663	0.18	-1.76
31637		0.18	0.77
1518		0.18	0.41
	12325	0.18	0.15
18139		0.18	0.53
1982		0.18	0.41
1961		0.18	0.72
5193		0.18	-0.48
32833		0.18	-0.16
5212		0.18	-0.06
32392		0.18	0.18
4846		0.18	-0.48
14945		0.18	-1.53
3973		0.18	0.16
4314		0.18	0.61
17150		0.18	0.85
11109		0.18	0.27
	16487	0.18	0.00
12942		0.18	-0.23
8493		0.18	-1.91
12926		0.18	-0.85

6644		0.18	0.93
10734		0.18	0.22
	9852	0.18	0.53
16782		0.18	-0.78
32145		0.18	0.36
6022		0.18	-0.25
32225		0.18	-2.03
	14639	0.18	-2.23
3303		0.18	0.01
12984		0.18	1.08
18102		0.17	0.21
4853		0.17	0.64
1262		0.17	1.07
12728		0.17	-0.67
	19162	0.17	-1.29
9410		0.17	0.61
	7203	0.17	0.66
8455		0.17	1.24
9218		0.17	0.70
	1136	0.17	1.04
30046		0.17	0.54
14677		0.17	-0.31
5316		0.17	0.81
14662		0.17	0.64
9135		0.17	0.07
5501		0.17	0.19
10949		0.17	0.81
	17468	0.17	-0.38
12076		0.17	-0.84
7339		0.17	-0.09
8824		0.17	-0.87
	3204	0.17	-0.01
14029		0.17	-1.72
11968		0.17	-0.14
7823		0.17	-0.69
31077		0.17	-0.55
3544		0.17	0.32
	3413	0.17	-0.94
31721		0.17	-1.31
6070		0.17	-0.43
4017		0.17	-1.52
31483		0.17	0.66
5915		0.17	0.95
	19528	0.17	-0.17
5958		0.17	0.57

15783		0.17	-0.10
11895		0.17	-0.80
9358		0.17	0.32
	13522	0.17	0.79
14613		0.17	-1.43
11437		0.17	0.91
32344		0.17	0.90
6863		0.17	0.24
4563		0.17	-0.44
10675		0.17	-2.41
2264		0.17	-1.12
14869		0.17	-0.25
12862		0.17	0.03
5568		0.17	0.33
	1882	0.17	1.24
14780		0.17	-0.10
13443		0.17	0.22
14995		0.17	-1.52
10852		0.17	-0.19
5206		0.17	0.35
4486		0.17	1.24
14528		0.17	0.91
1154		0.17	0.30
8116		0.17	1.62
7570		0.17	-1.52
33454		0.17	0.66
15353		0.17	0.02
9456		0.17	0.11
17765		0.17	0.37
12399		0.17	0.54
	11871	0.17	0.78
	13243	0.17	0.77
31543		0.16	-0.42
6938		0.16	1.22
32457		0.16	-0.32
	12017	0.16	0.08
6439		0.16	-0.36
1796		0.16	-1.00
9506		0.16	0.98
9313		0.16	0.48
	10973	0.16	0.24
15871		0.16	-0.46
5122		0.16	0.16
7615		0.16	0.13
	8136	0.16	-2.14

32554		0.16	0.25
31522		0.16	-0.85
	10898	0.16	0.53
1116		0.16	0.01
18616		0.16	-0.25
11293		0.16	0.41
15319		0.16	1.43
	9666	0.16	0.42
31493		0.16	-1.59
5861		0.16	2.29
14636		0.16	0.07
2158		0.16	-1.28
12501		0.16	-0.37
31045		0.16	1.27
14755		0.16	1.71
6211		0.16	3.17
11861		0.16	0.20
18251		0.16	0.09
8985		0.16	-2.02
8329		0.16	0.09
5156		0.16	0.31
32031		0.16	-0.47
32374		0.16	-0.18
	4549	0.16	-0.35
7045		0.16	-1.90
14774		0.16	0.47
15552		0.16	0.55
17234		0.16	0.91
9446		0.16	1.00
9540		0.16	0.60
10083		0.16	-0.17
9825		0.16	-0.24
14212		0.16	
33082		0.16	1.32
10950		0.16	-0.33
4183		0.16	0.66
18472		0.16	-0.46
14423		0.16	0.86
9565		0.16	1.79
18332		0.16	0.16
1964		0.16	-0.33
6601		0.16	-0.23
32687		0.16	0.94
8351		0.16	-0.85
33116		0.16	-0.86

31058		0.16	-0.41
8224		0.16	0.39
33066		0.16	1.26
3097		0.16	0.78
7257		0.16	0.01
32387		0.16	0.58
1934		0.16	-0.82
	8568	0.16	1.42
32027		0.16	0.47
	3425	0.16	-0.01
9281		0.16	-1.50
	14388	0.16	-0.83
12232		0.16	1.38
30471		0.16	0.17
8095		0.16	-0.10
5003		0.16	0.52
32658		0.16	-2.35
17970		0.16	-0.62
10064		0.16	-1.16
5962		0.16	0.15
2021		0.16	0.53
	6306	0.16	-0.01
18480		0.15	0.43
2932		0.15	1.24
4807		0.15	0.14
7842		0.15	-0.27
32232		0.15	0.03
5427		0.15	0.20
7484		0.15	-1.08
30428		0.15	0.22
10681		0.15	-0.50
2124		0.15	0.60
5729		0.15	1.46
15235		0.15	1.17
8058		0.15	1.43
7248		0.15	-1.73
32600		0.15	0.20
2889		0.15	-0.49
10098		0.15	0.38
	17179	0.15	-0.55
13277		0.15	0.19
13634		0.15	0.03
6637		0.15	-0.58
12298		0.15	-0.52
17579		0.15	-1.08

15861		0.15	0.15
5516		0.15	-1.29
10939		0.15	-0.03
11874		0.15	-1.39
8112		0.15	1.04
9177		0.15	0.49
1387		0.15	0.63
31538		0.15	0.15
	11489	0.15	1.14
32380		0.15	-1.75
	16113	0.15	0.88
	6189	0.15	0.14
4851		0.15	-0.33
16969		0.15	1.60
9666		0.15	-1.09
17145		0.15	-1.24
8836		0.15	0.85
12114		0.15	-1.27
	6552	0.15	-1.16
	11617	0.15	0.18
13329		0.15	0.12
3224		0.15	-0.61
13325		0.15	0.49
30070		0.15	0.81
13917		0.15	-0.16
13432		0.15	0.71
8083		0.15	-0.03
4919		0.15	-0.95
32175		0.15	-0.38
8355		0.15	-1.49
13664		0.15	0.90
1239		0.15	-0.42
1081		0.15	-0.06
11883		0.15	-0.08
	984	0.15	0.00
32405		0.15	0.80
	12661	0.15	0.77
7760		0.15	0.36
	19472	0.15	0.60
30329		0.15	-1.88
10789		0.15	1.42
3351		0.14	0.16
	6208	0.14	0.72
17593		0.14	-1.14
9353		0.14	-0.04

10633		0.14	-1.81
	14956	0.14	-0.27
4654		0.14	-0.44
11555		0.14	-1.06
14906		0.14	-0.75
15141		0.14	0.58
32137		0.14	-0.42
10559		0.14	-0.26
	14119	0.14	0.94
2165		0.14	0.81
	4692	0.14	-0.01
1582		0.14	0.56
8010		0.14	0.90
32988		0.14	0.20
8486		0.14	-0.15
7146		0.14	0.71
3251		0.14	0.53
6140		0.14	0.66
	11501	0.14	0.90
33490		0.14	-0.74
2904		0.14	0.23
30456		0.14	-0.42
17109		0.14	0.43
32089		0.14	-0.49
11306		0.14	0.13
1882		0.14	-0.79
8165		0.14	-0.03
11308		0.14	-0.60
	13165	0.14	-1.88
2448		0.14	-0.81
	2024	0.14	0.76
	12578	0.14	0.76
10962		0.14	0.99
33098		0.14	-0.84
8093		0.14	0.64
7305		0.14	0.24
13130		0.14	0.14
11892		0.14	-0.51
15596		0.14	0.25
	12323	0.14	1.33
	10049	0.14	-2.23
	14914	0.14	1.57
33256		0.14	1.04
31959		0.14	0.23
5481		0.14	-1.18

32700		0.14	0.42
18554		0.14	-0.26
32661		0.14	-0.10
33003		0.14	-1.02
	18875	0.14	0.64
12832		0.14	0.82
30493		0.14	-0.80
32055		0.14	0.37
13478		0.14	1.13
33307		0.14	-1.25
5245		0.14	0.24
3006		0.14	-0.49
15695		0.14	0.77
8207		0.14	-0.75
4054		0.14	-0.03
10050		0.14	1.55
5483		0.14	1.58
16961		0.14	-0.80
15099		0.14	-0.31
7857		0.14	-0.18
3969		0.14	-0.44
10596		0.14	-0.74
1662		0.14	1.33
4241		0.14	0.15
7347		0.13	-1.23
15144		0.13	-0.15
	470	0.13	-1.16
11669		0.13	0.12
6214		0.13	-0.56
32726		0.13	-0.07
6778		0.13	0.67
	12495	0.13	0.77
10360		0.13	-0.49
11203		0.13	0.54
	9443	0.13	1.04
31288		0.13	-1.77
4462		0.13	0.94
33139		0.13	-0.59
	1060	0.13	-0.65
3738		0.13	-1.13
7375		0.13	0.18
1478		0.13	0.87
15530		0.13	0.18
10280		0.13	1.14
7387		0.13	-0.28

	13340	0.13	0.99
13094		0.13	1.05
4107		0.13	-0.18
12560		0.13	0.28
14286		0.13	0.03
17745		0.13	2.37
14794		0.13	0.48
8957		0.13	0.94
30414		0.13	-1.24
12018		0.13	0.03
18085		0.13	0.10
18302		0.13	0.99
1885		0.13	-0.47
18402		0.13	0.36
	7873	0.13	0.24
14162		0.13	-0.13
33122		0.13	0.69
11529		0.13	0.73
11423		0.13	-0.94
14109		0.13	-0.08
14054		0.13	0.97
	12884	0.13	0.63
6113		0.13	-0.83
	13664	0.13	0.28
4947		0.13	0.50
	9254	0.13	-1.54
10029		0.13	-0.37
	14245	0.13	0.27
11353		0.13	0.82
30007		0.13	-0.50
	13253	0.13	-0.56
5474		0.13	0.02
30049		0.13	0.46
12124		0.13	0.00
	7421	0.13	-0.54
9444		0.13	1.85
7461		0.13	0.06
4583		0.13	-0.88
6654		0.13	-0.02
12299		0.13	-0.27
12376		0.13	1.02
2044		0.13	0.99
32466		0.13	-0.73
9226		0.13	-0.61
5084		0.13	1.06

	17293	0.13	0.89
3436		0.13	0.79
12753		0.13	0.86
4778		0.13	1.04
12496		0.13	-1.02
	16243	0.13	1.48
31812		0.13	0.09
4143		0.13	0.30
4909		0.13	0.02
1981		0.13	0.26
15452		0.13	-0.01
11354		0.12	-0.04
1138		0.12	-0.55
12906		0.12	-0.20
7113		0.12	-0.13
14566		0.12	0.00
15115		0.12	-0.06
9056		0.12	0.38
14285		0.12	-1.27
10591		0.12	-0.91
3753		0.12	1.31
1386		0.12	0.77
30274		0.12	-0.91
15780		0.12	-1.07
14931		0.12	-0.33
12564		0.12	0.34
11577		0.12	0.16
11025		0.12	0.54
15092		0.12	0.37
32667		0.12	0.19
8864		0.12	-0.13
6697		0.12	0.31
9602		0.12	1.02
	9535	0.12	0.97
4991		0.12	-0.42
6592		0.12	0.27
	2528	0.12	-1.17
	9863	0.12	-0.12
9100		0.12	0.31
6508		0.12	-1.07
13055		0.12	0.50
	3687	0.12	-1.08
9697		0.12	-0.23
	20057	0.12	-0.92
31496		0.12	-1.69

11333		0.12	0.03
18145		0.12	0.18
11739		0.12	0.09
11037		0.12	0.17
33188		0.12	-0.06
11247		0.12	0.97
16781		0.12	0.20
31922		0.12	-2.59
33147		0.12	0.68
3618		0.12	1.84
17212		0.12	0.48
10157		0.12	0.11
11318		0.12	-0.12
15739		0.12	-0.99
	17297	0.12	0.22
	10904	0.12	1.06
7131		0.12	0.08
6663		0.12	0.52
30065		0.12	-1.27
7163		0.12	0.43
	12293	0.12	1.34
4108		0.12	-0.44
31195		0.12	-0.28
2875		0.12	0.45
12345		0.12	0.38
32297		0.12	-1.74
30423		0.12	-0.17
5400		0.12	0.46
30483		0.12	0.11
4768		0.12	0.07
3805		0.12	0.25
4733		0.12	0.02
1942		0.12	-0.39
13880		0.12	0.71
17944		0.12	-1.22
	14949	0.12	0.07
12028		0.12	0.21
5470		0.12	-0.01
32852		0.12	0.91
12813		0.12	0.63
33169		0.12	0.28
	8443	0.12	1.14
1756		0.12	-1.13
6167		0.12	1.08
15477		0.12	-0.22

6155		0.11	0.81
	5610	0.11	0.33
6459		0.11	0.73
15860		0.11	0.07
31785		0.11	0.43
17934		0.11	-1.02
5675		0.11	-0.86
32164		0.11	-0.18
30022		0.11	-0.11
8428		0.11	-0.90
	12020	0.11	0.58
31630		0.11	0.50
4258		0.11	-0.46
10254		0.11	0.80
18803		0.11	-1.30
14491		0.11	1.47
1644		0.11	0.59
9606		0.11	-0.22
	15785	0.11	-0.55
5835		0.11	-1.73
7377		0.11	0.00
12235		0.11	-0.40
15546		0.11	0.37
	15808	0.11	-1.75
15352		0.11	-0.13
13969		0.11	-0.56
	3027	0.11	-2.46
12878		0.11	0.97
9752		0.11	0.40
31820		0.11	0.46
2595		0.11	1.49
14740		0.11	0.83
13582		0.11	0.54
17197		0.11	0.28
11937		0.11	-1.79
9173		0.11	0.18
5803		0.11	-0.29
10529		0.11	0.16
9089		0.11	-0.82
1950		0.11	-0.21
13244		0.11	-0.23
32065		0.11	0.66
	9604	0.11	0.09
11985		0.11	-0.39
8078		0.11	1.38

4803		0.11	-0.82
12109		0.11	1.42
3878		0.11	-1.23
11066		0.11	0.43
10916		0.11	0.49
31059		0.11	-1.68
8258		0.11	0.38
2967		0.11	0.59
18594		0.11	0.18
31221		0.11	0.81
	16184	0.11	1.65
5130		0.11	0.03
3620		0.11	2.51
18301		0.11	-0.10
1710		0.11	0.13
8270		0.11	0.54
8243		0.11	2.88
	16240	0.11	0.40
1070		0.11	-0.88
5387		0.11	-1.08
3340		0.11	0.17
18568		0.11	0.89
16989		0.11	-1.54
	18575	0.11	0.01
9992		0.11	-0.82
10338		0.11	-0.03
5036		0.11	-0.69
7516		0.11	-0.82
13622		0.11	0.55
8527		0.11	1.63
8891		0.10	1.57
13439		0.10	0.51
	7434	0.10	1.19
12755		0.10	-0.82
	18792	0.10	-0.01
4767		0.10	0.31
	13594	0.10	0.51
15888		0.10	-0.11
11169		0.10	0.57
7835		0.10	0.11
	9690	0.10	0.56
30005		0.10	0.37
31715		0.10	-1.49
10131		0.10	0.39
1941		0.10	0.00

11419		0.10	-0.34
10126		0.10	-0.55
31842		0.10	-1.70
9254		0.10	1.11
8339		0.10	0.01
6410		0.10	0.18
32336		0.10	-0.13
	14849	0.10	-0.65
5004		0.10	-0.87
15337		0.10	0.30
	5701	0.10	-0.34
32256		0.10	0.06
8191		0.10	-0.96
13410		0.10	0.29
32147		0.10	0.35
12488		0.10	0.40
2210		0.10	-0.48
7002		0.10	0.37
9817		0.10	0.18
32510		0.10	-0.47
17927		0.10	-1.79
9472		0.10	-0.13
1520		0.10	-0.26
5992		0.10	-0.30
32414		0.10	-2.78
8179		0.10	0.05
6504		0.10	-1.57
4822		0.10	0.56
32465		0.10	0.31
	7816	0.10	0.64
15643		0.10	-0.20
8888		0.10	1.49
15624		0.10	-0.21
2256		0.10	-0.51
32634		0.10	0.40
	6240	0.10	-0.13
14065		0.10	0.00
32734		0.10	0.46
15356		0.10	-1.32
5229		0.10	-0.17
14421		0.10	1.27
	429	0.10	-0.95
32859		0.10	-0.74
	18832	0.10	0.31
13585		0.10	-0.59

6171		0.10	0.77
31303		0.10	-0.23
3254		0.10	1.30
9071		0.10	0.66
10918		0.10	-1.29
8085		0.10	0.20
6798		0.10	0.85
13562		0.10	1.10
	13170	0.10	-0.51
13309		0.10	1.73
12096		0.10	-1.01
9543		0.10	-0.31
14489		0.10	-0.33
8286		0.10	0.92
13740		0.10	-0.97
	13362	0.10	0.97
1692		0.09	0.18
7266		0.09	0.37
14493		0.09	0.84
11405		0.09	0.95
6619		0.09	-0.50
4383		0.09	0.62
	10619	0.09	0.14
11127		0.09	-0.49
13021		0.09	-0.02
13160		0.09	-1.20
9295		0.09	0.86
8595		0.09	-0.93
1402		0.09	-0.04
12831		0.09	0.39
33124		0.09	0.14
3631		0.09	0.16
5567		0.09	0.22
	15228	0.09	0.76
6485		0.09	-0.44
1703		0.09	-1.14
7642		0.09	0.15
18516		0.09	0.95
9060		0.09	-0.71
5919		0.09	0.02
16704		0.09	0.41
15029		0.09	-0.09
	7629	0.09	0.81
9500		0.09	-0.66
9461		0.09	0.72

6751		0.09	-0.54
10382		0.09	0.00
11819		0.09	0.40
7807		0.09	0.00
9427		0.09	0.58
	19970	0.09	-1.15
	13314	0.09	0.58
12702		0.09	-0.27
17766		0.09	-1.49
7112		0.09	0.18
	2011	0.09	-0.75
31641		0.09	-0.12
	9485	0.09	0.00
	17746	0.09	0.19
	5448	0.09	1.23
9876		0.09	-1.24
13218		0.09	-0.38
14684		0.09	-0.49
	10520	0.09	0.52
32758		0.09	-0.75
33495		0.09	0.36
30401		0.09	0.66
5572		0.09	0.97
32737		0.09	-0.82
13058		0.09	-0.14
8117		0.09	0.55
	7427	0.09	1.30
32920		0.09	0.67
1789		0.09	1.68
5177		0.09	-0.60
	2694	0.09	0.42
32571		0.09	-0.72
14370		0.09	0.90
4793		0.09	0.61
31051		0.09	1.88
31232		0.09	0.10
7504		0.09	-0.64
14620		0.09	-1.48
2887		0.09	-0.82
13070		0.09	0.16
4952		0.09	-1.19
32835		0.09	-0.06
3302		0.09	-0.92
6947		0.09	-1.48
6510		0.09	1.85

14457		0.09	-0.38
3830		0.09	0.71
	11536	0.08	0.45
8508		0.08	-0.37
2512		0.08	-0.29
15189		0.08	0.02
1233		0.08	-2.03
7074		0.08	0.17
8309		0.08	1.79
12697		0.08	1.43
18105		0.08	-0.34
7300		0.08	0.18
	12326	0.08	-0.91
8586		0.08	0.46
17211		0.08	0.09
10166		0.08	-0.88
6957		0.08	0.49
5632		0.08	0.48
31658		0.08	0.92
4694		0.08	0.91
11508		0.08	0.61
8445		0.08	0.31
3388		0.08	0.59
18473		0.08	-0.83
	7583	0.08	-1.22
9819		0.08	0.27
13095		0.08	-2.30
3568		0.08	-1.21
33054		0.08	0.00
	15759	0.08	-0.45
10505		0.08	-1.25
	11543	0.08	0.41
7917		0.08	-0.94
7700		0.08	-0.79
32632		0.08	0.35
32668		0.08	0.17
9577		0.08	0.41
18584		0.08	-1.70
	17423	0.08	0.36
8138		0.08	-0.55
30417		0.08	-0.71
5945		0.08	-0.78
31061		0.08	-2.21
	15226	0.08	1.40
1317		0.08	0.01

	12378	0.08	1.06
13661		0.08	-1.74
13253		0.08	-0.12
13169		0.08	0.17
8649		0.08	-1.06
31107		0.08	0.25
8567		0.08	0.50
	12388	0.08	0.87
	19359	0.08	-1.56
15254		0.08	0.95
1100		0.08	0.37
14515		0.08	0.94
	11512	0.08	0.40
15563		0.08	-0.15
	4364	0.08	-0.83
5428		0.08	0.40
12517		0.08	-1.87
17270		0.08	0.64
17195		0.08	0.05
30172		0.08	1.22
12477		0.08	0.73
12367		0.08	0.39
10561		0.08	-1.32
32391		0.08	0.22
8345		0.08	-0.24
13305		0.08	-0.97
12311		0.08	-0.15
31438		0.08	-0.86
30362		0.08	-0.25
32483		0.08	-1.12
	16920	0.07	-0.49
7411		0.07	0.36
18428		0.07	-0.73
11209		0.07	0.47
11471		0.07	-1.63
17178		0.07	0.56
	11249	0.07	-1.34
4356		0.07	0.58
32465		0.07	0.55
2791		0.07	0.44
12525		0.07	0.99
11391		0.07	-1.12
14660		0.07	-0.22
5905		0.07	-0.33
5133		0.07	-0.74

31804		0.07	0.24
	9453	0.07	1.91
13957		0.07	-0.37
12363		0.07	-1.39
14612		0.07	1.19
	18269	0.07	0.44
	12978	0.07	-1.73
31450		0.07	0.77
	3074	0.07	-0.34
31523		0.07	-0.46
33093		0.07	0.81
6175		0.07	0.73
13656		0.07	-1.56
	9488	0.07	0.39
9386		0.07	-0.44
9841		0.07	-0.89
32686		0.07	-0.35
33300		0.07	-0.51
13211		0.07	-0.25
31929		0.07	-0.18
	7858	0.07	0.11
33466		0.07	-0.06
32015		0.07	0.31
18112		0.07	-0.36
18536		0.07	-0.01
32639		0.07	-0.08
	19238	0.07	-1.23
	1465	0.07	0.66
3759		0.07	-0.29
8349		0.07	-0.42
17697		0.07	0.37
	6595	0.07	-0.05
7952		0.07	-0.84
	5167	0.07	0.14
8500		0.07	0.16
8298		0.07	-1.82
	1070	0.07	0.81
17639		0.07	-1.60
4880		0.07	-0.04
14993		0.07	0.55
32838		0.07	-0.41
15055		0.07	0.50
7955		0.07	0.06
13627		0.07	0.38
3201		0.07	0.34

15589		0.07	0.13
4716		0.07	-0.07
17818		0.07	0.92
11089		0.07	0.38
31851		0.07	0.13
	14518	0.07	0.12
8434		0.07	-0.99
3509		0.07	0.12
14342		0.07	0.64
	14142	0.07	-0.52
30320		0.07	-0.36
13895		0.07	0.00
8442		0.07	0.34
11801		0.07	0.38
12223		0.07	-0.74
2478		0.07	
30350		0.07	0.79
	10387	0.07	0.01
31017		0.07	0.00
	6398	0.07	-0.18
	2551	0.07	0.39
17237		0.07	0.50
17257		0.07	-0.21
12207		0.07	0.17
2950		0.07	-0.65
4289		0.07	0.47
8953		0.07	-0.27
6420		0.07	-1.22
12759		0.07	0.16
14151		0.07	-1.87
9893		0.07	1.08
17134		0.07	-1.64
5547		0.07	0.22
15269		0.06	-0.97
1381		0.06	0.83
13883		0.06	0.26
2999		0.06	-0.01
4239		0.06	-0.60
	6273	0.06	1.02
10021		0.06	-0.03
14920		0.06	-0.32
10530		0.06	0.66
15771		0.06	1.31
2126		0.06	0.33
33060		0.06	-0.63

30008		0.06	0.01
10966		0.06	-1.67
8784		0.06	1.23
4531		0.06	0.08
31249		0.06	-0.42
32804		0.06	-0.89
11125		0.06	0.77
9517		0.06	-1.24
	20369	0.06	1.76
3022		0.06	-0.66
33075		0.06	-0.95
	16857	0.06	1.51
15066		0.06	0.72
16812		0.06	0.43
8055		0.06	0.79
13912		0.06	0.25
12620		0.06	-0.54
32580		0.06	0.76
9987		0.06	0.11
32163		0.06	0.90
33159		0.06	0.73
7056		0.06	-0.46
	17619	0.06	0.13
	12762	0.06	1.06
13460		0.06	-0.57
8201		0.06	-0.46
8272		0.06	0.78
13558		0.06	-1.55
11177		0.06	-0.10
32538		0.06	-0.09
6460		0.06	0.69
4786		0.06	-0.53
31021		0.06	0.34
4182		0.06	0.40
3407		0.06	-0.54
10578		0.06	-1.21
32644		0.06	0.99
16772		0.06	0.99
14427		0.06	-0.30
6969		0.06	1.62
6892		0.06	-0.06
1812		0.06	1.27
	18326	0.06	-1.47
4345		0.06	-0.51
4989		0.06	-0.09

13759		0.06	-2.05
9811		0.06	0.19
33475		0.06	1.17
11709		0.06	0.89
30147		0.06	0.65
12056		0.06	0.42
	7857	0.06	0.27
13441		0.06	0.70
9284		0.06	-0.48
4845		0.06	-0.12
4646		0.06	-0.36
8228		0.06	-0.48
32854		0.06	0.69
30004		0.06	0.34
1265		0.06	0.86
15274		0.06	-0.78
11226		0.06	0.58
16898		0.06	0.40
3221		0.06	0.63
13748		0.06	0.88
	11597	0.06	-0.26
10581		0.06	0.67
10303		0.05	0.34
4617		0.05	0.21
31530		0.05	-0.88
	17258	0.05	0.75
	2155	0.05	0.54
5644		0.05	0.55
15171		0.05	-0.51
13606		0.05	-1.86
30194		0.05	-0.07
4453		0.05	0.50
1624		0.05	-0.06
15004		0.05	0.35
14692		0.05	-0.03
11637		0.05	-0.74
7121		0.05	0.72
8384		0.05	-0.11
6156		0.05	0.39
3385		0.05	0.49
7525		0.05	-0.72
12672		0.05	0.27
11406		0.05	0.46
12400		0.05	0.18
32246		0.05	-1.75

32090		0.05	-0.24
7598		0.05	-0.55
5571		0.05	-0.73
30108		0.05	-0.19
15348		0.05	-0.12
	16721	0.05	-1.99
31769		0.05	0.74
11490		0.05	0.24
18396		0.05	0.24
11079		0.05	0.44
	6593	0.05	0.23
3166		0.05	-0.52
15541		0.05	0.70
10147		0.05	-0.64
	8564	0.05	1.78
9906		0.05	0.11
3193		0.05	-0.63
6800		0.05	1.56
10996		0.05	-1.08
32476		0.05	0.34
7393		0.05	0.57
11841		0.05	0.65
12677		0.05	-0.02
31373		0.05	0.96
3939		0.05	0.86
32650		0.05	0.69
12713		0.05	0.15
11210		0.05	0.93
12192		0.05	0.34
8604		0.05	0.89
2075		0.05	-0.12
7758		0.05	-0.40
31321		0.05	-0.32
	12310	0.05	-0.12
15408		0.05	-1.03
1330		0.05	1.66
33301		0.05	-0.88
	6900	0.05	-2.03
	13506	0.05	-0.97
12802		0.05	-1.12
18675		0.05	-0.73
14387		0.05	-0.50
15531		0.05	0.87
12436		0.05	0.66
14777		0.05	-0.45

1681		0.05	-1.67
15105		0.05	0.12
10091		0.05	0.10
30379		0.05	0.67
	11248	0.05	-1.24
7459		0.05	0.53
15580		0.05	1.17
11594		0.04	0.13
	2112	0.04	-0.50
13418		0.04	-0.22
9696		0.04	-2.27
4884		0.04	0.86
30147		0.04	0.55
14625		0.04	-1.89
	8435	0.04	0.99
7091		0.04	0.72
5840		0.04	0.12
11093		0.04	1.12
10105		0.04	-0.08
	11652	0.04	0.42
11502		0.04	0.23
8214		0.04	0.18
14079		0.04	0.51
3836		0.04	0.59
6623		0.04	0.40
12320		0.04	0.72
	5946	0.04	-0.43
	14887	0.04	1.00
13285		0.04	-1.06
13580		0.04	-0.52
5111		0.04	-0.17
6050		0.04	-0.94
12108		0.04	0.41
5739		0.04	1.09
14050		0.04	-2.03
2140		0.04	0.30
13005		0.04	0.21
12876		0.04	-1.53
13636		0.04	0.56
12279		0.04	-0.91
9064		0.04	0.88
32203		0.04	0.82
13849		0.04	0.07
	11658	0.04	1.33
17390		0.04	1.01

	5849	0.04	-0.23
	14371	0.04	1.18
8811		0.04	0.58
13652		0.04	0.20
5691		0.04	-1.47
32741		0.04	0.17
9014		0.04	-0.49
	6179	0.04	-1.19
11425		0.04	-1.06
16852		0.04	0.23
3260		0.04	1.03
	2130	0.04	-0.19
3906		0.04	0.52
11369		0.04	-0.63
30424		0.04	-0.14
4546		0.04	0.72
	9939	0.04	-0.22
32178		0.04	1.48
32149		0.04	0.21
	15101	0.04	0.02
17681		0.04	-0.08
8226		0.04	-0.07
32201		0.04	0.86
3740		0.04	-0.29
31362		0.04	1.12
7843		0.04	-0.55
2922		0.04	0.83
	7874	0.04	1.21
8398		0.04	1.05
11279		0.04	-1.14
8068		0.04	-0.08
	13059	0.04	-2.18
3984		0.04	-0.27
8368		0.04	-0.50
13733		0.04	0.40
	13967	0.04	-0.61
15481		0.03	-0.38
31187		0.03	-0.55
	9438	0.03	-1.26
31019		0.03	-1.55
15109		0.03	0.05
5880		0.03	-0.29
32548		0.03	0.15
2854		0.03	-1.10
9664		0.03	-0.06

32652		0.03	-1.13
	13322	0.03	0.35
9940		0.03	-0.12
6642		0.03	0.31
13646		0.03	0.36
	2359	0.03	-2.55
12680		0.03	-1.01
1998		0.03	0.02
	9046	0.03	-1.61
	5286	0.03	-1.00
10923		0.03	-0.38
	1245	0.03	0.80
12589		0.03	-1.42
14538		0.03	0.00
7371		0.03	-0.43
12464		0.03	-0.64
5359		0.03	-0.56
6544		0.03	1.04
7082		0.03	-0.48
2278		0.03	-0.36
6407		0.03	0.29
13789		0.03	0.55
15639		0.03	0.35
2111		0.03	-1.39
7610		0.03	-0.58
32353		0.03	1.28
7033		0.03	-0.42
14369		0.03	0.63
12591		0.03	-0.79
	6289	0.03	-1.22
3036		0.03	0.59
33486		0.03	0.24
17957		0.03	-0.06
9503		0.03	-1.74
31343		0.03	-1.07
15772		0.03	0.47
8411		0.03	0.16
13896		0.03	0.02
33223		0.03	-0.24
10443		0.03	-0.01
12548		0.03	0.59
4726		0.03	-0.24
6341		0.03	-0.33
32170		0.03	0.68
17239		0.03	-0.49

11586		0.03	-0.14
14333		0.03	0.16
1444		0.03	-0.50
1158		0.03	-0.22
6565		0.03	-0.27
33236		0.03	-0.48
2328		0.03	-0.63
5160		0.03	0.61
	3205	0.03	-1.77
9634		0.03	0.64
17387		0.03	0.14
8767		0.03	0.07
	13469	0.03	0.29
5649		0.03	-0.12
32527		0.03	0.16
13367		0.03	-0.03
	7166	0.03	0.76
31159		0.03	-0.42
	7807	0.03	0.45
30392		0.03	-0.67
13877		0.02	0.10
13131		0.02	-0.33
12107		0.02	1.02
9325		0.02	-0.84
15005		0.02	1.36
14721		0.02	0.81
13934		0.02	0.96
	11311	0.02	0.32
11095		0.02	0.91
	15443	0.02	-2.50
12898		0.02	0.86
8419		0.02	-0.91
6562		0.02	-0.01
31018		0.02	0.51
	10988	0.02	0.36
3752		0.02	0.90
10533		0.02	0.75
	19695	0.02	-1.11
5857		0.02	-2.21
8893		0.02	0.66
	20291	0.02	0.68
	11626	0.02	0.68
9476		0.02	-0.01
9257		0.02	0.18
32945		0.02	-0.72

32845		0.02	-0.99
	8939	0.02	1.43
	2762	0.02	-0.35
17193		0.02	1.06
9597		0.02	-1.11
14057		0.02	1.38
9646		0.02	-0.92
8676		0.02	-0.93
15645		0.02	-0.60
32271		0.02	0.88
15277		0.02	0.15
4377		0.02	0.07
12334		0.02	0.95
	18824	0.02	0.05
	8770	0.02	1.11
2200		0.02	0.53
32682		0.02	-2.46
	6405	0.02	-1.38
5700		0.02	0.77
15617		0.02	0.26
	7544	0.02	-1.06
7915		0.02	0.25
15461		0.02	-0.33
7596		0.02	-1.05
7171		0.02	-0.80
13595		0.02	-0.49
	17463	0.02	-0.06
7939		0.02	0.46
7178		0.02	-1.41
17453		0.02	1.08
30191		0.02	0.10
14750		0.02	0.66
2522		0.02	-0.58
30465		0.02	-0.45
14562		0.02	1.06
32047		0.02	-0.23
8807		0.02	0.38
4332		0.02	0.29
8336		0.02	-0.07
6531		0.02	0.17
16970		0.02	1.33
2160		0.02	1.14
	16444	0.02	0.70
11453		0.02	-0.48
	5055	0.02	0.30

	18205	0.02	-1.42
5146		0.02	-0.24
11854		0.02	0.54
12170		0.02	0.96
6914		0.02	-0.04
7109		0.02	0.58
14712		0.02	0.26
4114		0.02	0.54
15295		0.02	0.22
7406		0.02	0.42
6130		0.02	0.68
8333		0.02	-0.89
	2496	0.02	-0.17
	3539	0.02	0.11
7184		0.02	-0.98
9795		0.02	0.93
8464		0.02	1.13
4681		0.01	-0.83
	4917	0.01	0.18
30431		0.01	0.02
	7113	0.01	-1.44
13879		0.01	0.12
15088		0.01	-0.21
	13483	0.01	0.43
	11820	0.01	0.37
	12195	0.01	-0.45
15386		0.01	-1.40
11987		0.01	1.41
7158		0.01	-0.22
10379		0.01	-0.16
3389		0.01	0.71
1640		0.01	0.42
31887		0.01	-0.57
14488		0.01	0.21
9586		0.01	-0.31
	5891	0.01	-2.02
18287		0.01	0.21
7283		0.01	1.85
	9489	0.01	-1.15
15202		0.01	-1.21
3868		0.01	0.03
	14109	0.01	1.40
9734		0.01	1.05
13417		0.01	-2.43
	19889	0.01	0.19

31170		0.01	0.53
9000		0.01	0.16
8198		0.01	-0.87
13919		0.01	-1.44
32792		0.01	1.22
7447		0.01	-0.82
7321		0.01	1.10
30045		0.01	-0.27
13992		0.01	0.53
11992		0.01	2.91
6834		0.01	0.02
12483		0.01	-0.05
15101		0.01	-1.33
17116		0.01	0.29
4582		0.01	-0.83
5952		0.01	0.29
3488		0.01	-1.23
10337		0.01	-0.26
32679		0.01	-0.57
	3522	0.01	-0.76
4841		0.01	-0.87
32397		0.01	0.73
14542		0.01	1.03
4771		0.01	0.99
3294		0.01	-0.25
	18348	0.01	0.52
	2628	0.01	0.02
15727		0.01	-0.90
10924		0.01	-0.99
12644		0.01	-0.80
	13136	0.01	0.05
30366		0.01	-0.26
9601		0.01	1.28
3253		0.01	1.16
3885		0.01	-0.23
31078		0.01	-1.16
2145		0.01	1.14
11206		0.01	0.33
32531		0.01	0.52
11130		0.01	0.15
17757		0.01	0.41
9753		0.01	1.41
8690		0.01	1.63
	7362	0.01	1.51
12404		0.01	0.28

6180		0.01	1.01
31279		0.01	-0.75
	9606	0.01	-0.91
14811		0.01	0.31
11783		0.01	1.05
5731		0.01	0.41
6345		0.01	0.52
31548		0.01	-0.85
10267		0.01	0.35
	5416	0.01	0.70
7292		0.01	0.01
17245		0.01	-2.46
7380		0.01	0.84
14102		0.01	0.31
17046		0.01	0.24
	14431	0.01	0.21
1294		0.01	-1.06
	2779	0.01	-0.43
10583		0.01	-0.04
10465		0.01	0.46
3645		0.01	-0.60
15077		0.00	0.73
4484		0.00	0.15
3415		0.00	0.39
15594		0.00	1.22
13293		0.00	0.33
18177		0.00	-0.80
6028		0.00	3.39
32794		0.00	-0.27
12935		0.00	0.32
32655		0.00	-1.19
6662		0.00	0.51
10418		0.00	-0.15
15650		0.00	0.30
15435		0.00	0.26
1172		0.00	-0.69
16727		0.00	0.25
16905		0.00	1.30
7072		0.00	-0.09
7170		0.00	0.06
14482		0.00	-0.96
2125		0.00	0.01
18087		0.00	-1.71
32450		0.00	-2.08
12849		0.00	-0.52

15147		0.00	0.24
15781		0.00	-0.53
8421		0.00	0.07
11912		0.00	0.27
32160		0.00	-1.29
	2164	0.00	0.07
32382		0.00	-0.54
6553		0.00	-1.12
4342		0.00	1.79
7448		0.00	-0.04
13444		0.00	-0.22
3652		0.00	-0.04
4297		0.00	0.97
2559		0.00	-0.38
	16825	0.00	1.38
14694		0.00	-0.16
3760		0.00	0.33
2171		0.00	0.71
12921		0.00	-1.97
9514		0.00	-0.95
10265		0.00	-0.40
1131		0.00	-0.08
	18299	0.00	1.42
11994		0.00	-0.20
3515		0.00	-1.01
17977		0.00	1.06
9470		0.00	-0.67
9538		0.00	0.05
17658		0.00	0.88
	19553	0.00	-0.96
11561		0.00	-0.18
14127		0.00	-1.08
10077		0.00	-0.60
13737		0.00	1.50
	6246	0.00	-0.51
1014		0.00	0.04
31092		0.00	-0.62
9590		0.00	0.52
	5326	0.00	-0.66
30291		0.00	0.26
3629		0.00	-0.69
3640		0.00	-1.39
4058		0.00	0.07
4307		0.00	-1.75
4409		0.00	0.07

4821		0.00	-0.20
5009		0.00	0.62
5354		0.00	-0.52
5505		0.00	0.48
5566		0.00	-1.04
5583		0.00	0.49
6218		0.00	-0.76
6865		0.00	-0.50
8647		0.00	-0.28
9077		0.00	-0.25
9123		0.00	0.34
9379		0.00	-1.08
9633		0.00	-0.30
10011		0.00	0.24
11822		0.00	-0.25
12375		0.00	0.03
12783		0.00	0.84
13771		0.00	0.34
14296		0.00	0.42
15284		0.00	0.25
15472		0.00	-0.23
15538		0.00	0.82
15782		0.00	-1.18
15874		0.00	-1.91
16789		0.00	1.11
17028		0.00	-0.93
31009		0.00	-0.01
31481		0.00	0.62
31628		0.00	0.50
31907		0.00	-0.45
32177		0.00	-1.00
	1523	0.00	-0.66
	4493	0.00	0.34
	4644	0.00	-0.71
	8894	0.00	1.17
	14088	0.00	-0.72
	15392	0.00	0.89
	19718	0.00	-0.03
	20052	0.00	-1.73
3208		0.00	0.69
	5297	0.00	-1.22
9930		0.00	-0.62
6831		0.00	0.19
13596		0.00	-0.47
	6529	0.00	-1.16

17300		0.00	0.49
10194		0.00	0.66
11403		0.00	-0.69
12724		0.00	0.30
11977		0.00	0.09
13954		0.00	-0.79
31764		0.00	-0.15
11990		0.00	1.28
	17963	0.00	0.72
1065		0.00	0.31
30066		0.00	-0.01
2129		0.00	-1.58
8782		0.00	0.75
14887		0.00	0.67
	17124	0.00	0.10
13689		0.00	-1.09
3139		0.00	-0.42
7936		0.00	0.48
7487		0.00	-0.48
17820		0.00	-1.59
14483		0.00	-0.46
6453		0.00	0.57
7815		0.00	0.08
8541		0.00	0.19
	4260	0.00	-1.10
18495		0.00	1.07
15325		0.00	-0.63
11459		0.00	0.39
31801		0.00	-0.45
18609		0.00	0.07
14561		0.00	-0.40
6272		0.00	0.92
9258		0.00	-0.05
18275		0.00	-0.32
	17621	0.00	0.11
7204		0.00	-0.54
5338		0.00	0.67
13313		0.00	0.76
13891		0.00	0.80
6658		0.00	-0.29
17646		0.00	-0.61
2267		0.00	0.00
15398		0.00	0.02
15227		0.00	-1.05
10545		0.00	-0.51

31465		0.00	-0.46
15296		0.00	-0.85
30062		0.00	0.70
6016		0.00	2.07
18262		0.00	-1.39
13320		0.00	1.02
15180		0.00	0.74
3428		0.00	0.40
33484		0.00	-0.57
4559		0.00	0.01
15288		0.00	0.27
8948		-0.01	0.05
10672		-0.01	-1.79
12846		-0.01	0.54
3613		-0.01	1.25
9460		-0.01	0.52
4977		-0.01	0.34
32208		-0.01	-0.18
31642		-0.01	-0.35
7212		-0.01	0.62
11650		-0.01	0.81
13046		-0.01	1.18
	2131	-0.01	0.14
10296		-0.01	0.00
2938		-0.01	0.46
6752		-0.01	-0.89
6299		-0.01	-0.08
5821		-0.01	0.35
2650		-0.01	-2.70
	9788	-0.01	-0.75
5418		-0.01	0.26
8927		-0.01	-0.33
10655		-0.01	-1.18
15266		-0.01	-0.18
5730		-0.01	2.65
18349		-0.01	0.53
	7069	-0.01	-0.53
9238		-0.01	0.14
15768		-0.01	-0.44
9843		-0.01	-0.54
9390		-0.01	-1.37
11061		-0.01	-0.29
31634		-0.01	-0.83
15914		-0.01	0.46
14559		-0.01	0.08

3943		-0.01	0.30
1641		-0.01	-0.23
4279		-0.01	1.14
	14665	-0.01	-0.94
2239		-0.01	0.20
14514		-0.01	0.07
4979		-0.01	1.91
	7144	-0.01	-3.37
31832		-0.01	0.05
1636		-0.01	1.64
10908		-0.01	-0.23
32100		-0.01	-0.31
	2746	-0.01	0.85
5222		-0.01	-0.30
6219		-0.01	-2.30
13962		-0.01	-0.31
4574		-0.01	-1.51
5676		-0.01	0.02
17669		-0.01	-0.15
13243		-0.01	-0.72
6622		-0.01	0.46
14713		-0.01	0.41
6801		-0.01	-1.00
12659		-0.01	0.80
9486		-0.01	0.61
33085		-0.01	-1.19
16971		-0.01	0.27
12391		-0.01	-1.79
10120		-0.01	0.03
18039		-0.01	-0.23
4734		-0.01	-2.07
14007		-0.01	-0.36
6628		-0.01	-1.82
12443		-0.01	0.45
10847		-0.01	0.39
31802		-0.01	0.08
32304		-0.01	-1.00
	18498	-0.01	-0.73
8896		-0.01	0.22
31103		-0.01	-0.98
	7126	-0.01	1.25
	15396	-0.01	-0.24
15505		-0.01	0.61
32301		-0.01	-0.12
17176		-0.01	1.21

10041		-0.01	-0.27
14534		-0.01	-0.14
18608		-0.01	-1.47
9660		-0.01	-0.50
30502		-0.01	0.12
	6249	-0.01	0.28
14710		-0.01	0.45
31559		-0.01	0.06
13297		-0.01	0.59
14717		-0.01	0.23
	12998	-0.01	-1.32
	13173	-0.01	0.32
	18569	-0.01	0.68
5357		-0.01	-0.62
31911		-0.01	-1.00
3330		-0.01	-1.54
4538		-0.01	-0.97
15106		-0.01	0.51
13998		-0.01	-0.82
8303		-0.01	0.62
8519		-0.02	-0.12
9994		-0.02	0.86
32152		-0.02	-0.88
31554		-0.02	-0.95
7990		-0.02	-0.84
5788		-0.02	2.67
8396		-0.02	-0.02
4334		-0.02	0.88
12186		-0.02	-0.83
	2620	-0.02	-1.20
12194		-0.02	0.77
33091		-0.02	-1.20
5112		-0.02	0.35
	16465	-0.02	-0.15
33183		-0.02	0.38
18250		-0.02	-0.22
4607		-0.02	-1.26
9669		-0.02	0.23
33308		-0.02	-0.10
7968		-0.02	-0.90
5075		-0.02	0.24
31639		-0.02	-0.78
	4948	-0.02	-0.03
4477		-0.02	0.73
10270		-0.02	-1.21

2678		-0.02	-0.79
18362		-0.02	0.66
	5585	-0.02	-0.94
30188		-0.02	-0.30
31022		-0.02	-0.34
17331		-0.02	0.48
	7351	-0.02	0.92
11943		-0.02	-0.42
7130		-0.02	-0.28
7935		-0.02	-0.79
	18627	-0.02	-0.30
	750	-0.02	-1.42
18598		-0.02	0.14
32597		-0.02	0.13
4511		-0.02	-0.20
	367	-0.02	1.16
8648		-0.02	-0.46
2708		-0.02	0.17
7176		-0.02	-0.78
13803		-0.02	0.38
6174		-0.02	-0.47
7870		-0.02	-1.26
10215		-0.02	-0.39
3344		-0.02	-0.48
	9246	-0.02	-1.27
3505		-0.02	-1.25
	9729	-0.02	0.18
9628		-0.02	-1.02
10339		-0.02	-1.26
13000		-0.02	0.23
7122		-0.02	1.01
12229		-0.02	0.67
32678		-0.02	-0.12
14943		-0.02	-0.45
18358		-0.02	0.81
11186		-0.02	-0.12
	11856	-0.02	-0.27
12142		-0.02	0.33
	20366	-0.02	1.82
33461		-0.02	0.54
2096		-0.02	0.05
32452		-0.02	0.77
12286		-0.02	-2.13
13349		-0.02	0.65
4494		-0.02	-0.01

8632		-0.02	-0.55
3734		-0.02	0.28
13215		-0.02	0.91
5232		-0.02	-0.63
	13341	-0.02	0.41
	11246	-0.02	-1.34
14032		-0.02	0.92
16783		-0.02	-0.01
5063		-0.02	0.10
18548		-0.02	-1.53
17571		-0.02	-0.96
31824		-0.02	-1.40
15021		-0.02	0.17
9951		-0.02	-0.10
13059		-0.03	-1.71
9961		-0.03	0.70
	7667	-0.03	0.52
15704		-0.03	0.04
3052		-0.03	-1.35
15479		-0.03	-0.31
10663		-0.03	0.39
1311		-0.03	-0.59
4198		-0.03	0.65
14132		-0.03	-1.47
14799		-0.03	0.47
11534		-0.03	0.64
	10707	-0.03	0.08
1218		-0.03	-0.28
18647		-0.03	-1.44
18321		-0.03	-0.36
30077		-0.03	0.49
	17287	-0.03	0.23
7405		-0.03	0.10
	1137	-0.03	1.29
4061		-0.03	-0.71
9279		-0.03	0.23
	19797	-0.03	0.35
	1962	-0.03	-0.40
32433		-0.03	-0.87
33173		-0.03	-1.73
32970		-0.03	-1.02
32395		-0.03	0.88
17600		-0.03	-0.04
5787		-0.03	-0.28
15555		-0.03	0.88

8451		-0.03	-0.96
15524		-0.03	0.48
1102		-0.03	0.77
11598		-0.03	0.96
31509		-0.03	0.36
9806		-0.03	0.11
11668		-0.03	-0.31
2191		-0.03	1.99
	11845	-0.03	-1.27
	13980	-0.03	-1.62
3028		-0.03	1.14
30468		-0.03	-2.17
5656		-0.03	-0.08
	9854	-0.03	1.24
8599		-0.03	0.01
	12427	-0.03	-1.06
6984		-0.03	-1.06
10017		-0.03	0.14
7632		-0.03	0.08
14716		-0.03	0.66
	20326	-0.03	0.04
31467		-0.03	-0.15
10997		-0.03	-0.39
5005		-0.03	-0.23
12938		-0.03	0.68
6084		-0.03	-0.92
6600		-0.03	0.13
31370		-0.03	-0.58
1056		-0.03	-0.51
11386		-0.03	1.19
	14955	-0.03	-0.45
31515		-0.03	0.26
7438		-0.03	-0.13
9746		-0.03	1.83
4260		-0.03	0.14
30118		-0.03	-0.29
12019		-0.03	-0.03
4869		-0.03	-0.79
16757		-0.03	0.99
	7382	-0.03	1.59
18031		-0.03	0.10
	11204	-0.03	-0.55
1886		-0.03	0.00
12227		-0.03	-0.06
12951		-0.03	-0.07

14250		-0.03	-0.74
	10295	-0.03	-1.49
32697		-0.03	0.84
9347		-0.03	-0.53
	19877	-0.03	1.40
3359		-0.03	-0.04
1470		-0.03	0.61
18103		-0.03	-0.08
15925		-0.03	-0.62
	2636	-0.03	0.39
	6949	-0.03	-0.20
5432		-0.03	1.04
4898		-0.03	-0.14
	10334	-0.03	-0.61
12432		-0.04	0.05
	2702	-0.04	0.24
14095		-0.04	-0.43
8353		-0.04	-0.62
9781		-0.04	0.73
11248		-0.04	0.46
11162		-0.04	-0.54
33347		-0.04	0.46
	3386	-0.04	-0.53
13248		-0.04	-1.46
3231		-0.04	0.46
8340		-0.04	-0.55
32439		-0.04	1.47
17148		-0.04	0.42
15237		-0.04	0.34
	14375	-0.04	1.63
9091		-0.04	0.09
	9652	-0.04	0.77
9360		-0.04	2.43
2204		-0.04	-0.65
1308		-0.04	-0.04
10309		-0.04	0.10
	2651	-0.04	-0.97
7979		-0.04	0.48
17036		-0.04	0.17
12743		-0.04	0.65
3437		-0.04	0.56
32462		-0.04	-1.33
4781		-0.04	0.95
1241		-0.04	1.06
6204		-0.04	-0.95

	17443	-0.04	0.07
3121		-0.04	0.15
9094		-0.04	-0.24
32659		-0.04	-0.65
11368		-0.04	0.28
3479		-0.04	-1.45
17344		-0.04	0.00
6005		-0.04	0.34
5917		-0.04	0.23
10272		-0.04	-0.43
5854		-0.04	-1.54
6192		-0.04	-1.28
15306		-0.04	-0.19
10653		-0.04	0.82
30418		-0.04	0.10
13787		-0.04	0.20
4115		-0.04	-0.49
11784		-0.04	0.56
32443		-0.04	-0.24
31280		-0.04	-0.10
15549		-0.04	-0.42
13477		-0.04	-0.78
4437		-0.04	-0.25
3687		-0.04	0.10
14034		-0.04	1.24
31033		-0.04	-0.45
9975		-0.04	-1.27
10161		-0.04	-0.08
3925		-0.04	1.11
10324		-0.04	-0.18
10335		-0.04	-0.85
11880		-0.04	-0.88
7778		-0.04	0.91
31695		-0.04	-1.01
3790		-0.04	0.30
7034		-0.04	0.59
8944		-0.04	-0.83
3942		-0.04	1.67
6965		-0.04	0.86
5722		-0.05	0.43
17152		-0.05	0.42
10700		-0.05	0.87
3739		-0.05	-0.95
11641		-0.05	0.02
15776		-0.05	0.53

18660		-0.05	0.01
9383		-0.05	-0.03
5473		-0.05	-0.28
9554		-0.05	-0.25
1814		-0.05	0.81
6716		-0.05	0.06
9181		-0.05	-1.22
6610		-0.05	-1.63
31880		-0.05	-2.99
18745		-0.05	0.05
31741		-0.05	-3.92
12623		-0.05	-0.04
16940		-0.05	0.39
13073		-0.05	0.06
2310		-0.05	-0.01
	4600	-0.05	0.69
15023		-0.05	-0.49
10178		-0.05	0.46
8795		-0.05	1.70
10638		-0.05	0.44
14609		-0.05	-0.05
14497		-0.05	0.54
11221		-0.05	-0.13
17544		-0.05	0.19
9792		-0.05	0.21
9888		-0.05	-1.00
2336		-0.05	-0.88
5196		-0.05	-0.30
	12682	-0.05	-0.11
	1519	-0.05	0.39
1168		-0.05	-0.51
30090		-0.05	-1.32
9542		-0.05	0.87
4584		-0.05	0.40
	8877	-0.05	-1.43
5662		-0.05	0.32
1724		-0.05	0.34
8468		-0.05	-0.57
3485		-0.05	-0.94
1372		-0.05	0.97
8397		-0.05	0.22
33489		-0.05	-0.09
1738		-0.05	-0.89
	2544	-0.05	-0.07
1017		-0.05	0.48

6308		-0.05	-0.12
	6480	-0.05	0.82
9801		-0.05	0.25
6375		-0.05	-0.80
31619		-0.05	-1.11
17290		-0.05	-1.38
13316		-0.05	-1.07
31609		-0.05	-0.27
7094		-0.05	0.27
31783		-0.05	-0.44
12877		-0.05	0.17
33298		-0.05	-1.78
1605		-0.05	0.66
3219		-0.05	0.13
4084		-0.05	-0.27
14907		-0.05	-1.31
31200		-0.05	-0.10
33101		-0.05	-0.17
32320		-0.05	-0.12
10340		-0.05	-0.05
16947		-0.05	-2.32
31953		-0.06	-0.93
6475		-0.06	-0.09
17368		-0.06	0.59
4598		-0.06	-1.03
6007		-0.06	-0.27
	2924	-0.06	1.62
9492		-0.06	-1.58
31533		-0.06	-1.85
9373		-0.06	-0.92
11671		-0.06	0.38
9640		-0.06	0.26
3832		-0.06	-0.48
31495		-0.06	-0.14
13950		-0.06	0.85
6013		-0.06	3.17
15919		-0.06	-0.23
1907		-0.06	-0.25
14583		-0.06	-0.28
33114		-0.06	-0.91
15601		-0.06	-0.75
9206		-0.06	-0.79
9102		-0.06	0.27
18641		-0.06	-1.29
	7513	-0.06	-1.89

11278		-0.06	0.25
14731		-0.06	-1.13
9894		-0.06	0.30
11063		-0.06	0.39
16926		-0.06	-0.72
	9886	-0.06	0.17
11350		-0.06	1.03
	3049	-0.06	-1.48
	3831	-0.06	-1.80
3894		-0.06	-0.48
10119		-0.06	-0.17
12806		-0.06	-0.45
6310		-0.06	0.45
4995		-0.06	-0.05
1161		-0.06	-0.49
11915		-0.06	1.59
15918		-0.06	-0.50
10053		-0.06	-0.74
2047		-0.06	0.57
32862		-0.06	0.61
15212		-0.06	-0.56
15701		-0.06	-0.67
3964		-0.06	0.25
	19174	-0.06	-0.79
1544		-0.06	0.93
30015		-0.06	0.63
	5627	-0.06	1.02
8833		-0.06	-0.47
7638		-0.06	-1.79
17004		-0.06	-0.02
4110		-0.06	0.30
17323		-0.06	-0.44
32987		-0.06	0.93
7020		-0.06	-1.35
8443		-0.06	0.85
4863		-0.06	-1.08
10754		-0.06	-0.68
	19722	-0.06	0.29
8031		-0.06	0.41
11085		-0.06	0.33
33254		-0.06	1.10
	19543	-0.06	0.57
4205		-0.06	-0.08
13392		-0.06	-0.16
2213		-0.06	-0.31

10814		-0.06	-0.19
5590		-0.06	0.20
13727		-0.06	1.09
32645		-0.06	0.03
17943		-0.06	-0.51
9434		-0.06	1.24
3401		-0.06	-0.72
10090		-0.06	0.55
9495		-0.06	-0.45
31862		-0.06	-0.50
4398		-0.07	-0.84
5518		-0.07	0.52
	7424	-0.07	-0.90
	19072	-0.07	0.19
5925		-0.07	-0.16
12576		-0.07	0.06
32238		-0.07	0.57
10108		-0.07	-0.36
6549		-0.07	0.33
9809		-0.07	0.71
6815		-0.07	-1.85
	7640	-0.07	-0.99
	9502	-0.07	1.05
8907		-0.07	-1.20
12781		-0.07	1.15
17105		-0.07	0.20
4547		-0.07	0.63
	3202	-0.07	-0.14
13081		-0.07	-0.84
	12869	-0.07	0.38
9140		-0.07	-0.45
9773		-0.07	0.69
9080		-0.07	1.23
	2531	-0.07	-0.88
4282		-0.07	-0.81
8773		-0.07	-0.45
1417		-0.07	-0.23
3271		-0.07	0.24
4186		-0.07	-0.10
4713		-0.07	-0.93
30367		-0.07	1.46
5869		-0.07	-0.08
5949		-0.07	0.41
8248		-0.07	-0.13
18654		-0.07	0.29

	13036	-0.07	-2.04
5855		-0.07	-0.22
16953		-0.07	0.51
7771		-0.07	-0.15
16799		-0.07	-0.60
	18565	-0.07	1.00
6312		-0.07	0.43
2207		-0.07	-0.13
32227		-0.07	-1.05
	12508	-0.07	1.63
33181		-0.07	0.60
32514		-0.07	-0.66
15087		-0.07	0.26
4725		-0.07	-1.37
3597		-0.07	2.10
	18305	-0.07	0.38
5395		-0.07	-0.37
14490		-0.07	1.59
9571		-0.07	0.32
	18185	-0.07	0.01
11600		-0.07	1.00
7902		-0.07	0.64
3009		-0.07	-0.43
	2663	-0.07	-0.19
30488		-0.07	-1.88
12865		-0.07	0.65
6728		-0.07	0.36
	907	-0.07	-0.12
	15213	-0.07	0.86
13907		-0.07	0.73
1339		-0.07	-0.50
11770		-0.07	-0.72
	7671	-0.07	-2.18
7322		-0.07	-0.01
3584		-0.07	0.77
	15802	-0.08	-1.14
5646		-0.08	0.69
9881		-0.08	-0.80
	1261	-0.08	0.13
8511		-0.08	-1.48
	12757	-0.08	0.04
8453		-0.08	-1.68
33162		-0.08	0.02
	5805	-0.08	-0.38
	6647	-0.08	-0.01

3879		-0.08	-1.11
31823		-0.08	-0.44
32131		-0.08	-0.04
15862		-0.08	-1.07
14881		-0.08	0.20
5688		-0.08	1.18
14605		-0.08	0.11
6656		-0.08	-0.66
	19566	-0.08	-0.46
31272		-0.08	-1.13
14502		-0.08	0.32
4161		-0.08	0.59
9609		-0.08	-1.28
12626		-0.08	-0.63
14767		-0.08	0.39
11015		-0.08	-0.02
4683		-0.08	0.23
7579		-0.08	0.11
	12037	-0.08	-1.92
	9393	-0.08	-1.04
7230		-0.08	0.04
13163		-0.08	0.68
1504		-0.08	-0.38
31751		-0.08	-0.88
3000		-0.08	1.01
8107		-0.08	1.25
15313		-0.08	-0.15
32030		-0.08	0.90
8870		-0.08	0.80
5335		-0.08	0.55
5231		-0.08	-0.49
32529		-0.08	0.14
16785		-0.08	-0.12
32505		-0.08	-0.31
16815		-0.08	1.56
31788		-0.08	-0.07
10899		-0.08	-0.32
9826		-0.08	-0.38
10542		-0.08	-1.29
31361		-0.08	-0.30
8271		-0.08	0.50
5157		-0.08	0.36
11873		-0.08	0.79
6376		-0.08	-1.29
	4731	-0.08	1.61

15405		-0.08	1.17
	19720	-0.08	0.29
	17204	-0.08	0.30
4089		-0.08	-0.40
6157		-0.08	-0.51
9247		-0.08	0.83
4471		-0.08	-0.03
4434		-0.08	0.31
10756		-0.08	0.96
1894		-0.08	-1.09
15282		-0.08	-0.05
6025		-0.08	0.50
14200		-0.08	-1.55
10566		-0.08	-1.06
14141		-0.08	-0.50
	6017	-0.08	0.59
9239		-0.08	0.06
31350		-0.08	-0.83
2125		-0.08	-0.20
10055		-0.08	0.19
31454		-0.08	0.32
14548		-0.08	-0.06
15547		-0.08	0.59
13717		-0.08	-0.83
13916		-0.08	-0.45
	17530	-0.08	-0.21
9325		-0.08	-0.31
7673		-0.08	1.09
9617		-0.09	0.50
11858		-0.09	0.20
6151		-0.09	-1.50
12800		-0.09	0.79
1670		-0.09	-0.57
1449		-0.09	0.38
14675		-0.09	-1.36
10245		-0.09	-0.42
14804		-0.09	-0.75
5723		-0.09	0.31
14549		-0.09	0.21
	9391	-0.09	-2.33
31313		-0.09	0.28
6875		-0.09	-0.03
32314		-0.09	-1.33
8721		-0.09	-0.03
31720		-0.09	-0.01

32181		-0.09	-0.18
13426		-0.09	-0.59
5472		-0.09	-0.48
13897		-0.09	0.27
13434		-0.09	0.32
	3900	-0.09	0.39
7590		-0.09	0.30
4452		-0.09	0.08
	7503	-0.09	-1.96
	5822	-0.09	-1.17
13053		-0.09	0.65
4053		-0.09	-0.31
32506		-0.09	0.26
4799		-0.09	0.47
31796		-0.09	0.61
11909		-0.09	0.18
	3653	-0.09	0.50
3078		-0.09	-0.42
30498		-0.09	0.08
6448		-0.09	-0.51
8627		-0.09	0.45
16790		-0.09	0.94
	11674	-0.09	0.42
10170		-0.09	0.40
	16561	-0.09	-0.01
17917		-0.09	-1.77
6934		-0.09	1.24
18081		-0.09	-0.39
13512		-0.09	0.25
8344		-0.09	-0.85
	17712	-0.09	-0.94
	7436	-0.09	0.90
9111		-0.09	0.06
	11519	-0.09	-1.01
3819		-0.09	-1.12
12379		-0.09	-0.91
32176		-0.09	0.89
7730		-0.09	1.12
	2662	-0.09	0.87
4751		-0.09	0.70
32431		-0.09	-1.27
6827		-0.09	1.83
13739		-0.09	-2.76
17996		-0.09	-0.15
6661		-0.09	0.09

32422		-0.09	-0.97
	16732	-0.09	0.36
14565		-0.09	-0.72
32940		-0.09	-0.19
4925		-0.09	0.18
11401		-0.09	-0.68
9531		-0.09	0.23
4281		-0.09	0.55
	11198	-0.09	-0.50
3861		-0.09	-2.15
33143		-0.09	0.56
13953		-0.09	0.44
13611		-0.09	-2.00
11988		-0.09	0.31
	15351	-0.09	-0.29
31938		-0.09	-2.01
3322		-0.09	-0.43
12139		-0.09	-0.92
17530		-0.09	-0.06
10824		-0.09	0.22
1221		-0.09	-0.19
3356		-0.10	-0.13
12678		-0.10	-0.50
13694		-0.10	-0.60
6378		-0.10	-1.17
	5886	-0.10	0.36
9614		-0.10	0.10
14815		-0.10	0.86
	647	-0.10	-1.24
32375		-0.10	-1.98
1424		-0.10	-0.84
	18667	-0.10	-0.77
8668		-0.10	-0.41
32242		-0.10	0.78
4730		-0.10	0.10
4957		-0.10	0.22
8379		-0.10	0.52
15743		-0.10	0.29
4426		-0.10	-2.37
18679		-0.10	0.01
13480		-0.10	-2.12
7589		-0.10	0.33
3835		-0.10	-1.56
8173		-0.10	-1.53
10061		-0.10	-1.20

17200		-0.10	-0.19
5027		-0.10	-0.15
	4142	-0.10	-0.91
17795		-0.10	-1.96
4581		-0.10	-0.89
13890		-0.10	-0.11
16844		-0.10	0.60
	114	-0.10	0.76
8861		-0.10	0.61
9587		-0.10	-0.04
8936		-0.10	0.81
32611		-0.10	-0.19
18348		-0.10	-0.95
14693		-0.10	-0.31
	13408	-0.10	0.71
13074		-0.10	-1.71
	4210	-0.10	0.90
	3185	-0.10	-0.01
12351		-0.10	0.96
30339		-0.10	-0.87
11512		-0.10	0.05
16813		-0.10	1.59
9875		-0.10	-1.78
12844		-0.10	0.78
18106		-0.10	0.66
12023		-0.10	-0.37
5203		-0.10	-0.71
31729		-0.10	-0.22
	19782	-0.10	0.38
7563		-0.10	-0.01
13002		-0.10	1.47
4887		-0.10	1.76
31644		-0.10	-0.39
31233		-0.10	-0.06
	19942	-0.10	0.94
12897		-0.10	0.82
7457		-0.10	0.29
5485		-0.10	1.08
12864		-0.10	0.10
18026		-0.10	0.25
10962		-0.10	-1.34
32542		-0.10	-0.77
1552		-0.10	-0.07
9681		-0.10	0.13
3163		-0.10	0.42

3399		-0.10	0.26
9695		-0.10	-0.38
	1188	-0.10	0.94
33248		-0.10	0.16
32182		-0.10	-0.55
4008		-0.10	-1.19
15275		-0.10	-1.39
7565		-0.10	-0.23
32399		-0.10	0.19
3178		-0.10	0.02
5171		-0.10	-0.08
4287		-0.10	-0.04
32080		-0.10	1.03
7433		-0.10	-0.60
	13352	-0.10	-0.55
	4903	-0.10	0.92
32341		-0.10	0.25
13822		-0.11	-0.02
1806		-0.11	0.06
31819		-0.11	-0.84
	3496	-0.11	1.25
31511		-0.11	1.39
10176		-0.11	0.27
11576		-0.11	0.58
10014		-0.11	-0.55
	218	-0.11	-0.10
	6896	-0.11	-1.92
	16978	-0.11	-1.03
32664		-0.11	0.74
	2682	-0.11	1.15
11882		-0.11	-0.34
31699		-0.11	-1.41
17907		-0.11	-1.56
30106		-0.11	1.26
2952		-0.11	0.56
33342		-0.11	-0.98
	1643	-0.11	0.09
18094		-0.11	0.05
11158		-0.11	-1.36
	7468	-0.11	0.91
17592		-0.11	-1.85
16936		-0.11	0.56
7422		-0.11	0.62
9063		-0.11	0.08
2152		-0.11	-0.17

1099		-0.11	0.00
7712		-0.11	0.94
5989		-0.11	0.01
5824		-0.11	0.12
33087		-0.11	-0.63
	6343	-0.11	1.36
10232		-0.11	-0.43
6755		-0.11	0.31
9765		-0.11	0.20
11440		-0.11	-0.27
30174		-0.11	0.50
7420		-0.11	0.34
9896		-0.11	-0.13
31179		-0.11	-0.74
2794		-0.11	-0.29
32613		-0.11	0.75
	6308	-0.11	0.47
32300		-0.11	0.32
14218		-0.11	0.15
10953		-0.11	-0.06
10032		-0.11	-0.51
	1229	-0.11	2.02
32379		-0.11	0.52
14739		-0.11	1.46
	14455	-0.11	0.10
	20142	-0.11	-0.92
18088		-0.11	0.02
17437		-0.11	-0.47
6972		-0.11	-1.52
13024		-0.11	0.81
18130		-0.11	-0.02
33478		-0.11	0.38
5814		-0.11	-0.29
30151		-0.11	0.35
1708		-0.11	0.62
32372		-0.11	-0.99
10431		-0.11	0.43
14121		-0.11	0.00
	7838	-0.11	0.88
18067		-0.11	-0.02
11660		-0.11	-0.30
33045		-0.11	-0.03
17119		-0.11	0.74
	11670	-0.11	1.46
7263		-0.11	1.03

6189		-0.11	-2.32
11738		-0.11	0.58
4088		-0.11	0.03
30395		-0.11	-0.40
13590		-0.11	0.27
5186		-0.11	-0.58
	1691	-0.12	0.81
	11685	-0.12	0.66
6008		-0.12	-1.53
7937		-0.12	-1.38
32403		-0.12	-1.49
14020		-0.12	-1.63
	9603	-0.12	0.62
11165		-0.12	-0.34
14797		-0.12	-1.38
13791		-0.12	0.22
32950		-0.12	0.56
16734		-0.12	0.90
5374		-0.12	-0.24
11611		-0.12	-0.42
33320		-0.12	0.10
7168		-0.12	-0.06
17477		-0.12	0.26
	4199	-0.12	-0.01
12481		-0.12	-0.67
13632		-0.12	1.03
	443	-0.12	-1.39
	14493	-0.12	0.13
10366		-0.12	-1.30
31115		-0.12	-0.62
14157		-0.12	0.07
14708		-0.12	0.08
10252		-0.12	-0.21
10877		-0.12	-0.48
6643		-0.12	0.56
17746		-0.12	1.59
32381		-0.12	0.11
18522		-0.12	-0.26
4491		-0.12	1.33
12182		-0.12	0.45
32555		-0.12	0.06
4103		-0.12	-0.42
18004		-0.12	1.78
5198		-0.12	0.17
	12885	-0.12	0.68

31835		-0.12	-0.61
32244		-0.12	0.20
33280		-0.12	1.68
7922		-0.12	0.70
	11240	-0.12	1.81
32334		-0.12	-0.21
2224		-0.12	1.02
	12580	-0.12	0.20
30470		-0.12	-1.34
14698		-0.12	-0.07
	19247	-0.12	-0.61
3989		-0.12	-0.94
5439		-0.12	-1.11
18214		-0.12	-0.21
13084		-0.12	-1.88
9066		-0.12	-0.33
11420		-0.12	1.22
32245		-0.12	0.33
12205		-0.12	-0.39
10089		-0.12	-1.22
	4026	-0.12	-1.58
	9719	-0.12	0.09
10101		-0.12	-1.05
	5148	-0.12	0.34
8980		-0.12	-1.68
33349		-0.12	-0.15
4371		-0.12	-0.24
9667		-0.12	-0.94
	7112	-0.12	-1.73
7456		-0.12	0.88
8930		-0.12	-0.66
8886		-0.12	-0.21
	5603	-0.12	0.39
	4652	-0.12	0.15
31703		-0.12	-0.34
3748		-0.12	-0.25
11084		-0.12	0.24
17360		-0.12	0.38
6057		-0.12	-0.75
3788		-0.13	-0.03
5273		-0.13	0.09
33056		-0.13	-0.51
10965		-0.13	-1.14
12919		-0.13	-0.81
13398		-0.13	0.21

9649		-0.13	-0.54
9428		-0.13	0.21
31915		-0.13	-0.26
33110		-0.13	-1.52
30178		-0.13	-1.14
18265		-0.13	1.31
2108		-0.13	0.00
12058		-0.13	-0.15
1988		-0.13	0.09
4938		-0.13	-0.53
8743		-0.13	1.14
9943		-0.13	1.24
11307		-0.13	-0.66
15255		-0.13	0.54
10535		-0.13	-0.62
3062		-0.13	-0.22
1786		-0.13	-0.30
15564		-0.13	-0.68
3757		-0.13	-1.39
9536		-0.13	-0.25
4663		-0.13	0.33
7995		-0.13	-1.18
14584		-0.13	0.66
4215		-0.13	-0.89
	11545	-0.13	0.52
	17724	-0.13	-0.54
31256		-0.13	0.11
17117		-0.13	1.12
	9511	-0.13	0.68
31302		-0.13	1.02
16720		-0.13	-0.46
31886		-0.13	-0.26
1857		-0.13	2.61
18657		-0.13	-0.47
2929		-0.13	0.19
8365		-0.13	-0.23
7514		-0.13	-0.75
8199		-0.13	-0.63
5555		-0.13	0.48
6878		-0.13	0.00
11105		-0.13	-0.26
	4542	-0.13	-0.77
10811		-0.13	1.11
4550		-0.13	0.69
11218		-0.13	-0.04

5319		-0.13	-0.05
7899		-0.13	0.01
10479		-0.13	-0.25
30017		-0.13	1.03
30325		-0.13	-1.28
5658		-0.13	0.64
	3542	-0.13	0.41
12907		-0.13	0.74
	15711	-0.13	0.41
31794		-0.13	0.13
16896		-0.13	0.75
3248		-0.13	-1.26
11999		-0.13	-0.17
	18417	-0.13	1.94
31182		-0.13	-1.41
	7846	-0.14	1.16
11658		-0.14	-0.87
32806		-0.14	0.22
14251		-0.14	0.67
	19956	-0.14	-1.42
16801		-0.14	0.04
32604		-0.14	0.04
9770		-0.14	0.46
32606		-0.14	-0.19
11815		-0.14	-0.36
	12296	-0.14	0.37
7594		-0.14	0.63
30190		-0.14	-1.00
7408		-0.14	-0.24
14606		-0.14	-1.19
7134		-0.14	0.03
12366		-0.14	0.01
33158		-0.14	-0.74
12021		-0.14	0.21
	17201	-0.14	1.13
4174		-0.14	-0.01
17562		-0.14	-1.97
13106		-0.14	0.48
	19538	-0.14	1.13
7568		-0.14	-0.94
5963		-0.14	0.28
14933		-0.14	-0.28
9204		-0.14	0.04
12766		-0.14	-1.27
6597		-0.14	-0.72

2543		-0.14	1.62
	8642	-0.14	0.55
11417		-0.14	-0.67
	3333	-0.14	0.03
12141		-0.14	0.36
1430		-0.14	-0.58
8838		-0.14	0.39
6571		-0.14	-0.39
	3440	-0.14	0.93
	2927	-0.14	-0.43
1728		-0.14	0.43
10550		-0.14	-0.72
30187		-0.14	0.16
17248		-0.14	-1.43
11077		-0.14	-1.08
32061		-0.14	0.77
12963		-0.14	-0.79
10255		-0.14	0.68
1619		-0.14	-0.10
13072		-0.14	0.50
17976		-0.14	0.22
6975		-0.14	1.83
13323		-0.14	1.19
9790		-0.14	-0.42
13360		-0.14	-0.02
	4624	-0.14	0.52
6106		-0.14	-0.87
3422		-0.14	0.05
6305		-0.14	-0.10
	12390	-0.14	1.03
6711		-0.14	0.31
9755		-0.14	1.44
5836		-0.14	-0.64
18558		-0.14	-0.01
12025		-0.14	0.28
13893		-0.14	-0.52
30149		-0.14	0.20
5018		-0.15	0.03
8946		-0.15	0.65
31146		-0.15	0.46
	19082	-0.15	-0.28
31937		-0.15	0.50
11310		-0.15	0.37
	14118	-0.15	1.04
3123		-0.15	0.07

32984		-0.15	0.29
4780		-0.15	-1.16
33206		-0.15	-0.33
14445		-0.15	0.05
3408		-0.15	0.35
12610		-0.15	0.68
	10336	-0.15	0.65
	15219	-0.15	-0.82
7140		-0.15	0.71
16738		-0.15	-0.23
5948		-0.15	2.16
14070		-0.15	-0.45
11300		-0.15	0.81
17610		-0.15	0.41
8311		-0.15	-0.10
30284		-0.15	-0.18
8317		-0.15	0.66
31803		-0.15	-0.41
	10019	-0.15	-0.74
8765		-0.15	-1.37
1616		-0.15	-0.78
15201		-0.15	-0.61
	3423	-0.15	-0.55
4335		-0.15	0.26
12708		-0.15	0.03
5315		-0.15	0.76
11886		-0.15	-1.29
3168		-0.15	-0.76
31627		-0.15	-0.08
12362		-0.15	-1.26
15859		-0.15	0.06
13054		-0.15	0.48
14064		-0.15	0.81
1836		-0.15	0.69
10698		-0.15	-0.07
7011		-0.15	-0.47
12782		-0.15	0.38
32333		-0.15	-0.13
13982		-0.15	-2.09
15423		-0.15	0.26
13983		-0.15	0.83
4908		-0.15	0.34
6907		-0.15	0.51
9236		-0.15	0.15
11415		-0.15	-0.51

13442		-0.15	1.19
	1541	-0.15	0.84
3497		-0.15	-1.18
5832		-0.15	-0.15
5543		-0.15	-0.54
11793		-0.15	0.23
14872		-0.15	-0.15
2050		-0.15	-1.34
1628		-0.15	0.57
13751		-0.15	0.68
9743		-0.15	-0.25
30273		-0.15	-1.18
33107		-0.15	0.43
1092		-0.15	0.13
16798		-0.15	1.18
14130		-0.15	-1.01
10125		-0.15	0.22
5626		-0.15	-1.23
7608		-0.15	-1.67
4451		-0.15	-1.07
7789		-0.15	-0.33
	11513	-0.15	0.09
14312		-0.15	-0.03
7127		-0.15	-0.27
13762		-0.15	0.01
	9608	-0.16	-1.43
	6297	-0.16	-1.12
13598		-0.16	-1.64
	3043	-0.16	-2.17
	6590	-0.16	0.01
15719		-0.16	-1.43
	2692	-0.16	1.50
14072		-0.16	0.93
9369		-0.16	0.58
14658		-0.16	-1.77
31220		-0.16	0.17
13339		-0.16	-1.19
6269		-0.16	-0.62
4750		-0.16	0.20
14374		-0.16	-0.92
13195		-0.16	-1.43
14926		-0.16	-2.23
12529		-0.16	0.47
7724		-0.16	-0.74
9839		-0.16	0.17

1638		-0.16	-0.55
3567		-0.16	1.25
17097		-0.16	0.04
11041		-0.16	0.35
1707		-0.16	0.54
33289		-0.16	-0.74
32574		-0.16	-1.11
	17037	-0.16	0.01
18530		-0.16	-2.16
	6597	-0.16	0.96
12309		-0.16	-2.04
32698		-0.16	-0.09
6452		-0.16	-1.00
9008		-0.16	-1.89
10685		-0.16	-0.03
12585		-0.16	0.50
14240		-0.16	1.24
12746		-0.16	0.00
10629		-0.16	1.08
1943		-0.16	-0.15
8424		-0.16	1.16
	1521	-0.16	-0.31
32017		-0.16	-1.15
31219		-0.16	-1.41
10332		-0.16	0.39
32087		-0.16	-1.64
	16923	-0.16	-0.28
12992		-0.16	2.67
17717		-0.16	0.24
	7672	-0.16	-1.44
17509		-0.16	0.56
33199		-0.16	1.12
15357		-0.16	-0.63
33274		-0.16	0.04
15351		-0.16	1.05
	1386	-0.16	-0.22
30059		-0.16	-0.08
5605		-0.16	-1.81
31419		-0.16	-0.64
32376		-0.16	-0.18
	6346	-0.16	2.32
17389		-0.16	0.49
9720		-0.16	-0.27
12193		-0.16	-1.07
31660		-0.16	0.59

14689		-0.16	-0.46
3810		-0.16	-0.19
33157		-0.16	-0.58
15046		-0.16	0.84
11149		-0.16	-1.43
18585		-0.16	-1.90
31670		-0.16	0.60
3803		-0.16	-1.11
	2777	-0.16	0.20
5726		-0.16	-0.01
7929		-0.16	-0.67
14641		-0.16	-0.99
1298		-0.17	0.08
3315		-0.17	-1.16
13210		-0.17	-0.12
	13909	-0.17	-0.15
1428		-0.17	-0.26
15889		-0.17	-0.99
4440		-0.17	-0.08
31752		-0.17	0.54
3057		-0.17	-0.43
3331		-0.17	-0.42
13086		-0.17	0.24
12847		-0.17	-0.05
	15221	-0.17	0.75
3191		-0.17	-0.60
7963		-0.17	-0.44
32656		-0.17	-0.40
15143		-0.17	0.00
15245		-0.17	0.28
13905		-0.17	-0.50
1126		-0.17	1.51
10654		-0.17	1.09
17618		-0.17	0.60
13988		-0.17	0.38
8995		-0.17	-0.72
15431		-0.17	-0.39
33109		-0.17	-0.39
33218		-0.17	-1.36
17010		-0.17	-1.53
7793		-0.17	-0.65
15602		-0.17	-0.91
2663		-0.17	-2.07
18066		-0.17	-0.02
	19672	-0.17	0.33

	18479	-0.17	0.62
9062		-0.17	-1.14
3683		-0.17	-2.40
7975		-0.17	-0.05
7362		-0.17	0.57
7757		-0.17	-0.48
31841		-0.17	0.39
10691		-0.17	-1.28
	18028	-0.17	0.34
	13913	-0.17	0.10
14618		-0.17	-1.41
5608		-0.17	-0.90
13888		-0.17	0.00
	18204	-0.17	0.33
7301		-0.17	-0.53
9249		-0.17	0.52
7663		-0.17	0.24
5343		-0.17	0.03
14297		-0.17	-0.26
31263		-0.17	1.66
5346		-0.17	0.10
11447		-0.17	0.14
	6210	-0.17	0.29
11851		-0.17	0.05
2010		-0.17	0.40
13183		-0.17	-1.43
10036		-0.17	0.01
10648		-0.17	1.19
7118		-0.17	0.69
	12505	-0.17	1.71
14934		-0.17	-0.75
5367		-0.17	-0.39
17381		-0.17	-2.04
14211		-0.17	
32477		-0.17	0.34
10073		-0.17	1.60
3841		-0.17	-1.00
18208		-0.17	0.09
9363		-0.17	0.57
10289		-0.17	-0.87
2928		-0.17	-0.27
4999		-0.17	0.56
7840		-0.17	-0.29
6480		-0.17	-0.16
3136		-0.17	-0.75

6117		-0.17	-0.07
	6626	-0.17	-1.65
31834		-0.17	-0.10
31776		-0.17	0.25
	12875	-0.17	0.03
17198		-0.17	0.98
5991		-0.17	-0.13
4971		-0.17	-1.83
30150		-0.17	0.46
16736		-0.17	-0.29
10850		-0.17	0.73
11091		-0.17	-0.51
10463		-0.17	0.91
31961		-0.17	-0.34
18779		-0.17	0.04
3962		-0.17	0.39
6149		-0.17	-1.16
	12755	-0.17	-0.13
	7159	-0.18	0.41
6108		-0.18	0.28
	9505	-0.18	1.41
	12611	-0.18	0.44
5053		-0.18	-0.67
13208		-0.18	-0.07
	15048	-0.18	-0.32
12398		-0.18	0.01
18543		-0.18	-1.50
5988		-0.18	-0.80
31411		-0.18	-0.05
6741		-0.18	-1.18
	9081	-0.18	0.37
17086		-0.18	-1.40
4711		-0.18	0.34
8251		-0.18	-1.07
	11114	-0.18	-0.31
	1345	-0.18	0.16
30076		-0.18	0.20
7050		-0.18	-0.86
11563		-0.18	0.24
5224		-0.18	0.89
	3861	-0.18	-0.31
9722		-0.18	-1.02
5886		-0.18	-0.48
14709		-0.18	-3.28
1319		-0.18	-0.27

9786		-0.18	-0.90
	2703	-0.18	-0.28
1774		-0.18	-0.09
7658		-0.18	1.53
10184		-0.18	-1.63
9949		-0.18	0.04
4185		-0.18	-0.62
4872		-0.18	-0.62
11518		-0.18	0.62
5737		-0.18	-0.17
	2259	-0.18	-1.80
18823		-0.18	-0.63
8292		-0.18	-0.66
13311		-0.18	0.70
31380		-0.18	-1.18
8111		-0.18	0.27
1725		-0.18	0.30
17782		-0.18	-0.02
14075		-0.18	0.23
32106		-0.18	0.27
14787		-0.18	-0.32
4735		-0.18	0.32
14967		-0.18	-1.54
15887		-0.18	-0.07
7295		-0.18	-0.41
17137		-0.18	0.29
5862		-0.18	-0.23
8136		-0.18	1.46
1737		-0.18	0.52
17523		-0.18	-0.11
5734		-0.18	0.26
	202	-0.18	0.33
1146		-0.18	-0.43
12287		-0.18	-0.84
	6496	-0.18	1.16
8404		-0.18	0.74
14714		-0.18	0.54
	3943	-0.18	1.08
1240		-0.18	0.93
7369		-0.18	-1.75
	3263	-0.18	0.00
2105		-0.18	-0.05
12313		-0.18	0.62
	1525	-0.18	-1.05
16793		-0.18	-0.61

	6050	-0.18	0.74
32954		-0.18	-1.42
8966		-0.18	-1.53
	7620	-0.18	1.03
32772		-0.18	-0.36
1448		-0.19	1.21
	16804	-0.19	-1.15
10388		-0.19	-0.51
32407		-0.19	-1.20
11498		-0.19	0.80
2247		-0.19	0.08
17806		-0.19	0.00
	13484	-0.19	0.23
8616		-0.19	-1.34
32134		-0.19	-1.11
11866		-0.19	-0.08
1447		-0.19	-0.47
8316		-0.19	0.56
13882		-0.19	-0.05
6040		-0.19	-1.36
	10437	-0.19	0.45
5334		-0.19	1.04
10238		-0.19	-0.03
13186		-0.19	-1.63
	1358	-0.19	0.37
15517		-0.19	0.79
8409		-0.19	1.01
4567		-0.19	-0.47
12048		-0.19	-0.03
3919		-0.19	0.44
11035		-0.19	0.20
15008		-0.19	0.69
15765		-0.19	1.15
7974		-0.19	0.01
31795		-0.19	-1.08
11756		-0.19	-1.26
10575		-0.19	0.22
15786		-0.19	-2.40
	12259	-0.19	-0.62
3020		-0.19	0.24
33453		-0.19	0.66
	7194	-0.19	0.44
	13410	-0.19	0.97
7949		-0.19	0.18
	16228	-0.19	0.39

9659		-0.19	-0.80
10102		-0.19	0.32
33200		-0.19	0.37
13373		-0.19	-1.74
	3939	-0.19	0.37
11926		-0.19	0.77
1129		-0.19	0.02
17121		-0.19	0.00
	9961	-0.19	-0.82
6766		-0.19	-1.25
	3394	-0.19	-1.35
5681		-0.19	0.48
1994		-0.19	-0.56
6982		-0.19	0.10
	8765	-0.19	0.46
8089		-0.19	0.15
6525		-0.19	-1.09
31620		-0.19	-0.48
33142		-0.19	-0.59
6773		-0.19	0.21
9438		-0.19	0.00
	20116	-0.19	-1.12
12943		-0.19	-0.01
12824		-0.19	-0.89
32528		-0.19	-1.13
30075		-0.19	0.15
6433		-0.19	-0.13
12162		-0.19	0.00
1171		-0.19	-0.55
6450		-0.19	0.83
14396		-0.19	0.73
11835		-0.19	-1.37
1349		-0.19	-0.19
	9664	-0.19	0.31
	6929	-0.19	0.17
12480		-0.19	-0.22
	2593	-0.19	-1.23
5194		-0.19	0.26
11763		-0.19	-2.33
	8933	-0.19	-0.08
10621		-0.19	0.19
14883		-0.19	-0.25
2342		-0.19	2.14
10979		-0.19	0.11
13898		-0.19	-1.54

1488		-0.19	-0.97
1569		-0.19	0.09
11975		-0.20	0.31
10589		-0.20	-0.44
6675		-0.20	0.72
13197		-0.20	0.31
5323		-0.20	0.60
14004		-0.20	-0.20
7556		-0.20	0.42
1583		-0.20	-0.30
	3684	-0.20	-0.16
32756		-0.20	-1.18
17158		-0.20	1.42
18624		-0.20	-0.78
4623		-0.20	-0.80
2297		-0.20	0.11
7635		-0.20	-1.11
5893		-0.20	-0.21
5404		-0.20	-0.39
12948		-0.20	-1.99
1236		-0.20	-0.16
	8052	-0.20	-0.81
12807		-0.20	-0.53
11986		-0.20	0.30
	5885	-0.20	0.31
9680		-0.20	-0.57
31448		-0.20	-0.33
	7645	-0.20	-1.73
8778		-0.20	1.05
1827		-0.20	-0.26
6066		-0.20	-0.11
1623		-0.20	-0.42
31847		-0.20	-0.16
31999		-0.20	0.49
	13504	-0.20	-0.07
	5586	-0.20	0.01
6343		-0.20	0.92
11362		-0.20	-1.43
12904		-0.20	1.08
18361		-0.20	0.35
1968		-0.20	-0.96
11807		-0.20	0.15
7100		-0.20	1.08
10749		-0.20	-1.38
	14137	-0.20	0.40

8457		-0.20	1.09
15316		-0.20	0.11
9070		-0.20	0.30
12342		-0.20	-0.20
11315		-0.20	0.65
9458		-0.20	1.19
8103		-0.20	0.27
8280		-0.20	2.11
10144		-0.20	-1.22
8287		-0.20	-0.10
3291		-0.20	-0.57
3663		-0.20	-1.17
4690		-0.20	0.52
31326		-0.20	0.10
	2141	-0.20	0.96
12946		-0.20	-0.61
30360		-0.20	0.00
	18327	-0.20	-1.00
11941		-0.20	-0.15
32255		-0.20	0.24
	17697	-0.20	0.58
31663		-0.20	-0.50
11897		-0.20	-1.75
32694		-0.20	-0.49
30396		-0.20	0.18
14997		-0.20	-0.16
14262		-0.20	-0.99
6519		-0.20	1.09
5181		-0.20	1.00
31267		-0.20	0.21
3093		-0.20	0.66
33457		-0.21	-0.26
8338		-0.21	-0.94
33232		-0.21	-0.04
15591		-0.21	-0.42
17751		-0.21	0.39
4956		-0.21	-0.05
32557		-0.21	0.69
3829		-0.21	-1.58
1363		-0.21	0.32
14066		-0.21	-1.05
	19173	-0.21	-0.99
1765		-0.21	1.21
2855		-0.21	-0.90
30030		-0.21	-1.45

14492		-0.21	0.32
5509		-0.21	-1.27
11356		-0.21	-0.65
	16186	-0.21	1.50
8610		-0.21	-0.94
5677		-0.21	-0.13
8063		-0.21	-0.90
6785		-0.21	0.99
13521		-0.21	0.09
	16722	-0.21	0.39
15199		-0.21	-0.52
33327		-0.21	0.60
4936		-0.21	-0.74
1128		-0.21	0.35
14455		-0.21	0.80
5038		-0.21	-0.62
8696		-0.21	-0.65
	6741	-0.21	-0.23
12134		-0.21	-0.56
8532		-0.21	-1.16
6741		-0.21	0.38
31762		-0.21	1.82
11269		-0.21	-0.46
14143		-0.21	-1.70
11060		-0.21	0.35
17327		-0.21	-0.36
13591		-0.21	-2.80
	11647	-0.21	-0.28
9686		-0.21	-0.87
32111		-0.21	-1.53
8288		-0.21	0.85
10367		-0.21	-1.82
13712		-0.21	-0.52
	9647	-0.21	0.24
14928		-0.21	-1.02
5206		-0.21	-1.16
	18328	-0.21	0.52
31849		-0.21	0.18
10686		-0.21	0.28
14089		-0.21	0.46
	11123	-0.21	-0.51
7563		-0.21	0.18
5471		-0.21	0.39
	2932	-0.21	0.78
11494		-0.21	-0.14

13952		-0.21	0.09
31421		-0.21	-0.44
31990		-0.21	1.05
30185		-0.21	-1.45
2845		-0.21	0.25
5421		-0.21	0.26
9673		-0.21	-0.08
3226		-0.21	0.99
2467		-0.21	-0.59
7294		-0.21	0.02
14896		-0.21	-0.90
12899		-0.21	0.00
7526		-0.22	-0.78
5721		-0.22	-0.03
6672		-0.22	-1.18
10446		-0.22	-1.31
5993		-0.22	0.20
	19919	-0.22	0.83
31815		-0.22	-0.62
4451		-0.22	0.45
3610		-0.22	-0.14
3945		-0.22	0.50
15375		-0.22	0.50
	9484	-0.22	0.62
33323		-0.22	1.26
	4228	-0.22	1.17
30069		-0.22	-1.65
	5759	-0.22	0.07
12057		-0.22	0.70
	10996	-0.22	-0.55
7280		-0.22	-0.78
8942		-0.22	-0.51
30263		-0.22	-0.47
2187		-0.22	0.87
	6635	-0.22	-0.27
32029		-0.22	0.86
3313		-0.22	-0.74
	11504	-0.22	0.40
15658		-0.22	-0.28
3361		-0.22	-0.99
14221		-0.22	
32816		-0.22	-0.17
	6725	-0.22	-0.01
7189		-0.22	-0.01
12998		-0.22	-0.86

8211		-0.22	-1.37
	6847	-0.22	-0.78
7281		-0.22	-0.97
9139		-0.22	0.39
13390		-0.22	-0.40
13164		-0.22	-1.55
15069		-0.22	0.03
5707		-0.22	0.12
6707		-0.22	0.20
31413		-0.22	0.62
	5792	-0.22	0.58
17382		-0.22	-2.15
14129		-0.22	0.95
12306		-0.22	-0.80
7631		-0.22	-0.02
	9969	-0.22	-0.63
6347		-0.22	-0.07
11170		-0.22	0.64
30061		-0.22	-1.34
14947		-0.22	-1.28
	19529	-0.22	0.53
15638		-0.22	0.26
10710		-0.22	-1.12
9113		-0.22	-0.25
3004		-0.22	-0.82
7488		-0.22	-0.01
	6258	-0.22	0.93
8601		-0.22	0.34
5540		-0.22	-1.34
10221		-0.22	-0.91
32257		-0.22	-0.54
	16478	-0.22	-0.29
30089		-0.22	-1.08
31997		-0.22	-0.51
8933		-0.22	1.19
	6510	-0.22	0.76
17189		-0.22	-1.48
32458		-0.22	-1.25
17662		-0.22	0.21
13993		-0.22	0.45
1946		-0.22	-0.88
4195		-0.23	-0.23
	4641	-0.23	0.01
32204		-0.23	-0.26
7179		-0.23	-0.06

31052		-0.23	-0.47
	10481	-0.23	0.15
1468		-0.23	-0.75
10308		-0.23	0.51
33224		-0.23	0.60
	10750	-0.23	0.13
5732		-0.23	0.00
	18361	-0.23	0.95
9120		-0.23	-0.87
13702		-0.23	0.00
2211		-0.23	0.04
9261		-0.23	-1.22
18013		-0.23	0.18
17264		-0.23	0.34
11527		-0.23	-0.21
3762		-0.23	-0.59
	18631	-0.23	-1.19
12149		-0.23	0.24
1887		-0.23	0.07
32096		-0.23	-0.30
	16577	-0.23	-0.16
10717		-0.23	-0.99
5959		-0.23	0.02
13843		-0.23	0.36
11597		-0.23	0.14
5974		-0.23	-0.48
1572		-0.23	-1.24
9668		-0.23	-1.45
	7433	-0.23	-0.16
3500		-0.23	-1.46
11875		-0.23	-1.14
3478		-0.23	-1.68
10130		-0.23	-0.29
13141		-0.23	0.37
7599		-0.23	0.83
3429		-0.23	-1.29
5151		-0.23	0.51
7544		-0.23	-0.63
2183		-0.23	-0.46
32075		-0.23	0.36
3095		-0.23	0.02
7413		-0.23	0.00
32770		-0.23	0.97
9048		-0.23	-0.02
12770		-0.23	-0.42

11779		-0.23	-0.11
6821		-0.23	-0.53
8200		-0.23	-0.19
2038		-0.23	-0.52
5486		-0.23	1.56
	2029	-0.23	0.44
15618		-0.23	-1.63
12444		-0.23	1.21
17044		-0.23	-0.15
7149		-0.23	0.53
12653		-0.23	0.00
11145		-0.23	-2.00
13171		-0.23	-0.40
18234		-0.23	-0.96
11592		-0.23	-1.64
7038		-0.23	-0.92
9183		-0.23	0.56
10163		-0.23	-0.32
	16081	-0.23	0.97
	13250	-0.23	0.00
11180		-0.23	-1.27
31211		-0.23	1.09
33153		-0.23	-0.84
8798		-0.23	0.33
18730		-0.23	0.53
	12756	-0.23	-0.17
13539		-0.23	-0.77
12880		-0.23	0.96
11902		-0.24	-0.74
33247		-0.24	-0.01
12780		-0.24	0.05
32714		-0.24	-0.02
12814		-0.24	-1.56
4775		-0.24	0.80
13027		-0.24	0.32
12191		-0.24	-0.43
	7247	-0.24	-0.58
13784		-0.24	-0.06
31882		-0.24	-1.77
31872		-0.24	0.62
14249		-0.24	0.23
6963		-0.24	0.12
10907		-0.24	-0.79
4624		-0.24	0.82
	2124	-0.24	-0.50

8360		-0.24	-0.55
10704		-0.24	1.08
9095		-0.24	0.26
16954		-0.24	0.58
31647		-0.24	-1.35
17054		-0.24	-0.22
10341		-0.24	0.33
31212		-0.24	0.43
7047		-0.24	-0.88
	15117	-0.24	-0.74
15344		-0.24	1.13
5712		-0.24	-0.71
3312		-0.24	-0.23
17788		-0.24	-2.45
	19717	-0.24	0.35
31405		-0.24	-1.37
14691		-0.24	0.14
14407		-0.24	1.34
18572		-0.24	-1.10
12395		-0.24	0.84
11317		-0.24	1.39
4019		-0.24	-1.57
7423		-0.24	0.74
	19873	-0.24	0.43
2803		-0.24	-0.54
31777		-0.24	0.09
	9245	-0.24	-1.66
33343		-0.24	-1.35
	18217	-0.24	-0.98
13258		-0.24	0.46
6493		-0.24	0.30
14339		-0.24	0.78
30127		-0.24	-1.42
6363		-0.24	-0.69
17241		-0.24	-0.14
	7130	-0.24	-1.04
16741		-0.24	0.08
	15760	-0.24	-0.52
30175		-0.24	-0.65
	12767	-0.24	0.18
3270		-0.24	0.18
	18329	-0.24	0.32
30280		-0.24	0.13
7425		-0.24	0.62
31348		-0.24	1.12

4270		-0.24	-0.55
	11233	-0.24	-1.38
6236		-0.24	0.08
4064		-0.24	-0.21
9520		-0.25	0.24
8568		-0.25	-0.76
	11532	-0.25	0.18
4673		-0.25	-0.34
15125		-0.25	-2.31
6705		-0.25	-1.44
7299		-0.25	0.84
3425		-0.25	-0.28
32345		-0.25	0.04
5108		-0.25	-0.73
4220		-0.25	0.29
7450		-0.25	0.10
8804		-0.25	-0.10
10866		-0.25	-0.08
1609		-0.25	-0.87
30100		-0.25	-0.20
4709		-0.25	0.41
	17276	-0.25	-1.16
2657		-0.25	-0.51
	5732	-0.25	-0.38
	8947	-0.25	0.02
	5090	-0.25	-0.31
8779		-0.25	1.05
	7439	-0.25	-0.73
11231		-0.25	-1.40
32636		-0.25	0.40
11780		-0.25	0.29
15709		-0.25	-1.57
17725		-0.25	0.22
3491		-0.25	-1.21
	8030	-0.25	-0.41
4696		-0.25	-0.57
10001		-0.25	-0.20
13071		-0.25	1.23
31108		-0.25	0.74
6120		-0.25	0.93
11348		-0.25	-1.55
18672		-0.25	-0.31
5441		-0.25	0.33
32448		-0.25	0.10
13048		-0.25	-0.26

11376		-0.25	-0.48
4545		-0.25	-0.29
7277		-0.25	0.01
8407		-0.25	-0.33
32708		-0.25	0.41
	18313	-0.25	0.53
14911		-0.25	-0.81
5463		-0.25	-0.68
3654		-0.25	-0.63
7719		-0.25	-0.87
3386		-0.25	-0.36
	9850	-0.25	-0.05
	1527	-0.25	-0.92
10933		-0.25	-0.06
7264		-0.25	0.20
13626		-0.25	-0.37
	14620	-0.25	-0.37
11352		-0.25	0.58
4101		-0.25	0.47
4643		-0.25	0.71
15777		-0.25	1.25
4839		-0.25	0.69
11539		-0.25	-0.16
	14921	-0.25	-1.25
10904		-0.25	1.22
11098		-0.25	0.74
15317		-0.25	0.32
5623		-0.25	0.67
33294		-0.25	-1.61
6726		-0.25	-0.08
	2784	-0.25	0.57
	20041	-0.25	1.14
15539		-0.25	-0.46
9852		-0.25	0.04
17252		-0.25	0.18
	3045	-0.25	-2.26
14957		-0.25	-0.14
32045		-0.25	0.48
	2240	-0.25	-1.68
6464		-0.25	-0.79
1501		-0.25	0.53
32808		-0.25	-0.20
11760		-0.25	1.13
12840		-0.25	-0.77
17146		-0.25	-1.26

12932		-0.25	0.01
15120		-0.25	-1.09
9134		-0.25	0.07
11382		-0.25	0.23
	7747	-0.25	0.07
	17806	-0.26	-2.56
	18739	-0.26	-0.28
9934		-0.26	1.08
7137		-0.26	-1.41
	13292	-0.26	0.74
	4194	-0.26	-0.44
8100		-0.26	-1.69
7105		-0.26	-0.26
	7756	-0.26	-0.75
12792		-0.26	0.77
5398		-0.26	-0.77
12163		-0.26	0.20
1486		-0.26	-3.27
	19002	-0.26	-1.44
	290	-0.26	-0.75
6717		-0.26	-0.44
14695		-0.26	-0.92
18563		-0.26	-1.26
2316		-0.26	-1.22
13968		-0.26	-0.43
5270		-0.26	-0.49
7386		-0.26	-0.19
7285		-0.26	0.01
7824		-0.26	-1.25
13921		-0.26	-0.74
1354		-0.26	-0.89
31218		-0.26	0.68
9904		-0.26	-0.45
6213		-0.26	-1.20
	18559	-0.26	0.20
17601		-0.26	0.07
	2615	-0.26	-1.22
14835		-0.26	-1.89
	5714	-0.26	-1.39
12438		-0.26	0.79
31256		-0.26	-0.48
	12482	-0.26	-0.15
31793		-0.26	0.58
	9504	-0.26	1.40
	8083	-0.26	0.13

32331		-0.26	-0.20
31756		-0.26	-1.75
31920		-0.26	0.28
7754		-0.26	0.00
11340		-0.26	0.50
3454		-0.26	0.76
	13282	-0.26	0.71
5043		-0.26	-1.60
9986		-0.26	0.36
1140		-0.26	0.18
13640		-0.26	-1.48
30459		-0.26	0.85
7832		-0.26	0.35
31201		-0.26	-0.89
	20130	-0.26	-0.26
31014		-0.26	0.14
4250		-0.26	0.20
5519		-0.26	0.27
33194		-0.26	0.19
9090		-0.26	-0.38
12147		-0.26	1.28
14880		-0.26	-1.12
8839		-0.26	-0.66
11375		-0.26	-0.97
14302		-0.26	-0.98
31551		-0.26	0.03
9437		-0.26	-0.75
7314		-0.26	1.66
4423		-0.26	0.40
1979		-0.26	0.91
	8979	-0.26	-0.33
6999		-0.26	0.62
4216		-0.26	-0.33
15179		-0.26	-0.87
15369		-0.26	0.80
30042		-0.26	-0.15
9511		-0.26	-0.88
5562		-0.27	-0.18
4963		-0.27	-1.07
8478		-0.27	-0.78
	471	-0.27	-1.06
13744		-0.27	0.28
9418		-0.27	-0.37
5442		-0.27	-1.02
	12436	-0.27	-0.57

	13759	-0.27	0.63
4557		-0.27	-0.61
4164		-0.27	-0.39
14394		-0.27	-0.45
5521		-0.27	1.28
3800		-0.27	-1.53
32316		-0.27	-0.20
32951		-0.27	-0.16
17814		-0.27	-0.59
15754		-0.27	1.81
	15754	-0.27	-0.71
5462		-0.27	-0.44
5022		-0.27	-0.24
15167		-0.27	-1.49
9747		-0.27	1.48
10889		-0.27	0.00
15128		-0.27	-0.25
13925		-0.27	0.38
17378		-0.27	-0.10
4920		-0.27	-0.18
3104		-0.27	-0.18
12869		-0.27	-0.69
5025		-0.27	-0.76
11828		-0.27	-0.34
11951		-0.27	-0.35
14543		-0.27	-0.08
14301		-0.27	-0.25
15086		-0.27	-1.39
32701		-0.27	-0.14
6981		-0.27	-0.20
	5165	-0.27	0.00
10168		-0.27	-1.02
5026		-0.27	-0.49
17821		-0.27	-0.24
3026		-0.27	0.45
7085		-0.27	-0.35
14426		-0.27	0.58
10874		-0.27	-1.04
1902		-0.27	0.34
1075		-0.27	0.31
6357		-0.27	-1.18
9040		-0.27	0.32
30398		-0.27	-0.41
1503		-0.27	-0.82
31690		-0.27	-0.05

9068		-0.27	1.02
3698		-0.27	0.61
11262		-0.27	0.49
9053		-0.27	-0.82
33147		-0.27	0.71
8266		-0.27	0.29
32647		-0.27	-0.62
32855		-0.27	-0.01
9807		-0.27	-0.76
	16725	-0.27	0.51
14590		-0.27	-1.98
	9507	-0.27	0.77
	7321	-0.27	-2.75
3469		-0.28	1.43
	6250	-0.28	0.90
4041		-0.28	0.00
	8292	-0.28	-2.61
	7447	-0.28	0.26
33017		-0.28	-0.05
7884		-0.28	0.29
11291		-0.28	0.82
1093		-0.28	-0.63
	5699	-0.28	-1.56
	2975	-0.28	0.52
10492		-0.28	0.90
14061		-0.28	-0.06
4003		-0.28	0.32
12661		-0.28	0.80
17266		-0.28	-2.05
15822		-0.28	1.13
11686		-0.28	-2.12
	12431	-0.28	-0.07
7705		-0.28	-0.45
12498		-0.28	0.64
33462		-0.28	0.27
12958		-0.28	-1.85
31546		-0.28	0.16
13503		-0.28	0.86
4779		-0.28	-0.61
18432		-0.28	-2.43
	4031	-0.28	1.01
15910		-0.28	-0.75
	11875	-0.28	0.60
	2928	-0.28	-0.27
8934		-0.28	0.94

32659		-0.28	0.39
18508		-0.28	-0.23
	11854	-0.28	0.16
7199		-0.28	-1.43
12883		-0.28	0.00
30431		-0.28	-0.68
3410		-0.28	-1.68
32486		-0.28	0.52
5155		-0.28	-0.11
14205		-0.28	-0.42
5476		-0.28	1.25
13914		-0.28	0.05
12821		-0.28	0.47
2641		-0.28	0.89
7361		-0.28	0.54
5541		-0.28	-2.38
	16607	-0.28	-0.28
5208		-0.28	1.07
33052		-0.28	-0.38
3265		-0.28	-0.18
10200		-0.28	-0.08
	19931	-0.28	-3.02
18000		-0.28	0.23
14355		-0.28	-0.26
16984		-0.28	1.29
7713		-0.28	-0.02
33134		-0.28	1.96
13721		-0.28	0.32
7527		-0.28	-1.26
11591		-0.28	-1.50
4658		-0.28	-0.85
8268		-0.28	0.74
31532		-0.28	0.99
8369		-0.28	1.68
31206		-0.28	0.76
9682		-0.28	-0.29
11181		-0.28	0.23
	2764	-0.28	-0.24
14925		-0.28	-1.91
31084		-0.28	-0.01
33193		-0.28	0.16
30342		-0.28	1.04
11997		-0.28	-0.85
5126		-0.28	0.07
11271		-0.28	2.86

12891		-0.28	0.22
4706		-0.29	0.07
33241		-0.29	0.27
11513		-0.29	-0.34
1836		-0.29	0.96
6530		-0.29	-0.51
8657		-0.29	0.63
32038		-0.29	-1.47
30078		-0.29	-1.17
2865		-0.29	-1.90
12532		-0.29	-0.40
8998		-0.29	0.05
7259		-0.29	-0.89
4045		-0.29	-0.47
18557		-0.29	-0.88
	12550	-0.29	-1.05
9593		-0.29	0.44
15767		-0.29	-2.07
6718		-0.29	-1.59
	6366	-0.29	0.50
	17478	-0.29	-1.46
9505		-0.29	-0.56
14196		-0.29	0.81
	3502	-0.29	-1.06
9616		-0.29	0.02
11579		-0.29	0.01
4052		-0.29	0.41
9432		-0.29	0.35
7753		-0.29	0.12
31482		-0.29	-0.38
7098		-0.29	-0.53
	7256	-0.29	-0.35
31950		-0.29	-0.12
11062		-0.29	-0.07
10748		-0.29	-1.12
10153		-0.29	-0.18
	12632	-0.29	0.36
31560		-0.29	0.40
4661		-0.29	0.39
14948		-0.29	0.41
31472		-0.29	0.16
4586		-0.29	-1.09
11735		-0.29	-0.14
	12541	-0.29	1.17
	9519	-0.29	1.28

17347		-0.29	-1.06
17829		-0.29	0.31
5182		-0.29	0.65
17637		-0.29	-1.61
4165		-0.29	-0.71
6620		-0.29	0.12
	336	-0.29	0.18
11607		-0.29	0.31
8337		-0.29	-0.69
	10635	-0.29	0.26
33299		-0.29	-1.89
14443		-0.29	0.26
10693		-0.29	-1.94
17320		-0.29	-0.43
32264		-0.29	0.29
	17318	-0.29	-0.03
7133		-0.29	-0.73
2765		-0.29	-0.75
7838		-0.29	0.01
32099		-0.29	-0.67
	16544	-0.29	-0.82
7611		-0.29	0.22
10537		-0.29	-1.26
	658	-0.29	-2.54
12593		-0.29	-0.04
7825		-0.29	-0.22
11777		-0.29	-0.83
31797		-0.29	-0.08
12720		-0.29	-1.12
3671		-0.29	-1.32
9121		-0.29	1.01
4585		-0.29	-1.42
31733		-0.29	-1.36
6481		-0.29	-0.07
	8419	-0.29	0.41
32079		-0.30	0.53
14328		-0.30	0.55
	16354	-0.30	0.54
1483		-0.30	-1.73
	11858	-0.30	-0.55
	7168	-0.30	-0.30
7249		-0.30	0.18
14131		-0.30	0.37
31952		-0.30	-0.25
12621		-0.30	0.43

9629		-0.30	-0.08
6687		-0.30	-0.80
	9512	-0.30	0.36
4904		-0.30	0.77
	6630	-0.30	-0.19
5376		-0.30	-0.09
30432		-0.30	-0.67
11961		-0.30	0.77
8440		-0.30	0.97
2121		-0.30	-0.14
10148		-0.30	0.14
4354		-0.30	0.61
31320		-0.30	0.11
3666		-0.30	-0.62
14531		-0.30	0.00
	16037	-0.30	0.20
5504		-0.30	0.52
	12575	-0.30	0.15
	20049	-0.30	-0.01
32021		-0.30	-0.63
12845		-0.30	0.09
9984		-0.30	-0.30
13419		-0.30	-0.82
1830		-0.30	-2.40
	12377	-0.30	-0.16
30021		-0.30	0.76
33172		-0.30	-0.82
9203		-0.30	-1.48
8920		-0.30	0.04
14398		-0.30	0.65
	9677	-0.30	0.43
9097		-0.30	-0.45
18789		-0.30	0.20
	12199	-0.30	-0.57
	17490	-0.30	0.56
31802		-0.30	0.40
11757		-0.30	0.74
14283		-0.30	-1.49
13560		-0.30	-1.19
4162		-0.30	0.00
11893		-0.30	0.12
	19075	-0.30	-0.20
5897		-0.30	0.43
6540		-0.30	-0.04
	17476	-0.30	0.15

9399		-0.30	-0.84
14049		-0.30	-1.41
7466		-0.30	-0.62
12101		-0.30	-0.13
6836		-0.30	0.39
8666		-0.30	0.00
6012		-0.30	3.24
12673		-0.30	-2.09
	14516	-0.30	0.18
9378		-0.30	-1.27
6719		-0.30	-1.22
32281		-0.30	-0.77
	11103	-0.30	-0.42
5642		-0.30	0.08
14617		-0.30	0.88
9865		-0.30	-0.86
11201		-0.30	-1.50
15194		-0.30	0.64
8965		-0.30	-0.98
14974		-0.31	-0.99
32233		-0.31	-0.69
12002		-0.31	0.80
33348		-0.31	0.06
17217		-0.31	0.90
9877		-0.31	-0.33
31652		-0.31	0.01
17946		-0.31	-1.00
10405		-0.31	0.38
10390		-0.31	-0.20
7018		-0.31	-1.08
15766		-0.31	0.69
14068		-0.31	0.03
5384		-0.31	-1.75
11330		-0.31	0.41
3696		-0.31	-1.01
8470		-0.31	-0.86
7033		-0.31	-0.20
18561		-0.31	-0.49
6775		-0.31	-2.26
12558		-0.31	0.40
13224		-0.31	-0.45
1632		-0.31	-0.18
7603		-0.31	-0.08
1500		-0.31	-0.27
1550		-0.31	-1.26

7993		-0.31	-1.54
32412		-0.31	-1.06
	11021	-0.31	0.67
31451		-0.31	-1.56
4206		-0.31	-0.42
5317		-0.31	-0.56
31910		-0.31	-0.20
	12613	-0.31	0.47
10504		-0.31	-0.92
31740		-0.31	0.11
7021		-0.31	0.47
18490		-0.31	0.29
30179		-0.31	-0.95
15828		-0.31	1.21
8991		-0.31	0.33
7584		-0.31	-0.39
15250		-0.31	-0.27
8367		-0.31	-1.77
	15897	-0.31	-1.82
9241		-0.31	-0.67
1411		-0.31	-0.79
8549		-0.31	0.51
4749		-0.31	0.09
32429		-0.31	0.14
	12064	-0.31	-1.11
31772		-0.31	0.02
9953		-0.31	0.61
9159		-0.31	0.30
17127		-0.31	0.72
2444		-0.31	0.10
6598		-0.31	-0.87
32660		-0.31	0.48
7601		-0.31	0.50
7670		-0.31	0.31
	19375	-0.31	-2.13
2227		-0.31	0.83
8675		-0.31	-1.48
8318		-0.31	0.54
33304		-0.31	-0.68
17912		-0.31	-2.06
15821		-0.31	0.88
5164		-0.31	1.50
3831		-0.31	-1.27
5550		-0.31	-0.19
9149		-0.31	-0.35

8479		-0.32	-0.01
	495	-0.32	-0.12
13746		-0.32	0.80
8714		-0.32	-0.30
9289		-0.32	1.09
	14460	-0.32	-1.88
6417		-0.32	0.61
3124		-0.32	-1.93
10483		-0.32	-0.44
8239		-0.32	0.28
4622		-0.32	0.39
31828		-0.32	0.00
10560		-0.32	-0.29
9715		-0.32	0.31
10103		-0.32	-1.44
13365		-0.32	-1.24
3287		-0.32	0.45
12537		-0.32	-0.28
	14421	-0.32	0.42
18211		-0.32	0.19
3988		-0.32	-1.26
15231		-0.32	-1.10
	4695	-0.32	-0.73
11836		-0.32	-0.07
10372		-0.32	-1.59
15927		-0.32	-1.27
13894		-0.32	-0.11
30011		-0.32	-1.36
13565		-0.32	0.68
32763		-0.32	-0.74
30067		-0.32	0.23
	6519	-0.32	-0.31
	14513	-0.32	-0.01
32191		-0.32	0.52
8939		-0.32	-0.48
3548		-0.32	-1.16
	16918	-0.32	-0.31
15706		-0.32	0.06
6517		-0.32	0.75
	15389	-0.32	0.12
14321		-0.32	-1.25
	15747	-0.32	-1.68
31601		-0.32	-2.23
	2111	-0.32	-0.58
15733		-0.32	-0.62

1421		-0.32	0.34
	9225	-0.32	-2.30
11164		-0.32	0.68
12873		-0.32	-0.37
31808		-0.32	-0.71
32699		-0.32	1.36
	10656	-0.32	0.35
18146		-0.32	0.08
15529		-0.32	1.17
5983		-0.33	-0.12
8757		-0.33	0.34
	11508	-0.33	-0.06
16922		-0.33	0.02
	6600	-0.33	0.39
15276		-0.33	-0.62
9591		-0.33	-0.54
9209		-0.33	0.64
	12790	-0.33	-0.38
17799		-0.33	0.05
10880		-0.33	-1.53
31648		-0.33	-0.36
15049		-0.33	0.31
	5839	-0.33	-0.01
8510		-0.33	0.13
14014		-0.33	-0.92
	9230	-0.33	0.06
	2252	-0.33	-1.09
5634		-0.33	-1.06
	11611	-0.33	0.48
14185		-0.33	0.13
2269		-0.33	0.35
15736		-0.33	-0.07
14754		-0.33	0.07
	1954	-0.33	0.68
4500		-0.33	-0.43
11290		-0.33	0.60
5080		-0.33	-0.07
	4121	-0.33	-1.67
10521		-0.33	-0.29
	7171	-0.33	-1.34
13319		-0.33	0.37
6059		-0.33	-1.11
1522		-0.33	-0.42
12900		-0.33	0.61
31665		-0.33	-0.10

1966		-0.33	0.19
18741		-0.33	0.67
	11524	-0.33	0.35
13290		-0.33	
6054		-0.33	-1.02
32062		-0.33	-0.34
9455		-0.33	1.11
12161		-0.33	-0.07
7641		-0.33	-1.22
4144		-0.33	-1.37
14154		-0.33	-0.47
18255		-0.33	0.84
	5191	-0.33	0.36
17249		-0.33	-0.78
13873		-0.33	-0.46
13332		-0.33	0.64
15145		-0.33	-1.07
13700		-0.33	-1.23
9138		-0.33	0.51
13928		-0.33	-1.59
5581		-0.33	0.44
17282		-0.33	0.58
3225		-0.34	0.31
6570		-0.34	-0.28
6806		-0.34	-0.14
1782		-0.34	-0.63
11482		-0.34	-0.42
2750		-0.34	-1.50
9874		-0.34	-1.61
30018		-0.34	0.79
14930		-0.34	-0.55
18547		-0.34	-1.79
31268		-0.34	-1.72
14292		-0.34	-0.66
	16608	-0.34	1.09
11418		-0.34	-0.63
14577		-0.34	-0.21
9915		-0.34	-0.23
	4905	-0.34	0.59
10927		-0.34	-1.03
13142		-0.34	0.91
	9483	-0.34	-0.06
12896		-0.34	0.22
10086		-0.34	-0.73
31765		-0.34	0.86

12812		-0.34	0.77
	3927	-0.34	0.11
5052		-0.34	-0.09
4069		-0.34	-0.11
32369		-0.34	-0.92
32213		-0.34	0.28
18666		-0.34	-0.54
32665		-0.34	0.16
8661		-0.34	0.42
3770		-0.34	1.12
12027		-0.34	0.27
	15079	-0.34	0.84
1167		-0.34	0.34
	19860	-0.34	0.27
5808		-0.34	-0.29
15050		-0.34	-0.67
	3558	-0.34	0.43
32815		-0.34	-2.04
14659		-0.34	-0.51
4840		-0.34	-0.82
9884		-0.34	-0.72
8785		-0.34	-0.23
10802		-0.34	0.26
18596		-0.34	0.66
5028		-0.34	-1.50
2155		-0.34	-1.19
10462		-0.34	0.42
9999		-0.34	0.67
32436		-0.34	0.55
1359		-0.34	0.01
	6185	-0.34	-0.53
32795		-0.34	-0.52
5254		-0.34	0.35
7741		-0.34	-0.52
	6511	-0.34	0.28
	7393	-0.34	1.04
4998		-0.34	-0.34
	20086	-0.34	-0.61
3706		-0.34	-0.73
31243		-0.35	-1.70
30500		-0.35	0.19
14041		-0.35	0.08
11213		-0.35	-0.09
7135		-0.35	-0.87
15349		-0.35	-0.93

4413		-0.35	0.32
31473		-0.35	0.11
7223		-0.35	-1.35
18292		-0.35	0.44
8925		-0.35	-0.79
	10591	-0.35	0.10
	238	-0.35	-1.93
14414		-0.35	-0.67
16710		-0.35	-0.72
13902		-0.35	-0.12
9981		-0.35	0.78
5445		-0.35	-1.16
31038		-0.35	0.54
	4261	-0.35	-1.70
11217		-0.35	2.77
5226		-0.35	-0.53
5037		-0.35	-0.10
	13782	-0.35	0.12
13484		-0.35	-1.84
12428		-0.35	0.00
5217		-0.35	-1.36
9160		-0.35	-0.16
3598		-0.35	1.56
10306		-0.35	0.92
15812		-0.35	-0.31
7623		-0.35	0.53
9143		-0.35	0.71
	20158	-0.35	0.09
7434		-0.35	-0.37
13386		-0.35	1.03
4707		-0.35	-0.34
	17349	-0.35	-0.70
32262		-0.35	0.25
18676		-0.35	0.36
15711		-0.35	-1.00
6043		-0.35	-0.44
5205		-0.35	-1.12
15820		-0.35	0.48
	353	-0.35	0.17
13567		-0.35	-0.33
4790		-0.35	-0.64
6492		-0.35	0.81
4247		-0.35	-0.43
18005		-0.35	-0.12
15040		-0.35	-0.87

3493		-0.35	-1.36
12954		-0.35	-0.96
10165		-0.35	-1.98
10919		-0.35	-1.59
2229		-0.36	0.49
	20442	-0.36	-0.36
6696		-0.36	-0.33
5703		-0.36	-1.86
8050		-0.36	1.84
16825		-0.36	0.20
	20074	-0.36	-1.97
32042		-0.36	0.27
31743		-0.36	-0.42
9010		-0.36	-0.03
	7111	-0.36	-0.76
4677		-0.36	0.52
	11996	-0.36	0.00
1685		-0.36	-0.82
1967		-0.36	-1.34
14310		-0.36	0.39
9657		-0.36	-0.16
4038		-0.36	-0.59
3727		-0.36	0.19
8279		-0.36	0.55
32572		-0.36	-0.37
14919		-0.36	-1.31
9849		-0.36	-1.10
30053		-0.36	0.09
	5737	-0.36	-0.20
9162		-0.36	0.70
4379		-0.36	0.12
1776		-0.36	-0.98
1683		-0.36	-1.42
17083		-0.36	0.29
	1528	-0.36	0.39
32247		-0.36	-1.81
30039		-0.36	-0.74
15625		-0.36	0.24
17712		-0.36	-0.03
	15078	-0.36	-0.72
14356		-0.36	0.13
8323		-0.36	-0.42
33133		-0.36	-0.08
2691		-0.36	0.31
31928		-0.36	0.77

	3307	-0.36	-0.48
3967		-0.36	-0.85
	16610	-0.36	0.01
2968		-0.36	0.20
14903		-0.36	-1.01
32516		-0.36	0.57
	14453	-0.36	0.19
15113		-0.36	0.80
	3959	-0.36	0.91
14368		-0.36	0.37
10692		-0.36	-1.32
3966		-0.36	-1.07
32253		-0.36	-0.10
15526		-0.36	-0.80
3902		-0.36	0.29
11070		-0.36	0.95
32435		-0.36	0.32
30271		-0.36	-0.29
3985		-0.36	0.38
18327		-0.36	-0.37
10293		-0.36	-0.02
14650		-0.36	-0.73
8588		-0.36	-1.92
6846		-0.36	-0.23
31949		-0.36	-1.62
16711		-0.36	0.02
1071		-0.36	-1.14
	14384	-0.36	-2.25
7702		-0.36	-0.09
8465		-0.36	-1.24
33252		-0.36	0.26
10321		-0.36	-1.52
9200		-0.36	-1.28
18284		-0.36	-0.12
12007		-0.36	0.26
	1124	-0.36	0.25
	9735	-0.36	-0.10
10132		-0.36	-1.53
31300		-0.37	0.31
13251		-0.37	-1.17
8573		-0.37	-0.38
30165		-0.37	-0.21
	11480	-0.37	-0.20
6226		-0.37	0.35
17293		-0.37	-1.16

14631		-0.37	0.91
14648		-0.37	-0.48
8605		-0.37	0.30
13557		-0.37	-0.56
31730		-0.37	-0.03
8665		-0.37	-0.11
6715		-0.37	-0.31
	13962	-0.37	-1.34
17916		-0.37	-1.56
7557		-0.37	-0.02
10726		-0.37	-2.10
13489		-0.37	-0.43
11387		-0.37	-2.18
1696		-0.37	0.83
4655		-0.37	-0.56
7062		-0.37	1.31
32599		-0.37	0.30
	2996	-0.37	0.71
16986		-0.37	-1.15
32562		-0.37	0.39
4746		-0.37	-1.06
13723		-0.37	-0.20
	15194	-0.37	0.22
9703		-0.37	0.12
5747		-0.37	0.01
	19091	-0.37	-0.30
13003		-0.37	0.04
5287		-0.37	0.20
7398		-0.37	0.22
3881		-0.37	-0.96
8945		-0.37	-0.96
12715		-0.37	-0.02
	11104	-0.37	-0.65
9306		-0.37	0.40
3856		-0.37	-1.81
31468		-0.37	-0.24
11107		-0.37	-0.01
4717		-0.37	-0.60
31722		-0.37	-0.21
30082		-0.37	-0.78
	10416	-0.37	-0.17
2577		-0.37	0.66
32307		-0.37	0.40
13913		-0.37	0.10
9914		-0.37	0.00

4951		-0.37	0.13
18132		-0.37	0.22
6901		-0.37	0.08
8640		-0.37	0.63
6255		-0.37	0.77
2675		-0.37	0.41
	12957	-0.37	-2.00
17033		-0.38	-0.57
11211		-0.38	0.14
15651		-0.38	0.88
4894		-0.38	1.85
4330		-0.38	-0.02
31990		-0.38	-0.13
7604		-0.38	0.32
6953		-0.38	0.97
	4911	-0.38	0.80
	5702	-0.38	-1.03
	14293	-0.38	0.35
13203		-0.38	-1.46
	329	-0.38	-0.08
31140		-0.38	-0.63
	9009	-0.38	-0.30
	14632	-0.38	-1.30
30372		-0.38	-0.44
10881		-0.38	0.70
5024		-0.38	-0.81
7780		-0.38	0.24
9995		-0.38	0.29
	3672	-0.38	1.06
	19068	-0.38	-0.29
1716		-0.38	0.74
3108		-0.38	-0.64
32494		-0.38	-1.24
3754		-0.38	0.29
18519		-0.38	0.23
4573		-0.38	-0.31
13532		-0.38	0.49
12389		-0.38	-0.91
6224		-0.38	-1.62
	1052	-0.38	-0.89
7239		-0.38	-0.76
	11607	-0.38	-0.50
5872		-0.38	-0.65
14593		-0.38	-1.19
32355		-0.38	0.47

9212		-0.38	0.47
1791		-0.38	0.96
12015		-0.38	-0.53
8319		-0.38	0.56
9518		-0.38	-1.09
31329		-0.38	-0.41
4170		-0.38	0.27
9589		-0.38	-0.76
	3372	-0.38	0.61
15117		-0.38	1.09
2973		-0.38	0.86
11372		-0.38	-2.35
32048		-0.38	0.09
5738		-0.38	0.50
11413		-0.38	-0.77
	8783	-0.38	-0.09
1584		-0.38	-0.82
9403		-0.38	0.05
12446		-0.38	-0.37
6385		-0.38	-0.71
13654		-0.38	-1.25
13364		-0.38	-2.52
	16138	-0.38	0.84
2051		-0.39	0.36
5685		-0.39	-0.63
32252		-0.39	-1.53
12369		-0.39	0.50
8172		-0.39	-1.35
	9523	-0.39	1.10
7956		-0.39	0.30
8422		-0.39	0.69
	12671	-0.39	-0.64
8520		-0.39	-0.56
31007		-0.39	0.88
4972		-0.39	-0.39
1983		-0.39	0.06
16963		-0.39	0.31
9575		-0.39	0.24
16928		-0.39	-0.78
	18592	-0.39	1.34
4857		-0.39	0.43
18076		-0.39	-0.03
11533		-0.39	0.39
18802		-0.39	0.93
17824		-0.39	0.12

32669		-0.39	0.44
7421		-0.39	-0.63
5784		-0.39	0.25
	511	-0.39	-1.43
1208		-0.39	-0.23
13691		-0.39	-0.46
18744		-0.39	0.20
8825		-0.39	-2.06
8789		-0.39	-1.80
11537		-0.39	0.92
14372		-0.39	1.13
30097		-0.39	1.29
18023		-0.39	-0.85
	9237	-0.39	-1.36
5458		-0.39	0.43
8915		-0.39	-0.01
18271		-0.39	-2.90
12099		-0.39	-0.12
3688		-0.39	-1.29
5928		-0.39	0.10
12685		-0.39	-0.56
13510		-0.39	-0.47
6704		-0.39	-1.55
32730		-0.39	-0.08
11670		-0.39	-0.58
4212		-0.39	-0.76
32718		-0.39	-0.28
14227		-0.39	-0.35
	4208	-0.39	0.92
18258		-0.39	-0.61
17527		-0.39	0.14
2292		-0.39	-0.24
12721		-0.39	-1.45
	7401	-0.39	0.55
	7650	-0.39	0.18
	2763	-0.39	-1.09
10489		-0.39	0.07
7694		-0.39	-0.50
8834		-0.39	-0.37
4363		-0.39	-0.58
14022		-0.39	-0.89
14303		-0.39	-0.74
15307		-0.39	-1.61
15367		-0.39	-0.41
15322		-0.39	-2.11

5789		-0.39	-0.04
6361		-0.39	0.27
12125		-0.39	-0.32
30163		-0.39	-1.06
8506		-0.39	-0.55
3299		-0.39	0.28
	8290	-0.39	-2.08
4560		-0.39	0.27
	13416	-0.39	0.05
5246		-0.39	-0.58
5466		-0.40	0.61
7005		-0.40	-0.94
1149		-0.40	1.26
17559		-0.40	-0.46
	6390	-0.40	-0.88
	4154	-0.40	0.39
18661		-0.40	0.01
3342		-0.40	-0.06
18367		-0.40	-1.58
7435		-0.40	0.22
	2840	-0.40	-0.30
8119		-0.40	-0.13
15062		-0.40	-0.84
	19237	-0.40	-1.59
10343		-0.40	-0.69
8829		-0.40	0.46
	14089	-0.40	-2.06
32864		-0.40	1.10
3160		-0.40	-0.35
17370		-0.40	0.71
7452		-0.40	-0.05
4347		-0.40	0.06
18212		-0.40	0.12
5370		-0.40	-0.65
14630		-0.40	-1.06
9621		-0.40	-0.18
8241		-0.40	0.50
7017		-0.40	-1.43
11501		-0.40	-0.02
10506		-0.40	-0.89
17031		-0.40	-2.03
9118		-0.40	0.10
3480		-0.40	-1.33
30324		-0.40	0.34
	10616	-0.40	-0.48

15614		-0.40	-0.75
12140		-0.40	-0.09
12491		-0.40	
11639		-0.40	-0.02
10733		-0.40	0.18
6582		-0.40	-0.26
12531		-0.40	-1.48
8064		-0.40	-1.25
10909		-0.40	-0.29
	7966	-0.40	0.44
32454		-0.40	-1.69
2397		-0.40	0.21
7409		-0.40	-0.32
6727		-0.40	0.29
	19985	-0.40	-0.31
6698		-0.40	0.06
18673		-0.40	-0.75
	3088	-0.40	-0.03
30286		-0.40	0.76
30181		-0.40	0.91
7355		-0.40	-1.23
4123		-0.40	0.33
32616		-0.40	-0.52
5665		-0.40	-0.89
32873		-0.40	-0.59
17568		-0.40	-1.17
5268		-0.40	-1.24
7829		-0.40	-0.69
	15672	-0.41	0.16
	2850	-0.41	-0.10
32113		-0.41	-0.99
10345		-0.41	-0.04
	17749	-0.41	-1.19
4286		-0.41	-1.57
1389		-0.41	-0.98
32371		-0.41	-1.08
	427	-0.41	-1.88
9868		-0.41	-1.48
32771		-0.41	-1.08
	17643	-0.41	-0.50
8497		-0.41	0.08
2680		-0.41	-0.85
	11687	-0.41	0.77
12165		-0.41	-0.19
33268		-0.41	0.07

9192		-0.41	-0.29
2182		-0.41	-1.70
32689		-0.41	-0.42
	7396	-0.41	1.10
7037		-0.41	-1.52
12885		-0.41	-1.50
	2755	-0.41	-0.12
	18706	-0.41	-0.18
16876		-0.41	0.74
30447		-0.41	-0.20
11329		-0.41	-1.16
30193		-0.41	-1.15
	15717	-0.41	-1.29
3456		-0.41	1.91
10459		-0.41	0.35
	3533	-0.41	-0.51
	19613	-0.41	0.27
1064		-0.41	0.96
2253		-0.41	-0.81
8642		-0.41	-0.26
14809		-0.41	0.10
12009		-0.41	-0.65
8952		-0.41	0.76
15870		-0.41	-1.90
	11677	-0.41	0.91
5717		-0.41	0.38
8322		-0.41	0.21
3779		-0.41	1.31
	16224	-0.41	-0.23
4012		-0.41	-0.82
9985		-0.41	-0.23
9676		-0.41	-0.59
6434		-0.41	0.15
6105		-0.41	-0.68
31636		-0.41	0.25
1958		-0.41	-0.76
1851		-0.41	-0.55
30353		-0.41	0.01
14516		-0.41	-0.10
31041		-0.41	-0.27
5345		-0.41	-1.67
9092		-0.41	-0.87
3812		-0.41	0.87
31136		-0.41	2.66
9480		-0.41	1.48

4231		-0.41	-1.76
30154		-0.41	0.37
32490		-0.41	-0.57
8195		-0.42	-1.80
6231		-0.42	0.49
2774		-0.42	-0.76
10220		-0.42	-1.40
32706		-0.42	0.54
7320		-0.42	-1.82
	14233	-0.42	0.99
4854		-0.42	0.84
30354		-0.42	-0.28
7691		-0.42	-2.06
31368		-0.42	-0.38
33178		-0.42	-0.48
14593		-0.42	-1.52
7913		-0.42	-0.63
8175		-0.42	-2.54
5167		-0.42	-0.19
	8026	-0.42	-1.27
6222		-0.42	0.05
	17543	-0.42	0.12
1773		-0.42	0.43
	3899	-0.42	0.74
3350		-0.42	-0.92
	7384	-0.42	-0.01
5770		-0.42	0.87
10236		-0.42	-1.24
3703		-0.42	-1.85
15177		-0.42	0.45
5874		-0.42	-0.59
6139		-0.42	0.49
30071		-0.42	0.27
	9048	-0.42	0.17
9233		-0.42	-0.73
31818		-0.42	-0.41
	10777	-0.42	-0.23
7592		-0.42	-0.48
14153		-0.42	0.00
6770		-0.42	0.00
10514		-0.42	0.80
14182		-0.42	-0.11
31782		-0.42	0.58
31151		-0.42	-0.72
32396		-0.42	-1.27

13353		-0.42	0.25
	8751	-0.42	-0.85
2658		-0.42	-0.30
30152		-0.42	0.45
4007		-0.42	0.39
9656		-0.42	-0.11
6112		-0.42	0.62
4326		-0.42	-1.10
8484		-0.42	-0.10
	8027	-0.42	-0.73
6451		-0.42	-0.43
	17278	-0.42	0.14
13049		-0.42	0.23
8525		-0.42	0.16
31031		-0.42	-1.55
13541		-0.42	-0.94
14275		-0.42	-0.08
3283		-0.42	-0.59
16718		-0.42	-0.27
12752		-0.42	0.31
	16032	-0.42	0.63
2818		-0.42	-1.06
17286		-0.42	0.23
15059		-0.42	-0.44
30164		-0.42	-0.03
16762		-0.42	0.53
2246		-0.42	-1.69
8194		-0.42	-1.18
10275		-0.43	0.88
10362		-0.43	-1.22
7485		-0.43	-0.84
10528		-0.43	-0.85
1216		-0.43	0.00
9326		-0.43	0.42
	15624	-0.43	0.13
12121		-0.43	-1.12
9626		-0.43	-0.89
13504		-0.43	-1.27
	6432	-0.43	0.68
	7356	-0.43	0.01
3947		-0.43	-0.95
31067		-0.43	-0.68
9662		-0.43	1.41
18110		-0.43	-0.01
1433		-0.43	-1.06

	11673	-0.43	0.55
4580		-0.43	-0.47
3556		-0.43	-0.89
31415		-0.43	-0.85
	17523	-0.43	0.33
	20377	-0.43	0.83
12548		-0.43	-0.64
14898		-0.43	-0.67
33067		-0.43	-0.58
33278		-0.43	-0.12
9194		-0.43	0.53
8306		-0.43	0.34
14837		-0.43	-1.43
9484		-0.43	0.08
7833		-0.43	-0.48
17875		-0.43	0.03
7329		-0.43	-0.54
3446		-0.43	-0.82
14818		-0.43	-0.70
12519		-0.43	-0.99
14048		-0.43	-1.10
9615		-0.43	0.35
14362		-0.43	0.00
33129		-0.43	0.39
15744		-0.43	-0.16
9978		-0.43	-1.85
9357		-0.43	-1.49
14406		-0.43	0.72
	16170	-0.43	0.77
7012		-0.43	0.02
3792		-0.43	0.49
15332		-0.43	0.12
14477		-0.43	0.12
13226		-0.43	0.26
14325		-0.43	-0.23
31064		-0.43	-0.18
17911		-0.43	-0.26
	5447	-0.43	1.17
12253		-0.43	-0.75
17959		-0.43	0.72
14509		-0.43	0.38
30334		-0.43	1.07
5144		-0.43	0.55
10750		-0.43	0.24
32260		-0.43	0.75

	6277	-0.43	-0.03
3798		-0.43	0.52
13830		-0.43	0.18
31226		-0.43	0.15
13681		-0.43	-2.41
4400		-0.43	-1.85
9796		-0.43	0.48
8589		-0.43	0.16
4408		-0.43	0.10
	2683	-0.44	0.70
	15681	-0.44	-0.36
32063		-0.44	-0.50
7994		-0.44	-0.38
12543		-0.44	-1.95
10846		-0.44	0.41
14027		-0.44	-1.61
5663		-0.44	-1.00
	17488	-0.44	0.21
5976		-0.44	0.12
7333		-0.44	-0.39
4842		-0.44	-0.82
7351		-0.44	-1.81
32809		-0.44	-1.11
8454		-0.44	0.92
4280		-0.44	-0.39
31688		-0.44	-1.93
14377		-0.44	-0.73
8230		-0.44	-0.43
15350		-0.44	0.27
31541		-0.44	0.61
32117		-0.44	0.93
30145		-0.44	0.57
10357		-0.44	-1.45
4233		-0.44	-0.45
9294		-0.44	0.00
31931		-0.44	-0.21
10251		-0.44	1.68
30056		-0.44	0.76
14116		-0.44	0.71
10342		-0.44	-1.54
15506		-0.44	-0.64
33138		-0.44	0.09
33184		-0.44	0.69
9308		-0.44	0.19
3722		-0.44	-1.58

15603		-0.44	-0.81
9555		-0.44	-0.44
32276		-0.44	-1.24
6394		-0.44	-1.79
18231		-0.44	-1.09
3443		-0.44	0.14
	14312	-0.44	-1.71
3903		-0.44	-0.88
11397		-0.44	-0.90
13872		-0.44	-0.60
5884		-0.44	-0.33
4656		-0.44	-1.65
4922		-0.44	-1.54
11804		-0.44	-0.89
11001		-0.44	-1.51
	382	-0.44	0.21
	16686	-0.44	-0.01
	115	-0.44	0.10
11314		-0.44	0.30
8080		-0.44	0.64
12991		-0.44	-0.31
1469		-0.44	-0.60
14779		-0.44	0.54
3651		-0.44	-1.20
17584		-0.44	0.00
4370		-0.44	-0.60
13085		-0.44	-1.73
9128		-0.44	-0.10
13927		-0.44	-0.17
	15129	-0.44	-1.33
	18555	-0.44	0.19
6093		-0.44	-1.39
14876		-0.44	-0.15
1435		-0.44	-1.73
4704		-0.44	-0.09
5326		-0.44	2.15
2252		-0.44	-0.23
30047		-0.44	0.46
	11834	-0.44	0.32
	10495	-0.44	0.59
	12867	-0.44	-1.33
10809		-0.44	0.30
32736		-0.44	-0.45
8780		-0.44	-1.24
13966		-0.44	0.50

5161		-0.44	0.16
2611		-0.44	0.26
15929		-0.44	-0.44
3719		-0.44	-0.05
31069		-0.44	-0.53
10035		-0.45	-0.92
4742		-0.45	-0.41
6550		-0.45	-1.00
15785		-0.45	-0.20
10473		-0.45	0.52
15548		-0.45	-1.33
15200		-0.45	-0.95
	9797	-0.45	-0.42
4022		-0.45	0.24
17697		-0.45	-0.24
9573		-0.45	0.71
	13251	-0.45	-1.49
1360		-0.45	-0.10
7224		-0.45	-0.91
17739		-0.45	1.63
14882		-0.45	-0.22
5423		-0.45	-1.83
15161		-0.45	0.42
7653		-0.45	-0.75
33177		-0.45	-0.78
12370		-0.45	-0.86
13408		-0.45	0.95
12879		-0.45	0.10
6895		-0.45	0.39
31923		-0.45	-0.04
	9857	-0.45	0.56
7313		-0.45	-0.19
4674		-0.45	-0.11
13232		-0.45	0.72
	19530	-0.45	0.42
9491		-0.45	-0.79
32313		-0.45	-0.20
	7104	-0.45	0.54
9059		-0.45	-0.90
15072		-0.45	-0.18
6418		-0.45	-0.68
14591		-0.45	-1.45
1084		-0.45	1.15
32649		-0.45	-0.85
1849		-0.45	-0.80

1467		-0.45	0.18
31235		-0.45	-1.48
13162		-0.45	-1.90
	17498	-0.45	-0.32
18780		-0.45	-1.78
9903		-0.45	-1.15
	8089	-0.45	-0.70
2848		-0.45	-0.36
3380		-0.45	-1.30
4542		-0.45	0.56
13481		-0.45	1.04
31199		-0.45	0.47
5213		-0.45	-0.76
9250		-0.45	0.92
8237		-0.45	0.24
8734		-0.45	-0.07
11769		-0.46	0.52
13425		-0.46	0.21
4365		-0.46	0.53
12343		-0.46	0.85
13436		-0.46	-0.08
30272		-0.46	-1.01
14842		-0.46	-1.17
	18254	-0.46	0.49
14036		-0.46	-0.28
	7609	-0.46	-1.13
	18255	-0.46	0.26
11159		-0.46	-1.04
32261		-0.46	-1.87
7415		-0.46	0.54
3424		-0.46	-0.92
7481		-0.46	-0.77
14077		-0.46	-0.14
	2658	-0.46	0.95
1840		-0.46	-0.69
31860		-0.46	-0.74
	15448	-0.46	-0.33
3371		-0.46	-0.36
31183		-0.46	-0.37
32022		-0.46	-0.43
	6759	-0.46	-1.96
14668		-0.46	-0.75
18179		-0.46	-0.60
9298		-0.46	-1.98
2059		-0.46	0.24

6216		-0.46	-0.41
4311		-0.46	1.04
1024		-0.46	0.66
11019		-0.46	-0.03
7779		-0.46	0.08
10274		-0.46	0.00
4676		-0.46	-0.18
1386		-0.46	-0.10
32092		-0.46	-0.09
16735		-0.46	1.79
33503		-0.46	-1.06
15456		-0.46	-1.18
	14099	-0.46	0.39
	5522	-0.46	-0.74
	13704	-0.46	-1.77
14233		-0.46	-2.33
5077		-0.46	-0.71
6355		-0.46	-1.01
14210		-0.46	-0.12
17742		-0.46	-0.22
30033		-0.46	-1.85
9670		-0.46	0.98
2898		-0.46	-0.04
4882		-0.46	0.33
13788		-0.46	-0.10
12895		-0.46	-0.35
1143		-0.46	0.68
6073		-0.46	-0.39
7465		-0.46	0.76
2262		-0.46	-0.56
17956		-0.46	0.00
1288		-0.46	0.33
7735		-0.46	-1.41
11253		-0.46	-0.24
	2233	-0.46	-1.44
	9080	-0.47	0.01
10298		-0.47	0.19
13955		-0.47	-0.55
8146		-0.47	0.49
14956		-0.47	0.18
17107		-0.47	0.22
11857		-0.47	-0.11
	1267	-0.47	0.20
7900		-0.47	0.28
10855		-0.47	0.13

11313		-0.47	-0.87
7175		-0.47	-1.31
9485		-0.47	-1.91
8738		-0.47	0.30
8458		-0.47	0.49
12314		-0.47	-0.86
3977		-0.47	0.60
12995		-0.47	-0.46
13159		-0.47	-0.90
12565		-0.47	-0.42
	11210	-0.47	-2.39
31003		-0.47	-2.22
18582		-0.47	-0.77
11020		-0.47	0.68
14841		-0.47	0.17
8284		-0.47	0.56
1889		-0.47	0.51
15000		-0.47	-0.02
	11837	-0.47	1.41
2827		-0.47	0.68
	19938	-0.47	-2.20
2903		-0.47	-0.15
	10016	-0.47	-1.26
5320		-0.47	-0.78
	10337	-0.47	0.61
10737		-0.47	-0.81
32186		-0.47	-0.30
6182		-0.47	1.11
7331		-0.47	-1.29
6954		-0.47	0.48
	8856	-0.47	0.06
	2699	-0.47	-0.55
6657		-0.47	-1.01
14852		-0.47	5.36
3983		-0.47	0.89
30020		-0.47	-0.15
	8756	-0.47	0.76
13222		-0.47	0.44
13760		-0.47	-0.56
12171		-0.47	0.37
6905		-0.47	-1.06
3540		-0.47	-0.71
6659		-0.47	-0.56
3379		-0.47	-1.05
	12402	-0.47	0.74

31230		-0.47	-0.38
6049		-0.47	-0.83
7429		-0.47	-1.59
7378		-0.47	0.67
	19160	-0.47	-1.20
8145		-0.47	0.63
3827		-0.47	-1.98
8297		-0.47	0.10
11068		-0.47	1.19
3501		-0.47	-1.35
2184		-0.48	-0.04
1213		-0.48	-0.34
16858		-0.48	-0.09
14615		-0.48	0.05
6822		-0.48	0.56
8617		-0.48	0.22
10987		-0.48	0.40
2146		-0.48	0.37
8028		-0.48	-0.24
12944		-0.48	1.46
	12634	-0.48	0.16
	5859	-0.48	-0.50
12187		-0.48	0.59
	8411	-0.48	-0.52
12252		-0.48	-0.47
9741		-0.48	0.04
3358		-0.48	0.17
5612		-0.48	-0.52
5528		-0.48	-1.21
13807		-0.48	0.72
31093		-0.48	-0.79
5790		-0.48	-0.46
30419		-0.48	0.03
14071		-0.48	-1.80
13324		-0.48	0.56
14719		-0.48	-0.54
13651		-0.48	0.45
31436		-0.48	-0.38
8234		-0.48	-1.52
32709		-0.48	1.39
15259		-0.48	-1.02
1751		-0.48	0.53
11325		-0.48	-0.21
	11190	-0.48	-0.28
	15604	-0.48	0.47

13929		-0.48	-1.65
5955		-0.48	0.92
14353		-0.48	-0.77
8257		-0.48	-0.48
13308		-0.48	1.16
	19859	-0.48	0.56
1442		-0.48	-0.73
32162		-0.48	-0.33
2921		-0.48	0.22
10730		-0.48	-1.09
11619		-0.48	-1.56
30183		-0.48	-1.18
10124		-0.48	-1.66
12933		-0.48	-0.39
15534		-0.48	-1.71
31105		-0.48	0.49
15803		-0.48	0.13
14453		-0.48	-0.47
11071		-0.48	0.74
4236		-0.48	-0.65
	15274	-0.48	0.44
11139		-0.48	0.24
13018		-0.48	0.20
5758		-0.48	0.20
14315		-0.48	-0.15
4911		-0.48	-1.07
13956		-0.48	-0.47
11693		-0.48	-0.32
	20112	-0.48	-0.80
11622		-0.49	-0.77
13599		-0.49	-1.69
12660		-0.49	-0.52
17065		-0.49	-0.63
5347		-0.49	-1.36
33458		-0.49	0.13
4364		-0.49	-0.23
15047		-0.49	-1.01
1989		-0.49	-1.11
10637		-0.49	0.83
31259		-0.49	0.28
31698		-0.49	0.26
	7670	-0.49	0.55
8312		-0.49	-0.78
	10624	-0.49	-1.34
	13906	-0.49	0.10

6004		-0.49	-0.06
14996		-0.49	-1.43
	6265	-0.49	1.85
15556		-0.49	0.47
4871		-0.49	-0.83
7359		-0.49	-0.78
5810		-0.49	-0.43
2209		-0.49	-1.12
13985		-0.49	0.43
6551		-0.49	-1.04
7328		-0.49	-1.03
9072		-0.49	-1.13
	2919	-0.49	1.60
6660		-0.49	0.24
10233		-0.49	-0.23
31178		-0.49	-1.67
	13515	-0.49	-0.39
14447		-0.49	0.03
	10007	-0.49	-2.24
31460		-0.49	0.21
8974		-0.49	-0.82
1775		-0.49	-0.24
	3720	-0.49	0.31
	18593	-0.49	0.25
6896		-0.49	-0.04
16889		-0.49	-1.16
13307		-0.49	0.25
3495		-0.49	-1.43
16791		-0.49	2.71
2943		-0.49	0.12
14513		-0.49	-1.73
33235		-0.49	-0.73
8182		-0.49	-0.64
13989		-0.49	-2.26
33038		-0.49	-0.42
	8888	-0.49	0.52
3206		-0.49	-0.40
2939		-0.49	-0.11
14781		-0.49	0.20
	12552	-0.49	-0.31
1925		-0.49	0.96
	14964	-0.49	1.06
2698		-0.49	0.09
3756		-0.49	0.80
5304		-0.49	-1.71

	12794	-0.49	-2.02
8302		-0.50	0.65
7376		-0.50	-0.20
2508		-0.50	-0.16
1615		-0.50	-1.06
	12553	-0.50	0.62
32280		-0.50	0.68
33455		-0.50	-1.88
18374		-0.50	-1.12
8660		-0.50	0.84
8613		-0.50	0.64
6538		-0.50	1.12
6679		-0.50	-0.16
7662		-0.50	-0.70
9430		-0.50	-1.74
15286		-0.50	-0.19
32151		-0.50	-0.80
14354		-0.50	-0.39
8402		-0.50	-1.30
13204		-0.50	-2.57
13492		-0.50	-0.94
6574		-0.50	-1.34
6511		-0.50	0.80
13908		-0.50	-0.53
30290		-0.50	-2.14
9114		-0.50	0.09
3200		-0.50	-0.64
1750		-0.50	0.59
18377		-0.50	0.70
10095		-0.50	-1.15
	7437	-0.50	-0.11
16752		-0.50	1.15
5517		-0.50	0.04
9700		-0.50	-0.25
8918		-0.50	-0.35
9970		-0.50	-0.95
11924		-0.50	0.88
8282		-0.50	1.00
6613		-0.50	-0.86
31633		-0.50	0.14
11550		-0.50	0.78
17388		-0.50	0.01
13322		-0.50	0.23
1362		-0.50	-0.10
6148		-0.50	-0.32

6179		-0.50	1.19
18477		-0.50	0.23
3573		-0.50	-0.90
31906		-0.50	-1.43
	19057	-0.50	-0.63
31682		-0.50	1.67
12605		-0.50	-2.33
18278		-0.50	-0.63
6695		-0.50	-0.11
8791		-0.50	0.89
	16559	-0.50	0.17
12512		-0.50	0.95
18180		-0.50	-0.47
3696		-0.50	-0.64
14430		-0.50	-0.21
30361		-0.50	0.53
6428		-0.50	0.85
4675		-0.50	-1.26
10209		-0.50	-1.96
6524		-0.50	1.16
1046		-0.50	-0.12
12676		-0.50	0.04
10601		-0.50	-0.79
	4273	-0.51	-1.34
1695		-0.51	0.27
	5479	-0.51	-0.11
	8173	-0.51	-0.57
13202		-0.51	-0.31
18746		-0.51	0.40
16944		-0.51	0.99
3353		-0.51	-1.05
	7883	-0.51	0.46
	1518	-0.51	1.62
32560		-0.51	0.25
10650		-0.51	-0.32
	17188	-0.51	0.88
	11892	-0.51	-0.21
14317		-0.51	-0.06
11342		-0.51	-1.90
17998		-0.51	0.12
8460		-0.51	1.04
	20081	-0.51	0.10
18143		-0.51	-1.86
	8810	-0.51	0.77
13022		-0.51	-0.55

10706		-0.51	-2.75
9507		-0.51	-0.59
	4256	-0.51	-0.98
14944		-0.51	-0.30
	8921	-0.51	0.91
2147		-0.51	-0.33
31055		-0.51	-0.19
	9503	-0.51	1.43
2446		-0.51	-0.19
15216		-0.51	0.48
2304		-0.51	-0.65
9804		-0.51	-0.43
	12053	-0.51	-1.12
32849		-0.51	1.22
14112		-0.51	-0.32
5147		-0.51	1.12
15017		-0.51	-0.07
12340		-0.51	-2.20
5174		-0.51	-0.90
9610		-0.51	-0.59
1850		-0.51	-0.25
33468		-0.51	-1.32
11168		-0.51	-0.73
32224		-0.51	-2.19
10106		-0.51	0.98
2052		-0.51	-0.05
15019		-0.51	0.09
31453		-0.51	0.46
17802		-0.51	-1.40
7981		-0.51	0.04
15071		-0.51	-1.82
13098		-0.51	-2.36
17962		-0.51	0.47
15890		-0.51	-1.43
8110		-0.51	-0.94
5711		-0.51	-0.65
2185		-0.51	0.95
6560		-0.51	-0.39
8526		-0.51	-0.18
6296		-0.51	0.31
3056		-0.51	0.01
9361		-0.52	-0.05
7335		-0.52	-0.14
4238		-0.52	-0.57
	20380	-0.52	-0.08

13829		-0.52	-0.49
17760		-0.52	-0.43
7246		-0.52	-0.14
4104		-0.52	-1.06
	14962	-0.52	-0.50
3021		-0.52	-0.23
31555		-0.52	-0.85
32251		-0.52	-0.89
32404		-0.52	-0.45
	15077	-0.52	-1.23
6293		-0.52	0.42
5524		-0.52	-1.16
3655		-0.52	-0.28
	12004	-0.52	0.16
1643		-0.52	0.23
	754	-0.52	-0.86
8680		-0.52	-0.53
7354		-0.52	-0.87
5495		-0.52	-0.20
5424		-0.52	-0.98
7551		-0.52	-0.11
33283		-0.52	0.11
7720		-0.52	-0.78
11238		-0.52	-0.94
33464		-0.52	-0.03
31240		-0.52	-0.25
4324		-0.52	0.00
2031		-0.52	-0.56
9907		-0.52	0.12
14189		-0.52	-0.50
11008		-0.52	0.64
17840		-0.52	0.37
11388		-0.52	-1.61
32239		-0.52	-1.33
10033		-0.52	-0.09
14431		-0.52	-1.29
3987		-0.52	-0.03
10318		-0.52	-1.10
	7783	-0.52	0.07
	14905	-0.52	1.24
30429		-0.52	-0.72
16932		-0.52	-0.41
	18662	-0.52	-1.21
8370		-0.52	-0.57
31177		-0.52	0.53

6829		-0.52	-0.60
12945		-0.52	-0.88
11593		-0.52	0.05
7095		-0.52	-0.35
15150		-0.52	-0.83
31431		-0.52	0.00
13424		-0.52	0.20
32635		-0.52	1.26
3169		-0.52	-1.29
11138		-0.52	-0.08
11154		-0.52	-1.48
	14351	-0.52	-0.27
1090		-0.52	-0.60
	9994	-0.52	-0.36
5201		-0.52	-0.38
14624		-0.52	0.78
12522		-0.52	0.49
7449		-0.52	-0.24
5701		-0.52	-1.05
3588		-0.53	-2.25
15533		-0.53	-0.59
11156		-0.53	-1.63
3149		-0.53	-0.48
3339		-0.53	-0.74
32409		-0.53	-1.76
13904		-0.53	-0.19
15330		-0.53	0.00
5125		-0.53	-0.80
11438		-0.53	0.11
7129		-0.53	0.70
6533		-0.53	0.97
	4205	-0.53	-0.26
	14588	-0.53	-0.25
9780		-0.53	0.30
	4984	-0.53	0.18
	10897	-0.53	0.11
14905		-0.53	-0.59
30373		-0.53	-0.43
3192		-0.53	-0.34
1634		-0.53	-1.83
14017		-0.53	0.33
17732		-0.53	-1.04
	9160	-0.53	-0.17
17085		-0.53	0.08
12502		-0.53	-0.18

5522		-0.53	-0.36
8448		-0.53	-0.48
4006		-0.53	-0.19
10603		-0.53	0.37
	7169	-0.53	0.18
7512		-0.53	-0.69
8507		-0.53	-0.41
32683		-0.53	-0.31
4180		-0.53	0.14
	16272	-0.53	-0.16
6052		-0.53	-0.28
4620		-0.53	-0.77
10472		-0.53	-0.09
1558		-0.53	-0.48
33202		-0.53	-0.05
	2357	-0.53	-2.26
10540		-0.53	-1.24
2238		-0.53	3.24
8607		-0.53	0.24
12650		-0.53	-1.21
11655		-0.53	-2.00
31426		-0.53	0.10
11588		-0.53	-0.45
	16641	-0.53	-1.75
4016		-0.53	-1.16
13999		-0.53	0.20
8631		-0.53	-1.01
15312		-0.54	-1.55
14826		-0.54	0.15
	66	-0.54	-1.18
	14533	-0.54	-0.56
14318		-0.54	-0.16
14893		-0.54	-0.42
7271		-0.54	-0.79
7722		-0.54	-0.49
15811		-0.54	-0.92
5878		-0.54	-0.78
10563		-0.54	-1.49
7470		-0.54	-0.85
2662		-0.54	-0.72
17828		-0.54	-2.28
5029		-0.54	-0.35
3376		-0.54	-0.59
14228		-0.54	
32440		-0.54	-0.27

6563		-0.54	-0.92
	5797	-0.54	0.48
13881		-0.54	-0.26
5490		-0.54	1.21
10841		-0.54	0.35
6405		-0.54	0.07
10701		-0.54	0.18
6879		-0.54	1.22
8584		-0.54	0.67
4375		-0.54	0.47
	9874	-0.54	-1.24
15585		-0.54	-0.61
4444		-0.54	0.01
4533		-0.54	-1.23
30122		-0.54	-0.61
	13360	-0.54	0.54
12682		-0.54	-1.83
7659		-0.54	-1.89
	14472	-0.54	-0.39
13165		-0.54	-0.27
12003		-0.54	-0.28
11101		-0.54	0.40
	10917	-0.54	-1.32
8372		-0.54	-0.86
6599		-0.54	0.21
6814		-0.54	-1.51
17352		-0.54	-0.75
15696		-0.54	0.64
13549		-0.54	-1.78
6844		-0.54	-1.70
16788		-0.54	1.32
14179		-0.55	-0.47
13561		-0.55	1.07
	15105	-0.55	-0.34
32114		-0.55	0.86
15281		-0.55	-1.61
11166		-0.55	0.35
3318		-0.55	-1.57
9559		-0.55	-0.95
7510		-0.55	-0.80
31322		-0.55	-0.95
	18883	-0.55	-0.61
33469		-0.55	-1.50
15404		-0.55	0.28
4702		-0.55	-1.07

4907		-0.55	1.17
12600		-0.55	-0.48
	12224	-0.55	0.96
31668		-0.55	-0.77
9702		-0.55	1.28
8027		-0.55	1.13
7306		-0.55	0.37
3781		-0.55	0.07
5828		-0.55	0.86
31615		-0.55	1.74
5500		-0.55	0.11
6547		-0.55	0.83
33115		-0.55	-1.84
10034		-0.55	-0.82
	5690	-0.55	1.01
10174		-0.55	0.21
4556		-0.55	-1.01
5303		-0.55	0.34
	20139	-0.55	0.20
5265		-0.55	-1.01
12426		-0.55	-0.94
13836		-0.55	0.34
14232		-0.55	1.29
16826		-0.55	1.84
10007		-0.55	-0.38
1528		-0.55	1.99
4600		-0.55	-0.66
17667		-0.55	0.13
31142		-0.55	-1.18
9280		-0.55	-0.41
14306		-0.55	-1.02
15844		-0.55	0.40
4763		-0.55	-0.57
11370		-0.55	-0.41
11377		-0.55	0.50
18681		-0.55	-0.18
2685		-0.55	0.00
4039		-0.55	-0.15
18859		-0.55	-2.55
14088		-0.55	-1.06
30037		-0.55	-0.25
1314		-0.55	-1.65
12737		-0.56	0.33
3909		-0.56	-1.43
6692		-0.56	-0.58

8705		-0.56	-0.93
31646		-0.56	-0.30
32704		-0.56	0.02
6576		-0.56	-1.73
9334		-0.56	-0.68
13231		-0.56	-0.62
	19986	-0.56	-0.18
4509		-0.56	-0.07
2871		-0.56	-1.49
17375		-0.56	0.08
31365		-0.56	-0.41
9156		-0.56	1.79
6873		-0.56	0.46
	9977	-0.56	-2.05
10460		-0.56	0.94
	14219	-0.56	1.25
6176		-0.56	-1.48
	11615	-0.56	-0.31
9867		-0.56	-0.44
3758		-0.56	0.40
3290		-0.56	-0.66
1086		-0.56	0.40
	9233	-0.56	-2.49
11808		-0.56	-0.21
5589		-0.56	0.08
9184		-0.56	-0.74
31036		-0.56	-1.04
1607		-0.56	0.14
18646		-0.56	-1.71
11051		-0.56	-0.31
14960		-0.56	0.61
	9383	-0.56	-0.87
	7758	-0.56	0.21
10978		-0.56	-2.22
6234		-0.56	-1.10
6206		-0.56	-1.83
10635		-0.56	0.64
10973		-0.56	0.13
17059		-0.56	-1.61
14086		-0.56	0.61
9855		-0.56	-1.45
14501		-0.56	-1.22
31392		-0.56	-1.32
31239		-0.56	-0.33
16747		-0.56	-0.23

7695		-0.56	-0.52
1877		-0.56	-1.37
15262		-0.56	0.57
	3974	-0.56	0.26
10609		-0.56	-1.54
9689		-0.56	-2.18
	12330	-0.56	-0.92
12135		-0.56	0.07
33344		-0.56	-0.65
14762		-0.56	-0.69
13833		-0.56	-1.49
14865		-0.56	-0.84
3105		-0.56	-0.47
	13998	-0.56	-1.96
6737		-0.56	-0.87
13678		-0.57	-1.12
5706		-0.57	-1.42
	16566	-0.57	-0.83
6276		-0.57	0.32
9927		-0.57	-1.63
33233		-0.57	-0.46
4385		-0.57	0.13
7464		-0.57	-1.23
6985		-0.57	0.00
15705		-0.57	0.03
31152		-0.57	0.07
11776		-0.57	0.43
11876		-0.57	-1.70
33274		-0.57	-1.01
6131		-0.57	0.24
	14405	-0.57	-0.10
6641		-0.57	-1.70
32109		-0.57	-1.73
6742		-0.57	-1.02
2173		-0.57	-0.05
8425		-0.57	1.73
9414		-0.57	-0.22
3409		-0.57	0.38
	9532	-0.57	0.45
11833		-0.57	-1.01
10794		-0.57	0.59
14001		-0.57	-0.79
7509		-0.57	-1.37
5131		-0.57	0.42
3081		-0.57	0.55

3047		-0.57	-0.22
	20058	-0.57	-1.37
13113		-0.57	-0.98
8538		-0.57	-1.04
6883		-0.57	-1.34
12452		-0.57	0.80
33075		-0.57	
2358		-0.57	-0.43
11630		-0.57	-0.26
7987		-0.57	-0.40
	13999	-0.57	-2.44
10297		-0.57	0.76
14526		-0.57	-1.14
6097		-0.57	-1.13
5070		-0.57	0.00
5847		-0.57	1.32
3222		-0.57	-1.37
	9451	-0.57	1.08
9351		-0.57	-2.43
14808		-0.57	-0.95
9431		-0.57	-1.18
31681		-0.57	-0.39
9922		-0.58	0.27
32657		-0.58	-2.30
15536		-0.58	1.36
13157		-0.58	-0.26
32050		-0.58	-1.73
5280		-0.58	0.07
	20136	-0.58	-1.96
6404		-0.58	-0.15
10047		-0.58	-0.46
	10559	-0.58	0.50
1715		-0.58	0.70
7048		-0.58	1.28
14753		-0.58	-0.96
2341		-0.58	0.23
	19028	-0.58	-1.50
31457		-0.58	-1.62
13220		-0.58	0.17
7794		-0.58	-0.42
8853		-0.58	0.95
9799		-0.58	0.12
13926		-0.58	0.36
5876		-0.58	-0.44
5996		-0.58	1.53

	6652	-0.58	-0.62
8592		-0.58	0.40
32006		-0.58	0.00
7737		-0.58	-0.27
31175		-0.58	0.24
	6615	-0.58	-0.52
13686		-0.58	-0.93
32154		-0.58	-0.72
3499		-0.58	-1.40
3158		-0.58	-1.56
	14228	-0.58	0.63
14290		-0.58	-0.57
7025		-0.58	0.09
12863		-0.58	0.80
33008		-0.58	-1.97
6866		-0.58	-1.81
8137		-0.58	0.99
32648		-0.58	-1.30
15525		-0.58	0.64
9288		-0.58	1.32
12499		-0.58	0.89
12785		-0.58	0.58
13402		-0.58	-1.36
15204		-0.58	-1.27
4438		-0.58	-0.95
2948		-0.58	-1.05
33196		-0.58	-0.07
12689		-0.58	-1.94
7874		-0.58	0.25
14016		-0.58	-1.89
9692		-0.58	-1.25
1669		-0.58	-0.83
6036		-0.59	1.55
17187		-0.59	-0.60
6754		-0.59	0.72
8104		-0.59	0.80
	9756	-0.59	-0.44
10470		-0.59	-0.61
16853		-0.59	-0.32
11698		-0.59	-0.24
5079		-0.59	-1.68
	13350	-0.59	-1.09
15604		-0.59	-0.69
	1782	-0.59	-0.41
17724		-0.59	-0.18

31845		-0.59	-1.88
32475		-0.59	-0.23
1134		-0.59	-1.87
	628	-0.59	-2.22
17349		-0.59	-0.07
17032		-0.59	-0.43
30073		-0.59	-0.43
18769		-0.59	-1.77
6083		-0.59	-1.38
6264		-0.59	-0.54
	3682	-0.59	0.71
4360		-0.59	0.30
31339		-0.59	0.89
32052		-0.59	-0.57
3561		-0.59	0.51
5327		-0.59	0.01
12853		-0.59	-1.91
6490		-0.59	1.12
	7492	-0.59	-1.49
	9253	-0.59	-1.71
11052		-0.59	-0.78
	8895	-0.59	0.63
	5823	-0.59	-0.37
11737		-0.59	-0.97
15721		-0.59	1.75
8380		-0.59	-1.54
32576		-0.59	0.05
6388		-0.59	-1.33
33479		-0.59	-0.27
6534		-0.59	-0.82
5341		-0.59	-1.12
11847		-0.59	-1.34
32121		-0.59	0.02
6793		-0.59	-1.52
32982		-0.59	-0.14
8932		-0.59	1.23
32158		-0.59	-0.99
9882		-0.59	-1.03
7296		-0.59	-2.14
11129		-0.59	-1.44
4033		-0.59	-1.16
	9102	-0.59	-0.54
5994		-0.59	0.84
15067		-0.59	0.24
	12882	-0.59	-0.63

8144		-0.59	1.46
4051		-0.59	-0.75
4199		-0.59	0.54
9652		-0.59	0.65
11601		-0.59	0.83
8729		-0.59	-1.63
7529		-0.59	-0.95
14821		-0.59	-1.36
32249		-0.59	-0.11
15127		-0.59	-0.76
32044		-0.59	-1.32
16904		-0.59	0.49
9267		-0.59	0.06
10778		-0.59	-1.90
14147		-0.59	-0.09
	2355	-0.60	-1.34
7428		-0.60	-0.31
31224		-0.60	-0.41
5055		-0.60	-0.68
	2016	-0.60	0.05
18417		-0.60	0.44
1066		-0.60	-0.74
3961		-0.60	1.09
7523		-0.60	0.02
	901	-0.60	-1.02
1546		-0.60	-0.12
	17458	-0.60	-1.47
14260		-0.60	-0.41
	6747	-0.60	-0.87
10553		-0.60	-0.33
31355		-0.60	-0.94
6440		-0.60	-0.01
6297		-0.60	-0.31
2684		-0.60	-0.62
7352		-0.60	-1.23
4278		-0.60	-0.58
18313		-0.60	-0.59
32332		-0.60	-0.62
4459		-0.60	-0.04
17081		-0.60	-0.41
14424		-0.60	0.76
1647		-0.60	-0.57
5863		-0.60	0.16
	13174	-0.60	0.30
32614		-0.60	0.18

6359		-0.60	0.12
31391		-0.60	-0.93
11099		-0.60	0.25
8051		-0.60	0.14
	9884	-0.60	-0.51
10426		-0.60	-0.31
	6015	-0.60	-0.81
11009		-0.60	1.14
32205		-0.60	1.07
	11680	-0.60	-0.17
7039		-0.60	-1.12
5762		-0.60	0.06
8221		-0.60	0.00
5059		-0.60	0.93
	7438	-0.60	-0.52
17002		-0.60	-0.46
10000		-0.60	0.02
12304		-0.60	-0.83
	13417	-0.60	0.00
9342		-0.60	-0.65
13687		-0.60	0.15
6074		-0.60	-0.76
15280		-0.60	-0.11
10152		-0.60	-0.80
31160		-0.60	-0.86
9244		-0.60	-0.96
10951		-0.60	-1.20
15080		-0.60	0.86
30343		-0.60	-1.02
	9872	-0.60	-1.00
14444		-0.60	0.32
15566		-0.60	-0.80
	20406	-0.60	-0.39
4551		-0.60	-0.87
17110		-0.60	0.19
13518		-0.60	0.31
3171		-0.60	0.42
2221		-0.60	-0.07
	14963	-0.60	1.39
6851		-0.60	-0.03
6990		-0.60	1.07
14735		-0.61	-1.62
1819		-0.61	-0.77
4067		-0.61	-1.67
7886		-0.61	0.26

31097		-0.61	-1.57
31960		-0.61	0.58
	9753	-0.61	-0.27
31759		-0.61	0.76
6689		-0.61	-1.44
9242		-0.61	-0.54
32077		-0.61	-0.57
11133		-0.61	-1.58
12765		-0.61	-0.27
8630		-0.61	-0.34
3692		-0.61	-0.78
31145		-0.61	0.29
	17713	-0.61	-0.50
3532		-0.61	0.21
13708		-0.61	0.19
18173		-0.61	-0.57
5767		-0.61	0.04
	4643	-0.61	-0.98
6824		-0.61	0.38
16724		-0.61	0.53
3743		-0.61	0.57
9198		-0.61	-0.91
18255		-0.61	-0.09
31330		-0.61	0.15
2837		-0.61	-0.14
6693		-0.61	0.10
13870		-0.61	-0.02
13650		-0.61	-1.46
	3055	-0.61	-1.23
18766		-0.61	0.55
32485		-0.61	0.16
	18992	-0.61	-0.58
1944		-0.61	-0.52
15186		-0.61	1.24
15311		-0.61	-0.79
7813		-0.61	0.14
32776		-0.61	0.66
2025		-0.61	-0.83
6889		-0.61	-0.21
9362		-0.61	0.76
	13468	-0.61	0.20
8383		-0.61	-0.26
15476		-0.61	-1.11
14813		-0.61	-1.10
	5854	-0.61	0.71

31035		-0.61	-0.52
14856		-0.61	-0.44
1147		-0.61	0.12
6027		-0.61	-0.18
9164		-0.61	0.98
31517		-0.61	-0.98
31306		-0.61	0.67
31364		-0.61	0.15
13333		-0.61	-0.51
6677		-0.61	-0.17
14860		-0.61	0.96
8206		-0.61	-0.76
6816		-0.61	-1.45
11522		-0.61	-0.92
	2826	-0.61	0.27
32284		-0.61	-0.58
10173		-0.61	0.82
	6381	-0.61	-0.16
3309		-0.62	-1.16
5429		-0.62	0.14
16749		-0.62	0.60
	421	-0.62	-1.46
2204		-0.62	-1.63
31247		-0.62	0.14
3382		-0.62	-0.45
8566		-0.62	-0.01
4641		-0.62	1.45
7453		-0.62	0.24
15543		-0.62	-0.55
31368		-0.62	-0.99
32094		-0.62	-1.17
4705		-0.62	-0.43
5183		-0.62	0.19
2849		-0.62	-0.43
17777		-0.62	-1.63
	11897	-0.62	-0.05
	17050	-0.62	0.23
	16013	-0.62	0.68
15598		-0.62	-0.79
13467		-0.62	-0.22
7616		-0.62	-0.90
3244		-0.62	-1.61
6190		-0.62	-0.66
	6177	-0.62	-1.98
30410		-0.62	-1.55

8783		-0.62	-0.26
12950		-0.62	-0.47
6053		-0.62	-0.37
18324		-0.62	-1.29
9036		-0.62	0.01
3511		-0.62	-1.03
10269		-0.62	-0.72
3172		-0.62	-1.44
13491		-0.62	-1.46
31137		-0.62	-0.99
31285		-0.62	-0.23
5104		-0.62	-1.02
8196		-0.62	-0.41
30369		-0.62	-1.07
6542		-0.62	0.10
	13423	-0.62	-1.50
9240		-0.62	0.19
17261		-0.62	-0.72
	6620	-0.62	-0.30
8299		-0.62	0.17
8959		-0.62	-0.68
	3067	-0.62	-0.02
30391		-0.62	-0.69
31301		-0.62	0.26
1418		-0.62	-0.58
	2792	-0.62	0.68
6090		-0.62	-1.74
33476		-0.62	1.40
9195		-0.62	-1.28
7084		-0.62	0.48
	8957	-0.62	-0.01
	16344	-0.62	1.23
11328		-0.62	-0.45
30420		-0.62	0.33
7777		-0.62	0.02
18682		-0.62	-2.10
	4576	-0.63	0.23
18607		-0.63	-2.26
13704		-0.63	-0.28
	9186	-0.63	-1.07
6484		-0.63	-1.07
11552		-0.63	-0.91
3797		-0.63	1.75
32595		-0.63	-0.59
5267		-0.63	-0.25

13369		-0.63	0.15
3354		-0.63	1.56
13380		-0.63	-0.68
18604		-0.63	-0.32
13551		-0.63	-2.59
31139		-0.63	0.50
32354		-0.63	2.24
9775		-0.63	-0.66
12084		-0.63	-0.99
13455		-0.63	-1.72
2692		-0.63	1.45
32081		-0.63	-1.12
9317		-0.63	-0.11
14877		-0.63	0.59
15031		-0.63	-1.86
17045		-0.63	0.15
	15599	-0.63	-0.25
11778		-0.63	-0.41
17267		-0.63	1.10
30171		-0.63	-0.40
	13343	-0.63	-0.26
	5153	-0.63	1.13
2189		-0.63	1.08
4139		-0.63	-1.31
18331		-0.63	0.62
31753		-0.63	-1.00
3135		-0.63	-0.47
4999		-0.63	0.41
2095		-0.63	-0.05
15879		-0.63	-1.55
3494		-0.63	-1.32
31650		-0.63	0.73
17383		-0.63	-1.87
8742		-0.63	-0.16
5670		-0.63	-1.91
	11841	-0.63	-0.37
8155		-0.63	-0.08
31735		-0.63	-1.28
10822		-0.63	-0.81
	11022	-0.63	0.01
9654		-0.63	-0.60
6154		-0.63	0.78
17801		-0.63	-0.54
12924		-0.63	-0.48
11922		-0.63	0.04

3845		-0.63	0.55
	12357	-0.63	0.27
9318		-0.63	-1.78
10038		-0.63	-0.90
10801		-0.63	-0.50
5405		-0.63	-1.02
14352		-0.63	0.28
	15159	-0.63	-0.72
	4177	-0.63	0.40
5023		-0.63	-0.47
8846		-0.63	-1.48
31719		-0.63	-0.69
17180		-0.63	0.29
10571		-0.63	1.88
6512		-0.63	0.43
13531		-0.63	-0.63
30322		-0.63	-1.87
30035		-0.64	-0.34
32437		-0.64	-0.44
8492		-0.64	-1.05
3717		-0.64	-0.96
32790		-0.64	0.18
4802		-0.64	0.22
15664		-0.64	0.00
5714		-0.64	-1.17
	8529	-0.64	1.67
4755		-0.64	-0.02
1142		-0.64	0.02
10183		-0.64	-0.40
12265		-0.64	0.06
9263		-0.64	-0.39
9929		-0.64	-0.50
12123		-0.64	-0.53
1232		-0.64	1.01
4013		-0.64	-1.21
11905		-0.64	0.85
8001		-0.64	-2.40
31848		-0.64	-0.24
11921		-0.64	-0.59
4449		-0.64	-0.18
	18325	-0.64	-0.08
4025		-0.64	0.42
30186		-0.64	-1.38
11856		-0.64	-0.18
	5696	-0.64	0.97

15220		-0.64	0.61
15279		-0.64	-0.10
7506		-0.64	-0.97
10283		-0.64	-0.45
4865		-0.64	0.92
15152		-0.64	0.05
10422		-0.64	-0.52
32413		-0.64	-1.12
17931		-0.64	-0.55
30051		-0.64	-0.98
11877		-0.64	-0.15
3592		-0.64	0.28
14803		-0.64	-0.37
1374		-0.64	0.31
9282		-0.64	0.79
31972		-0.64	-0.73
5507		-0.64	-0.30
30091		-0.64	-1.18
32067		-0.64	-1.48
5655		-0.64	0.30
1844		-0.64	-0.63
6888		-0.64	-2.56
14507		-0.65	1.40
32189		-0.65	-0.11
31229		-0.65	-0.91
31314		-0.65	-0.01
17268		-0.65	-1.40
12455		-0.65	-0.66
	15523	-0.65	-0.69
14793		-0.65	-0.24
15661		-0.65	-0.19
12541		-0.65	-0.53
	12031	-0.65	-0.52
	14467	-0.65	0.46
15306		-0.65	0.01
	11348	-0.65	-2.35
8412		-0.65	-0.27
5440		-0.65	-0.35
13835		-0.65	0.39
	12547	-0.65	-0.23
	14369	-0.65	1.15
7298		-0.65	-1.32
15327		-0.65	-1.37
32165		-0.65	-1.25
6356		-0.65	-1.10

8626		-0.65	-0.46
3058		-0.65	-1.20
	4385	-0.65	0.47
32627		-0.65	-0.04
2151		-0.65	-0.27
6472		-0.65	-0.23
33099		-0.65	0.18
	5668	-0.65	1.36
13698		-0.65	-1.05
	16139	-0.65	0.56
31775		-0.65	0.62
7101		-0.65	0.15
1263		-0.65	-3.98
6854		-0.65	0.41
11196		-0.65	-0.44
13064		-0.65	0.14
7000		-0.65	1.19
14687		-0.65	-1.18
12413		-0.65	0.29
	15712	-0.65	-0.59
4565		-0.65	-0.72
8444		-0.65	0.16
30422		-0.65	0.33
11113		-0.65	-0.51
30103		-0.65	-0.48
1401		-0.65	-0.01
18731		-0.65	-0.31
8889		-0.65	0.82
8295		-0.65	-0.50
	7869	-0.66	-0.51
14806		-0.66	-0.30
14866		-0.66	0.01
16838		-0.66	-0.87
2679		-0.66	-1.04
13589		-0.66	-0.62
	14558	-0.66	-2.62
15173		-0.66	0.83
6371		-0.66	-0.53
18408		-0.66	-1.30
1169		-0.66	-0.72
	17261	-0.66	-0.48
8161		-0.66	0.22
12011		-0.66	-0.26
5014		-0.66	-0.93
	20370	-0.66	-1.52

16743		-0.66	-0.20
31112		-0.66	-1.44
4698		-0.66	0.12
9339		-0.66	0.08
7809		-0.66	-0.82
2961		-0.66	-1.45
11638		-0.66	-1.29
32266		-0.66	-2.02
	12945	-0.66	-0.90
4795		-0.66	-1.04
	11086	-0.66	-0.74
6992		-0.66	0.94
6843		-0.66	-1.67
32484		-0.66	0.23
7882		-0.66	-0.50
30000		-0.66	-0.88
8277		-0.66	0.14
15287		-0.66	-1.22
11299		-0.66	0.12
	11681	-0.66	0.57
2360		-0.66	-1.58
7152		-0.66	-0.36
6986		-0.66	0.00
12218		-0.66	-1.73
7154		-0.66	-1.32
	9385	-0.66	-0.87
12683		-0.66	-0.21
12081		-0.66	-0.80
18314		-0.66	-0.10
11771		-0.66	-1.25
4994		-0.66	-1.84
	2698	-0.66	0.36
	15267	-0.66	0.07
3258		-0.66	-1.92
	20117	-0.66	-2.16
	19987	-0.67	-0.03
3355		-0.67	-0.59
5652		-0.67	-1.09
30344		-0.67	0.14
	8084	-0.67	0.59
8890		-0.67	1.17
14958		-0.67	-0.41
32533		-0.67	-0.17
9613		-0.67	-0.23
	1993	-0.67	-1.06

30219		-0.67	-0.09
15515		-0.67	0.16
	9864	-0.67	0.25
31121		-0.67	-0.80
5197		-0.67	-1.36
6443		-0.67	-0.77
5422		-0.67	-0.09
11966		-0.67	-1.05
8073		-0.67	0.60
4154		-0.67	-0.22
8515		-0.67	-0.35
14166		-0.67	-2.11
18107		-0.67	-1.30
31710		-0.67	-1.84
8913		-0.67	0.67
	5575	-0.67	-1.90
16708		-0.67	-2.00
14239		-0.67	-1.28
13108		-0.67	-0.31
15514		-0.67	-0.36
	14215	-0.67	-1.47
	2219	-0.67	-1.75
14868		-0.67	-1.20
11404		-0.67	-1.02
13282		-0.67	1.07
14456		-0.67	-1.99
6850		-0.67	0.16
3473		-0.67	0.83
7345		-0.67	-0.42
4481		-0.67	0.31
5565		-0.67	-0.39
	15041	-0.67	-1.43
5916		-0.67	-0.19
12093		-0.67	-0.69
15579		-0.67	-0.03
7265		-0.67	0.25
6631		-0.67	-0.11
6071		-0.67	-0.75
13827		-0.67	0.23
13025		-0.68	-0.70
31664		-0.68	-1.88
1702		-0.68	-1.09
32074		-0.68	-1.31
3038		-0.68	-0.63
31439		-0.68	-1.59

12667		-0.68	-1.08
	11903	-0.68	-0.76
12408		-0.68	0.71
1343		-0.68	-1.10
	3390	-0.68	-1.17
3132		-0.68	-0.27
32972		-0.68	-0.22
6539		-0.68	1.08
11033		-0.68	-0.81
17994		-0.68	0.65
2128		-0.68	-0.46
32194		-0.68	-1.57
12333		-0.68	0.17
11910		-0.68	-0.08
14264		-0.68	-2.12
	12759	-0.68	0.05
14026		-0.68	-1.00
6713		-0.68	0.23
6902		-0.68	0.61
4435		-0.68	-0.72
12795		-0.68	-0.08
10830		-0.68	-0.01
10803		-0.68	-1.60
2981		-0.68	-0.69
14966		-0.68	-1.36
8301		-0.68	0.43
8892		-0.68	0.58
9509		-0.68	-0.16
10222		-0.68	-0.89
31172		-0.68	0.19
31519		-0.68	0.19
7923		-0.68	-0.06
3433		-0.68	-0.29
	13939	-0.68	-1.14
	2211	-0.68	-1.70
4386		-0.68	-1.78
12190		-0.68	-0.50
3085		-0.68	-0.61
12636		-0.68	0.39
	5063	-0.68	-1.75
12442		-0.68	0.96
	20284	-0.68	-2.17
7213		-0.68	0.03
1969		-0.68	0.52
5150		-0.68	-0.93

17278		-0.68	-1.58
2241		-0.68	-0.47
8787		-0.68	-0.70
30486		-0.68	-1.45
12546		-0.68	-1.35
1630		-0.68	0.49
2947		-0.69	-1.24
17047		-0.69	-1.19
6703		-0.69	-0.62
6639		-0.69	0.84
	7491	-0.69	0.08
14413		-0.69	-1.12
	2005	-0.69	-1.20
	8017	-0.69	-2.40
13965		-0.69	0.03
4962		-0.69	-0.23
33103		-0.69	0.41
2772		-0.69	-0.95
18136		-0.69	0.70
12911		-0.69	0.57
18228		-0.69	0.34
5515		-0.69	-1.26
13641		-0.69	0.19
	7105	-0.69	-0.51
6403		-0.69	0.84
6690		-0.69	-1.56
4973		-0.69	-0.56
32143		-0.69	-1.30
7083		-0.69	0.11
6342		-0.69	0.55
2989		-0.69	-0.45
11138		-0.69	-0.61
3625		-0.69	0.77
31612		-0.69	-0.15
4501		-0.69	-0.21
15153		-0.69	-1.39
	20403	-0.69	-1.94
10062		-0.69	-1.44
	9632	-0.69	0.13
12006		-0.69	0.15
18217		-0.69	-0.22
31870		-0.69	-0.23
3174		-0.69	-0.85
14156		-0.69	-0.20
13964		-0.69	-0.15

4105		-0.69	-0.88
8075		-0.69	1.23
6596		-0.69	-1.07
	5997	-0.69	-1.08
30110		-0.69	0.01
16820		-0.69	-1.33
4678		-0.69	0.17
3611		-0.69	-0.34
13050		-0.70	-0.16
12104		-0.70	0.08
	20382	-0.70	-0.01
12977		-0.70	1.00
30501		-0.70	-0.02
2666		-0.70	-0.51
31169		-0.70	-0.99
33048		-0.70	-0.27
6210		-0.70	-0.70
11454		-0.70	-0.40
32803		-0.70	-0.95
6020		-0.70	-0.07
4596		-0.70	-1.04
13040		-0.70	-1.97
12385		-0.70	-0.09
15304		-0.70	-0.90
15623		-0.70	-1.09
12662		-0.70	-0.14
6867		-0.70	-1.87
2196		-0.70	0.60
8914		-0.70	0.77
	9972	-0.70	-1.20
9451		-0.70	-1.25
	8750	-0.70	0.34
32727		-0.70	0.28
3896		-0.70	0.91
	20085	-0.70	-0.27
11495		-0.70	0.23
3929		-0.70	-0.95
13120		-0.70	-2.38
30293		-0.70	0.29
7530		-0.70	-0.17
4686		-0.70	-0.09
31852		-0.70	1.59
3884		-0.70	0.35
9035		-0.70	-0.37
	8350	-0.70	-1.87

9776		-0.70	-0.64
7436		-0.70	-0.54
31015		-0.70	-0.66
10623		-0.70	-0.57
9287		-0.70	1.00
3918		-0.70	-0.23
30327		-0.70	0.69
11199		-0.70	-0.40
2457		-0.70	0.34
33117		-0.70	0.01
5733		-0.70	-0.27
7665		-0.70	0.49
10718		-0.70	0.04
12692		-0.70	-0.03
31312		-0.70	-1.30
6254		-0.71	0.01
15510		-0.71	-0.46
1965		-0.71	-0.49
14266		-0.71	1.12
4738		-0.71	-1.09
32850		-0.71	0.26
8636		-0.71	2.51
13747		-0.71	-1.32
5860		-0.71	0.02
1120		-0.71	-0.33
	12211	-0.71	0.30
6119		-0.71	0.63
11642		-0.71	-0.74
14752		-0.71	-1.06
31344		-0.71	-0.82
32569		-0.71	-0.16
4875		-0.71	-1.31
15358		-0.71	-1.75
2004		-0.71	0.52
8987		-0.71	-0.06
13785		-0.71	0.02
16777		-0.71	-0.85
7600		-0.71	-1.27
1912		-0.71	-1.05
1633		-0.71	-0.90
32190		-0.71	0.72
17567		-0.71	-0.69
9619		-0.71	0.84
31989		-0.71	-2.02
9050		-0.71	0.53

15251		-0.71	-0.41
7826		-0.71	-1.18
10175		-0.71	0.02
5553		-0.71	0.18
	16353	-0.71	-0.44
32387		-0.71	0.87
3324		-0.71	-1.09
31022		-0.71	0.27
15024		-0.71	-1.93
4532		-0.71	-0.74
17524		-0.71	-1.38
10110		-0.71	0.32
32098		-0.71	0.39
3736		-0.71	0.26
9885		-0.71	-0.94
7798		-0.71	0.28
3997		-0.71	-0.06
31176		-0.72	-0.47
9246		-0.72	-0.59
9148		-0.72	-0.07
14137		-0.72	-0.03
31478		-0.72	-0.21
	10905	-0.72	0.86
31400		-0.72	-1.45
2969		-0.72	-0.21
15376		-0.72	0.78
14840		-0.72	0.74
	5285	-0.72	-0.14
4068		-0.72	-1.39
2707		-0.72	0.81
9004		-0.72	-1.54
32243		-0.72	-0.69
10952		-0.72	-1.62
	14217	-0.72	-2.02
15249		-0.72	-0.89
15522		-0.72	-2.65
	20121	-0.72	-1.31
	20070	-0.72	-0.51
3606		-0.72	-0.25
12851		-0.72	-0.35
13393		-0.72	-0.09
	326	-0.72	-0.11
7152		-0.72	-0.61
12526		-0.72	-0.88
	16183	-0.72	1.22

18478		-0.72	0.68
31388		-0.72	0.24
14101		-0.72	0.54
4570		-0.72	-1.31
1409		-0.72	-0.22
7528		-0.72	-1.37
	15083	-0.72	0.23
31217		-0.72	0.14
8004		-0.72	-0.61
3850		-0.72	-0.10
11881		-0.72	-0.72
12927		-0.72	-0.28
4457		-0.72	-0.47
31193		-0.72	0.74
	5132	-0.72	-2.09
13786		-0.72	-0.99
4226		-0.72	-0.19
31227		-0.72	-0.31
8916		-0.72	-0.03
4062		-0.72	0.27
9698		-0.72	-0.92
9057		-0.72	-1.60
13361		-0.72	1.01
10208		-0.72	0.11
7057		-0.72	-0.87
14234		-0.73	-2.25
5705		-0.73	-1.07
32721		-0.73	-2.26
7896		-0.73	-1.31
10823		-0.73	0.53
17005		-0.73	0.29
	7870	-0.73	0.18
14346		-0.73	0.30
14329		-0.73	-0.15
	18488	-0.73	-0.39
13569		-0.73	0.05
14053		-0.73	0.16
14608		-0.73	-0.27
4562		-0.73	0.15
	6760	-0.73	-1.67
6445		-0.73	-1.19
33136		-0.73	-0.04
2982		-0.73	0.01
1960		-0.73	-0.29
4785		-0.73	-1.30

	17990	-0.73	0.41
15464		-0.73	-1.95
12330		-0.73	0.71
13331		-0.73	-1.89
4688		-0.73	-1.47
15817		-0.73	0.14
11153		-0.73	-1.54
1211		-0.73	-0.03
31917		-0.73	-0.02
32438		-0.73	-0.17
6478		-0.73	0.26
17743		-0.73	0.68
16888		-0.73	-1.44
3697		-0.73	-0.77
9977		-0.73	0.06
4587		-0.73	-1.30
4021		-0.73	0.45
11898		-0.73	-1.57
	6006	-0.73	-2.72
	5850	-0.73	0.03
4302		-0.73	-0.82
4268		-0.73	0.06
8564		-0.73	0.30
9568		-0.73	0.49
	8757	-0.73	0.56
	6628	-0.73	-0.49
	7619	-0.73	-0.63
	2913	-0.73	0.22
15917		-0.73	-0.84
4804		-0.73	-1.02
13790		-0.73	0.20
	6498	-0.73	0.93
9449		-0.73	-1.55
	19715	-0.73	-0.53
	20062	-0.73	-0.70
32295		-0.74	-0.89
10912		-0.74	-0.23
17498		-0.74	0.51
	5827	-0.74	-0.88
2723		-0.74	-1.02
15219		-0.74	-1.12
9046		-0.74	-0.82
	4622	-0.74	0.32
14847		-0.74	-1.61
	9490	-0.74	-1.56

10931		-0.74	-1.11
7841		-0.74	-0.23
9144		-0.74	0.53
7404		-0.74	0.10
32302		-0.74	-1.01
4004		-0.74	-1.61
	20433	-0.74	-1.98
32685		-0.74	-0.07
16884		-0.74	-1.12
7781		-0.74	-0.17
8840		-0.74	0.19
3876		-0.74	-0.53
14247		-0.74	1.46
6246		-0.74	-0.92
8553		-0.74	-0.38
4867		-0.74	-1.14
	2911	-0.74	-0.13
31318		-0.74	0.44
5192		-0.74	-0.91
31508		-0.74	0.09
31291		-0.74	-0.39
10181		-0.74	0.15
15663		-0.74	-0.52
13855		-0.74	0.45
8174		-0.74	-0.14
2990		-0.74	-0.52
	9867	-0.74	-0.58
6094		-0.74	0.21
3647		-0.74	-0.29
3117		-0.74	-0.42
6038		-0.74	-0.64
11703		-0.74	-1.17
14572		-0.74	-1.64
4561		-0.74	-1.36
6386		-0.74	-1.17
1179		-0.74	-1.09
	3057	-0.74	-0.64
15225		-0.74	-0.27
6791		-0.74	-0.09
16765		-0.74	-0.53
32675		-0.74	-0.03
6201		-0.74	-0.89
14720		-0.74	0.13
	20020	-0.74	0.74
7954		-0.74	-0.20

	12052	-0.74	-2.13
9264		-0.74	1.13
4042		-0.74	-0.40
15154		-0.74	-0.55
33249		-0.75	-1.33
2681		-0.75	-0.28
1911		-0.75	-0.86
15660		-0.75	0.14
14941		-0.75	-2.09
6568		-0.75	-0.11
7161		-0.75	-0.76
15747		-0.75	-0.14
	11505	-0.75	0.59
10622		-0.75	-0.45
10063		-0.75	-0.07
	14395	-0.75	0.03
3536		-0.75	0.38
32540		-0.75	-0.83
31501		-0.75	-1.86
15136		-0.75	-1.11
31686		-0.75	-1.43
3898		-0.75	-1.00
4930		-0.75	-0.28
5288		-0.75	-0.43
9494		-0.75	-1.33
10478		-0.75	0.18
31867		-0.75	-0.37
31675		-0.75	-1.08
5934		-0.75	-0.07
8929		-0.75	-1.29
15142		-0.75	-0.53
17361		-0.75	-0.13
15380		-0.75	-2.06
	7475	-0.75	0.43
	12359	-0.75	0.19
1151		-0.75	-0.26
5337		-0.75	0.51
31800		-0.75	-2.21
18223		-0.75	-1.50
2046		-0.75	-0.06
31811		-0.75	0.09
10121		-0.75	0.03
13260		-0.75	-0.76
	16125	-0.75	1.67
	8875	-0.75	-0.75

14764		-0.75	-2.59
17906		-0.75	0.04
6114		-0.75	-0.78
10570		-0.75	-1.11
31874		-0.75	0.85
14805		-0.75	-1.37
5811		-0.75	-0.29
	14378	-0.75	0.28
3373		-0.75	-0.30
5887		-0.75	-0.71
16960		-0.75	-0.73
	15428	-0.75	-1.35
3086		-0.75	-0.66
31117		-0.76	-0.46
16761		-0.76	0.44
7852		-0.76	-0.11
	13492	-0.76	-0.44
	6418	-0.76	0.80
7507		-0.76	-0.56
11449		-0.76	-1.61
12245		-0.76	-0.83
10864		-0.76	0.77
17736		-0.76	-1.91
8476		-0.76	-0.73
	2642	-0.76	0.88
30295		-0.76	-1.51
14253		-0.76	0.02
9168		-0.76	-0.40
6223		-0.76	0.09
8070		-0.76	-0.05
	9179	-0.76	-0.29
1461		-0.76	-1.57
11152		-0.76	-1.67
11765		-0.76	-0.28
	7075	-0.76	-0.18
12360		-0.76	-0.07
12256		-0.76	-0.82
14419		-0.76	-0.42
14270		-0.76	-0.34
3114		-0.76	-0.22
7761		-0.76	-0.15
15022		-0.76	-0.04
17129		-0.76	-0.25
15897		-0.76	-1.38
14666		-0.76	0.89

32315		-0.76	0.64
32654		-0.76	0.77
1697		-0.76	0.79
7650		-0.76	0.10
4680		-0.76	-0.61
6721		-0.76	-1.64
33106		-0.76	0.05
9172		-0.76	1.50
3763		-0.76	-0.81
5369		-0.76	-0.97
3587		-0.76	-0.57
6527		-0.76	0.20
2263		-0.76	-1.15
	19052	-0.76	-0.27
12189		-0.76	-0.25
14446		-0.76	-0.81
32169		-0.77	0.38
8186		-0.77	-1.50
3839		-0.77	-1.51
10911		-0.77	-0.39
14327		-0.77	0.74
31761		-0.77	0.79
1101		-0.77	1.07
13479		-0.77	-2.67
1639		-0.77	-0.34
2655		-0.77	-0.90
4959		-0.77	-1.60
7967		-0.77	-0.75
3847		-0.77	0.41
16700		-0.77	-1.32
9592		-0.77	-1.36
16758		-0.77	0.64
13534		-0.77	-1.03
11489		-0.77	-1.94
10724		-0.77	-1.42
9998		-0.77	0.14
5435		-0.77	-1.24
12403		-0.77	0.21
3880		-0.77	-0.77
12155		-0.77	-1.62
18627		-0.77	-0.52
	9002	-0.77	-0.65
5867		-0.77	0.04
2109		-0.77	-0.67
6383		-0.77	-0.61

33484		-0.77	-0.11
4825		-0.77	-1.19
10373		-0.77	-1.72
17216		-0.77	-1.84
33228		-0.77	-2.47
15520		-0.77	-1.36
9471		-0.77	-0.57
10722		-0.77	0.64
3159		-0.77	-1.17
9677		-0.77	-0.41
31612		-0.77	-0.44
3599		-0.77	1.45
16834		-0.77	-0.28
7853		-0.77	-0.21
	13914	-0.77	-0.63
17574		-0.77	-0.01
1505		-0.77	-0.84
	7868	-0.77	-0.67
	10550	-0.77	1.02
	12014	-0.78	-0.47
9262		-0.78	0.40
1847		-0.78	-0.99
5050		-0.78	-1.24
14870		-0.78	-0.15
	11908	-0.78	-1.09
33111		-0.78	-0.52
9067		-0.78	-0.42
31784		-0.78	0.06
7686		-0.78	-1.93
1770		-0.78	0.70
11147		-0.78	-0.33
4535		-0.78	-1.08
31524		-0.78	2.36
5383		-0.78	-1.14
4891		-0.78	-1.21
7816		-0.78	-0.21
3203		-0.78	-0.14
9413		-0.78	-0.73
17905		-0.78	-1.81
14033		-0.78	-1.71
2049		-0.78	0.73
	14380	-0.78	-0.46
9243		-0.78	-1.23
2910		-0.78	0.00
5361		-0.78	-0.94

33330		-0.78	2.67
8587		-0.78	-0.23
2412		-0.78	-1.25
12063		-0.78	-0.84
	18673	-0.78	-0.48
7571		-0.78	-0.74
3675		-0.78	-1.21
17753		-0.78	-1.59
32944		-0.78	-1.30
10059		-0.78	-1.16
14145		-0.79	-0.71
31319		-0.79	-1.05
6638		-0.79	0.15
31076		-0.79	0.19
2808		-0.79	-1.16
3924		-0.79	0.91
3249		-0.79	-0.20
	4650	-0.79	0.08
5687		-0.79	-1.21
14115		-0.79	0.03
16886		-0.79	-1.19
9596		-0.79	-0.57
3218		-0.79	0.11
8315		-0.79	0.02
15188		-0.79	0.41
15018		-0.79	-0.47
	11030	-0.79	-1.85
12908		-0.79	0.58
7630		-0.79	-1.54
17438		-0.79	-1.28
4858		-0.79	-0.99
3245		-0.79	-0.87
6205		-0.79	-1.91
4190		-0.79	-1.79
11178		-0.79	-1.62
10002		-0.79	-0.55
13008		-0.79	0.21
8408		-0.79	0.14
5910		-0.79	0.03
32586		-0.79	-0.72
32912		-0.79	-1.29
3604		-0.79	0.39
8461		-0.79	-0.02
15016		-0.79	-0.61
5408		-0.79	-0.50

15012		-0.79	0.89
1815		-0.79	0.01
10946		-0.79	-1.59
14523		-0.79	-0.85
14274		-0.79	0.52
	6000	-0.79	-2.10
	4628	-0.79	-1.20
30010		-0.79	0.13
13326		-0.79	-0.54
5202		-0.79	-0.88
5013		-0.79	-1.80
17319		-0.79	-0.36
11654		-0.79	-1.49
7685		-0.79	-1.23
14478		-0.79	-0.35
4757		-0.79	0.65
5078		-0.79	-0.90
3025		-0.79	-0.11
8147		-0.79	-0.24
7739		-0.80	-0.93
8156		-0.80	-0.10
	16039	-0.80	0.84
7879		-0.80	-1.13
10751		-0.80	-0.56
8569		-0.80	0.75
5998		-0.80	0.00
1091		-0.80	-1.08
32827		-0.80	-0.45
9222		-0.80	-0.13
17011		-0.80	0.03
7706		-0.80	-1.14
15258		-0.80	0.64
1794		-0.80	-0.09
11581		-0.80	-0.50
32527		-0.80	-0.84
15592		-0.80	-1.41
3252		-0.80	0.76
32801		-0.80	-1.30
8157		-0.80	-1.30
	5855	-0.80	-0.83
8177		-0.80	-0.07
9175		-0.80	0.56
10122		-0.80	-1.06
3955		-0.80	0.74
32551		-0.80	-0.99

9620		-0.80	0.00
11134		-0.80	-1.07
9886		-0.80	2.88
7372		-0.80	-1.80
8602		-0.80	1.47
3092		-0.80	-1.49
1180		-0.80	-1.44
7081		-0.80	-1.35
9372		-0.80	0.18
16908		-0.80	1.16
15507		-0.80	-1.05
31924		-0.81	0.10
	11746	-0.81	0.13
	19992	-0.81	0.09
2807		-0.81	-0.37
1999		-0.81	0.04
15635		-0.81	0.59
15368		-0.81	-2.16
4313		-0.81	-0.36
4604		-0.81	-1.13
4984		-0.81	-0.98
	5999	-0.81	-1.45
30381		-0.81	-1.38
10071		-0.81	-0.73
4325		-0.81	-0.19
9576		-0.81	2.13
13517		-0.81	1.27
14607		-0.81	-0.09
8902		-0.81	-0.42
15671		-0.81	-0.96
18131		-0.81	-0.02
31273		-0.81	-1.62
5371		-0.81	-1.10
5491		-0.81	-0.42
10084		-0.81	-0.58
16956		-0.81	-1.44
15654		-0.81	-0.08
2198		-0.81	0.29
7918		-0.81	-1.03
11590		-0.81	-0.99
30267		-0.81	1.28
30072		-0.81	-1.50
12931		-0.81	-0.72
13920		-0.81	-0.89
12858		-0.81	0.08

8612		-0.81	1.07
3496		-0.81	-0.59
7156		-0.81	-0.18
13980		-0.81	-1.22
8663		-0.81	-0.05
17726		-0.81	0.33
11734		-0.82	-1.10
4976		-0.82	-0.24
12822		-0.82	0.89
14460		-0.82	-0.99
9176		-0.82	0.64
12717		-0.82	-0.02
	17265	-0.82	-1.32
9115		-0.82	-1.95
9401		-0.82	1.64
	13589	-0.82	0.09
	9875	-0.82	-0.73
10858		-0.82	-1.64
31053		-0.82	0.77
	13853	-0.82	-0.17
17223		-0.82	0.23
17009		-0.82	-0.94
2014		-0.82	-0.31
1666		-0.82	-0.98
	5001	-0.82	-0.09
13427		-0.82	-0.36
5127		-0.82	-0.32
8638		-0.82	0.45
12983		-0.82	0.27
9701		-0.82	-1.90
30336		-0.82	-0.47
3107		-0.82	-1.26
4193		-0.82	0.02
31488		-0.82	0.16
15035		-0.82	-0.40
7227		-0.82	0.68
5637		-0.82	-0.04
	11479	-0.82	0.27
	12051	-0.82	-1.98
9005		-0.82	-0.41
8624		-0.82	0.01
5643		-0.82	0.78
	9801	-0.82	0.07
30171		-0.82	0.74
3445		-0.82	-0.20

	10933	-0.83	0.04
3090		-0.83	-0.23
	8015	-0.83	-1.08
1893		-0.83	-2.10
12393		-0.83	0.11
7183		-0.83	-0.17
8135		-0.83	0.95
2368		-0.83	-0.62
33322		-0.83	0.05
4995		-0.83	-1.23
3626		-0.83	0.32
13042		-0.83	-1.53
5709		-0.83	-1.15
	548	-0.83	-2.08
15535		-0.83	0.06
	16058	-0.83	0.07
33231		-0.83	0.55
31935		-0.83	-1.83
2519		-0.83	-1.78
	8087	-0.83	-0.19
15247		-0.83	-0.15
1137		-0.83	0.04
13901		-0.83	-0.40
33213		-0.83	-1.76
31683		-0.83	1.41
1135		-0.83	-1.56
	18607	-0.83	-0.40
	9053	-0.83	-1.68
11076		-0.83	-0.23
12241		-0.83	-1.10
31531		-0.83	0.61
31921		-0.83	0.47
4173		-0.83	0.02
14118		-0.83	-0.86
13991		-0.83	-0.35
14224		-0.83	
3711		-0.83	-0.75
10501		-0.83	
3860		-0.83	-1.35
7710		-0.83	-0.40
31550		-0.83	-0.15
14040		-0.83	0.33
16996		-0.83	-1.82
17838		-0.83	0.10
3546		-0.83	-0.48

	13905	-0.84	-0.27
31202		-0.84	-1.74
	4000	-0.84	-0.95
13221		-0.84	-0.60
7644		-0.84	0.01
9155		-0.84	-0.16
31196		-0.84	-0.38
15572		-0.84	-1.03
33208		-0.84	-0.47
5310		-0.84	-2.62
18775		-0.84	0.26
12490		-0.84	-0.10
11396		-0.84	-0.30
30376		-0.84	-0.39
16837		-0.84	-0.10
14915		-0.84	-0.71
6488		-0.84	1.01
	15895	-0.84	0.43
8235		-0.84	0.22
32498		-0.84	0.18
33141		-0.84	-1.20
32814		-0.84	0.21
2972		-0.84	-0.54
13345		-0.84	-0.50
32046		-0.84	-1.20
	3722	-0.84	0.04
11920		-0.84	-0.55
5720		-0.84	-0.52
4086		-0.84	-1.72
	2050	-0.84	0.07
	224	-0.84	-1.13
8026		-0.84	0.43
14039		-0.84	-1.10
1665		-0.84	-0.46
	13940	-0.84	-1.37
	19807	-0.84	-0.56
	11378	-0.84	-1.04
15471		-0.84	-1.27
3289		-0.84	-0.95
12970		-0.84	-0.97
5000		-0.84	-0.40
12042		-0.84	-0.51
31287		-0.84	-0.42
5290		-0.84	-1.08
5586		-0.84	-1.17

32401		-0.84	-0.16
15033		-0.84	-0.04
	13937	-0.84	-2.34
	20082	-0.85	-0.54
8704		-0.85	-0.82
5846		-0.85	1.66
17301		-0.85	-1.63
9323		-0.85	-0.20
7587		-0.85	-0.89
12825		-0.85	-2.23
10129		-0.85	0.90
10584		-0.85	-0.19
18336		-0.85	-0.97
4200		-0.85	-0.40
31352		-0.85	-0.48
9320		-0.85	-0.41
2962		-0.85	-1.06
5489		-0.85	-0.40
12307		-0.85	-1.11
7142		-0.85	0.21
6137		-0.85	0.59
8971		-0.85	-0.94
4049		-0.85	-0.56
	2071	-0.85	-0.69
	10515	-0.85	0.14
13588		-0.85	-1.05
32577		-0.85	-0.63
9272		-0.85	0.77
6776		-0.85	0.55
32082		-0.85	0.27
13194		-0.85	-2.10
14307		-0.85	-1.05
32110		-0.85	0.63
	20009	-0.85	0.52
3665		-0.85	-1.27
8152		-0.85	-3.11
11608		-0.85	-0.35
4349		-0.85	-1.21
14573		-0.85	-1.02
	2867	-0.85	-0.20
31498		-0.85	-0.71
12202		-0.85	-0.19
10938		-0.85	0.18
	19691	-0.85	-0.29
1635		-0.85	-1.37

30390		-0.85	-1.68
8205		-0.86	-1.13
	15315	-0.86	-0.57
8178		-0.86	-0.33
3267		-0.86	-0.99
8947		-0.86	-1.45
	11206	-0.86	-1.45
15745		-0.86	-0.72
15896		-0.86	-1.34
16833		-0.86	-0.85
	12415	-0.86	0.25
3796		-0.86	1.53
12158		-0.86	-0.87
17329		-0.86	-0.03
10954		-0.86	-1.08
8643		-0.86	0.77
31083		-0.86	-0.35
	20060	-0.86	-1.09
32846		-0.86	-1.01
17350		-0.86	-0.62
3664		-0.86	0.06
	9819	-0.86	0.00
17565		-0.86	-1.28
17154		-0.86	-1.20
1345		-0.86	-0.26
8269		-0.86	-1.18
9450		-0.86	-0.59
32549		-0.86	-1.91
	16647	-0.86	-1.18
13063		-0.86	-1.72
12910		-0.86	-0.21
3986		-0.86	-0.32
	7057	-0.86	-0.21
7867		-0.86	-0.79
17385		-0.86	-0.16
9031		-0.86	-1.41
12700		-0.86	0.15
6513		-0.86	-0.88
14080		-0.86	-0.60
4697		-0.86	-0.28
32579		-0.86	-1.75
3571		-0.86	0.20
32024		-0.86	0.37
14902		-0.86	-1.45
7102		-0.86	0.67

4036		-0.86	-1.09
	20063	-0.86	-1.12
9836		-0.86	-1.09
9411		-0.86	-1.24
9834		-0.86	0.70
8475		-0.86	0.88
1391		-0.86	-0.35
9129		-0.87	-0.54
6230		-0.87	0.14
3818		-0.87	-0.84
31275		-0.87	-0.83
2911		-0.87	-0.63
	14605	-0.87	-2.06
4949		-0.87	-1.59
	388	-0.87	-1.13
10151		-0.87	-1.39
17876		-0.87	0.61
1637		-0.87	-1.02
10395		-0.87	0.00
10171		-0.87	-0.08
5549		-0.87	-0.44
13783		-0.87	-0.27
	19854	-0.87	-0.37
13437		-0.87	0.13
32550		-0.87	0.03
8843		-0.87	-0.21
18188		-0.87	-0.55
10971		-0.87	-2.27
15003		-0.87	-0.91
	20135	-0.87	-1.91
7855		-0.87	-0.09
7849		-0.87	0.48
	12658	-0.87	-0.82
6290		-0.87	-0.32
2411		-0.87	-0.51
12901		-0.87	-1.19
31175		-0.87	-0.93
13857		-0.87	0.16
	328	-0.87	-0.41
4009		-0.87	-1.10
17184		-0.87	-0.45
	16694	-0.87	-0.65
32032		-0.87	-0.26
13506		-0.87	0.27
7582		-0.87	0.23

	17592	-0.87	-0.66
3106		-0.87	-0.80
2885		-0.87	-0.60
9878		-0.87	-1.31
	18435	-0.87	-0.54
32159		-0.87	-2.21
6621		-0.87	0.48
3039		-0.87	-1.31
12693		-0.87	0.08
3510		-0.88	-1.06
11562		-0.88	0.28
9889		-0.88	-1.40
	3065	-0.88	-0.13
6971		-0.88	0.84
7946		-0.88	-0.83
16870		-0.88	0.31
	17351	-0.88	0.07
11907		-0.88	-0.54
31374		-0.88	-0.41
	16684	-0.88	-0.05
11268		-0.88	0.47
5849		-0.88	-0.64
2851		-0.88	-0.93
15537		-0.88	0.67
5124		-0.88	-0.38
12240		-0.88	-2.00
5639		-0.88	-1.15
7337		-0.88	0.35
16885		-0.88	-1.81
14195		-0.88	-1.19
32043		-0.88	-1.38
32828		-0.88	0.25
	14318	-0.88	0.64
31418		-0.88	-1.12
	15896	-0.88	-0.07
	8159	-0.88	-0.36
16965		-0.88	0.05
	6629	-0.88	-0.40
30394		-0.88	-1.07
11906		-0.88	-1.05
	5671	-0.88	0.54
32013		-0.88	-0.28
12105		-0.88	-0.70
32563		-0.88	1.53
33456		-0.88	-0.92

13826		-0.88	0.91
6241		-0.88	-0.15
6395		-0.89	-2.22
12859		-0.89	-0.13
6467		-0.89	1.45
3088		-0.89	-0.41
18024		-0.89	-0.68
2879		-0.89	-1.01
1866		-0.89	-0.52
15377		-0.89	-0.29
12756		-0.89	-0.96
7471		-0.89	-0.82
3862		-0.89	-2.40
10991		-0.89	-2.30
9821		-0.89	0.12
12224		-0.89	-0.73
13690		-0.89	0.05
2206		-0.89	-2.47
15221		-0.89	0.08
15030		-0.89	-1.07
	9876	-0.89	-0.25
31037		-0.89	-0.19
12796		-0.89	0.22
12414		-0.89	0.63
4030		-0.89	-1.27
31661		-0.89	0.32
13033		-0.89	0.33
14756		-0.89	-0.67
32393		-0.89	-0.62
13540		-0.89	-1.83
14673		-0.89	-1.43
9412		-0.89	0.76
7846		-0.89	-0.75
12179		-0.89	0.41
3352		-0.89	-1.00
1103		-0.89	1.15
	11142	-0.89	-0.51
1621		-0.90	-1.23
6055		-0.90	-1.48
2983		-0.90	0.21
1358		-0.90	-1.06
	9390	-0.90	-0.52
	5864	-0.90	-0.71
15028		-0.90	-0.73
8216		-0.90	-0.37

31755		-0.90	-0.57
18111		-0.90	-0.12
16739		-0.90	-0.25
	2020	-0.90	0.00
	7888	-0.90	0.47
18735		-0.90	0.08
2699		-0.90	-0.31
4905		-0.90	0.35
5358		-0.90	0.10
31208		-0.90	0.28
12115		-0.90	-0.15
15497		-0.90	-0.33
31163		-0.90	0.03
18648		-0.90	-1.13
33463		-0.90	0.19
	17031	-0.90	-0.36
11034		-0.90	-0.30
14309		-0.90	0.29
11120		-0.90	0.03
18241		-0.90	-0.69
8335		-0.90	0.00
6019		-0.90	-0.23
2179		-0.90	-0.42
31988		-0.90	0.42
6931		-0.90	0.19
	8196	-0.90	-1.26
12492		-0.90	-2.14
3695		-0.90	-0.30
10998		-0.90	0.91
10887		-0.90	0.27
14163		-0.90	0.66
31029		-0.90	-0.01
13951		-0.91	-2.07
1454		-0.91	-1.80
4032		-0.91	-1.61
5081		-0.91	-0.50
16849		-0.91	-1.12
3691		-0.91	-2.46
13997		-0.91	-0.85
10474		-0.91	1.20
8709		-0.91	0.46
5074		-0.91	0.43
13295		-0.91	-0.35
6220		-0.91	-1.95
	16926	-0.91	0.00

32532		-0.91	-0.65
4015		-0.91	-1.76
3198		-0.91	-1.51
13731		-0.91	-0.17
4202		-0.91	-0.46
	2979	-0.91	2.32
2647		-0.91	0.09
8547		-0.91	-1.92
5545		-0.91	-0.89
7950		-0.91	-0.95
31807		-0.91	-0.04
30503		-0.91	-0.78
14186		-0.91	0.25
	16366	-0.91	-0.47
6871		-0.91	1.05
2064		-0.91	-0.80
	5813	-0.91	-1.18
7358		-0.91	0.02
9271		-0.91	-0.78
11652		-0.91	-1.90
11384		-0.91	-1.31
4502		-0.91	-1.61
14622		-0.91	-2.01
7866		-0.92	-1.94
8132		-0.92	1.04
12014		-0.92	-1.02
30357		-0.92	0.17
9933		-0.92	-1.15
16771		-0.92	-0.48
6457		-0.92	-0.99
5779		-0.92	-0.14
	12791	-0.92	-0.41
2694		-0.92	-0.60
15814		-0.92	0.34
30054		-0.92	-0.14
5105		-0.92	-2.94
8777		-0.92	0.15
10564		-0.92	-2.00
3269		-0.92	-0.39
14890		-0.92	-1.30
33261		-0.92	0.55
4921		-0.92	-0.98
3572		-0.92	-0.11
2218		-0.92	0.17
17219		-0.92	-0.70

17199		-0.92	0.34
4338		-0.92	0.00
15926		-0.92	-0.99
17224		-0.92	-1.50
9595		-0.92	-0.53
31956		-0.92	-0.48
18605		-0.92	-0.94
9468		-0.92	0.29
32005		-0.93	0.03
15034		-0.93	-0.61
11398		-0.93	-1.12
1864		-0.93	-0.86
31547		-0.93	0.37
5508		-0.93	-1.48
14401		-0.93	-1.36
30040		-0.93	-1.16
30445		-0.93	-1.46
8213		-0.93	-0.44
5520		-0.93	-0.49
1030		-0.93	-1.32
	10081	-0.93	1.28
30114		-0.93	-0.37
14952		-0.93	-0.39
31737		-0.93	-0.57
15923		-0.93	-1.16
	3977	-0.93	-0.40
	2899	-0.93	-0.56
12118		-0.93	-1.28
	8108	-0.93	-0.23
14746		-0.93	-2.06
15666		-0.93	0.11
5274		-0.93	-0.82
13719		-0.93	-0.32
2177		-0.93	-0.22
2161		-0.93	0.70
8708		-0.93	-1.59
12210		-0.93	-1.08
7015		-0.93	-0.95
4878		-0.93	-0.06
17042		-0.93	-2.40
11523		-0.93	-2.28
15657		-0.93	-0.26
12386		-0.93	0.20
8664		-0.93	-0.67
5888		-0.93	2.22

11326		-0.93	-0.20
13312		-0.93	-1.00
	8233	-0.93	-1.39
31025		-0.93	-0.52
4261		-0.93	-1.34
12539		-0.93	-2.61
	6456	-0.93	0.40
2980		-0.93	-0.68
8159		-0.93	-0.54
8439		-0.94	-0.01
15708		-0.94	-2.09
30374		-0.94	0.87
17003		-0.94	-1.59
	14321	-0.94	0.52
1753		-0.94	-0.55
5883		-0.94	-1.25
11626		-0.94	-0.70
30088		-0.94	-0.96
3059		-0.94	0.12
13362		-0.94	-0.93
1863		-0.94	-1.51
4196		-0.94	-0.14
2926		-0.94	-0.98
13711		-0.94	-1.30
15001		-0.94	-1.79
12641		-0.94	-0.62
18740		-0.94	0.06
2984		-0.94	-0.24
	20122	-0.94	-2.09
4135		-0.94	-0.10
14894		-0.94	-1.16
5372		-0.94	-1.18
9699		-0.94	-1.07
	12266	-0.94	0.85
1890		-0.94	0.54
2202		-0.94	-0.29
7543		-0.94	-1.72
18041		-0.94	-0.12
7554		-0.94	-0.45
33137		-0.94	-1.21
32037		-0.94	-0.87
	7444	-0.94	-1.80
17279		-0.94	-0.08
15437		-0.95	-1.94
4376		-0.95	-0.23

13568		-0.95	0.47
8565		-0.95	-0.19
12497		-0.95	-1.76
6830		-0.95	0.32
15374		-0.95	-1.19
31342		-0.95	-1.06
9205		-0.95	-1.12
13121		-0.95	-2.42
2069		-0.95	0.42
6196		-0.95	-0.39
11137		-0.95	-0.37
14358		-0.95	0.24
12868		-0.95	-0.41
	9086	-0.95	0.04
7989		-0.95	-1.66
9490		-0.95	-2.45
9653		-0.95	-1.51
17343		-0.95	-1.59
12085		-0.95	-0.78
14736		-0.95	-0.99
10191		-0.95	-0.20
8667		-0.95	-0.14
2260		-0.95	-0.97
8256		-0.95	-0.16
11312		-0.95	-0.95
15797		-0.95	-0.01
4152		-0.95	-1.36
	11843	-0.95	-1.20
9127		-0.95	0.73
13045		-0.95	0.15
2174		-0.95	0.94
32214		-0.95	-0.17
15470		-0.95	-1.21
	6910	-0.95	-2.13
31706		-0.95	-0.94
	15060	-0.95	-1.03
10827		-0.95	-0.41
15693		-0.95	1.02
12181		-0.95	-0.38
2023		-0.95	-1.63
10652		-0.95	-1.06
7742		-0.96	0.17
12288		-0.96	-0.95
3853		-0.96	-1.98
10781		-0.96	0.44

2212		-0.96	-0.34
14120		-0.96	-1.04
6294		-0.96	-0.67
7860		-0.96	-0.92
15425		-0.96	-0.08
33191		-0.96	-0.51
1871		-0.96	-2.02
15642		-0.96	-0.08
7009		-0.96	0.49
31714		-0.96	-0.08
4756		-0.96	0.31
7573		-0.96	-0.94
16998		-0.96	-1.92
13806		-0.96	-0.28
13400		-0.96	-0.82
32039		-0.96	-0.25
5103		-0.96	-0.34
	3840	-0.96	-1.37
6227		-0.96	0.23
10959		-0.96	-1.55
32589		-0.96	-1.11
	20072	-0.96	-1.08
17440		-0.96	-0.34
6282		-0.96	0.60
17377		-0.96	-0.70
6725		-0.96	0.77
4118		-0.96	-1.41
17166		-0.96	-0.66
2713		-0.97	-0.89
15740		-0.97	-1.09
	8777	-0.97	0.48
10914		-0.97	0.11
17843		-0.97	-0.94
6989		-0.97	0.45
31072		-0.97	-2.04
1340		-0.97	-0.60
17839		-0.97	0.24
18281		-0.97	-1.56
4168		-0.97	-0.78
13388		-0.97	0.00
5149		-0.97	-0.11
	1059	-0.97	-1.38
7061		-0.97	0.79
11249		-0.97	0.11
	11025	-0.97	-1.20

15577		-0.97	-0.54
33152		-0.97	0.04
33331		-0.97	0.17
9797		-0.97	-0.23
6497		-0.97	-1.48
11911		-0.97	-0.21
6018		-0.97	-0.42
12985		-0.97	-0.91
32019		-0.97	-1.92
7026		-0.97	-0.72
9454		-0.97	0.56
	16553	-0.97	0.04
	16564	-0.97	-0.44
13114		-0.97	-2.19
	19671	-0.97	-0.92
3714		-0.97	-0.94
8598		-0.97	1.15
33318		-0.97	-0.28
1657		-0.97	-1.44
7483		-0.97	-1.19
15042		-0.97	-0.22
17929		-0.97	-0.18
18606		-0.97	-2.03
17330		-0.97	-2.68
32199		-0.97	-0.30
3848		-0.97	0.09
31711		-0.97	-1.28
13981		-0.97	-1.34
4893		-0.97	1.05
6509		-0.97	-0.25
13401		-0.98	-0.15
5692		-0.98	-1.37
18372		-0.98	-1.42
	5802	-0.98	-0.42
7972		-0.98	-0.47
32767		-0.98	-0.04
9400		-0.98	0.37
14639		-0.98	1.70
4380		-0.98	0.16
12239		-0.98	0.16
11257		-0.98	-0.97
	8815	-0.98	0.43
14084		-0.98	0.06
33148		-0.98	-0.20
10376		-0.98	-1.63

5165		-0.98	-0.97
6893		-0.98	0.12
14180		-0.98	-1.25
33276		-0.98	-2.38
30148		-0.98	0.05
33275		-0.98	-0.58
8403		-0.98	0.15
3982		-0.98	-0.22
2574		-0.98	0.64
17785		-0.98	-0.85
	14326	-0.98	0.30
8219		-0.98	-0.37
	20056	-0.98	-0.22
9154		-0.98	-0.23
10486		-0.98	-1.51
	8545	-0.98	0.97
33279		-0.98	0.01
8108		-0.98	-2.53
7567		-0.98	-0.45
5204		-0.99	-0.30
31008		-0.99	-0.89
1646		-0.99	0.02
	17467	-0.99	-0.04
7365		-0.99	-0.68
10657		-0.99	-0.59
1115		-0.99	-0.76
1228		-0.99	-1.15
9674		-0.99	-0.84
5339		-0.99	-0.47
6048		-0.99	-0.97
	17644	-0.99	-0.59
7810		-0.99	-0.76
7891		-0.99	-1.67
10480		-0.99	-0.57
7147		-0.99	-0.38
14174		-0.99	-2.15
	13893	-0.99	-0.65
2906		-0.99	-0.60
	8792	-0.99	-1.73
13075		-0.99	-1.38
31918		-0.99	-0.66
15085		-0.99	-0.36
31412		-0.99	-0.64
31032		-0.99	-0.76
33007		-1.00	-1.96

9772		-1.00	0.64
5030		-1.00	-1.67
13057		-1.00	-2.01
14135		-1.00	-0.33
5815		-1.00	-0.77
5695		-1.00	-2.21
14671		-1.00	-0.80
3821		-1.00	0.33
18507		-1.00	0.97
3953		-1.00	0.01
	2909	-1.00	-1.22
7180		-1.00	-0.42
4701		-1.00	0.28
4572		-1.00	-0.76
32226		-1.00	-1.17
9655		-1.00	-0.56
	8169	-1.00	-0.49
15336		-1.00	-0.32
1153		-1.00	-0.15
17575		-1.00	0.00
17560		-1.00	-1.20
33084		-1.00	-1.48
10248		-1.00	-0.88
11112		-1.00	-1.18
7080		-1.01	-1.48
11357		-1.01	-0.33
	14090	-1.01	1.49
31855		-1.01	-0.77
31673		-1.01	1.01
4866		-1.01	-1.73
10363		-1.01	-1.69
	11667	-1.01	1.37
	14534	-1.01	-0.59
12838		-1.01	0.03
7966		-1.01	-1.23
13705		-1.01	
	17689	-1.01	-1.64
8733		-1.01	-0.40
1671		-1.01	-1.15
1368		-1.01	-1.42
	11212	-1.01	-1.60
15731		-1.01	-1.64
3420		-1.01	-1.27
3268		-1.01	-1.04
33092		-1.01	0.56

31492		-1.01	-0.88
32185		-1.01	-0.50
6712		-1.01	-0.74
32830		-1.01	-3.16
15685		-1.01	1.06
9990		-1.01	0.52
15634		-1.01	-0.35
14035		-1.01	-2.48
6295		-1.01	0.12
11044		-1.01	-0.16
12654		-1.01	-0.18
8408		-1.01	0.36
	9861	-1.02	0.71
13868		-1.02	0.13
13942		-1.02	-0.35
2201		-1.02	-0.37
17716		-1.02	-2.42
1655		-1.02	-1.89
	14590	-1.02	-0.37
5344		-1.02	-1.42
6421		-1.02	0.44
6842		-1.02	-1.21
7059		-1.02	-0.90
31225		-1.02	-0.02
14904		-1.02	-1.64
9504		-1.02	-1.22
3008		-1.02	-0.98
10932		-1.02	-0.46
6950		-1.02	-0.88
33321		-1.02	0.79
32602		-1.02	-0.97
6521		-1.02	-0.51
6132		-1.02	-0.36
31653		-1.02	-0.93
1659		-1.03	-1.36
	17126	-1.03	-1.15
6232		-1.03	-0.70
	9188	-1.03	-0.03
3656		-1.03	0.00
14067		-1.03	-0.60
7245		-1.03	0.45
8229		-1.03	0.49
1451		-1.03	-0.60
31203		-1.03	-0.04
9153		-1.03	-0.11

10391		-1.03	-0.56
4937		-1.03	-0.32
11825		-1.03	-0.38
11276		-1.03	1.12
11136		-1.03	-1.71
15905		-1.03	-2.41
7379		-1.03	-0.29
15347		-1.03	0.68
	9065	-1.03	-0.25
11012		-1.03	1.51
3157		-1.03	-1.48
3603		-1.03	0.69
32802		-1.03	0.40
32793		-1.03	1.35
32305		-1.03	-1.02
	7574	-1.03	-1.72
16786		-1.03	0.59
10496		-1.03	-0.47
8988		-1.03	0.70
	14635	-1.03	-1.99
8014		-1.03	-1.53
14879		-1.04	-0.50
13707		-1.04	-1.88
10435		-1.04	-1.98
15257		-1.04	0.12
14119		-1.04	-1.20
17325		-1.04	-0.60
5417		-1.04	-1.95
31094		-1.04	-2.38
7845		-1.04	-0.87
17058		-1.04	-1.00
17793		-1.04	-1.19
	14379	-1.04	-0.09
	15383	-1.04	0.00
	7498	-1.04	-1.51
12079		-1.04	-1.41
17386		-1.04	-0.79
30384		-1.04	-1.17
7897		-1.04	-0.85
	20351	-1.04	0.02
4940		-1.04	-0.60
10164		-1.04	-0.70
5671		-1.04	-0.89
	20393	-1.05	-0.82
9508		-1.05	-0.80

32805		-1.05	-0.28
9891		-1.05	-0.87
15597		-1.05	0.46
11191		-1.05	0.38
8732		-1.05	-1.72
	8133	-1.05	-1.44
8563		-1.05	0.08
31623		-1.05	-0.97
15636		-1.05	1.08
8989		-1.05	-0.67
11345		-1.05	0.27
10277		-1.05	-0.17
3621		-1.05	0.76
6026		-1.05	-0.65
11546		-1.05	1.55
31626		-1.05	-0.14
	17589	-1.05	-0.73
14861		-1.05	-0.73
5809		-1.05	-0.20
17262		-1.06	-1.27
11699		-1.06	-0.08
13994		-1.06	-0.04
6058		-1.06	-1.09
16775		-1.06	-1.49
11360		-1.06	-0.97
14229		-1.06	-1.28
6034		-1.06	-0.31
1663		-1.06	-1.18
	2997	-1.06	1.91
32223		-1.06	
30340		-1.06	-1.27
1837		-1.06	0.05
5139		-1.06	0.06
5978		-1.06	-0.48
5447		-1.06	-2.66
5595		-1.06	-0.35
8600		-1.06	0.55
5851		-1.06	-0.55
6177		-1.06	-0.11
14921		-1.06	-0.75
5168		-1.06	-1.09
15116		-1.06	0.55
	17765	-1.06	-1.89
	5888	-1.06	-1.78
	11023	-1.06	-0.55

5895		-1.06	-0.49
11680		-1.06	-1.32
1859		-1.06	-0.16
5465		-1.06	0.62
5056		-1.06	-1.62
15107		-1.06	-1.86
18304		-1.06	0.07
18593		-1.07	-1.24
1845		-1.07	-0.99
1441		-1.07	-1.21
31100		-1.07	-1.39
13377		-1.07	-0.12
12433		-1.07	-0.26
5597		-1.07	0.18
13409		-1.07	-1.30
7357		-1.07	-0.73
5225		-1.07	-2.51
9452		-1.07	-0.19
7811		-1.07	-0.39
	8171	-1.07	0.60
9079		-1.07	-0.73
7279		-1.07	-0.24
5715		-1.07	-1.33
18343		-1.07	-0.25
15653		-1.07	-0.51
13067		-1.07	-1.38
	15209	-1.07	-0.28
14834		-1.07	-0.65
15166		-1.07	-1.26
14110		-1.07	-0.03
8181		-1.07	-1.23
	14094	-1.07	0.41
	16127	-1.07	-0.31
13743		-1.07	-1.22
10475		-1.07	0.62
1698		-1.07	-0.75
14517		-1.07	-0.30
12870		-1.08	-1.13
12990		-1.08	-1.09
14148		-1.08	-1.29
6332		-1.08	-0.92
7548		-1.08	-0.52
18809		-1.08	-1.67
11322		-1.08	-0.94
31798		-1.08	-2.53

8474		-1.08	-0.26
1412		-1.08	-1.22
7115		-1.08	-0.27
9965		-1.08	-0.34
31811		-1.08	-1.81
2060		-1.08	-1.08
7921		-1.08	-0.01
4393		-1.08	-0.83
9924		-1.08	-1.07
4262		-1.08	-1.66
32402		-1.08	-1.14
12078		-1.08	-1.90
8049		-1.08	0.07
32202		-1.08	0.00
6116		-1.08	-0.86
7865		-1.08	-0.92
10494		-1.08	-1.59
18659		-1.08	-2.75
9054		-1.08	-0.24
10361		-1.08	-1.97
4050		-1.08	-0.82
8079		-1.08	-1.54
15002		-1.09	-0.84
	17336	-1.09	-0.61
32575		-1.09	-0.35
12971		-1.09	0.44
17205		-1.09	1.72
8385		-1.09	-0.98
8912		-1.09	0.01
5321		-1.09	-1.29
9925		-1.09	-0.66
1176		-1.09	0.05
6401		-1.09	0.03
9395		-1.09	-0.18
8503		-1.09	0.10
	16821	-1.09	-1.19
5406		-1.09	-1.48
14532		-1.09	-0.03
1986		-1.09	-0.35
32680		-1.09	-2.45
10671		-1.09	-0.57
5154		-1.09	-1.13
9588		-1.10	0.91
30269		-1.10	-0.05
31463		-1.10	0.25

5794		-1.10	-0.19
	10197	-1.10	-0.62
10205		-1.10	-0.98
9171		-1.10	0.87
17967		-1.10	-0.39
8102		-1.10	0.08
9336		-1.10	0.09
6673		-1.10	-0.67
8034		-1.10	0.11
17244		-1.10	-1.71
8681		-1.10	-0.18
1133		-1.10	-1.41
10052		-1.10	-1.11
11023		-1.10	0.03
32641		-1.10	0.39
9099		-1.10	-0.08
3582		-1.10	-0.09
2017		-1.10	-0.65
10741		-1.10	-0.52
16733		-1.10	-0.52
31600		-1.10	-0.52
11455		-1.10	-0.16
14193		-1.10	0.17
14575		-1.10	0.74
33097		-1.10	-2.08
3858		-1.10	0.46
4669		-1.10	-2.45
3100		-1.10	-0.92
9429		-1.10	-0.27
	16402	-1.10	-0.16
5187		-1.11	0.13
11381		-1.11	-1.03
10695		-1.11	-3.01
1987		-1.11	-1.56
33287		-1.11	0.59
13977		-1.11	-0.37
3492		-1.11	-1.82
	19955	-1.11	-0.90
31098		-1.11	-0.19
8543		-1.11	1.18
	19951	-1.11	-0.46
18444		-1.11	-0.09
15908		-1.11	0.06
5697		-1.11	-1.53
14748		-1.11	-0.95

8142		-1.11	-0.74
31499		-1.11	0.31
5968		-1.11	-1.87
15707		-1.11	-0.77
15184		-1.11	1.57
2702		-1.11	-0.96
3897		-1.11	-1.09
1980		-1.11	2.07
12866		-1.11	0.12
9270		-1.12	-1.00
11367		-1.12	-0.97
32235		-1.12	-1.39
	10908	-1.12	-1.31
	15291	-1.12	-0.78
11237		-1.12	-0.85
30448		-1.12	-1.08
	6576	-1.12	-0.78
30490		-1.12	0.11
32146		-1.12	-1.60
6092		-1.12	0.14
30345		-1.12	-3.13
8585		-1.12	0.71
12065		-1.12	-1.12
7708		-1.12	-1.26
1434		-1.12	-1.61
	10938	-1.12	0.16
12833		-1.12	0.65
10009		-1.12	-0.47
	15531	-1.12	-0.44
14454		-1.12	-2.38
	1104	-1.12	-1.01
11967		-1.12	-1.30
4889		-1.12	1.14
17207		-1.12	-1.35
11878		-1.12	0.02
1511		-1.12	-1.05
15825		-1.12	-1.20
17597		-1.12	-2.33
3328		-1.12	-1.91
	5879	-1.13	-1.57
4389		-1.13	-1.31
4210		-1.13	-1.56
8637		-1.13	0.17
18389		-1.13	-1.50
31190		-1.13	-1.24

13039		-1.13	-2.04
18294		-1.13	-1.56
1891		-1.13	-0.96
6724		-1.13	-0.52
15329		-1.13	-2.20
5032		-1.13	-2.01
15649		-1.13	0.39
14376		-1.13	0.06
3040		-1.13	-0.73
31122		-1.13	0.10
9109		-1.13	1.20
11617		-1.13	-2.06
4306		-1.13	-0.16
	13358	-1.13	0.00
12213		-1.13	-0.07
2701		-1.13	-0.55
1664		-1.13	-1.14
10223		-1.13	1.36
3152		-1.13	-1.50
8166		-1.13	-1.59
3732		-1.13	0.46
	12949	-1.13	-1.06
15873		-1.13	-1.81
8343		-1.13	-0.80
4192		-1.13	-0.43
31778		-1.13	-0.39
	8138	-1.13	0.49
10849		-1.13	0.05
8003		-1.14	-1.21
5195		-1.14	-0.98
6864		-1.14	-0.72
31060		-1.14	-1.85
6315		-1.14	0.77
4229		-1.14	0.25
10399		-1.14	-0.82
1152		-1.14	0.37
	7448	-1.14	0.47
1200		-1.14	-1.80
4703		-1.14	-0.63
32184		-1.14	-0.36
15388		-1.14	-1.91
10145		-1.14	-0.45
13066		-1.14	-2.14
2976		-1.14	-1.16
7110		-1.14	-0.61

32707		-1.14	-0.49
17334		-1.14	-1.92
6915		-1.14	-0.37
32042		-1.14	-0.76
6398		-1.14	-2.26
15855		-1.14	0.85
12775		-1.14	0.04
15593		-1.14	-0.48
	11478	-1.14	-0.48
	16206	-1.14	-0.09
4230		-1.14	-1.89
16854		-1.14	-1.08
	1066	-1.14	-1.34
5188		-1.14	-0.63
3682		-1.15	-0.26
30060		-1.15	0.65
9691		-1.15	-1.46
11720		-1.15	-2.03
6422		-1.15	0.38
31231		-1.15	-0.37
1945		-1.15	-1.85
18030		-1.15	0.09
15178		-1.15	1.02
3194		-1.15	-1.19
13193		-1.15	0.31
9871		-1.15	-0.55
31562		-1.15	-0.64
32461		-1.15	-0.10
11281		-1.15	-0.21
17242		-1.15	-1.67
12249		-1.15	-0.57
31010		-1.15	-1.51
7848		-1.15	-0.89
11870		-1.15	-2.52
8613		-1.15	1.80
1842		-1.15	-1.58
12055		-1.15	0.44
11349		-1.15	0.00
	13886	-1.15	-1.54
31171		-1.15	-1.78
12691		-1.15	-0.79
8192		-1.15	-0.36
32241		-1.16	-1.18
10646		-1.16	-0.72
31109		-1.16	-0.47

7578		-1.16	-0.17
9285		-1.16	0.15
1487		-1.16	-1.43
	16611	-1.16	-0.81
9467		-1.16	-0.60
30094		-1.16	0.15
17275		-1.16	0.25
15734		-1.16	-1.26
14259		-1.16	-1.09
14814		-1.16	0.70
17914		-1.16	-0.83
33214		-1.16	-0.83
6136		-1.16	-0.05
7828		-1.16	-1.01
17348		-1.16	-0.15
15920		-1.16	-0.01
30338		-1.16	-0.37
18628		-1.16	-2.11
30467		-1.16	-2.35
4659		-1.16	-1.34
12278		-1.16	-0.38
2975		-1.16	-1.80
6178		-1.16	0.63
1817		-1.16	-0.30
9030		-1.16	-1.07
13645		-1.16	0.11
12917		-1.16	0.21
4208		-1.17	-1.29
31640		-1.17	-0.85
10075		-1.17	-1.52
5870		-1.17	-0.88
31616		-1.17	-0.55
1420		-1.17	-0.43
4758		-1.17	-0.08
	12044	-1.17	-2.82
7971		-1.17	-0.34
7186		-1.17	-1.40
6541		-1.17	-0.85
15609		-1.17	0.60
32677		-1.17	-0.27
11176		-1.17	-1.93
3129		-1.17	-1.28
12317		-1.17	-0.69
	13878	-1.17	-1.99
	11209	-1.17	-1.65

4455		-1.17	-2.89
8597		-1.17	0.57
2617		-1.17	-0.08
13250		-1.18	-0.64
4743		-1.18	-1.04
8651		-1.18	-2.33
6923		-1.18	-1.45
3642		-1.18	-0.28
	19161	-1.18	-1.65
31716		-1.18	-1.25
30475		-1.18	-1.10
6738		-1.18	-1.75
10869		-1.18	-0.10
3134		-1.18	-1.83
2245		-1.18	-0.98
7869		-1.18	
	11307	-1.18	-1.40
5859		-1.18	-0.56
13315		-1.19	0.97
30116		-1.19	-1.35
	7472	-1.19	0.37
9344		-1.19	0.74
32791		-1.19	-2.59
	8747	-1.19	0.78
17240		-1.19	-0.47
10697		-1.19	-1.02
16850		-1.19	-0.45
14655		-1.19	-1.08
6535		-1.19	-0.63
	14222	-1.19	-0.13
4043		-1.19	-0.42
1523		-1.19	-0.89
17052		-1.19	-0.65
16879		-1.19	1.33
7717		-1.19	-1.72
11989		-1.19	-0.85
13538		-1.19	-0.49
7920		-1.19	-1.25
13930		-1.19	-1.84
6437		-1.20	-0.50
	19897	-1.20	0.35
	17586	-1.20	-1.98
1838		-1.20	-0.34
6042		-1.20	-0.47
5510		-1.20	-0.78

14294		-1.20	-0.45
	16429	-1.20	-1.77
17735		-1.20	-1.20
	14861	-1.20	-1.80
6891		-1.20	-0.96
31649		-1.20	-0.25
6796		-1.20	-0.20
18817		-1.20	0.26
30426		-1.20	0.21
5377		-1.20	-1.42
32231		-1.20	0.34
7010		-1.20	0.45
15478		-1.20	-1.64
7282		-1.20	0.47
33315		-1.20	-0.73
10759		-1.20	-0.02
1884		-1.20	-0.37
6706		-1.20	-1.60
	13505	-1.20	-0.98
10307		-1.20	0.12
6208		-1.20	-0.73
14043		-1.21	-0.39
4859		-1.21	-0.79
6996		-1.21	-2.75
	15625	-1.21	-0.60
2520		-1.21	-1.24
11908		-1.21	-1.62
4294		-1.21	0.34
8495		-1.21	1.33
14935		-1.21	-1.03
12809		-1.21	-1.59
	19000	-1.21	-1.09
12184		-1.21	0.01
	8011	-1.21	-0.52
10747		-1.21	-0.02
15270		-1.21	-2.61
31347		-1.21	-1.18
4800		-1.21	-0.27
5021		-1.21	-1.03
	8231	-1.21	-2.21
33051		-1.21	-0.61
9675		-1.21	-0.58
8805		-1.21	-0.41
3534		-1.21	0.21
13428		-1.21	-0.89

31102		-1.21	-0.56
18319		-1.21	-1.58
2736		-1.21	-0.24
30002		-1.21	-0.97
3699		-1.21	-0.46
9300		-1.21	-0.06
5965		-1.22	0.61
10182		-1.22	-0.21
	16221	-1.22	-0.68
	17764	-1.22	-1.92
16707		-1.22	-1.01
8677		-1.22	0.12
5892		-1.22	-0.77
31045		-1.22	-0.68
4382		-1.22	-0.43
12359		-1.22	0.67
7718		-1.22	-0.83
3978		-1.22	0.46
13673		-1.22	-0.07
1193		-1.22	-1.39
12841		-1.22	0.05
6691		-1.22	-0.41
32743		-1.22	-2.46
3822		-1.22	0.00
33329		-1.22	-0.55
7003		-1.22	-1.03
8849		-1.22	-1.28
	7405	-1.22	-0.43
10956		-1.22	-1.64
3664		-1.23	-0.95
11900		-1.23	-0.45
5248		-1.23	-1.54
10732		-1.23	0.82
	7490	-1.23	0.92
9842		-1.23	-1.13
1825		-1.23	-2.65
6683		-1.23	0.92
10579		-1.23	-1.00
	13888	-1.23	-0.61
12206		-1.23	-0.82
9466		-1.23	-1.19
10799		-1.23	-0.93
15172		-1.23	-1.07
	13539	-1.23	0.07
5249		-1.23	-1.31

32922		-1.23	-0.29
	7561	-1.23	-0.71
32193		-1.23	-1.99
15578		-1.23	0.11
5781		-1.23	-0.64
3326		-1.23	-1.65
1548		-1.23	-0.99
17090		-1.23	0.00
11305		-1.23	-0.89
	10836	-1.23	-0.03
10546		-1.23	-1.61
13773		-1.23	-0.10
3799		-1.24	-0.25
1868		-1.24	-0.73
7656		-1.24	-1.43
8548		-1.24	0.47
4269		-1.24	-1.24
2061		-1.24	-0.77
	2637	-1.24	-1.16
7016		-1.24	-1.32
17525		-1.24	-0.82
	7709	-1.24	-2.84
7951		-1.24	0.53
6465		-1.24	-0.15
13581		-1.24	-0.23
18011		-1.24	-2.11
33102		-1.24	-1.37
1915		-1.24	-0.85
	17695	-1.24	-0.83
13659		-1.24	-0.56
10662		-1.24	-0.04
3304		-1.24	-1.11
31677		-1.24	0.36
10185		-1.24	-0.82
4568		-1.24	-1.88
7539		-1.25	0.33
8545		-1.25	0.23
1673		-1.25	-1.06
13761		-1.25	0.03
32626		-1.25	-1.21
30421		-1.25	-1.16
1795		-1.25	-0.54
30074		-1.25	-1.32
6739		-1.25	0.66
10612		-1.25	-0.85

12826		-1.25	-0.76
30029		-1.25	-0.50
6554		-1.25	-0.94
9972		-1.25	-0.79
32633		-1.25	-1.00
10146		-1.25	-0.74
	2165	-1.25	-1.22
	17446	-1.25	-1.26
11983		-1.25	0.43
2970		-1.26	-0.64
32798		-1.26	-1.35
	7260	-1.26	0.37
14763		-1.26	0.09
9487		-1.26	-0.44
12534		-1.26	-1.81
	11852	-1.26	-1.19
2244		-1.26	-0.60
31271		-1.26	-2.70
18812		-1.26	-3.11
10618		-1.26	-1.78
7970		-1.26	-1.02
6235		-1.26	-2.33
3325		-1.26	-1.58
7986		-1.26	-1.09
2746		-1.26	-0.52
2861		-1.26	-1.79
10325		-1.26	
3823		-1.26	-2.31
	17687	-1.26	-1.71
11228		-1.26	-0.86
9328		-1.26	-1.09
32469		-1.26	-1.68
12113		-1.26	-1.12
2259		-1.27	-0.20
14052		-1.27	-0.52
30068		-1.27	-1.50
8546		-1.27	0.31
3565		-1.27	-0.93
3127		-1.27	-1.11
10202		-1.27	-1.50
10488		-1.27	0.80
13473		-1.27	-0.45
13482		-1.27	-0.54
5362		-1.27	-0.99
5969		-1.27	0.22

18765		-1.27	-0.62
10068		-1.27	-1.40
33189		-1.28	-1.49
14656		-1.28	0.79
6967		-1.28	-0.63
10023		-1.28	0.00
	11347	-1.28	-1.91
3678		-1.28	-1.01
10582		-1.28	-0.77
10536		-1.28	-0.01
2714		-1.28	-1.24
10476		-1.28	-0.82
7024		-1.28	-0.67
16784		-1.28	0.43
13299		-1.28	0.52
7290		-1.28	-2.32
9659		-1.28	-1.41
32848		-1.28	-1.07
4329		-1.28	0.28
4390		-1.28	-2.10
31691		-1.29	-1.34
11059		-1.29	-0.30
13343		-1.29	-0.13
	8195	-1.29	-0.97
10598		-1.29	-0.26
15632		-1.29	-0.55
6249		-1.29	-0.71
10538		-1.29	-2.35
5751		-1.29	-0.85
31749		-1.29	-1.22
	19804	-1.29	-0.02
11993		-1.29	-0.98
2977		-1.29	-1.49
15684		-1.29	0.55
31666		-1.29	2.28
6948		-1.30	-1.17
	10835	-1.30	0.95
17888		-1.30	0.16
32156		-1.30	-0.69
33175		-1.30	-0.67
15442		-1.30	0.27
10776		-1.30	-0.47
13065		-1.30	-1.44
3508		-1.30	-1.06
	11910	-1.30	-1.22

6946		-1.30	-0.13
13069		-1.30	-0.14
2053		-1.30	-0.43
15389		-1.30	-2.12
32406		-1.30	-0.65
7200		-1.30	0.69
31286		-1.31	0.31
32703		-1.31	-1.18
3308		-1.31	-1.69
8069		-1.31	0.48
11331		-1.31	-0.90
4372		-1.31	-2.32
14785		-1.31	0.14
7692		-1.31	0.60
5281		-1.31	-1.89
7830		-1.31	
14230		-1.31	-2.20
4879		-1.31	-1.01
10723		-1.31	0.94
3368		-1.31	-2.16
10006		-1.31	-0.27
10930		-1.31	-1.87
	12033	-1.31	-1.50
1407		-1.31	-0.21
9847		-1.31	-1.88
30457		-1.32	-1.55
3771		-1.32	0.24
3701		-1.32	-0.48
15261		-1.32	-0.32
3700		-1.32	-0.89
13068		-1.32	-0.61
31651		-1.32	1.39
17522		-1.32	-0.33
8622		-1.32	-1.44
6945		-1.32	-0.23
31697		-1.32	-2.22
2924		-1.32	-0.63
4606		-1.32	-1.34
6446		-1.32	-0.63
11309		-1.32	-0.08
8187		-1.32	-1.02
6818		-1.32	-0.48
3766		-1.32	0.76
5067		-1.32	-1.83
	1943	-1.32	-1.12

1818		-1.32	-0.25
32847		-1.33	-0.49
32078		-1.33	-0.14
11942		-1.33	-1.19
32237		-1.33	-1.97
17379		-1.33	-0.40
10420		-1.33	-1.23
4412		-1.33	-1.76
6580		-1.33	-1.31
6578		-1.33	-1.52
10143		-1.33	-1.01
31643		-1.33	-0.08
	8098	-1.33	-1.65
	15743	-1.33	-2.77
14610		-1.33	-0.96
15382		-1.33	-1.46
31671		-1.33	-0.63
	15724	-1.33	-2.07
17202		-1.33	0.09
8326		-1.33	-2.05
17762		-1.33	0.57
8128		-1.33	0.35
12489		-1.34	-0.76
31813		-1.34	-0.22
31090		-1.34	1.58
6868		-1.34	-1.56
13330		-1.34	-1.84
7924		-1.34	-0.06
8048		-1.34	0.26
1506		-1.34	-1.08
7502		-1.34	-0.17
12034		-1.34	-0.97
17549		-1.34	-2.23
14334		-1.34	0.33
3690		-1.34	-1.17
12126		-1.34	0.25
9858		-1.34	-0.13
2682		-1.35	-0.62
7370		-1.35	0.33
11172		-1.35	-1.49
6358		-1.35	-1.13
14479		-1.35	-2.34
3285		-1.35	-1.29
10234		-1.35	-1.35
4719		-1.35	-2.10

33317		-1.35	0.27
32390		-1.35	-0.44
	12768	-1.35	-0.58
3702		-1.35	-0.18
5548		-1.35	-1.07
31216		-1.35	-1.19
14047		-1.35	-1.09
13466		-1.35	0.25
5903		-1.36	-0.41
	13498	-1.36	-0.55
4483		-1.36	-0.98
13586		-1.36	-1.31
1034		-1.36	0.00
10626		-1.36	-1.26
7407		-1.36	0.22
11123		-1.36	0.34
3024		-1.36	-1.18
5460		-1.36	1.12
31248		-1.37	-0.24
	15492	-1.37	-0.87
	17465	-1.37	-0.73
9745		-1.37	-0.25
1021		-1.37	-1.08
9265		-1.37	-1.48
5492		-1.37	-2.23
	11317	-1.37	-1.70
3156		-1.37	-1.58
30166		-1.37	-0.66
7639		-1.37	-0.62
1650		-1.37	-0.43
3927		-1.37	-0.01
1701		-1.37	-0.72
32103		-1.38	-2.32
7546		-1.38	-1.49
5768		-1.38	-0.25
9465		-1.38	-1.47
8420		-1.38	0.53
31528		-1.38	-1.72
1897		-1.38	-0.28
32473		-1.38	0.72
6441		-1.38	-2.42
	16555	-1.38	-1.37
32596		-1.38	-1.83
14965		-1.39	-0.04
10897		-1.39	0.29

14864		-1.39	1.29
9760		-1.39	-0.68
13127		-1.39	-0.20
30380		-1.39	-2.12
11103		-1.39	-2.07
4346		-1.39	0.20
	19979	-1.39	-1.99
	14459	-1.39	-1.47
32141		-1.39	-1.22
	16824	-1.39	-2.32
12947		-1.39	-1.00
3298		-1.39	0.84
15760		-1.39	0.04
14621		-1.39	-2.36
8909		-1.39	0.84
11538		-1.39	0.04
32559		-1.40	-1.47
17035		-1.40	-1.11
17144		-1.40	-0.48
7988		-1.40	-1.79
17870		-1.40	-0.07
15741		-1.40	-2.24
13404		-1.40	-0.85
	7115	-1.40	-0.21
7655		-1.40	-1.45
8184		-1.40	-0.19
2056		-1.40	-1.57
32691		-1.40	-0.83
6765		-1.41	0.37
6195		-1.41	0.58
5269		-1.41	0.40
	17606	-1.41	-0.59
5525		-1.41	-1.45
8342		-1.41	-1.31
	17588	-1.41	-1.21
	17006	-1.41	-2.43
5379		-1.41	-1.63
2507		-1.41	-0.81
8090		-1.41	-1.80
7356		-1.41	-1.78
32386		-1.41	-1.42
10728		-1.41	-1.30
6520		-1.41	-1.04
	11179	-1.41	-0.93
7881		-1.42	-0.55

5937		-1.42	-0.39
13587		-1.42	-2.11
14452		-1.42	-0.56
13038		-1.42	-2.30
6045		-1.42	-0.95
30497		-1.42	
9319		-1.42	0.29
33325		-1.42	0.57
2727		-1.42	-0.40
5653		-1.42	-1.23
7042		-1.42	-1.28
	13870	-1.42	-0.38
6930		-1.42	1.54
33094		-1.42	-1.13
7445		-1.42	-0.38
14745		-1.42	-0.47
7234		-1.42	-1.18
2979		-1.42	-1.31
30159		-1.42	-1.17
2945		-1.42	-0.89
14940		-1.42	-2.51
32464		-1.43	-0.75
15911		-1.43	-2.11
2712		-1.43	-1.15
	715	-1.43	-1.79
3257		-1.43	0.54
6699		-1.43	-0.67
31437		-1.43	-1.62
5718		-1.43	-1.61
17190		-1.43	0.95
15446		-1.43	-1.41
12818		-1.43	0.07
6193		-1.43	-0.55
32552		-1.43	-1.15
2974		-1.43	-0.84
1848		-1.43	-0.65
32036		-1.44	-1.20
4001		-1.44	-0.16
1078		-1.44	-0.42
13001		-1.44	-2.62
17210		-1.44	1.64
33201		-1.44	-0.96
8153		-1.44	-0.16
14231		-1.44	-0.74
13176		-1.44	-2.75

13420		-1.44	-0.63
4237		-1.44	-2.15
2099		-1.44	-0.67
33258		-1.44	-1.17
32220		-1.44	-1.00
3857		-1.45	-0.66
13387		-1.45	-0.52
10392		-1.45	-0.36
15084		-1.45	0.57
9305		-1.45	-0.17
5191		-1.45	-1.68
7583		-1.45	-0.92
7697		-1.45	-0.23
9415		-1.45	-0.23
17800		-1.45	0.44
3349		-1.45	-0.96
6839		-1.45	-1.73
14711		-1.45	-1.44
31367		-1.45	-0.40
13296		-1.45	-0.20
5527		-1.46	-0.78
17341		-1.46	0.79
11412		-1.46	-0.75
13544		-1.46	-1.38
2944		-1.46	-0.86
9398		-1.46	-1.18
	16126	-1.46	0.58
3457		-1.46	-0.99
9009		-1.46	-2.45
32373		-1.46	2.21
4860		-1.46	-1.88
3305		-1.46	-0.58
13225		-1.46	-2.44
11927		-1.46	-0.66
32491		-1.46	-1.06
31125		-1.47	-1.44
1732		-1.47	-0.51
12986		-1.47	-0.10
3680		-1.47	-0.68
12913		-1.47	-0.14
4720		-1.47	-1.63
8201		-1.47	-1.64
9075		-1.47	0.52
9946		-1.47	0.82
1651		-1.47	-1.31

11151		-1.47	-2.55
5871		-1.47	-0.84
1077		-1.47	-0.83
10580		-1.47	-1.02
	10080	-1.47	-0.41
5190		-1.48	-1.47
6447		-1.48	-0.90
17520		-1.48	-0.72
6870		-1.48	0.68
8867		-1.48	0.29
7041		-1.48	-1.92
1648		-1.48	-0.77
14560		-1.48	-0.41
15483		-1.48	-1.17
14963		-1.48	-1.16
8745		-1.48	1.85
6955		-1.48	-1.81
2720		-1.48	-0.71
11723		-1.48	-1.43
7861		-1.48	-1.42
16957		-1.48	0.00
14304		-1.48	-0.73
12768		-1.49	0.63
31645		-1.49	-0.52
5538		-1.49	-0.77
32758		-1.49	-0.96
9539		-1.49	-1.07
14044		-1.49	-0.51
	20124	-1.49	-1.62
11183		-1.49	-0.87
5210		-1.49	-0.75
13475		-1.49	-2.11
18625		-1.50	-2.22
	1172	-1.50	0.00
9424		-1.50	-0.10
	11304	-1.50	-0.15
5669		-1.50	-1.68
8421		-1.50	2.09
7698		-1.50	-0.72
32278		-1.50	-1.52
32693		-1.50	-1.51
	15583	-1.50	-1.41
10562		-1.50	-0.70
15111		-1.51	-0.16
6225		-1.51	-1.45

33006		-1.51	-2.40
10069		-1.51	-1.84
15904		-1.51	0.05
6753		-1.51	-0.81
12396		-1.51	-1.02
	13868	-1.51	-0.17
13875		-1.51	-1.17
8687		-1.51	-0.10
2985		-1.51	-0.47
6939		-1.51	0.01
1978		-1.52	-1.14
1862		-1.52	-0.88
33288		-1.52	-1.53
30330		-1.52	-1.29
32474		-1.52	-0.42
16732		-1.52	-1.18
32383		-1.52	-1.47
14857		-1.52	-1.17
5039		-1.52	-2.50
31635		-1.53	-0.16
	13925	-1.53	-0.78
	5151	-1.53	-0.76
2747		-1.53	-0.46
6708		-1.53	-0.64
2560		-1.53	-0.30
7008		-1.53	-0.64
12645		-1.53	-2.21
4209		-1.53	-2.03
7759		-1.54	0.97
	15233	-1.54	-0.76
1453		-1.54	-1.63
13892		-1.54	-0.28
17759		-1.54	-0.43
32952		-1.54	-1.31
7261		-1.54	-0.85
10755		-1.54	-0.50
	9206	-1.54	-0.90
13229		-1.54	-0.22
17309		-1.54	-0.89
5353		-1.55	0.42
18176		-1.55	-1.27
8634		-1.55	0.18
	14958	-1.55	-0.80
8790		-1.55	1.20
7182		-1.55	-1.34

32028		-1.55	-1.03
2070		-1.55	0.00
13088		-1.55	-0.46
1057		-1.55	-1.23
11086		-1.55	-1.11
12839		-1.55	-1.06
10746		-1.55	0.58
17836		-1.55	0.49
8002		-1.55	-1.34
14914		-1.55	-0.63
	423	-1.56	-2.02
18243		-1.56	-1.20
4721		-1.56	-2.45
33135		-1.56	-0.76
5630		-1.56	-0.20
31111		-1.56	-0.67
18531		-1.56	-1.11
30161		-1.56	-1.77
7207		-1.56	-1.15
16982		-1.57	-1.30
31543		-1.57	-1.13
14895		-1.57	-0.62
	17690	-1.57	-1.15
13287		-1.57	-1.16
13123		-1.57	-2.76
8415		-1.57	-0.81
	20365	-1.57	0.26
10960		-1.57	-2.42
30019		-1.57	-1.08
14676		-1.57	-1.72
	7446	-1.58	-0.04
3570		-1.58	-1.42
3694		-1.58	-0.18
2054		-1.58	-1.07
2216		-1.58	-0.13
4824		-1.58	-1.18
10682		-1.58	-1.12
4536		-1.59	-0.04
5719		-1.59	-2.01
14220		-1.59	
9967		-1.59	-1.93
8968		-1.59	0.30
9665		-1.59	-1.60
4605		-1.60	-1.30
4373		-1.60	-1.07

12909		-1.60	-0.10
14076		-1.60	0.07
8094		-1.60	-2.04
11791		-1.60	-0.36
6382		-1.60	-1.15
9101		-1.60	-0.99
8193		-1.60	-1.47
31447		-1.60	-0.48
9338		-1.60	-0.49
7046		-1.60	-2.12
13375		-1.61	0.60
11970		-1.61	-0.85
2988		-1.61	-0.50
6845		-1.61	-3.20
8850		-1.61	-1.88
	1530	-1.61	0.25
13278		-1.61	-1.80
31868		-1.62	-0.99
2019		-1.62	-1.20
5999		-1.62	-0.75
7497		-1.62	-0.59
13144		-1.62	-0.35
11928		-1.62	-1.11
31191		-1.62	0.00
13300		-1.62	-0.09
4554		-1.62	-1.03
3600		-1.62	-0.61
4974		-1.63	-0.62
4528		-1.63	-1.19
10305		-1.63	0.98
8494		-1.63	0.27
14023		-1.63	-1.88
2049		-1.63	-1.71
6612		-1.63	-3.07
8582		-1.63	1.06
17380		-1.63	0.10
8679		-1.63	-0.72
6380		-1.63	-1.56
13082		-1.63	-3.03
7424		-1.63	-3.54
33083		-1.63	-0.26
8844		-1.63	-0.65
1347		-1.64	0.16
33149		-1.64	1.00
7985		-1.64	-2.30

11395		-1.64	-0.95
16705		-1.64	-1.80
7602		-1.64	-1.03
10955		-1.64	-1.58
10565		-1.64	-1.99
32447		-1.64	-0.46
32066		-1.64	-0.86
14647		-1.65	-1.12
31327		-1.65	-1.52
2261		-1.65	-0.69
6723		-1.65	-0.14
11030		-1.65	-1.97
13579		-1.65	-1.29
11648		-1.65	-1.05
1322		-1.65	-2.40
1345		-1.65	-0.14
	16649	-1.65	-2.53
12204		-1.65	-1.94
31529		-1.65	-1.11
1273		-1.65	-1.93
3346		-1.65	-0.41
4569		-1.65	-2.22
14846		-1.66	-1.27
14897		-1.66	-0.63
5382		-1.66	-1.88
32171		-1.66	-0.79
12769		-1.66	-1.30
4886		-1.66	-1.87
6899		-1.66	-0.99
9117		-1.66	-1.34
2072		-1.66	-0.60
12244		-1.67	-1.03
6104		-1.67	-0.98
9481		-1.67	-1.17
12630		-1.67	-2.40
	16210	-1.67	-0.82
5889		-1.67	-0.55
6665		-1.67	-2.09
13527		-1.67	-0.91
2960		-1.67	
5033		-1.67	-2.43
17100		-1.68	-0.36
6339		-1.68	-0.93
6890		-1.68	-1.32
15355		-1.68	-0.63

2711		-1.68	-0.91
15669		-1.68	-0.23
4764		-1.68	-0.05
4996		-1.68	-0.91
17566		-1.68	-1.49
7293		-1.69	-0.85
13990		-1.69	-1.30
8160		-1.69	-2.40
15379		-1.69	-1.08
2987		-1.69	-0.43
	8156	-1.69	-0.52
9106		-1.69	-1.62
1234		-1.69	-2.59
31865		-1.69	-1.87
5813		-1.70	-1.84
6056		-1.70	2.26
8583		-1.70	0.06
5020		-1.70	-1.52
	8137	-1.70	-0.72
18642		-1.70	-0.77
16840		-1.70	0.73
10721		-1.70	-0.89
11303		-1.70	-0.83
17255		-1.70	-1.66
2718		-1.70	-1.26
5380		-1.70	-1.20
11820		-1.71	-0.78
15749		-1.71	-0.07
32468		-1.71	-0.60
12758		-1.71	-0.52
31973		-1.71	-0.94
6503		-1.71	-0.86
	14458	-1.71	-1.35
14025		-1.71	-1.12
9488		-1.71	-1.99
1675		-1.71	-1.38
4603		-1.71	-1.83
8289		-1.72	-1.56
31621		-1.72	-0.88
32705		-1.72	-0.55
14962		-1.72	-0.97
11634		-1.72	-1.17
	16128	-1.72	-0.85
	17554	-1.73	-1.40
7241		-1.73	0.33

3001		-1.73	-0.17
9151		-1.73	0.70
10677		-1.73	-1.95
6614		-1.73	-2.03
30143		-1.74	-0.85
1109		-1.74	-1.57
31760		-1.74	-0.78
10445		-1.74	-1.25
6207		-1.74	-1.43
32161		-1.74	1.06
8924		-1.74	0.39
11697		-1.74	-0.32
13031		-1.74	-1.08
5017		-1.75	-2.16
10336		-1.75	-1.30
8264		-1.75	-0.98
32248		-1.75	-0.66
32192		-1.75	-0.89
8378		-1.76	-1.16
	16416	-1.76	-0.66
	19624	-1.76	0.36
	7409	-1.76	-0.31
5478		-1.76	-0.12
14395		-1.76	-1.68
31258		-1.76	-1.34
1869		-1.77	-1.00
10421		-1.77	-1.16
13722		-1.77	-0.18
4167		-1.77	-1.95
5953		-1.77	-1.91
18811		-1.77	-2.09
33305		-1.77	-1.36
14992		-1.77	-0.88
5227		-1.78	-1.43
10327		-1.78	-1.59
9337		-1.78	-2.02
8176		-1.78	0.03
10863		-1.79	-1.43
14910		-1.79	0.54
18550		-1.79	-2.31
18345		-1.79	-0.32
6000		-1.79	-0.01
14946		-1.79	-0.96
31057		-1.79	-0.68
2076		-1.79	-1.42

4881		-1.79	-1.21
32733		-1.79	-1.74
11124		-1.80	-2.05
9093		-1.80	-0.78
6618		-1.80	0.02
32335		-1.81	-1.82
32179		-1.81	-1.78
9624		-1.81	-0.68
10469		-1.81	-1.65
17209		-1.81	-0.19
8993		-1.81	-1.26
17018		-1.81	1.58
9406		-1.82	-1.27
13526		-1.82	0.10
5526		-1.82	-2.26
31816		-1.82	-1.80
13533		-1.82	-0.26
15628		-1.82	-1.02
9302		-1.83	-0.15
7006		-1.83	-1.16
1162		-1.83	-0.27
	16096	-1.83	-0.59
4029		-1.84	0.00
3996		-1.84	-1.08
10719		-1.85	1.54
8053		-1.85	0.36
14901		-1.85	-0.05
15387		-1.85	-2.16
6017		-1.86	0.19
32594		-1.86	-2.10
32417		-1.87	-1.03
1900		-1.87	-1.84
15720		-1.87	0.27
4700		-1.88	-1.90
1676		-1.88	-1.58
6015		-1.88	-1.43
13472		-1.88	-1.24
9088		-1.88	0.32
8756		-1.88	0.14
10825		-1.88	0.31
2071		-1.88	-0.68
30489		-1.89	0.97
3506		-1.89	-1.22
4482		-1.89	-1.62
3199		-1.89	-1.36

3306		-1.89	-3.05
6847		-1.89	-0.92
	14311	-1.89	-0.81
12200		-1.90	-1.65
	9327	-1.90	1.09
6988		-1.90	0.02
1112		-1.90	-2.40
6253		-1.91	-0.34
14217		-1.91	
30122		-1.91	-1.42
15032		-1.91	-1.97
7160		-1.91	-0.51
1656		-1.91	
13772		-1.92	-0.63
10777		-1.92	-1.25
4178		-1.92	-1.73
32581		-1.92	-1.90
6877		-1.92	-0.87
8376		-1.92	-3.30
12238		-1.93	-1.54
10491		-1.93	1.25
1475		-1.93	-2.22
	13936	-1.94	-1.58
6041		-1.94	-0.99
1577		-1.94	-1.29
5325		-1.94	-0.95
4602		-1.95	-1.75
11416		-1.95	-2.21
1212		-1.95	-1.13
8887		-1.96	-0.05
17841		-1.96	-2.53
7187		-1.96	-2.41
15383		-1.97	-1.85
14365		-1.97	0.34
4090		-1.97	-0.10
32467		-1.98	-0.46
9392		-1.98	-0.99
11899		-1.98	-1.36
33207		-1.98	-0.28
9632		-1.98	0.11
30097		-1.98	-2.45
12590		-1.98	-1.54
5061		-1.98	-1.74
30458		-1.99	-2.96
13643		-1.99	-0.45

3173		-1.99	-1.94
8730		-1.99	-0.18
6141		-1.99	-1.80
5506		-1.99	-0.27
10513		-1.99	-0.75
14939		-2.00	-2.28
6173		-2.00	-1.49
7269		-2.01	-1.13
	16059	-2.02	-1.24
1839		-2.02	-0.65
4577		-2.02	1.19
7914		-2.02	-1.01
	2356	-2.02	-1.62
6720		-2.03	-1.53
5792		-2.03	-1.39
6061		-2.03	-0.57
5657		-2.03	-1.53
	13887	-2.03	-1.22
14619		-2.03	-2.06
13465		-2.04	-1.72
6153		-2.04	0.31
8426		-2.04	-1.89
13570		-2.04	-0.95
7023		-2.04	-1.80
5172		-2.05	-0.98
14073		-2.05	-3.03
12384		-2.06	-1.61
4982		-2.06	-1.15
	10097	-2.07	-0.69
5684		-2.07	-1.46
33125		-2.07	-0.04
8489		-2.07	-0.68
15646		-2.08	-1.09
10788		-2.08	0.10
12377		-2.08	-1.17
2767		-2.08	-1.02
5877		-2.09	-2.46
12740		-2.09	-0.20
31537		-2.09	-1.21
32279		-2.09	-2.28
2812		-2.10	-1.53
12997		-2.10	-1.02
13625		-2.10	-2.03
18157		-2.11	-0.69
10203		-2.12	-1.47

12976		-2.12	-0.44
32132		-2.12	-1.52
1841		-2.12	-1.05
	15592	-2.13	-2.09
	18630	-2.13	-1.41
	8154	-2.13	-0.87
32570		-2.13	-0.66
6169		-2.14	0.19
6694		-2.14	-2.08
11284		-2.14	-0.12
12928		-2.14	-2.03
6869		-2.15	-0.35
14968		-2.15	-1.57
5366		-2.16	-2.62
6143		-2.16	-2.14
5169		-2.16	-1.78
7334		-2.16	-1.81
	18629	-2.16	-0.59
3875		-2.18	-0.42
2759		-2.18	-0.85
4832		-2.18	-1.53
17161		-2.19	-2.12
16903		-2.19	-0.07
33324		-2.19	0.10
3125		-2.19	-0.68
10645		-2.20	-0.36
32499		-2.20	-0.58
33465		-2.22	-2.07
10986		-2.22	-2.20
7028		-2.24	-1.07
10385		-2.24	-1.85
15816		-2.25	-0.36
	7335	-2.25	-2.28
2062		-2.25	-0.08
3151		-2.25	-1.60
3689		-2.26	-2.00
10793		-2.26	-0.06
17136		-2.27	-0.26
17492		-2.28	-1.58
10495		-2.28	-0.64
15057		-2.29	-1.21
1259		-2.29	0.07
2291		-2.29	-0.84
14435		-2.29	-0.39
2042		-2.30	-0.41

8183		-2.30	-1.95
33292		-2.30	-1.31
11081		-2.30	-2.29
3817		-2.31	0.35
16874		-2.31	-2.03
	8157	-2.31	-0.54
1252		-2.32	-1.44
10396		-2.33	-1.24
14657		-2.34	-1.68
8614		-2.34	-0.36
3661		-2.34	-0.91
7107		-2.35	-0.98
8949		-2.35	-0.82
14891		-2.35	-0.51
17298		-2.36	0.07
17183		-2.36	-2.34
15599		-2.36	-0.84
1652		-2.37	-2.04
14080		-2.38	-1.60
7518		-2.38	-1.41
	2074	-2.38	-1.12
1429		-2.38	-2.11
6091		-2.38	-0.49
2063		-2.38	-1.24
8851		-2.39	-0.96
7716		-2.40	-2.15
10800		-2.41	-1.79
	17686	-2.41	-1.51
8580		-2.42	-1.56
17896		-2.43	-2.35
7508		-2.43	-0.65
8827		-2.46	1.03
9948		-2.47	-1.76
10359		-2.49	-1.84
6956		-2.49	-1.38
14126		-2.50	-1.11
15884		-2.52	-0.32
15737		-2.52	-0.29
9738		-2.53	-2.53
	8833	-2.55	-1.71
16742		-2.55	-1.88
14258		-2.57	-0.40
10975		-2.59	-2.48
31545		-2.59	0.15
14351		-2.60	0.14

13109		-2.61	-1.72
17041		-2.62	-2.02
12361		-2.62	-1.86
10600		-2.65	-1.29
13779		-2.66	-1.34
12348		-2.67	-0.84
9364		-2.67	-0.99
	16589	-2.68	-1.39
	10534	-2.70	0.39
10576		-2.72	-2.32
3131		-2.73	-1.32
1874		-2.75	-0.75
8293		-2.76	-3.83
9748		-2.78	-0.45
3563		-2.78	1.57
7734		-2.81	-1.39
7803		-2.84	-2.16
18497		-2.85	-1.68
15864		-2.85	0.12
10375		-2.86	-2.00
15630		-2.87	-0.05
10078		-2.87	-2.51
13044		-2.89	-2.11
7065		-2.90	-0.79
14938		-2.93	-2.23
6817		-2.93	-0.62
9124		-2.98	-0.50
4353		-3.04	-1.62
31353		-3.06	-2.53
15742		-3.07	-1.57
1130		-3.12	-0.87
7619		-3.13	-2.62
5827		-3.14	-1.91
	1143	-3.14	-1.15
5680		-3.15	-1.86
16973		-3.15	0.49
18375		-3.17	-1.56
5060		-3.19	-2.97
32139		-3.20	-3.39
11200		-3.23	-1.37
10279		-3.24	-2.38
11006		-3.24	-1.48
7726		-3.31	-2.03
5163		-3.32	-1.25
32105		-3.32	-1.72

8669		-3.37	-1.98
10160		-3.41	-0.46
8815		-3.42	-2.26
10377		-3.42	-2.46
7274		-3.44	-1.79
3241		-3.47	-1.32
7486		-3.49	-2.38
4124		-3.50	-2.66
14187		-3.52	-1.04
	271	-3.53	-1.83
1821		-3.57	-1.91
6393		-3.73	-3.69
14564		-3.75	0.25
5576		-3.75	-1.91
15881		-4.02	-1.22
7417		-4.28	-2.65
31657		-4.38	-3.71
12297		-4.47	-2.77
2762		-4.53	-0.36
4432		-4.66	-3.65
12437		-4.72	-1.90
	18088	-4.96	-2.70
7185		-5.22	-3.16
18492		-5.70	-3.16
