# Supplementary material for: A Quantitative RNAi Screen for JNK Modifiers Identifies Pvr as a Novel Regulator of Drosophila Immune Signaling
Source: PLoS Pathog. 2009 Nov 6;5(11):e1000655. doi: 10.1371/journal.ppat.1000655 (PMC2766254; doi:10.1371/journal.ppat.1000655)
Supplement: Table S2 — z-score analysis of dsRNA-mediated depletion of enhancers of PGN-induced dJNK phosphorylation. In-cell Western z-scores were calculated from P-JNK:f-actin values from S2 cells incubated with 15,683 dsRNAs and treated with PGN for 15 min. dsRNAs that modified P-JNK:f-actin z-scores below 1.96 (95% CI) are ordered from smallest to highest z-score. The fold change in dJNK phosphorylation relative to the plate median is shown alongside the z-score values. Each dsRNA is identified by its symbol and Celera Genome (CG) number or by its Heidelberg Drosophila Consortium identification number (HCDID). (0.33 MB DOC) [file ppat.1000655.s003.doc]

Table S2 - Enhancers of 15 min PGN-induced P-JNK.

| Symbol | FBGN | CG | HDCID | Function | 15min z-score | 15min ΔP-JNK |
| --- | --- | --- | --- | --- | --- | --- |
| Tak1 | 26323 | 18492 |  | Immune signaling | -5.70 | 0.33 |
| CG7185 | 35872 | 7185 |  | RNA binding | -5.22 | 0.40 |
| HDC18088 |  |  | 18088 | Unknown | -4.96 | 0.63 |
| raw | 3209 | 12437 |  | Signaling | -4.72 | 0.77 |
| PGRP-LC | 35976 | 4432 |  | Immune signaling | -4.66 | 0.21 |
| ush | 3963 | 2762 |  | DNA binding | -4.53 | 0.61 |
| dFadd | 38928 | 12297 |  | Immune signaling | -4.47 | 0.52 |
| PNUTS | 31291 | 31657 |  | Signaling | -4.38 | 0.69 |
| Tab2 | 34431 | 7417 |  | Immune signaling | -4.28 | 0.62 |
| CG15881 | 36909 | 15881 |  | Unknown | -4.02 | 0.47 |
| imd | 13983 | 5576 |  | Immune signaling | -3.75 | 0.60 |
| CG14564 | 37131 | 14564 |  | Unknown | -3.75 | 0.87 |
| CG6393 | 34685 | 6393 |  | Unknown | -3.73 | 0.47 |
| RpL31 | 25286 | 1821 |  | Translation | -3.57 | 0.62 |
| HDC00271 |  |  | 271 | Unknown | -3.53 | 0.70 |
| CG14187 | 36938 | 14187 |  | Unknown | -3.52 | 0.74 |
| PNUTS | 31291 | 4124 |  | Signaling | -3.50 | 0.42 |
| Dredd | 20381 | 7486 |  | Immune signaling | -3.49 | 0.25 |
| msl-2 | 5616 | 3241 |  | DNA binding | -3.47 | 0.78 |
| CG7274 | 30965 | 7274 |  | DNA binding | -3.44 | 0.59 |
| Hrb27C | 4838 | 10377 |  | RNA binding | -3.42 | 0.57 |
| Sin3A | 22764 | 8815 |  | DNA binding | -3.42 | 0.50 |
| ImpL3 | 1258 | 10160 |  | Metabolism | -3.41 | 0.60 |
| crc | 370 | 8669 |  | DNA binding | -3.37 | 0.77 |
| CG32105 | 52105 | 32105 |  | DNA binding | -3.32 | 0.43 |
| TfIIA-S | 13347 | 5163 |  | DNA binding | -3.32 | 0.40 |
| RpL11 | 13325 | 7726 |  | Translation | -3.31 | 0.69 |
| CG11006 | 27534 | 11006 |  | Unknown | -3.24 | 0.73 |
| Rm62 | 3261 | 10279 |  | RNA binding | -3.24 | 0.40 |
| CG11200 | 34500 | 11200 |  | Metabolism | -3.23 | 0.62 |
| Sox21b | 42630 | 32139 |  | DNA binding | -3.20 | 0.74 |
| CG5060 | 38780 | 5060 |  | Unknown | -3.19 | 0.79 |
| CG18375 | 34606 | 18375 |  | Unknown | -3.17 | 0.58 |
| msn | 10909 | 16973 |  | Immune signaling | -3.15 | 0.70 |
| bsk | 229 | 5680 |  | Immune signaling | -3.15 | 0.58 |
| HDC01143 |  |  | 1143 | Unknown | -3.14 | 0.88 |
| RpL37A | 28696 | 5827 |  | Translation | -3.14 | 0.62 |
| Pros54 | 15283 | 7619 |  | Proteolysis | -3.13 | 0.68 |
| scrt | 4880 | 1130 |  | DNA binding | -3.12 | 0.53 |
| CG15742 | 30462 | 15742 |  | Unknown | -3.07 | 0.72 |
| CG31353 | 51353 | 31353 |  | Unknown | -3.06 | 0.84 |
| hep | 10303 | 4353 |  | Immune signaling | -3.04 | 0.46 |
| eIF-3p40 | 22023 | 9124 |  | RNA binding | -2.98 | 0.71 |
| foi | 24236 | 6817 |  | Other | -2.93 | 0.72 |
| crol | 20309 | 14938 |  | DNA binding | -2.93 | 0.86 |
| CG7065 | 30091 | 7065 |  | Unknown | -2.90 | 0.61 |
| CG13044 | 36599 | 13044 |  | Unknown | -2.89 | 0.80 |
| Prat2 | 41194 | 10078 |  | Metabolism | -2.87 | 0.97 |
| CG15630 | 31627 | 15630 |  | Other | -2.87 | 0.62 |
| CG10375 | 39116 | 10375 |  | Other | -2.86 | 0.74 |
| CG15864 | 40528 | 15864 |  | Metabolism | -2.85 | 0.73 |
| spen | 16977 | 18497 |  | RNA binding | -2.85 | 0.75 |
| z | 4050 | 7803 |  | DNA binding | -2.84 | 0.71 |
| shn | 3396 | 7734 |  | DNA binding | -2.81 | 0.57 |
| CG3563 | 38259 | 3563 |  | Unknown | -2.78 | 0.87 |
| bel | 171 | 9748 |  | RNA binding | -2.78 | 0.64 |
| Iap2 | 15247 | 8293 |  | Immune signaling | -2.76 | 0.30 |
| CG1874 | 33425 | 1874 |  | Unknown | -2.75 | 0.86 |
| Duox | 31464 | 3131 |  | Other | -2.73 | 0.74 |
| CG10576 | 35630 | 10576 |  | Unknown | -2.72 | 0.94 |
| HDC10534 |  |  | 10534 | Unknown | -2.70 | 0.76 |
| HDC16589 |  |  | 16589 | Unknown | -2.68 | 0.73 |
| Treh | 3748 | 9364 |  | Metabolism | -2.67 | 0.94 |
| Sh | 3380 | 12348 |  | Other | -2.67 | 0.49 |
| CG13779 | 40954 | 13779 |  | Unknown | -2.66 | 0.83 |
| CG10600 | 32717 | 10600 |  | Unknown | -2.65 | 0.78 |
| CG12361 | 35292 | 12361 |  | DNA binding | -2.62 | 0.57 |
| CG17041 | 33822 | 17041 |  | Unknown | -2.62 | 0.99 |
| tai | 41092 | 13109 |  | Signaling | -2.61 | 0.89 |
| CG14351 | 31349 | 14351 |  | Unknown | -2.60 | 0.47 |
| CG31545 | 51545 | 31545 |  | Unknown | -2.59 | 0.81 |
| Ptp69D | 14007 | 10975 |  | Signaling | -2.59 | 0.95 |
| CG14258 | 39482 | 14258 |  | Unknown | -2.57 | 0.96 |
| CG16742 | 34529 | 16742 |  | Unknown | -2.55 | 0.92 |
| HDC08833 |  |  | 8833 | Unknown | -2.55 | 0.78 |
| Mkk4 | 24326 | 9738 |  | Immune signaling | -2.53 | 0.62 |
| CG15737 | 30353 | 15737 |  | DNA binding | -2.52 | 0.79 |
| CG15884 | 39481 | 15884 |  | Unknown | -2.52 | 0.97 |
| CG14126 | 36223 | 14126 |  | Unknown | -2.50 | 0.93 |
| Lcp65Ac | 20642 | 6956 |  | Unknown | -2.49 | 0.89 |
| CG10359 | 35452 | 10359 |  | Unknown | -2.49 | 0.73 |
| CG9948 | 35721 | 9948 |  | Unknown | -2.47 | 0.89 |
| Ance | 12037 | 8827 |  | Metabolism | -2.46 | 0.82 |
| ato | 10433 | 7508 |  | DNA binding | -2.43 | 0.82 |
| CG17896 | 23537 | 17896 |  | Metabolism | -2.43 | 0.83 |
| bhr | 35773 | 8580 |  | Unknown | -2.42 | 0.65 |
| HDC17686 |  |  | 17686 | Unknown | -2.41 | 0.93 |
| Rca1 | 17551 | 10800 |  | Other | -2.41 | 0.85 |
| CG7716 | 35800 | 7716 |  | Cytoskeletal | -2.40 | 0.88 |
| CG8851 | 31546 | 8851 |  | Cytoskeletal | -2.39 | 0.72 |
| CG2063 | 33400 | 2063 |  | Unknown | -2.38 | 0.69 |
| CG6091 | 36180 | 6091 |  | Proteolysis | -2.38 | 0.67 |
| Mef2 | 11656 | 1429 |  | DNA binding | -2.38 | 0.62 |
| HDC02074 |  |  | 2074 | Unknown | -2.38 | 0.94 |
| CG7518 | 38108 | 7518 |  | Unknown | -2.38 | 0.70 |
| Mkp3 | 36844 | 14080 |  | Signaling | -2.38 | 0.78 |
| lectin-46Cb | 40092 | 1652 |  | Unknown | -2.37 | 0.86 |
| CG15599 | 30667 | 15599 |  | Unknown | -2.36 | 0.90 |
| MED30 | 35149 | 17183 |  | DNA binding | -2.36 | 0.63 |
| CG17298 | 38879 | 17298 |  | Unknown | -2.36 | 0.97 |
| CG14891 | 38445 | 14891 |  | Unknown | -2.35 | 1.06 |
| CG8949 | 30812 | 8949 |  | Unknown | -2.35 | 0.83 |
| up | 4169 | 7107 |  | Cytoskeletal | -2.35 | 0.65 |
| RpL23 | 10078 | 3661 |  | Translation | -2.34 | 0.70 |
| Neos | 24542 | 8614 |  | Unknown | -2.34 | 0.73 |
| CG14657 | 37282 | 14657 |  | Unknown | -2.34 | 0.74 |
| CG10396 | 33020 | 10396 |  | Metabolism | -2.33 | 0.75 |
| Ccp84Ab | 4782 | 1252 |  | Unknown | -2.32 | 0.87 |
| HDC08157 |  |  | 8157 | Unknown | -2.31 | 0.81 |
| Vm32E | 14076 | 16874 |  | Unknown | -2.31 | 0.94 |
| CG3817 | 38275 | 3817 |  | Unknown | -2.31 | 0.79 |
| plexA | 25741 | 11081 |  | Signaling | -2.30 | 0.67 |
| CG33292 | 53292 | 33292 |  | Unknown | -2.30 | 0.87 |
| Khc-73 | 19968 | 8183 |  | Cytoskeletal | -2.30 | 0.74 |
| CG2042 | 32944 | 2042 |  | Unknown | -2.30 | 0.91 |
| CG14435 | 29911 | 14435 |  | Unknown | -2.29 | 0.62 |
| CG2291 | 33279 | 2291 |  | Unknown | -2.29 | 0.78 |
| CG1259 | 35513 | 1259 |  | Unknown | -2.29 | 0.81 |
| CG15057 | 30908 | 15057 |  | Unknown | -2.29 | 0.86 |
| CG10495 | 32750 | 10495 |  | Metabolism | -2.28 | 0.60 |
| mib2 | 32742 | 17492 |  | Signaling | -2.28 | 0.79 |
| Rbp1 | 10252 | 17136 |  | RNA binding | -2.27 | 0.72 |
| CG10793 | 29656 | 10793 |  | Other | -2.26 | 0.82 |
| CG3689 | 35987 | 3689 |  | RNA binding | -2.26 | 0.74 |
| Rbp9 | 10263 | 3151 |  | RNA binding | -2.25 | 0.83 |
| Cyp4e1 | 15034 | 2062 |  | Metabolism | -2.25 | 0.83 |
| HDC07335 |  |  | 7335 | Unknown | -2.25 | 0.89 |
| CG15816 | 30866 | 15816 |  | Unknown | -2.25 | 0.94 |
| msl-1 | 5617 | 10385 |  | DNA binding | -2.24 | 0.74 |
| CG7028 | 27587 | 7028 |  | Unknown | -2.24 | 0.86 |
| g | 1087 | 10986 |  | Other | -2.22 | 0.76 |
| CG33465 | 53465 | 33465 |  | Unknown | -2.22 | 0.81 |
| CG32499 | 52499 | 32499 |  | Metabolism | -2.20 | 0.67 |
| lama | 16031 | 10645 |  | Other | -2.20 | 0.92 |
| l(1)G0060 | 29797 | 3125 |  | Unknown | -2.19 | 0.63 |
| CG33324 | 53324 | 33324 |  | Unknown | -2.19 | 0.81 |
| CG16903 | 40394 | 16903 |  | DNA binding | -2.19 | 0.77 |
| grp | 11598 | 17161 |  | Signaling | -2.19 | 0.75 |
| cnn | 13765 | 4832 |  | Other | -2.18 | 1.01 |
| w | 3996 | 2759 |  | Metabolism | -2.18 | 0.90 |
| CG3875 | 34740 | 3875 |  | RNA binding | -2.18 | 0.89 |
| HDC18629 |  |  | 18629 | Unknown | -2.16 | 0.80 |
| Sug | 36191 | 7334 |  | DNA binding | -2.16 | 0.81 |
| GckIII | 38477 | 5169 |  | Signaling | -2.16 | 0.78 |
| Pep | 4401 | 6143 |  | RNA binding | -2.16 | 0.94 |
| CG5366 | 27568 | 5366 |  | DNA binding | -2.16 | 0.84 |
| CG14968 | 35431 | 14968 |  | Unknown | -2.15 | 0.97 |
| FucTA | 36485 | 6869 |  | Metabolism | -2.15 | 0.89 |
| CG12928 | 33432 | 12928 |  | Unknown | -2.14 | 0.87 |
| CG11284 | 30056 | 11284 |  | Metabolism | -2.14 | 0.69 |
| CG6694 | 35900 | 6694 |  | Unknown | -2.14 | 0.87 |
| CG6169 | 36534 | 6169 |  | RNA binding | -2.14 | 0.60 |
| CG32570 | 52570 | 32570 |  | Unknown | -2.13 | 1.03 |
| HDC08154 |  |  | 8154 | Unknown | -2.13 | 0.87 |
| HDC18630 |  |  | 18630 | Unknown | -2.13 | 0.79 |
| HDC15592 |  |  | 15592 | Unknown | -2.13 | 0.88 |
| Tango10 | 30330 | 1841 |  | Other | -2.12 | 0.82 |
| CG32132 | 52132 | 32132 |  | Unknown | -2.12 | 0.87 |
| CG12976 | 37053 | 12976 |  | Unknown | -2.12 | 0.95 |
| xl6 | 28554 | 10203 |  | RNA binding | -2.12 | 0.89 |
| CG18157 | 30563 | 18157 |  | Unknown | -2.11 | 1.04 |
| CG13625 | 39210 | 13625 |  | Unknown | -2.10 | 0.74 |
| CG12997 | 30831 | 12997 |  | Unknown | -2.10 | 1.02 |
| CG2812 | 34931 | 2812 |  | Unknown | -2.10 | 0.87 |
| dro2 | 52279 | 32279 |  | Other | -2.09 | 0.97 |
| CG31537 | 51537 | 31537 |  | Unknown | -2.09 | 0.93 |
| RpL28 | 35422 | 12740 |  | Translation | -2.09 | 0.64 |
| CG5877 | 30625 | 5877 |  | Unknown | -2.09 | 0.98 |
| CG2767 | 37537 | 2767 |  | Metabolism | -2.08 | 0.92 |
| CG12377 | 37168 | 12377 |  | Unknown | -2.08 | 0.99 |
| ng3 | 10295 | 10788 |  | Unknown | -2.08 | 0.81 |
| CG15646 | 30665 | 15646 |  | Unknown | -2.08 | 0.96 |
| CG8489 | 38225 | 8489 |  | Unknown | -2.07 | 0.91 |
| CG33125 | 53125 | 33125 |  | Unknown | -2.07 | 1.12 |
| Pop2 | 36239 | 5684 |  | RNA binding | -2.07 | 0.76 |
| HDC10097 |  |  | 10097 | Unknown | -2.07 | 0.84 |
| CG4982 | 36598 | 4982 |  | Unknown | -2.06 | 0.90 |
| CG12384 | 33624 | 12384 |  | Unknown | -2.06 | 0.94 |
| CG14073 | 36814 | 14073 |  | Unknown | -2.05 | 0.98 |
| CG5172 | 30830 | 5172 |  | Unknown | -2.05 | 0.84 |
| CG7023 | 39025 | 7023 |  | Proteolysis | -2.04 | 0.91 |
| spag | 15544 | 13570 |  | Unknown | -2.04 | 0.98 |
| l(2)NC136 | 33029 | 8426 |  | DNA binding | -2.04 | 0.64 |
| CG6153 | 32445 | 6153 |  | Unknown | -2.04 | 0.71 |
| CG13465 | 40809 | 13465 |  | Unknown | -2.04 | 0.97 |
| CG14619 | 31187 | 14619 |  | Proteolysis | -2.03 | 0.87 |
| HDC13887 |  |  | 13887 | Unknown | -2.03 | 0.84 |
| Scgbeta | 38042 | 5657 |  | Cytoskeletal | -2.03 | 0.98 |
| mip120 | 33846 | 6061 |  | DNA binding | -2.03 | 0.74 |
| CG5792 | 32455 | 5792 |  | Unknown | -2.03 | 0.97 |
| UbcD2 | 15320 | 6720 |  | Other | -2.03 | 0.83 |
| HDC02356 |  |  | 2356 | Unknown | -2.02 | 0.85 |
| CG7914 | 30995 | 7914 |  | Unknown | -2.02 | 0.92 |
| CG4577 | 31306 | 4577 |  | Unknown | -2.02 | 1.02 |
| CG1839 | 30555 | 1839 |  | Unknown | -2.02 | 0.77 |
| HDC16059 |  |  | 16059 | Unknown | -2.02 | 0.96 |
| Hel25E | 14189 | 7269 |  | RNA binding | -2.01 | 0.76 |
| kal-1 | 39155 | 6173 |  | Other | -2.00 | 0.72 |
| CG14939 | 32378 | 14939 |  | Unknown | -2.00 | 1.00 |
| CG10513 | 39311 | 10513 |  | Unknown | -1.99 | 0.87 |
| CG5506 | 36766 | 5506 |  | Unknown | -1.99 | 0.94 |
| RpL9 | 15756 | 6141 |  | Translation | -1.99 | 0.59 |
| drosha | 26722 | 8730 |  | RNA binding | -1.99 | 0.71 |
| CG3173 | 34964 | 3173 |  | Unknown | -1.99 | 0.78 |
| CG13643 | 40601 | 13643 |  | Other | -1.99 | 0.82 |
| CG30458 | 50458 | 30458 |  | Unknown | -1.99 | 0.93 |
| capt | 28388 | 5061 |  | Cytoskeletal | -1.98 | 0.72 |
| CG12590 | 37294 | 12590 |  | Unknown | -1.98 | 1.00 |
| Menl-2 | 29153 | 30097 |  | Metabolism | -1.98 | 0.81 |
| CG9632 | 38377 | 9632 |  | Unknown | -1.98 | 0.82 |
| pxb | 53207 | 33207 |  | Unknown | -1.98 | 0.85 |
| CG11899 | 14427 | 11899 |  | Metabolism | -1.98 | 0.77 |
| CG9392 | 36895 | 9392 |  | Unknown | -1.98 | 0.88 |
| CG32467 | 52467 | 32467 |  | Unknown | -1.98 | 0.96 |
| CG4090 | 38492 | 4090 |  | Other | -1.97 | 0.87 |
| CG14365 | 38177 | 14365 |  | Unknown | -1.97 | 0.83 |
| CG15383 | 31394 | 15383 |  | Unknown | -1.97 | 0.94 |
| Ssdp | 11481 | 7187 |  | DNA binding | -1.96 | 0.86 |
| CG17841 | 28480 | 17841 |  | Unknown | -1.96 | 0.83 |
