# Supplementary material for: A Quantitative RNAi Screen for JNK Modifiers Identifies Pvr as a Novel Regulator of Drosophila Immune Signaling
Source: PLoS Pathog. 2009 Nov 6;5(11):e1000655. doi: 10.1371/journal.ppat.1000655 (PMC2766254; doi:10.1371/journal.ppat.1000655)
Supplement: Table S3 — z-score analysis of dsRNA-mediated depletion of suppressors of 15 min PGN-induced dJNK phosphorylation. In-cell Western z-scores were calculated from P-JNK:f-actin values from S2 cells incubated with 15,683 dsRNAs and treated with PGN for 15 or 60 min. dsRNAs that modified 15 min PGN-induced P-JNK:f-actin z-scores above 1.96 (95% CI) are ordered from highest to lowest z-score. The fold change in dJNK phosphorylation relative to the plate median is shown alongside the z-score values for both 15 and 60 min time points. Each dsRNA is identified by its symbol and Celera Genome (CG) number or by its Heidelberg Drosophila Consortium identification number (HCDID). (1.05 MB DOC) [file ppat.1000655.s004.doc]

Table S3 - Suppressors of 15 min PGN-induced P-JNK.

| Sym | FBGN | CG | HDCID | Function | 15min  z-score | 15min  ΔP-dJNK | 60min  z-score | 60min  ΔP-dJNK |
| --- | --- | --- | --- | --- | --- | --- | --- | --- |
| key | 41205 | 16910 |  | Immune signaling | 9.06 | 2.85 | 9.23 | 3.43 |
| HDC03194 |  |  | 3194 | Unknown | 7.72 | 1.77 | -0.33 | 0.92 |
| Cka | 44323 | 7392 |  | Immune signaling | 7.70 | 2.51 | 7.20 | 2.11 |
| CG11526 | 35437 | 11526 |  | Unknown | 7.35 | 2.26 | 4.67 | 1.50 |
| HDC12197 |  |  | 12197 | Unknown | 7.11 | 1.99 | -1.10 | 1.00 |
| ird5 | 24222 | 4201 |  | Immune signaling | 6.23 | 1.76 | 7.74 | 2.18 |
| CG11799 | 36134 | 11799 |  | DNA binding | 5.89 | 1.58 | 1.56 | 0.84 |
| porin | 4363 | 6647 |  | Other | 5.88 | 1.41 | 5.50 | 1.28 |
| HDC06860 |  |  | 6860 | Unknown | 5.85 | 1.76 | -0.53 | 1.01 |
| bun | 10460 | 5461 |  | DNA binding | 5.75 | 1.54 | 2.05 | 1.20 |
| CG15732 | 30385 | 15732 |  | Other | 5.72 | 1.74 | -0.74 | 0.98 |
| drpr | 27594 | 2086 |  | Signaling | 5.56 | 1.60 |  | 1.23 |
| HLHm3 | 2609 | 8346 |  | DNA binding | 5.37 | 2.00 | 2.54 | 0.92 |
| RpII140 | 3276 | 3180 |  | DNA binding | 5.37 | 1.88 | 2.74 | 1.41 |
| pnt | 3118 | 17077 |  | DNA binding | 5.32 | 1.17 | -1.34 | 0.70 |
| CG14314 | 38581 | 14314 |  | Unknown | 5.24 | 1.50 | 1.76 | 1.34 |
| cnk | 21818 | 6556 |  | Signaling | 5.20 | 2.04 | 3.63 | 1.31 |
| Taf1 | 10355 | 17603 |  | DNA binding | 5.08 | 1.65 | 0.38 | 1.04 |
| ken | 11236 | 5575 |  | DNA binding | 5.07 | 1.35 | 3.57 | 1.19 |
| HDC02525 |  |  | 2525 | Unknown | 5.04 | 1.61 | 0.61 | 1.13 |
| CG12264 | 32393 | 12264 |  | Metabolism | 5.02 | 1.65 | -0.49 | 0.98 |
| Pvr | 32006 | 8222 |  | Signaling | 5.01 | 1.99 | 1.77 | 0.99 |
| HDC02579 |  |  | 2579 | Unknown | 5.01 | 1.51 | -0.59 | 1.01 |
| CG3403 | 33094 | 3403 |  | Unknown | 5.00 | 2.44 | 0.24 | 1.37 |
| UBL3 | 26076 | 9038 |  | Other | 4.96 | 1.72 | 6.13 | 1.29 |
| CG10936 | 34253 | 10936 |  | Unknown | 4.92 | 1.30 | 0.57 | 1.15 |
| Act5C | 42 | 4027 |  | Cytoskeleton | 4.90 | 1.37 | 4.28 | 1.42 |
| RpP1 | 3274 | 4918 |  | Translation | 4.80 | 0.77 | 1.75 | 1.15 |
| CG11848 | 39282 | 11848 |  | Unknown | 4.78 | 1.77 | -0.66 | 1.01 |
| CG15321 | 30150 | 15321 |  | Unknown | 4.76 | 1.41 | 0.71 | 1.04 |
| HDC09403 |  |  | 9403 | Unknown | 4.73 | 1.60 | 1.68 | 1.18 |
| gw | 51992 | 31992 |  | RNA binding | 4.71 | 1.40 | 0.70 | 1.00 |
| HDC12021 |  |  | 12021 | Unknown | 4.69 | 1.50 | 0.91 | 1.09 |
| CG8828 | 33740 | 8828 |  | Unknown | 4.69 | 1.23 | 1.46 | 1.11 |
| Elongin-C | 23211 | 9291 |  | RNA Binding | 4.69 | 1.45 | 0.59 | 1.11 |
| CG32681 | 52681 | 32681 |  | Unknown | 4.66 | 1.46 | 0.25 | 0.96 |
| pims | 34647 | 15678 |  | Unknown | 4.63 | 1.82 | 7.28 | 2.40 |
| dm | 472 | 10798 |  | DNA binding | 4.62 | 1.36 | 1.06 | 1.05 |
| HDC08340 |  |  | 8340 | Unknown | 4.61 | 1.33 | -0.04 | 1.00 |
| CG8954 | 19890 | 8954 |  | RNA binding | 4.59 | 1.80 | 2.12 | 1.25 |
| CG9864 | 34490 | 9864 |  | Other | 4.57 | 1.85 | -0.08 | 1.13 |
| CG13083 | 32789 | 13083 |  | Unknown | 4.57 | 1.35 | -0.89 | 0.98 |
| Mst35Ba | 13300 | 4479 |  | DNA binding | 4.57 | 1.25 | 1.52 | 1.04 |
| CG10158 | 31871 | 10158 |  | Unknown | 4.57 | 1.34 | 3.88 | 1.42 |
| RpII215 | 3277 | 1554 |  | DNA binding | 4.52 | 1.91 | 1.65 | 1.29 |
| dec-1 | 427 | 2175 |  | Other | 4.47 | 1.57 | -0.90 | 1.00 |
| Src42A | 4603 | 7873 |  | Signaling | 4.31 | 1.35 | 2.30 | 1.25 |
| HDC05820 |  |  | 5820 | Unknown | 4.24 | 1.46 | -1.12 | 0.99 |
| HDC10149 |  |  | 10149 | Unknown | 4.24 | 1.29 | 2.05 | 1.14 |
| HDC00113 |  |  | 113 | Unknown | 4.24 | 1.39 | -0.18 | 1.00 |
| HDC02195 |  |  | 2195 | Unknown | 4.23 | 1.66 | 1.40 | 1.24 |
| Acox57D-d | 34629 | 9709 |  | Metabolism | 4.23 | 1.40 | 1.57 | 0.98 |
| kto | 1324 | 8491 |  | RNA Binding | 4.23 | 1.28 | 2.05 | 1.19 |
| Pxd | 4577 | 3477 |  | Other | 4.23 | 2.20 | 0.64 | 1.51 |
| Rpb10 | 39218 | 13628 |  | DNA binding | 4.23 | 1.42 | -0.82 | 0.99 |
| HDC06790 |  |  | 6790 | Unknown | 4.20 | 1.70 | -0.92 | 0.95 |
| RhoGAP18B | 30986 | 7481 |  | Signaling | 4.20 | 1.42 | 3.12 | 1.29 |
| CG4729 | 36623 | 4729 |  | Metabolism | 4.19 | 1.34 | 4.63 | 1.35 |
| CG12263 | 34346 | 12263 |  | Metabolism | 4.17 | 1.60 | 1.96 | 1.29 |
| CG17494 | 40011 | 12002 |  | Unknown | 4.16 | 1.47 | 2.38 | 1.27 |
| Pez | 31799 | 9493 |  | Signaling | 4.12 | 1.57 | 1.54 | 1.11 |
| CG15674 | 34642 | 15674 |  | Unknown | 4.07 | 1.61 | 0.75 | 1.18 |
| CG4320 | 29840 | 4320 |  | Signaling | 4.07 | 1.30 | 3.48 | 1.21 |
| MED19 | 36761 | 5546 |  | DNA binding | 4.06 | 1.55 | 2.40 | 1.22 |
| CG12663 | 29961 | 12663 |  | Unknown | 4.06 | 1.59 | -1.23 | 1.01 |
| CG18172 | 35261 | 2086 |  | Signaling | 4.03 | 1.35 | 2.58 | 1.17 |
| dome | 43903 | 14226 |  | Signaling | 3.99 | 1.27 | 1.64 | 1.16 |
| Ca-alpha1T | 29846 | 15899 |  | Other | 3.96 | 1.28 | -1.47 | 0.79 |
| CG3176 | 29524 | 3176 |  | Unknown | 3.94 | 1.67 | -1.04 | 0.98 |
| igl | 13467 | 18285 |  | Unknown | 3.93 | 1.33 | 1.43 | 1.14 |
| Rac2 | 14011 | 8556 |  | Signaling | 3.93 | 1.27 | 1.48 | 1.02 |
| CG13363 | 25639 | 13363 |  | Other | 3.92 | 1.14 | 4.00 | 1.22 |
| mthl7 | 35847 | 7476 |  | Signaling | 3.89 | 1.26 | 0.28 | 0.94 |
| l(3)mbt | 2441 | 5954 |  | DNA binding | 3.88 | 1.68 | 0.03 | 1.28 |
| CG8446 | 34089 | 8446 |  | Other | 3.86 | 1.53 | 0.84 | 0.97 |
| RpII18 | 3275 | 1163 |  | DNA binding | 3.86 | 1.44 | 1.16 | 1.16 |
| RfC40 | 15287 | 14999 |  | DNA binding | 3.83 | 1.34 | 0.67 | 1.10 |
| HDC05815 |  |  | 5815 | Unknown | 3.81 | 1.52 | -1.31 | 0.96 |
| HDC11835 |  |  | 11835 | Unknown | 3.81 | 1.26 | 0.06 | 0.94 |
| RpP2 | 2593 | 4087 |  | Translation | 3.80 | 1.62 | 1.38 | 1.05 |
| CG32000 | 52000 | 32000 |  | Metabolism | 3.76 | 1.23 | 3.14 | 1.29 |
| CG32690 | 52690 | 32690 |  | Unknown | 3.75 | 1.29 | -1.10 | 0.98 |
| CG3891 | 35993 | 3891 |  | DNA binding | 3.70 | 1.52 | 2.55 | 1.35 |
| CG17233 | 36958 | 17233 |  | Unknown | 3.70 | 1.33 | -0.19 | 0.96 |
| CG5360 | 34873 | 5360 |  | Unknown | 3.67 | 1.14 | 1.26 | 1.14 |
| CG31047 | 51047 | 31047 |  | Unknown | 3.66 | 1.49 | 0.84 | 1.02 |
| HDC17826 |  |  | 17826 | Unknown | 3.65 | 1.33 | 1.41 | 0.96 |
| CG6280 | 33866 | 6280 |  | Unknown | 3.65 | 1.30 | 0.27 | 1.06 |
| Trf2 | 26758 | 18009 |  | DNA binding | 3.64 | 1.29 | 2.18 | 1.20 |
| CG10189 | 32793 | 10189 |  | Unknown | 3.63 | 1.43 | -0.85 | 1.00 |
| HDC10185 |  |  | 10185 | Unknown | 3.63 | 1.25 | 1.40 | 1.07 |
| CG4119 | 28474 | 4119 |  | RNA binding | 3.62 | 1.43 | 2.24 | 1.23 |
| CG14782 | 25381 | 14782 |  | Cytoskeleton | 3.61 | 1.44 | -0.03 | 1.02 |
| CG11132 | 34537 | 11132 |  | DNA binding | 3.61 | 1.74 | 1.91 | 1.38 |
| HDC17828 |  |  | 17828 | Unknown | 3.60 | 1.40 | 1.19 | 0.95 |
| Taf6 | 10417 | 32211 |  | DNA binding | 3.59 | 1.53 | 1.61 | 1.22 |
| cnc | 338 | 17894 |  | DNA binding | 3.58 | 1.47 | 2.70 | 1.34 |
| CG11984 | 37655 | 11984 |  | Other | 3.57 | 1.40 | 2.69 | 1.13 |
| Doa | 53553 | 31049 |  | Signaling | 3.57 | 1.53 | 1.65 | 1.12 |
| HDC04702 |  |  | 4702 | Unknown | 3.56 | 1.37 | 0.66 | 1.14 |
| Bx | 242 | 6500 |  | DNA binding | 3.56 | 1.37 | 2.96 | 1.28 |
| HDC13104 |  |  | 13104 | Unknown | 3.51 | 1.28 | 0.22 | 0.99 |
| CG5757 | 34299 | 5757 |  | Metabolism | 3.51 | 1.47 | 0.50 | 1.04 |
| Rpb11 | 32634 | 6840 |  | DNA binding | 3.49 | 1.32 | 1.28 | 1.17 |
| CG7215 | 38571 | 32920 |  | Unknown | 3.49 | 1.28 | 1.00 | 1.05 |
| CG9663 | 31516 | 9663 |  | Other | 3.48 | 1.38 | 0.36 | 0.90 |
| HDC00888 |  |  | 888 | Unknown | 3.48 | 1.42 | 1.43 | 1.19 |
| CG8771 | 33766 | 8771 |  | Unknown | 3.48 | 1.08 | 2.99 | 1.08 |
| lz | 2576 | 1689 |  | DNA binding | 3.47 | 1.24 | 0.54 | 0.94 |
| Dref | 15664 | 5838 |  | DNA binding | 3.47 | 1.22 | 0.72 | 1.02 |
| HDC19487 |  |  | 19487 | Unknown | 3.46 | 1.26 | 1.61 | 0.95 |
| CG31739 | 51739 | 31739 |  | RNA binding | 3.46 | 1.61 | -0.08 | 1.11 |
| CG10225 | 39110 | 10225 |  | Other | 3.45 | 1.43 | 1.83 | 1.21 |
| HDC00403 |  |  | 403 | Unknown | 3.45 | 1.31 | -1.56 | 0.83 |
| HDC00897 |  |  | 897 | Unknown | 3.45 | 1.49 | -0.14 | 1.05 |
| Ras85D | 3205 | 9375 |  | Signaling | 3.44 | 1.42 | 0.72 | 1.03 |
| HDC08321 |  |  | 8321 | Unknown | 3.44 | 1.17 | 0.25 | 0.96 |
| Tom40 | 16041 | 12157 |  | Other | 3.42 | 1.62 | 1.32 | 1.16 |
| Eip74EF | 567 | 32180 |  | DNA binding | 3.42 | 1.32 | 2.51 | 1.19 |
| CG15121 | 34456 | 15121 |  | Unknown | 3.42 | 1.39 | -0.76 | 0.97 |
| B52 | 4587 | 10851 |  | RNA binding | 3.42 | 1.27 | 2.63 | 1.29 |
| CG12341 | 33550 | 12341 |  | Unknown | 3.41 | 1.08 | 0.04 | 0.81 |
| CG14313 | 38579 | 14313 |  | Unknown | 3.41 | 1.50 | 4.46 | 1.72 |
| mys | 4657 | 1560 |  | Cytoskeleton | 3.40 | 1.39 | 1.59 | 0.97 |
| CG6735 | 36472 | 6735 |  | Cytoskeleton | 3.40 | 1.71 | 2.16 | 1.26 |
| MED10 | 36581 | 5057 |  | DNA binding | 3.39 | 1.52 | 0.72 | 1.10 |
| HDC09508 |  |  | 9508 | Unknown | 3.38 | 1.30 | 0.65 | 1.00 |
| maf-S | 34534 | 9954 |  | DNA binding | 3.38 | 1.43 | 2.03 | 1.10 |
| CG12923 | 33461 | 12923 |  | Unknown | 3.36 | 1.26 | 0.14 | 0.94 |
| CG9304 | 34674 | 9304 |  | Unknown | 3.36 | 1.40 | 4.28 | 1.22 |
| HDC08330 |  |  | 8330 | Unknown | 3.35 | 1.20 | -0.16 | 0.92 |
| srp | 3507 | 3992 |  | DNA binding | 3.35 | 1.15 |  | 0.65 |
| CG8517 | 34472 | 8517 |  | Metabolism | 3.33 | 1.60 | 0.62 | 1.12 |
| HDC14726 |  |  | 14726 | Unknown | 3.33 | 1.39 | -0.19 | 0.90 |
| HDC09514 |  |  | 9514 | Unknown | 3.33 | 1.54 | -0.74 | 0.98 |
| CG15119 | 34430 | 15119 |  | Unknown | 3.32 | 1.46 | -1.27 | 0.84 |
| zf30C | 22720 | 3998 |  | DNA binding | 3.31 | 1.44 | -0.22 | 0.82 |
| Hrb98DE | 1215 | 9983 |  | RNA binding | 3.31 | 1.60 | 0.98 | 1.08 |
| CG8057 | 33383 | 8057 |  | Signaling | 3.31 | 1.53 | 1.84 | 1.10 |
| HDC02034 |  |  | 2034 | Unknown | 3.30 | 1.30 | 1.24 | 1.12 |
| CG15324 | 29966 | 15324 |  | Other | 3.28 | 1.50 | -1.28 | 0.97 |
| Act57B | 44 | 10067 |  | Cytoskeleton | 3.28 | 1.20 | 4.28 | 1.13 |
| HDC14831 |  |  | 14831 | Unknown | 3.27 | 1.17 | 1.35 | 1.05 |
| Taf12 | 11290 | 17358 |  | DNA binding | 3.27 | 1.63 | -0.24 | 1.07 |
| CG1910 | 22349 | 1910 |  | Unknown | 3.26 | 1.30 | 1.62 | 1.09 |
| thr | 3701 | 5785 |  | Unknown | 3.25 | 1.42 | -0.89 | 0.97 |
| CG9394 | 34588 | 9394 |  | Unknown | 3.24 | 1.42 | 1.66 | 1.11 |
| Tektin-C | 35638 | 10541 |  | Cytoskeletal | 3.24 | 1.78 | 0.53 | 1.12 |
| HDC03047 |  |  | 3047 | Unknown | 3.24 | 1.46 | -0.61 | 1.01 |
| CG7808 | 39713 | 7808 |  | Translation | 3.23 | 1.45 | 0.33 | 1.10 |
| CG12050 | 32915 | 12050 |  | Unknown | 3.22 | 1.16 | 3.17 | 1.00 |
| Rpt3 | 28686 | 16916 |  | Proteolysis | 3.20 | 1.41 | -1.13 | 0.93 |
| sec23 | 37357 | 1250 |  | Signaling | 3.19 | 1.51 | -0.29 | 1.10 |
| HDC10130 |  |  | 10130 | Unknown | 3.19 | 1.30 | 1.92 | 1.21 |
| scaf6 | 52168 | 32168 |  | RNA binding | 3.19 | 1.13 | 3.41 | 1.09 |
| CG32817 | 52817 | 32817 |  | Unknown | 3.18 | 1.28 | -0.57 | 1.04 |
| if | 1250 | 9623 |  | Signaling | 3.18 | 1.42 | 0.59 | 1.08 |
| CG3476 | 31881 | 3476 |  | Metabolism | 3.18 | 1.92 | 0.90 | 1.52 |
| CG4631 | 32590 | 4631 |  | Unknown | 3.17 | 1.22 | -0.14 | 1.07 |
| CG14160 | 36066 | 14160 |  | Unknown | 3.16 | 1.15 | 1.55 | 1.13 |
| CG16865 | 28919 | 16865 |  | Unknown | 3.15 | 1.17 | 1.22 | 1.15 |
| CG11245 | 30388 | 11245 |  | Unknown | 3.15 | 1.14 | 2.01 | 0.93 |
| spz | 3495 | 6134 |  | Signaling | 3.15 | 1.20 | 1.12 | 1.14 |
| CG13609 | 39170 | 13609 |  | Unknown | 3.14 | 1.25 | -0.56 | 0.83 |
| CG30217 | 50217 | 30217 |  | Unknown | 3.13 | 1.19 | 1.20 | 1.01 |
| CG13802 | 35330 | 13802 |  | Unknown | 3.13 | 1.19 | 1.61 | 1.06 |
| CG7177 | 37098 | 7177 |  | Signaling | 3.12 | 1.45 | 2.33 | 1.35 |
| HDC09397 |  |  | 9397 | Unknown | 3.12 | 1.36 | 1.38 | 1.03 |
| HDC11912 |  |  | 11912 | Unknown | 3.12 | 1.45 | -1.49 | 0.95 |
| HDC00497 |  |  | 497 | Unknown | 3.12 | 1.33 | 1.09 | 1.15 |
| CG11321 | 31857 | 11321 |  | Unknown | 3.12 | 1.32 | 1.48 | 1.10 |
| CG1244 | 35357 | 1244 |  | Unknown | 3.10 | 1.48 | 2.40 | 1.27 |
| CG13675 | 35845 | 13675 |  | Unknown | 3.09 | 1.33 | 0.55 | 1.02 |
| fidipidine | 25519 | 7773 |  | Other | 3.09 | 1.64 | -0.38 | 1.03 |
| CG3731 | 38271 | 3731 |  | Proteolysis | 3.08 | 1.06 | 1.00 | 1.01 |
| CG5746 | 39186 | 5746 |  | Unknown | 3.08 | 1.23 | 0.06 | 1.12 |
| Pvf2 | 31888 | 13780 |  | Signaling | 3.06 | 1.50 | -0.96 | 1.03 |
| kay | 1297 | 15509 |  | DNA binding | 3.06 | 1.27 | 3.56 | 1.48 |
| CG9895 | 34810 | 9895 |  | DNA binding | 3.06 | 1.38 | -0.84 | 0.92 |
| CG18545 | 37812 | 18545 |  | Unknown | 3.05 | 1.13 | 2.74 | 1.13 |
| CG14722 | 37943 | 14722 |  | Unknown | 3.04 | 1.10 | 1.71 | 1.09 |
| HDC17852 |  |  | 17852 | Unknown | 3.04 | 1.33 | 1.35 | 0.96 |
| Appl | 108 | 7727 |  | Unknown | 3.01 | 1.50 | 1.72 | 1.27 |
| CG11227 | 31139 | 11227 |  | Unknown | 3.01 | 1.26 | 0.85 | 1.09 |
| CG12681 | 29730 | 12681 |  | Unknown | 3.01 | 1.47 | -0.62 | 1.03 |
| rtet | 28468 | 5760 |  | Other | 3.00 | 1.42 | 0.57 | 1.03 |
| CG9523 | 31812 | 9523 |  | Unknown | 3.00 | 1.30 | 2.69 | 1.22 |
| CG2083 | 35376 | 2083 |  | Unknown | 2.99 | 1.32 | 0.22 | 0.84 |
| RpL1 | 3279 | 5502 |  | Translation | 2.99 | 1.31 | 1.98 | 0.99 |
| CG8260 | 30684 | 8260 |  | Unknown | 2.98 | 1.17 | 0.12 | 0.94 |
| CG2652 | 25838 | 2652 |  | Unknown | 2.96 | 1.40 | -1.90 | 0.91 |
| mRpL-CI-B8 | 34893 | 5479 |  | Translation | 2.96 | 1.25 | 3.05 | 1.18 |
| HDC17815 |  |  | 17815 | Unknown | 2.95 | 1.34 | 0.33 | 0.97 |
| HDC14028 |  |  | 14028 | Unknown | 2.95 | 1.15 | 1.23 | 1.00 |
| CG13845 | 38971 | 13845 |  | Unknown | 2.95 | 1.08 | 0.22 | 1.04 |
| Hem | 11771 | 5837 |  | Signaling | 2.95 | 1.45 | -0.02 | 0.94 |
| CG4896 | 31319 | 4896 |  | RNA binding | 2.94 | 1.62 | 2.69 | 1.27 |
| CG31394 | 51394 | 31394 |  | Unknown | 2.94 | 1.32 | 0.58 | 1.16 |
| HDC19488 |  |  | 19488 | Unknown | 2.94 | 1.25 | 1.49 | 0.95 |
| CG33296 | 53296 | 33296 |  | Unknown | 2.94 | 1.28 | 1.47 | 1.21 |
| CG32104 | 52104 | 32104 |  | Unknown | 2.94 | 1.36 | -0.21 | 0.96 |
| CG1553 | 33224 | 1553 |  | Unknown | 2.94 | 1.31 | -0.74 | 1.05 |
| CG15488 | 32440 | 15488 |  | Unknown | 2.94 | 1.25 | 1.89 | 1.05 |
| HDC14817 |  |  | 14817 | Unknown | 2.93 | 1.19 | 2.81 | 1.15 |
| Act42A | 43 | 12051 |  | Cytoskeleton | 2.92 | 1.08 | 4.13 | 1.00 |
| CG30034 | 50034 | 30034 |  | Unknown | 2.92 | 1.36 | 0.21 | 1.00 |
| SCAR | 41781 | 4636 |  | Cytoskeleton | 2.90 | 1.19 | 1.50 | 1.09 |
| PQBP-1 | 51369 | 31369 |  | Unknown | 2.89 | 1.13 | 0.35 | 0.94 |
| CG18166 | 29526 | 18166 |  | Unknown | 2.88 | 1.26 | -0.58 | 1.02 |
| HDC09406 |  |  | 9406 | Unknown | 2.88 | 1.30 | 0.22 | 0.92 |
| CG9122 | 35187 | 9122 |  | Metabolism | 2.87 | 1.52 | 0.64 | 1.02 |
| dap | 10316 | 1772 |  | Signaling | 2.87 | 1.45 | 0.55 | 1.04 |
| CG3777 | 24989 | 3777 |  | Unknown | 2.87 | 1.24 | 0.63 | 1.09 |
| HDC07441 |  |  | 7441 | Unknown | 2.87 | 1.36 | -1.35 | 0.98 |
| Arp53D | 11743 | 5409 |  | Cytoskeleton | 2.87 | 1.13 | 1.57 | 0.94 |
| CG4612 | 35016 | 4612 |  | RNA binding | 2.87 | 1.09 | 1.32 | 1.01 |
| Arc92 | 38760 | 12254 |  | DNA binding | 2.85 | 1.02 |  | 0.55 |
| Obp58d | 34770 | 13519 |  | Other | 2.85 | 1.09 | 0.95 | 1.04 |
| HDC15864 |  |  | 15864 | Unknown | 2.85 | 1.12 | 0.90 | 0.93 |
| HDC12184 |  |  | 12184 | Unknown | 2.84 | 1.29 | 1.07 | 0.99 |
| Dl | 463 | 3619 |  | Signaling | 2.84 | 1.06 | 3.40 | 1.22 |
| HDC13103 |  |  | 13103 | Unknown | 2.83 | 1.30 | 0.29 | 0.98 |
| HDC08318 |  |  | 8318 | Unknown | 2.83 | 1.19 | -0.35 | 0.91 |
| CG12255 | 36618 | 12255 |  | Unknown | 2.83 | 1.08 | 1.05 | 0.99 |
| l(2)k01209 | 22029 | 4798 |  | Metabolism | 2.82 | 1.06 | 2.44 | 1.10 |
| HDC07043 |  |  | 7043 | Unknown | 2.82 | 1.13 | 0.54 | 1.00 |
| CG13041 | 36605 | 13041 |  | Unknown | 2.81 | 1.25 | -1.28 | 0.98 |
| dsf | 15381 | 9019 |  | DNA binding | 2.81 | 1.40 | -0.69 | 0.92 |
| CG16817 | 37728 | 16817 |  | Unknown | 2.80 | 1.14 | 1.93 | 1.24 |
| CG6369 | 39260 | 6369 |  | RNA binding | 2.80 | 1.46 | 1.54 | 1.23 |
| RluA-2 | 32256 | 6187 |  | Metabolism | 2.79 | 1.34 | 1.82 | 1.13 |
| ear | 26441 | 4913 |  | DNA binding | 2.78 | 1.25 | 0.99 | 1.07 |
| HDC07585 |  |  | 7585 | Unknown | 2.78 | 1.19 | 0.57 | 1.06 |
| so | 3460 | 11121 |  | DNA binding | 2.78 | 1.34 | 1.15 | 1.12 |
| CG8509 | 30696 | 8509 |  | Signaling | 2.78 | 1.60 | 1.76 | 1.18 |
| HDC06322 |  |  | 6322 | Unknown | 2.78 | 1.23 | 2.10 | 1.14 |
| CG15006 | 35510 | 15006 |  | Unknown | 2.77 | 1.13 | 0.84 | 1.03 |
| HDC17406 |  |  | 17406 | Unknown | 2.77 | 1.10 | 0.48 | 1.01 |
| TfIIE&bgr; | 15829 | 1276 |  | DNA binding | 2.76 | 1.27 | 2.90 | 1.35 |
| mRpL21 | 36853 | 9730 |  | Translation | 2.75 | 1.27 | 1.23 | 1.00 |
| HDC14730 |  |  | 14730 | Unknown | 2.74 | 1.38 | -1.37 | 0.92 |
| CkII&agr;-i1 | 15025 | 6215 |  | Unknown | 2.74 | 1.55 | 0.67 | 1.15 |
| CG5800 | 30855 | 5800 |  | RNA binding | 2.73 | 1.20 | 0.06 | 1.04 |
| CG9862 | 34646 | 9862 |  | RNA binding | 2.72 | 1.51 | -0.04 | 1.05 |
| eIF-2&bgr; | 4926 | 4153 |  | Translation | 2.72 | 1.56 | 1.18 | 1.20 |
| CG11843 | 39630 | 11843 |  | Proteolysis | 2.72 | 1.39 | 0.78 | 1.04 |
| AlstR | 28961 | 2872 |  | signaling | 2.71 | 1.54 | -3.14 | 0.78 |
| pAbp | 3031 | 5119 |  | RNA binding | 2.71 | 1.21 | 4.48 | 1.39 |
| Pomp | 32884 | 9324 |  | Proteolysis | 2.71 | 1.58 | 5.65 | 1.95 |
| CG15185 | 37449 | 15185 |  | Unknown | 2.71 | 1.13 | 1.51 | 1.04 |
| Mov34 | 2787 | 3416 |  | Proteolysis | 2.71 | 1.23 | 1.92 | 1.12 |
| CG18446 | 33458 | 18446 |  | Unknown | 2.71 | 1.23 | -0.11 | 0.97 |
| HDC19486 |  |  | 19486 | Unknown | 2.70 | 1.08 | 1.30 | 0.94 |
| CG10320 | 34645 | 10320 |  | Other | 2.70 | 1.14 | 0.33 | 1.07 |
| CG12175 | 30502 | 12175 |  | DNA binding | 2.70 | 1.23 | 1.07 | 0.93 |
| PRL-1 | 24734 | 4993 |  | Signaling | 2.70 | 1.18 | 0.47 | 1.01 |
| CG11260 | 39912 | 11260 |  | Unknown | 2.70 | 1.40 | -0.55 | 1.00 |
| CG8885 | 31656 | 8885 |  | Metabolism | 2.70 | 1.50 | -0.09 | 1.07 |
| HDC17312 |  |  | 17312 | Unknown | 2.70 | 1.14 | 1.30 | 1.06 |
| CG7366 | 35855 | 7366 |  | Unknown | 2.69 | 1.26 | -0.01 | 0.91 |
| CG33346 | 53346 | 33346 |  | Unknown | 2.69 | 1.38 | 1.00 | 1.05 |
| Hexo2 | 41629 | 1787 |  | Metabolism | 2.69 | 1.34 | 2.39 | 1.19 |
| CG3838 | 32130 | 3838 |  | Unknown | 2.68 | 1.37 | -3.08 | 0.75 |
| HDC10201 |  |  | 10201 | Unknown | 2.68 | 1.19 | 0.56 | 1.00 |
| HDC13864 |  |  | 13864 | Unknown | 2.68 | 1.19 | -0.05 | 0.99 |
| HDC15843 |  |  | 15843 | Unknown | 2.68 | 1.09 | 1.03 | 0.94 |
| scaf6 | 53522 | 6615 |  | RNA binding | 2.68 | 1.03 | 1.94 | 1.03 |
| CG12744 | 33459 | 12744 |  | Unknown | 2.67 | 1.20 | 0.86 | 0.96 |
| HDC19511 |  |  | 19511 | Unknown | 2.67 | 1.36 | 0.01 | 1.00 |
| HDC14841 |  |  | 14841 | Unknown | 2.67 | 1.13 | 0.39 | 0.98 |
| CG15326 | 29965 | 15326 |  | Unknown | 2.66 | 1.49 | -1.17 | 0.99 |
| mud | 2873 | 12047 |  | Unknown | 2.65 | 1.16 | 0.96 | 1.25 |
| Lasp | 63485 | 3849 |  | Cytoskeleton | 2.65 | 1.21 | 0.40 | 1.11 |
| bw | 241 | 17632 |  | Other | 2.64 | 1.36 | -0.32 | 1.00 |
| HDC08316 |  |  | 8316 | Unknown | 2.63 | 1.19 | -0.69 | 0.91 |
| CG12582 | 37215 | 12582 |  | Metabolism | 2.62 | 1.25 | 0.59 | 1.02 |
| CG13358 | 26874 | 13358 |  | Unknown | 2.62 | 1.21 | 0.01 | 0.98 |
| CG30196 | 50196 | 30196 |  | Unknown | 2.61 | 1.09 | 1.31 | 1.07 |
| CG10990 | 30520 | 10990 |  | Unknown | 2.60 | 1.25 | 0.60 | 1.21 |
| CG32058 | 52058 | 32058 |  | Unknown | 2.60 | 1.09 | 1.49 | 1.13 |
| HDC07692 |  |  | 7692 | Unknown | 2.59 | 1.30 | -0.81 | 1.03 |
| Mes2 | 37207 | 11100 |  | Unknown | 2.59 | 1.35 | 1.68 | 1.10 |
| CG13047 | 36594 | 13047 |  | Unknown | 2.59 | 1.08 | 2.01 | 1.14 |
| HDC13099 |  |  | 13099 | Unknown | 2.59 | 1.33 | 0.06 | 0.91 |
| CG15909 | 33090 | 15909 |  | Unknown | 2.59 | 1.36 | -0.96 | 0.94 |
| Arp66B | 11744 | 7558 |  | Cytoskeleton | 2.58 | 1.13 | 3.28 | 1.12 |
| CG12091 | 35228 | 12091 |  | Signaling | 2.58 | 1.37 | 0.03 | 0.99 |
| rut | 3301 | 9533 |  | Signaling | 2.58 | 1.35 | -0.61 | 1.02 |
| CG6674 | 36063 | 6674 |  | Unknown | 2.58 | 1.15 | 1.18 | 1.14 |
| tun | 50084 | 30084 |  | Unknown | 2.58 | 1.50 | 1.48 | 0.93 |
| peb | 3053 | 12212 |  | DNA binding | 2.57 | 1.41 | 0.90 | 1.17 |
| CG31712 | 51712 | 31712 |  | Unknown | 2.56 | 1.18 | 0.13 | 0.97 |
| CG32054 | 52054 | 32054 |  | Other | 2.56 | 1.17 | 0.57 | 1.09 |
| HDC14728 |  |  | 14728 | Unknown | 2.56 | 1.29 | 0.03 | 0.96 |
| CG10630 | 35608 | 10630 |  | Unknown | 2.56 | 1.30 | 0.80 | 1.13 |
| CG2556 | 30396 | 2556 |  | Unknown | 2.55 | 1.18 | 0.63 | 1.01 |
| Pros&bgr;2 | 23174 | 3329 |  | Proteolysis | 2.55 | 1.77 | 1.08 | 1.52 |
| Os-E | 10403 | 11422 |  | Signaling | 2.55 | 1.14 | 0.73 | 0.97 |
| Sra-1 | 38320 | 4931 |  | Signaling | 2.54 | 1.09 | 1.90 | 1.07 |
| HDC19504 |  |  | 19504 | Unknown | 2.54 | 1.29 | 0.58 | 0.96 |
| Cap-H2 | 37831 | 14685 |  | Other | 2.54 | 1.16 | 1.34 | 1.00 |
| CG9293 | 32516 | 9293 |  | DNA binding | 2.52 | 1.15 | 0.51 | 0.82 |
| CG8223 | 37624 | 8223 |  | Unknown | 2.52 | 1.04 | 1.62 | 1.02 |
| Tbp-1 | 28684 | 10370 |  | proteolysis | 2.51 | 1.51 | 0.17 | 1.05 |
| Mdr50 | 10241 | 8523 |  | Other | 2.51 | 1.58 | 0.15 | 1.23 |
| CG9973 | 35378 | 9973 |  | Unknown | 2.50 | 1.13 | 1.30 | 1.10 |
| Ets97D | 4510 | 6338 |  | DNA binding | 2.50 | 1.43 | 0.15 | 1.12 |
| CG8042 | 27554 | 8042 |  | Unknown | 2.50 | 1.22 | -0.96 | 0.96 |
| CG8066 | 38243 | 8066 |  | Unknown | 2.50 | 1.23 | 1.30 | 1.15 |
| CG15894 | 29864 | 15894 |  | Unknown | 2.50 | 1.17 | 1.07 | 1.03 |
| rl | 3256 | 12559 |  | Signaling | 2.50 | 1.34 | 0.13 | 1.07 |
| Pros25 | 10405 | 5266 |  | Proteolysis | 2.49 | 1.53 | 1.58 | 1.31 |
| HDC17817 |  |  | 17817 | Unknown | 2.49 | 1.32 | 0.19 | 0.94 |
| CG32755 | 52755 | 32755 |  | Unknown | 2.49 | 1.14 | -0.35 | 0.88 |
| UbcD6 | 4436 | 2013 |  | Other | 2.48 | 1.24 | 1.08 | 1.10 |
| CG6685 | 36062 | 6685 |  | Unknown | 2.48 | 1.17 | 0.85 | 1.12 |
| CG31607 | 51607 | 31607 |  | Unknown | 2.48 | 1.01 | 0.88 | 1.00 |
| msi | 11666 | 5099 |  | RNA binding | 2.48 | 1.48 | 1.90 | 1.36 |
| CG33203 | 53203 | 33203 |  | Unknown | 2.47 | 1.36 | 1.69 | 1.09 |
| zormin | 52311 | 33484 |  | Unknown | 2.47 | 1.26 | 2.36 | 1.18 |
| HDC12190 |  |  | 12190 | Unknown | 2.47 | 1.26 | 0.35 | 0.97 |
| CG1973 | 39692 | 1973 |  | Signaling | 2.46 | 1.00 | 1.46 | 0.92 |
| NHP2 | 29148 | 5258 |  | RNA binding | 2.46 | 1.14 | 1.97 | 1.09 |
| HDC06940 |  |  | 6940 | Unknown | 2.46 | 1.16 | 0.37 | 0.99 |
| can | 11569 | 6577 |  | DNA binding | 2.46 | 1.06 | 0.63 | 1.02 |
| CG33187 | 53187 | 33187 |  | Unknown | 2.46 | 1.06 | 1.22 | 1.18 |
| CG14194 | 30996 | 14194 |  | Unknown | 2.46 | 1.10 | 0.71 | 0.94 |
| CG32946 | 52946 | 32946 |  | Unknown | 2.45 | 1.13 | 0.49 | 0.97 |
| beat-Vb | 38092 | 31298 |  | Unknown | 2.45 | 1.16 | 0.84 | 0.99 |
| HDC17821 |  |  | 17821 | Unknown | 2.45 | 1.35 | -0.49 | 0.97 |
| Taf10b | 26324 | 3069 |  | DNA binding | 2.45 | 1.10 | 1.52 | 1.07 |
| HDC06591 |  |  | 6591 | Unknown | 2.44 | 1.20 | 0.52 | 1.03 |
| CG12920 | 33481 | 12920 |  | Unknown | 2.44 | 1.34 | -0.85 | 0.96 |
| HDC06795 |  |  | 6795 | Unknown | 2.44 | 1.44 | -0.91 | 0.98 |
| smg | 16070 | 5263 |  | RNA binding | 2.43 | 1.16 | 2.15 | 1.06 |
| CG12119 | 30102 | 12119 |  | Unknown | 2.43 | 1.27 | 0.46 | 1.03 |
| fne | 40222 | 4396 |  | RNA binding | 2.43 | 1.28 | 0.71 | 1.13 |
| HDC00033 |  |  | 33 | Unknown | 2.42 | 1.31 | -2.48 | 0.76 |
| slpr | 30018 | 2272 |  | Signaling | 2.42 | 1.40 | -0.09 | 0.97 |
| HDC14799 |  |  | 14799 | Unknown | 2.42 | 1.33 | -0.51 | 0.92 |
| HDC14842 |  |  | 14842 | Unknown | 2.41 | 1.09 | 0.07 | 0.94 |
| CG10752 | 36325 | 10752 |  | Unknown | 2.41 | 1.07 | 2.20 | 1.06 |
| CG7763 | 40503 | 7763 |  | Unknown | 2.41 | 1.31 | -1.23 | 0.94 |
| Taf8 | 22724 | 7128 |  | DNA binding | 2.40 | 1.28 | 1.16 | 1.11 |
| CG8501 | 33724 | 8501 |  | Unknown | 2.40 | 1.13 | 0.51 | 1.03 |
| Rpn6 | 28689 | 10149 |  | Proteolysis | 2.40 | 1.10 | 5.02 | 1.44 |
| Hsc70-1 | 1216 | 8937 |  | Other | 2.40 | 1.29 | 0.97 | 0.91 |
| ss | 3513 | 6993 |  | DNA binding | 2.40 | 1.28 | 3.91 | 1.25 |
| HDC16223 |  |  | 16223 | Unknown | 2.39 | 1.38 | 0.06 | 0.97 |
| HDC11436 |  |  | 11436 | Unknown | 2.39 | 1.15 | 0.58 | 1.00 |
| HDC13111 |  |  | 13111 | Unknown | 2.39 | 1.26 | -0.31 | 0.96 |
| CG13338 | 33867 | 13338 |  | Unknown | 2.39 | 1.25 | 0.12 | 1.09 |
| HDC06912 |  |  | 6912 | Unknown | 2.39 | 1.30 | -1.56 | 0.93 |
| CG4367 | 38783 | 4367 |  | Unknown | 2.39 | 1.11 | -0.17 | 0.98 |
| TER94 | 24923 | 2331 |  | Other | 2.38 | 1.21 | 1.63 | 1.10 |
| CG11575 | 39879 | 11575 |  | Unknown | 2.38 | 1.20 | 0.87 | 1.03 |
| mus201 | 2887 | 32956 |  | DNA binding | 2.38 | 1.02 | 1.23 | 1.08 |
| Pp2A-29B | 5776 | 33297 |  | Signaling | 2.38 | 1.29 | -0.22 | 0.96 |
| CG18266 | 31724 | 18266 |  | Unknown | 2.38 | 1.12 | 0.71 | 1.04 |
| ksr | 15402 | 2899 |  | Signaling | 2.38 | 1.11 | 2.01 | 1.05 |
| CG12069 | 39796 | 12069 |  | Signaling | 2.37 | 1.53 | 0.12 | 1.31 |
| CG11486 | 35397 | 11486 |  | Unknown | 2.37 | 1.48 | 0.35 | 1.17 |
| CG32368 | 52368 | 32368 |  | Unknown | 2.36 | 1.20 | -0.07 | 1.00 |
| CG9603 | 40529 | 9603 |  | Metabolism | 2.36 | 1.10 | -0.17 | 0.94 |
| CG3528 | 31430 | 3528 |  | Unknown | 2.36 | 1.22 | 1.46 | 1.23 |
| snRNP70K | 16978 | 8749 |  | RNA binding | 2.36 | 1.44 | -0.54 | 1.09 |
| Cip4 | 35533 | 15015 |  | Signaling | 2.36 | 1.23 | 1.65 | 1.34 |
| HDC19521 |  |  | 19521 | Unknown | 2.36 | 1.42 | 0.30 | 1.09 |
| CG4615 | 29935 | 4615 |  | Unknown | 2.36 | 1.11 | 0.57 | 1.12 |
| CG12773 | 24365 | 12773 |  | Other | 2.35 | 1.22 | 1.33 | 1.19 |
| CG13530 | 34777 | 13530 |  | Unknown | 2.35 | 1.11 | 1.76 | 1.14 |
| CG13932 | 35259 | 13932 |  | Unknown | 2.35 | 1.06 | 0.24 | 0.92 |
| CG14801 | 24988 | 14801 |  | Metabolism | 2.35 | 0.86 | 0.41 | 0.86 |
| HDC20230 |  |  | 20230 | Unknown | 2.35 | 1.35 | 0.01 | 1.03 |
| CG17540 | 40024 | 7371 |  | RNA binding | 2.34 | 1.25 | 1.82 | 1.24 |
| v(2)k05816 | 42627 | 3524 |  | Metabolism | 2.34 | 1.38 | 0.02 | 0.96 |
| CG17265 | 31488 | 17265 |  | Unknown | 2.34 | 1.14 | -2.31 | 0.65 |
| CG30264 | 50264 | 30264 |  | Unknown | 2.34 | 1.08 | 1.76 | 1.07 |
| P5cr | 15781 | 6009 |  | Metabolism | 2.33 | 1.31 | 1.02 | 1.08 |
| CG6006 | 63649 | 6006 |  | Other | 2.33 | 1.11 | 2.79 | 1.32 |
| Chd1 | 16132 | 3733 |  | DNA binding | 2.33 | 1.17 | 0.23 | 1.07 |
| Trap19 | 40020 | 11023 |  | DNA binding | 2.33 | 1.13 | 2.56 | 1.29 |
| CG12237 | 31048 | 12237 |  | Unknown | 2.33 | 1.52 | -0.70 | 0.99 |
| Gr59f | 41234 | 33150 |  | Signaling | 2.32 | 0.99 | 1.74 | 1.07 |
| mthl6 | 35789 | 16992 |  | Signaling | 2.32 | 1.23 | -0.01 | 1.06 |
| CG9650 | 29939 | 9650 |  | DNA binding | 2.32 | 1.16 | 1.75 | 0.90 |
| CG9346 | 34572 | 9346 |  | RNA binding | 2.32 | 1.45 | 0.08 | 1.16 |
| CG7678 | 38613 | 7678 |  | Other | 2.32 | 1.28 | 0.04 | 1.13 |
| Dot | 15663 | 2788 |  | Metabolism | 2.32 | 1.28 | 1.25 | 1.04 |
| CG6023 | 30912 | 6023 |  | Unknown | 2.32 | 1.06 | 1.22 | 1.04 |
| CG1746 | 39830 | 1746 |  | Metabolism | 2.31 | 1.14 | 0.77 | 0.97 |
| HDC07586 |  |  | 7586 | Unknown | 2.31 | 1.14 | 1.08 | 1.04 |
| CG31044 | 51044 | 31044 |  | Unknown | 2.31 | 1.30 | 0.67 | 0.99 |
| CG8710 | 33265 | 8710 |  | Unknown | 2.31 | 1.11 | 0.54 | 0.96 |
| CG14998 | 35500 | 14998 |  | Unknown | 2.31 | 1.15 | 2.27 | 1.17 |
| HDC19522 |  |  | 19522 | Unknown | 2.30 | 1.43 | 0.25 | 1.05 |
| CG13198 | 33640 | 13198 |  | Unknown | 2.30 | 1.33 | -0.23 | 0.98 |
| CG7876 | 31000 | 7876 |  | Other | 2.30 | 1.25 | -0.81 | 1.00 |
| Su(Tpl) | 14037 | 32217 |  | DNA binding | 2.29 | 1.13 | 2.74 | 1.29 |
| CG13564 | 34973 | 13564 |  | Unknown | 2.29 | 1.11 | 0.61 | 1.00 |
| Arc70 | 39923 | 1793 |  | DNA binding | 2.29 | 1.49 | 2.59 | 1.46 |
| HDC16885 |  |  | 16885 | Unknown | 2.29 | 1.04 | 1.50 | 0.97 |
| CG14520 | 39618 | 14520 |  | Unknown | 2.28 | 1.25 | 1.13 | 1.03 |
| CG9350 | 34576 | 9350 |  | Unknown | 2.28 | 1.29 | 0.48 | 1.08 |
| Rpt4 | 28685 | 3455 |  | Proteolysis | 2.28 | 1.58 | 2.28 | 1.62 |
| Adar | 26086 | 12598 |  | RNA binding | 2.28 | 1.30 | -0.12 | 0.96 |
| HDC09478 |  |  | 9478 | Unknown | 2.28 | 1.10 | 1.75 | 1.09 |
| CG8290 | 26573 | 8290 |  | DNA binding | 2.27 | 1.04 | 1.19 | 0.96 |
| CG1906 | 39672 | 1906 |  | Signaling | 2.26 | 1.22 | 0.48 | 1.04 |
| HDC19473 |  |  | 19473 | Unknown | 2.26 | 1.22 | 0.96 | 0.99 |
| HDC07589 |  |  | 7589 | Unknown | 2.26 | 1.09 | 1.29 | 1.00 |
| insv | 31434 | 3227 |  | Unknown | 2.26 | 1.14 | 2.29 | 1.20 |
| CG7349 | 30975 | 7349 |  | Metabolism | 2.26 | 1.12 | -0.40 | 0.93 |
| CG9757 | 3060 | 9757 |  | Unknown | 2.25 | 1.20 | 0.45 | 1.16 |
| Trap37 | 37359 | 1245 |  | DNA binding | 2.25 | 1.33 | 0.76 | 1.12 |
| eIF2B-&egr; | 23512 | 3806 |  | DNA binding | 2.24 | 1.25 | -0.42 | 0.99 |
| HDC14720 |  |  | 14720 | Unknown | 2.24 | 1.28 | -0.49 | 0.91 |
| Mlp84B | 14863 | 1019 |  | Unknown | 2.24 | 1.18 | 1.09 | 1.08 |
| Nap1 | 15268 | 5330 |  | DNA binding | 2.24 | 1.32 | -0.70 | 0.96 |
| HDC02627 |  |  | 2627 | Unknown | 2.24 | 1.18 | -0.37 | 0.93 |
| lig | 20279 | 8715 |  | Unknown | 2.23 | 0.99 | 1.34 | 1.02 |
| Egfr | 3731 | 10079 |  | Signaling | 2.23 | 1.15 | 1.67 | 1.32 |
| wibg | 34918 | 30176 |  | Unknown | 2.23 | 1.05 | 0.44 | 0.98 |
| mRNA-capping-enzyme | 30556 | 1810 |  | RNA binding | 2.23 | 1.22 | 4.60 | 1.51 |
| CG14683 | 37822 | 14683 |  | Unknown | 2.23 | 1.14 | 1.39 | 1.05 |
| Gp150 | 13272 | 5820 |  | Signaling | 2.23 | 1.23 | 0.23 | 1.01 |
| CG12842 | 33131 | 12842 |  | Unknown | 2.23 | 1.12 | 0.79 | 1.01 |
| CG18397 | 32723 | 18397 |  | Unknown | 2.22 | 1.14 | 0.19 | 0.95 |
| dnk | 22338 | 5452 |  | Metabolism | 2.22 | 1.13 | 2.34 | 1.14 |
| CG1458 | 62442 | 1458 |  | Unknown | 2.22 | 1.32 | 0.85 | 1.01 |
| HDC12145 |  |  | 12145 | Unknown | 2.22 | 1.16 | 1.33 | 1.05 |
| ik2 | 28633 | 2615 |  | Signaling | 2.22 | 1.18 | 2.99 | 1.20 |
| CG15263 | 28853 | 15263 |  | Unknown | 2.22 | 1.08 | 1.74 | 1.07 |
| CG14972 | 35450 | 14972 |  | Unknown | 2.22 | 1.21 | 0.66 | 1.08 |
| HDC13135 |  |  | 13135 | Unknown | 2.21 | 1.30 | -0.29 | 0.94 |
| CG8877 | 33688 | 8877 |  | RNA binding | 2.21 | 1.29 | 0.15 | 0.96 |
| CG7181 | 37097 | 7181 |  | Metabolism | 2.21 | 1.12 | 1.31 | 1.05 |
| CG10419 | 36850 | 10419 |  | Unknown | 2.21 | 1.10 | 1.61 | 1.08 |
| CG14853 | 38246 | 14853 |  | Unknown | 2.20 | 1.18 | 0.74 | 0.96 |
| CG13283 | 32613 | 13283 |  | Unknown | 2.19 | 1.14 | 1.27 | 1.14 |
| CG12584 | 37257 | 12584 |  | Unknown | 2.19 | 1.30 | 0.44 | 1.11 |
| CG32673 | 52673 | 32673 |  | Signaling | 2.19 | 1.24 | -1.04 | 1.00 |
| CG32148 | 47338 | 32148 |  | Unknown | 2.19 | 1.09 | 1.03 | 1.08 |
| Os9 | 14000 | 10658 |  | Unknown | 2.19 | 1.14 | 0.81 | 1.03 |
| CG3934 | 37783 | 3934 |  | Unknown | 2.19 | 1.16 | -0.21 | 1.03 |
| CG13288 | 35648 | 13288 |  | Unknown | 2.18 | 1.11 | 0.77 | 0.98 |
| CG2082 | 27608 | 2082 |  | Unknown | 2.18 | 1.31 | 0.13 | 0.88 |
| CG14909 | 38458 | 14909 |  | Metabolism | 2.18 | 1.23 | -0.73 | 0.99 |
| CG32053 | 52053 | 32053 |  | Unknown | 2.18 | 1.09 | 0.65 | 1.05 |
| CG5375 | 32221 | 5375 |  | Unknown | 2.18 | 1.16 | 1.66 | 1.02 |
| CG5454 | 38667 | 5454 |  | RNA binding | 2.18 | 1.12 | 1.22 | 1.14 |
| Scg&agr; | 32013 | 7851 |  | Cytoskeleton | 2.18 | 1.77 | 5.58 | 2.34 |
| Hsc70-4 | 1219 | 4264 |  | Other | 2.18 | 1.29 | 0.85 | 0.95 |
| HDC17108 |  |  | 17108 | Unknown | 2.17 | 1.01 | 1.28 | 1.01 |
| CG15403 | 31504 | 15403 |  | Unknown | 2.17 | 1.06 | 1.09 | 1.02 |
| csul | 15925 | 3730 |  | Signaling | 2.17 | 0.99 | 1.56 | 0.96 |
| CG11971 | 22347 | 11971 |  | Unknown | 2.17 | 1.06 | 1.81 | 1.13 |
| CG3708 | 40345 | 3708 |  | DNA binding | 2.17 | 1.14 | 0.26 | 0.98 |
| CG18748 | 42105 | 18748 |  | Unknown | 2.17 | 1.32 | 0.69 | 1.10 |
| e(y)1 | 617 | 6474 |  | DNA binding | 2.17 | 1.16 | 3.33 | 1.14 |
| Os-C | 10401 | 3250 |  | Unknown | 2.17 | 1.04 | 0.77 | 0.95 |
| CG3713 | 40343 | 3713 |  | Unknown | 2.16 | 1.06 | -0.01 | 0.96 |
| Gr59e | 41233 | 33151 |  | Unknown | 2.16 | 1.05 | 1.25 | 1.03 |
| CG31238 | 51238 | 31238 |  | Unknown | 2.16 | 1.00 | 1.19 | 1.01 |
| puc | 4210 | 7850 |  | Immune signaling | 2.16 | 1.74 | 5.23 | 2.31 |
| HDC05276 |  |  | 5276 | Unknown | 2.15 | 1.17 | 0.26 | 1.04 |
| CG32638 | 52638 | 32638 |  | Unknown | 2.15 | 1.09 | 1.50 | 1.00 |
| CG5048 | 36437 | 5048 |  | Unknown | 2.15 | 1.30 | 0.68 | 1.11 |
| Her | 30899 | 5927 |  | DNA binding | 2.15 | 1.02 | 0.27 | 0.96 |
| HDC18862 |  |  | 18862 | Unknown | 2.15 | 1.11 | 1.17 | 1.02 |
| HDC03592 |  |  | 3592 | Unknown | 2.14 | 1.17 | 0.92 | 1.02 |
| Cyp9c1 | 15040 | 3616 |  | Metabolism | 2.14 | 1.00 | 1.75 | 1.08 |
| CG12493 | 35571 | 12493 |  | Unknown | 2.13 | 1.28 | -0.15 | 1.06 |
| CG33339 | 53339 | 33339 |  | Unknown | 2.13 | 1.12 | 0.54 | 0.95 |
| HDC16673 |  |  | 16673 | Unknown | 2.13 | 1.43 | -1.33 | 0.89 |
| HDC13081 |  |  | 13081 | Unknown | 2.12 | 1.35 | -0.82 | 0.96 |
| CG32102 | 52102 | 32102 |  | Unknown | 2.12 | 1.02 | 1.42 | 1.00 |
| Tsp29Fb | 32075 | 9496 |  | Unknown | 2.12 | 1.27 | 0.11 | 0.98 |
| CG32365 | 52365 | 32365 |  | Unknown | 2.12 | 1.26 | -0.44 | 0.96 |
| CG15627 | 31634 | 15627 |  | Signaling | 2.12 | 1.12 | 0.75 | 1.10 |
| Alas | 20764 | 3017 |  | Metabolism | 2.11 | 1.22 | 1.32 | 0.94 |
| CG32428 | 52428 | 32428 |  | Unknown | 2.11 | 1.05 | 1.70 | 1.02 |
| CG9098 | 31762 | 9098 |  | Signaling | 2.11 | 1.26 | 0.04 | 0.87 |
| CG6121 | 26080 | 6121 |  | DNA binding | 2.10 | 1.22 | 2.57 | 1.26 |
| HDC05924 |  |  | 5924 | Unknown | 2.10 | 1.30 | 0.34 | 1.10 |
| CG33309 | 53309 | 33309 |  | Unknown | 2.10 | 1.21 | 0.53 | 0.99 |
| CG30495 | 50495 | 30495 |  | Metabolism | 2.10 | 1.34 | -0.92 | 0.91 |
| HDC04697 |  |  | 4697 | Unknown | 2.10 | 1.08 | 1.54 | 1.09 |
| CG7498 | 40833 | 7498 |  | Unknown | 2.10 | 1.29 | 0.51 | 1.06 |
| bonsai | 26261 | 4207 |  | DNA binding | 2.10 | 1.07 | -0.29 | 0.98 |
| HDC15882 |  |  | 15882 | Unknown | 2.09 | 1.04 | 0.82 | 0.92 |
| CG14305 | 38630 | 14305 |  | Signaling | 2.09 | 1.11 | 1.15 | 1.10 |
| CG4461 | 35982 | 4461 |  | Unknown | 2.09 | 1.18 | 1.30 | 1.15 |
| CG32745 | 52745 | 32745 |  | DNA binding | 2.09 | 1.26 | -0.90 | 1.01 |
| zip | 5634 | 15792 |  | Cytoskeletal | 2.09 | 1.30 | 1.13 | 1.17 |
| CG18586 | 35642 | 18586 |  | Metabolism | 2.09 | 1.32 | -1.11 | 1.13 |
| HDC17872 |  |  | 17872 | Unknown | 2.09 | 1.30 | 0.27 | 0.99 |
| CG9561 | 31203 | 16788 |  | RNA binding | 2.08 | 1.26 | 0.78 | 1.01 |
| CG30270 | 61435 | 30270 |  | Unknown | 2.08 | 1.07 | 0.97 | 1.05 |
| CG31427 | 51427 | 31427 |  | Proteolysis | 2.08 | 1.30 | 0.73 | 1.00 |
| AGO1 | 26611 | 6671 |  | Other | 2.08 | 1.32 | 0.13 | 1.02 |
| HDC06022 |  |  | 6022 | Unknown | 2.08 | 1.06 | 1.35 | 1.08 |
| HDC08262 |  |  | 8262 | Unknown | 2.08 | 1.31 | -1.04 | 1.01 |
| slmo | 29161 | 9131 |  | Unknown | 2.07 | 1.20 | 2.45 | 1.31 |
| CG13474 | 36439 | 13474 |  | Unknown | 2.07 | 1.20 | -0.22 | 1.00 |
| janB | 1281 | 7931 |  | Unknown | 2.07 | 1.45 | -0.50 | 0.95 |
| CG1287 | 37506 | 1287 |  | Unknown | 2.07 | 1.20 | 0.97 | 1.11 |
| mit(1)15 | 4643 | 9900 |  | Other | 2.07 | 1.34 | -1.57 | 0.98 |
| CG33260 | 53260 | 33260 |  | Unknown | 2.07 | 1.25 | 0.12 | 1.05 |
| Sr-CII | 20377 | 8856 |  | Other | 2.07 | 1.35 | 1.60 | 1.13 |
| CG5742 | 34304 | 5742 |  | Unknown | 2.07 | 1.12 | 0.63 | 1.10 |
| alien | 13746 | 9556 |  | Proteolysis | 2.07 | 1.20 | 1.27 | 0.94 |
| CG5107 | 39342 | 5107 |  | Unknown | 2.06 | 1.17 | -1.18 | 0.89 |
| CG17855 | 32124 | 17855 |  | Unknown | 2.06 | 1.16 | 0.33 | 1.07 |
| CG14635 | 29535 | 14635 |  | Unknown | 2.06 | 1.10 | 0.46 | 0.98 |
| CG15256 | 28880 | 15256 |  | Unknown | 2.06 | 1.23 | 1.30 | 1.04 |
| CG8550 | 33742 | 8550 |  | Other | 2.06 | 1.14 | 1.63 | 1.08 |
| Pros26 | 2284 | 4097 |  | Proteolysis | 2.06 | 1.28 | 1.74 | 1.19 |
| HDC07058 |  |  | 7058 | Unknown | 2.06 | 1.14 | -0.29 | 1.00 |
| CG14545 | 40602 | 14545 |  | Unknown | 2.06 | 1.09 | 0.52 | 0.91 |
| CG9086 | 30809 | 9086 |  | Other | 2.06 | 1.55 | 0.48 | 1.12 |
| CG17666 | 36311 | 17666 |  | Unknown | 2.06 | 1.04 | 1.11 | 1.00 |
| msta | 53548 | 32800 |  | Unknown | 2.06 | 1.37 | -1.07 | 1.01 |
| CG13167 | 33706 | 13167 |  | Metabolism | 2.05 | 1.27 | -1.47 | 0.78 |
| CG31787 | 51787 | 31787 |  | Unknown | 2.05 | 1.06 | 0.37 | 1.07 |
| Rpn1 | 28695 | 7762 |  | Proteolysis | 2.05 | 1.31 | 1.09 | 1.20 |
| HDC14836 |  |  | 14836 | Unknown | 2.05 | 1.05 | 0.13 | 0.95 |
| org-1 | 21767 | 11202 |  | DNA binding | 2.05 | 1.16 | 0.21 | 0.88 |
| l(3)82Fd | 13576 | 32464 |  | Unknown | 2.05 | 1.18 | 0.57 | 1.00 |
| tim | 14396 | 3234 |  | Other | 2.05 | 1.10 | 1.18 | 0.93 |
| CG31423 | 51423 | 31423 |  | Unknown | 2.05 | 1.15 | 0.77 | 1.09 |
| CG12581 | 37213 | 12581 |  | Unknown | 2.05 | 1.26 | 0.77 | 1.05 |
| CG9822 | 34623 | 9822 |  | Unknown | 2.05 | 1.25 | 0.55 | 1.08 |
| CG3065 | 34946 | 3065 |  | DNA binding | 2.04 | 1.19 | 1.92 | 1.22 |
| CG6479 | 36710 | 6479 |  | Unknown | 2.04 | 1.02 | 1.18 | 0.98 |
| CG13800 | 35338 | 13800 |  | Unknown | 2.04 | 1.03 | 1.76 | 1.03 |
| ine | 11603 | 15444 |  | Other | 2.04 | 1.24 | 0.00 | 0.95 |
| Taf4 | 10280 | 5444 |  | DNA binding | 2.04 | 1.42 | 2.36 | 1.17 |
| CG9641 | 31483 | 9641 |  | Unknown | 2.04 | 1.12 | 1.03 | 1.07 |
| CG8310 | 40377 | 8310 |  | Other | 2.04 | 1.49 | 1.94 | 1.08 |
| Pros26.4 | 15282 | 5289 |  | Proteolysis | 2.03 | 1.34 | 1.01 | 1.32 |
| Cyp6d5 | 38194 | 3050 |  | Metabolism | 2.03 | 1.00 | 1.68 | 0.98 |
| CG6124 | 39484 | 6124 |  | Unknown | 2.03 | 1.07 | 1.17 | 1.02 |
| Gr22e | 45497 | 31936 |  | Unknown | 2.03 | 1.07 | 0.65 | 1.12 |
| CG31638 | 51638 | 31638 |  | Unknown | 2.03 | 1.09 | 1.24 | 1.09 |
| CG33182 | 53182 | 33182 |  | Unknown | 2.03 | 1.00 | 1.49 | 1.02 |
| zfh2 | 4607 | 1449 |  | DNA binding | 2.02 | 1.31 | 0.38 | 1.08 |
| lkb1 | 38167 | 9374 |  | Signaling | 2.02 | 1.32 | 0.41 | 1.00 |
| HDC07059 |  |  | 7059 | Unknown | 2.02 | 1.18 | -0.58 | 0.99 |
| CG2249 | 40773 | 2249 |  | Metabolism | 2.02 | 1.16 | -0.65 | 0.92 |
| CG14113 | 40814 | 14113 |  | Unknown | 2.01 | 1.04 | 1.44 | 1.05 |
| CG30265 | 50265 | 30265 |  | Other | 2.01 | 1.09 | 1.94 | 1.12 |
| CG14223 | 31053 | 14223 |  | Unknown | 2.01 | 1.10 | -0.06 | 1.04 |
| CG31128 | 51128 | 31128 |  | Unknown | 2.01 | 1.02 | 1.07 | 0.95 |
| CG32016 | 52016 | 32016 |  | Unknown | 2.01 | 1.37 | -0.87 | 0.99 |
| CG2079 | 29944 | 2079 |  | Signaling | 2.01 | 1.33 | -1.06 | 0.78 |
| CG17612 | 31597 | 17612 |  | DNA binding | 2.01 | 1.28 | -0.33 | 0.89 |
| CG14384 | 38097 | 14384 |  | Unknown | 2.00 | 1.16 | -0.12 | 0.93 |
| HDC09412 |  |  | 9412 | Unknown | 2.00 | 1.22 | 0.04 | 0.92 |
| dpr12 | 33044 | 14469 |  | Unknown | 2.00 | 1.12 | 2.02 | 1.06 |
| Med24 | 40339 | 3034 |  | Unknown | 2.00 | 1.04 | 1.69 | 1.04 |
| CG11872 | 37806 | 11872 |  | Unknown | 2.00 | 1.13 | 0.28 | 1.01 |
| HDC04700 |  |  | 4700 | Unknown | 2.00 | 1.54 | -0.76 | 1.01 |
| CG4629 | 31299 | 4629 |  | Signaling | 2.00 | 1.04 | -0.17 | 1.03 |
| RpII33 | 26373 | 7885 |  | DNA binding | 2.00 | 1.29 | 1.68 | 1.26 |
| CG7236 | 31730 | 7236 |  | Signaling | 1.99 | 1.25 | 1.95 | 1.22 |
| CG4945 | 34137 | 4945 |  | Signaling | 1.99 | 0.93 | -0.11 | 1.08 |
| CG7071 | 38949 | 7071 |  | Unknown | 1.99 | 1.03 | 1.18 | 1.00 |
| HDC00770 |  |  | 770 | Unknown | 1.99 | 1.08 | -0.27 | 0.92 |
| CG5783 | 32670 | 5783 |  | Unknown | 1.99 | 1.12 | 0.83 | 0.97 |
| CG32937 | 52937 | 32937 |  | Unknown | 1.99 | 1.12 | 1.37 | 1.12 |
| HDC02900 |  |  | 2900 | Unknown | 1.99 | 1.32 | -1.06 | 1.04 |
| CG32297 | 52297 | 32297 |  | RNA binding | 1.99 | 1.35 | -0.21 | 1.08 |
| HDC12189 |  |  | 12189 | Unknown | 1.98 | 1.17 | 0.69 | 0.98 |
| Acyp2 | 38363 | 18505 |  | Unknown | 1.98 | 1.10 | 0.73 | 0.98 |
| CG5604 | 32208 | 5604 |  | Other | 1.98 | 1.24 | 1.54 | 1.28 |
| east | 10110 | 4399 |  | Unknown | 1.98 | 1.25 | 1.25 | 1.27 |
| Fur1 | 4509 | 10772 |  | Proteolysis | 1.97 | 1.23 | 0.46 | 1.17 |
| CG11110 | 34535 | 11110 |  | Proteolysis | 1.97 | 1.25 | 1.66 | 1.14 |
| CG31213 | 51213 | 31213 |  | Other | 1.97 | 1.02 | 0.59 | 1.02 |
| CG11050 | 31836 | 11050 |  | Unknown | 1.97 | 1.26 | 0.81 | 1.07 |
| CG32553 | 52553 | 32553 |  | Unknown | 1.97 | 1.03 | -0.11 | 0.95 |
| CG14633 | 29537 | 14633 |  | Unknown | 1.97 | 1.19 | -0.17 | 0.99 |
| hay | 1179 | 8019 |  | DNA binding | 1.97 | 1.40 | -0.65 | 0.98 |
| skl | 36786 | 13701 |  | Signaling | 1.97 | 1.05 | 0.38 | 0.93 |
| CG8120 | 37675 | 8120 |  | DNA binding | 1.97 | 1.34 | 2.12 | 1.08 |
| CG31082 | 51082 | 31082 |  | Unknown | 1.96 | 1.05 | 0.85 | 1.03 |
| HDC20240 |  |  | 20240 | Unknown | 1.96 | 1.30 | 1.74 | 1.04 |
| CG16941 | 38464 | 16941 |  | RNA binding | 1.96 | 1.16 | 0.89 | 1.15 |
| CG5694 | 32197 | 5694 |  | Unknown | 1.96 | 1.06 | 0.84 | 1.08 |
| Trap170 | 35145 | 12031 |  | DNA binding | 1.96 | 1.43 | 1.35 | 1.24 |
