# Supplementary material for: A Quantitative RNAi Screen for JNK Modifiers Identifies Pvr as a Novel Regulator of Drosophila Immune Signaling
Source: PLoS Pathog. 2009 Nov 6;5(11):e1000655. doi: 10.1371/journal.ppat.1000655 (PMC2766254; doi:10.1371/journal.ppat.1000655)
Supplement: Table S4 — z-score analysis of dsRNA-mediated depletion of suppressors of 60 min PGN-induced dJNK phosphorylation. In-cell Western z-scores were calculated from P-JNK:f-actin values from S2 cells incubated with 15,683 dsRNAs and treated with PGN for 15 or 60 min. dsRNAs that modified 60 min PGN-induced P-JNK:f-actin z-scores above 1.96 (95% CI) are ordered from highest to lowest z-score. The fold change in dJNK phosphorylation relative to the plate median is shown alongside the z-score values for both 15 and 60 min time points. Each dsRNA is identified by its symbol, Celera Genome (CG) number, Heidelberg Drosophila Consortium identification number (HCDID) and general function. (0.50 MB DOC) [file ppat.1000655.s005.doc]

Table S4 – Suppressors of 60 min PGN-induced P-dJNK.

| Symbol | FBGN | CG | HDCID | GO | 60min  z-score | 60min  ΔP-JNK | 15min  z-score | 15min ΔP-JNK |
| --- | --- | --- | --- | --- | --- | --- | --- | --- |
| key | 41205 | 16910 |  | Immune Signaling | 9.23 | 3.43 | 9.06 | 2.85 |
| ird5 | 24222 | 4201 |  | Immune Signaling | 7.74 | 2.18 | 6.23 | 1.76 |
| pims | 34647 | 15678 |  | Immune Signaling | 7.28 | 2.40 | 4.63 | 1.82 |
| Cka | 44323 | 7392 |  | Signaling | 7.20 | 2.11 | 7.70 | 2.51 |
| UBL3 | 26076 | 9038 |  | Other | 6.13 | 1.29 | 4.96 | 1.72 |
| Pomp | 32884 | 9324 |  | Proteolysis | 5.65 | 1.95 | 2.71 | 1.58 |
| Scgα | 32013 | 7851 |  | Cytoskeletal | 5.58 | 2.34 | 2.18 | 1.77 |
| porin | 4363 | 6647 |  | Other | 5.50 | 1.28 | 5.88 | 1.41 |
| CG14852 | 38242 | 14852 |  | Unknown | 5.36 | 1.46 | -0.47 | 1.06 |
| puc | 4210 | 7850 |  | Immune Signaling | 5.23 | 2.31 | 2.16 | 1.74 |
| Rpn6 | 28689 | 10149 |  | Proteolysis | 5.02 | 1.44 | 2.40 | 1.10 |
| CG11526 | 35437 | 11526 |  | Unknown | 4.67 | 1.50 | 7.35 | 2.26 |
| CG4729 | 36623 | 4729 |  | Metabolism | 4.63 | 1.35 | 4.19 | 1.34 |
| mRNA-capping-enzyme | 30556 | 1810 |  | RNA binding | 4.60 | 1.51 | 2.23 | 1.22 |
| Spt6 | 28982 | 12225 |  | RNA binding | 4.58 | 1.60 | 1.72 | 1.39 |
| dup | 996 | 8171 |  | DNA binding | 4.54 | 1.16 | 0.64 | 0.93 |
| pAbp | 3031 | 5119 |  | RNA binding | 4.48 | 1.39 | 2.71 | 1.21 |
| CG14313 | 38579 | 14313 |  | Unknown | 4.46 | 1.72 | 3.41 | 1.50 |
| Act57B | 44 | 10067 |  | Cytoskeletal | 4.28 | 1.13 | 3.28 | 1.20 |
| CG9304 | 34674 | 9304 |  | Unknown | 4.28 | 1.22 | 3.36 | 1.40 |
| Act5C | 42 | 4027 |  | Cytoskeletal | 4.28 | 1.42 | 4.90 | 1.37 |
| CG31386 | 51386 | 31386 |  | Unknown | 4.18 | 1.42 | 1.30 | 1.04 |
| Act42A | 43 | 12051 |  | Cytoskeletal | 4.13 | 1.00 | 2.92 | 1.08 |
| Suv4-20 | 25639 | 13363 |  | Other | 4.00 | 1.22 | 3.92 | 1.14 |
| ss | 3513 | 6993 |  | DNA binding | 3.91 | 1.25 | 2.40 | 1.28 |
| CG10158 | 31871 | 10158 |  | Unknown | 3.88 | 1.42 | 4.57 | 1.34 |
| CG11294 | 30058 | 11294 |  | DNA binding | 3.75 | 1.22 | 0.75 | 1.05 |
| CG9769 | 37270 | 9769 |  | Translation | 3.72 | 1.19 | 0.94 | 1.01 |
| CG5819 | 34717 | 5819 |  | Unknown | 3.69 | 1.64 | 0.23 | 1.00 |
| cnk | 21818 | 6556 |  | Signaling | 3.63 | 1.31 | 5.20 | 2.04 |
| SNF4Aγ | 25803 | 17299 |  | Signaling | 3.62 | 1.68 | 1.66 | 1.37 |
| ken | 11236 | 5575 |  | DNA binding | 3.57 | 1.19 | 5.07 | 1.35 |
| kay | 1297 | 15509 |  | DNA binding | 3.56 | 1.48 | 3.06 | 1.27 |
| HDC08161 |  |  | 8161 | Unknown | 3.54 | 1.19 | 1.12 | 1.03 |
| raptor | 29840 | 4320 |  | Signaling | 3.48 | 1.21 | 4.07 | 1.30 |
| not | 13717 | 4166 |  | Other | 3.42 | 1.45 | 1.72 | 1.26 |
| scaf6 | 52168 | 32168 |  | RNA binding | 3.41 | 1.09 | 3.19 | 1.13 |
| Dl | 463 | 3619 |  | Signaling | 3.40 | 1.22 | 2.84 | 1.06 |
| CG6028 | 38924 | 6028 |  | Metabolism | 3.39 | 1.66 | 0.00 | 1.07 |
| e(y)1 | 617 | 6474 |  | DNA binding | 3.33 | 1.14 | 2.17 | 1.16 |
| CG9297 | 38181 | 9297 |  | Unknown | 3.32 | 1.04 | 1.83 | 1.20 |
| Arp66B | 11744 | 7558 |  | Cytoskeletal | 3.28 | 1.12 | 2.58 | 1.13 |
| RpS23 | 33912 | 8415 |  | Translation | 3.27 | 1.00 | 0.34 | 0.89 |
| RpS3A | 17545 | 2168 |  | Translation | 3.26 | 1.16 | 1.19 | 1.08 |
| dve | 20307 | 5799 |  | DNA binding | 3.25 | 1.67 | 1.45 | 1.09 |
| HmgD | 4362 | 17950 |  | DNA binding | 3.25 | 1.44 | 1.13 | 1.15 |
| SNF1A | 23169 | 3051 |  | Signaling | 3.24 | 1.42 | 0.45 | 0.99 |
| CG6012 | 32615 | 6012 |  | Metabolism | 3.24 | 1.56 | -0.30 | 1.02 |
| Ef2b | 559 | 2238 |  | Translation | 3.24 | 1.29 | -0.53 | 1.01 |
| Rpn12 | 28693 | 4157 |  | Proteolysis | 3.19 | 1.43 | 1.20 | 1.29 |
| gce | 30627 | 6211 |  | Unknown | 3.17 | 1.32 | 0.16 | 1.15 |
| CG12050 | 32915 | 12050 |  | Unknown | 3.17 | 1.00 | 3.22 | 1.16 |
| CG6013 | 38675 | 6013 |  | Unknown | 3.17 | 1.66 | -0.06 | 1.14 |
| CG32000 | 52000 | 32000 |  | Other | 3.14 | 1.29 | 3.76 | 1.23 |
| Prosβ3 | 26380 | 11981 |  | Proteolysis | 3.12 | 1.29 | 1.88 | 1.24 |
| RhoGAP18B | 30986 | 7481 |  | Signaling | 3.12 | 1.29 | 4.20 | 1.42 |
| mRpL-CI-B8 | 34893 | 5479 |  | Translation | 3.05 | 1.18 | 2.96 | 1.25 |
| CG5114 | 36460 | 5114 |  | Unknown | 3.04 | 1.38 | 1.09 | 1.02 |
| CG5728 | 39182 | 5728 |  | RNA binding | 3.02 | 1.72 | 0.87 | 1.09 |
| lilli | 41111 | 8817 |  | Other | 3.01 | 1.28 | 1.23 | 1.07 |
| CG8771 | 33766 | 8771 |  | Unknown | 2.99 | 1.08 | 3.48 | 1.08 |
| ik2 | 28633 | 2615 |  | Signaling | 2.99 | 1.20 | 2.22 | 1.18 |
| Bx | 242 | 6500 |  | DNA binding | 2.96 | 1.28 | 3.56 | 1.37 |
| Jon99Ci | 3358 | 31039 |  | Proteolysis | 2.95 | 1.39 | 1.57 | 1.13 |
| CG31406 | 51406 | 31406 |  | Unknown | 2.92 | 1.12 | 0.81 | 1.06 |
| Rel | 14018 | 11992 |  | Immune Signaling | 2.91 | 1.57 | 0.01 | 1.03 |
| dpr9 | 38282 | 33485 |  | Unknown | 2.90 | 1.21 | 0.69 | 1.01 |
| TfIIEβ | 15829 | 1276 |  | DNA binding | 2.90 | 1.35 | 2.76 | 1.27 |
| CG9886 | 31428 | 9886 |  | Metabolism | 2.88 | 1.10 | -0.80 | 0.88 |
| rept | 40075 | 9750 |  | DNA binding | 2.88 | 1.15 | 1.79 | 1.11 |
| AnnX | 84 | 9579 |  | Other | 2.88 | 1.12 | 0.84 | 1.05 |
| CG8243 | 33349 | 8243 |  | Signaling | 2.88 | 1.03 | 0.11 | 0.96 |
| CG32628 | 52628 | 32628 |  | Unknown | 2.87 | 1.22 | 1.41 | 1.18 |
| CycT | 25455 | 6292 |  | Signaling | 2.87 | 1.30 | 0.73 | 1.07 |
| CG5909 | 39495 | 5909 |  | Proteolysis | 2.86 | 1.50 | 1.36 | 1.27 |
| RpS12 | 14027 | 11271 |  | Translation | 2.86 | 1.05 | -0.28 | 0.81 |
| CG8436 | 37670 | 8436 |  | Unknown | 2.85 | 1.25 | 1.00 | 1.00 |
| HDC14817 |  |  | 14817 | Unknown | 2.81 | 1.15 | 2.93 | 1.19 |
| HDC19589 |  |  | 19589 | Unknown | 2.81 | 0.98 |  | 0.96 |
| CG32073 | 52073 | 32073 |  | Unknown | 2.80 | 1.15 | 1.56 | 1.01 |
| CG8793 | 36894 | 8793 |  | Unknown | 2.80 | 1.27 | 1.24 | 1.13 |
| CG6006 | 63649 | 6006 |  | Other | 2.79 | 1.32 | 2.33 | 1.11 |
| CG11436 | 29713 | 11436 |  | Unknown | 2.77 | 1.09 | 1.77 | 1.13 |
| CanB2 | 15614 | 11217 |  | Signaling | 2.77 | 1.40 | -0.35 | 1.04 |
| RpL18 | 35753 | 8615 |  | Translation | 2.75 | 1.23 | 1.56 | 1.19 |
| RpII140 | 3276 | 3180 |  | DNA binding | 2.74 | 1.41 | 5.37 | 1.88 |
| Su(Tpl) | 14037 | 32217 |  | DNA binding | 2.74 | 1.29 | 2.29 | 1.13 |
| CG18545 | 37812 | 18545 |  | Unknown | 2.74 | 1.13 | 3.05 | 1.13 |
| CG16791 | 38881 | 16791 |  | Unknown | 2.71 | 1.63 | -0.49 | 1.03 |
| cnc | 338 | 17894 |  | DNA binding | 2.70 | 1.34 | 3.58 | 1.47 |
| CG9523 | 31812 | 9523 |  | Unknown | 2.69 | 1.22 | 3.00 | 1.30 |
| CG11984 | 37655 | 11984 |  | Other | 2.69 | 1.13 | 3.57 | 1.40 |
| Camta | 33417 | 8809 |  | DNA binding | 2.69 | 0.83 | 1.95 | 0.98 |
| CG4896 | 31319 | 4896 |  | RNA binding | 2.69 | 1.27 | 2.94 | 1.62 |
| CG33330 | 53330 | 33330 |  | Unknown | 2.67 | 1.24 | -0.78 | 1.06 |
| CG5823 | 38515 | 5823 |  | Proteolysis | 2.67 | 1.43 | 1.00 | 0.95 |
| CG12992 | 30846 | 12992 |  | Unknown | 2.67 | 1.50 | -0.16 | 1.11 |
| UbcD10 | 26316 | 5788 |  | Proteolysis | 2.67 | 1.41 | -0.02 | 0.94 |
| Syx1A | 13343 | 31136 |  | Other | 2.66 | 1.33 | -0.41 | 0.94 |
| AnnIX | 83 | 5730 |  | Signaling | 2.65 | 1.49 | -0.01 | 1.01 |
| CG11811 | 36099 | 11811 |  | Metabolism | 2.65 | 1.23 | 0.20 | 1.01 |
| caup | 15919 | 10605 |  | DNA binding | 2.64 | 1.10 | 1.21 | 0.86 |
| B52 | 4587 | 10851 |  | RNA binding | 2.63 | 1.29 | 3.42 | 1.27 |
| Fs(2)Ket | 986 | 2637 |  | Other | 2.62 | 1.19 | 1.75 | 1.14 |
| nec | 2930 | 1857 |  | Signaling | 2.61 | 1.47 | -0.13 | 1.09 |
| MED26 | 39923 | 1793 |  | DNA binding | 2.59 | 1.46 | 2.29 | 1.49 |
| LBR | 34657 | 17952 |  | Unknown | 2.59 | 1.24 | 1.70 | 1.14 |
| CG13647 | 39256 | 13647 |  | Unknown | 2.58 | 1.17 | 1.26 | 0.99 |
| HDC14013 |  |  | 14013 | Unknown | 2.58 | 1.08 | 1.00 | 1.01 |
| drpr | 35261 | 2086 |  | Signaling | 2.58 | 1.17 | 4.03 | 1.35 |
| Tip60 | 26080 | 6121 |  | DNA binding | 2.57 | 1.26 | 2.10 | 1.22 |
| MED21 | 40020 | 11023 |  | DNA binding | 2.56 | 1.29 | 2.33 | 1.13 |
| CG3891 | 35993 | 3891 |  | DNA binding | 2.55 | 1.35 | 3.70 | 1.52 |
| HLHm3 | 2609 | 8346 |  | DNA binding | 2.54 | 0.92 | 5.37 | 2.00 |
| CG9572 | 31089 | 9572 |  | Unknown | 2.52 | 1.13 | 0.40 | 0.90 |
| Eip74EF | 567 | 32180 |  | DNA binding | 2.51 | 1.19 | 3.42 | 1.32 |
| norpA | 4625 | 3620 |  | Metabolism | 2.51 | 1.24 | 0.11 | 0.89 |
| CG8636 | 29629 | 8636 |  | RNA binding | 2.51 | 1.33 | -0.71 | 0.91 |
| RpS18 | 10411 | 8900 |  | Translation | 2.51 | 0.97 | 1.06 | 0.95 |
| CG5822 | 31674 | 31919 |  | Unknown | 2.49 | 1.41 | 0.50 | 0.98 |
| aur | 147 | 3068 |  | Signaling | 2.49 | 1.24 | 1.78 | 1.10 |
| sprt | 50023 | 30023 |  | Unknown | 2.46 | 1.11 |  | 1.05 |
| Cyp4d2 | 11576 | 3466 |  | Metabolism | 2.45 | 1.49 | 0.99 | 1.09 |
| slmo | 29161 | 9131 |  | Unknown | 2.45 | 1.31 | 2.07 | 1.20 |
| drk | 4638 | 6033 |  | Signaling | 2.44 | 1.49 | 1.57 | 1.43 |
| l(2)k01209 | 22029 | 4798 |  | Metabolism | 2.44 | 1.10 | 2.82 | 1.06 |
| CG9360 | 30332 | 9360 |  | Metabolism | 2.43 | 1.31 | -0.04 | 1.00 |
| Ppox | 20018 | 5796 |  | Metabolism | 2.41 | 1.38 | 0.94 | 1.13 |
| MED19 | 36761 | 5546 |  | DNA binding | 2.40 | 1.22 | 4.06 | 1.55 |
| CG1244 | 35357 | 1244 |  | Unknown | 2.40 | 1.27 | 3.10 | 1.48 |
| α-Cat | 10215 | 17947 |  | Cytoskeletal | 2.39 | 1.34 | 1.55 | 1.23 |
| CG30352 | 50352 | 30352 |  | Unknown | 2.39 | 1.11 | 1.00 | 1.04 |
| Hexo2 | 41629 | 1787 |  | Metabolism | 2.39 | 1.19 | 2.69 | 1.34 |
| CG17494 | 40011 | 12002 |  | Other | 2.38 | 1.27 | 4.16 | 1.47 |
| CG2616 | 37512 | 2616 |  | Other | 2.38 | 1.06 | 0.47 | 0.92 |
| HDC07791 |  |  | 7791 | Unknown | 2.37 | 1.13 | 0.89 | 1.10 |
| CG18363 | 36808 | 18363 |  | Other | 2.37 | 1.27 | 0.62 | 1.12 |
| CG17745 | 32386 | 17745 |  | Unknown | 2.37 | 1.08 | 0.13 | 0.91 |
| CG7231 | 31968 | 7231 |  | Unknown | 2.37 | 1.01 | 1.36 | 1.05 |
| CG31524 | 51524 | 31524 |  | Metabolism | 2.36 | 1.43 | -0.78 | 1.03 |
| Taf4 | 10280 | 5444 |  | DNA binding | 2.36 | 1.17 | 2.04 | 1.42 |
| HDC02460 |  |  | 2460 | Unknown | 2.36 | 1.06 | 1.24 | 1.04 |
| zormin | 52311 | 33484 |  | Cytoskeletal | 2.36 | 1.18 | 2.47 | 1.26 |
| CG7069 | 38952 | 7069 |  | Metabolism | 2.35 | 1.08 | 1.57 | 1.04 |
| dnk | 22338 | 5452 |  | Metabolism | 2.34 | 1.14 | 2.22 | 1.13 |
| CG8105 | 30661 | 8105 |  | Metabolism | 2.34 | 1.12 | 0.29 | 0.99 |
| CG6095 | 39401 | 6095 |  | Unknown | 2.33 | 1.21 | 0.52 | 1.00 |
| CG7177 | 37098 | 7177 |  | Signaling | 2.33 | 1.35 | 3.12 | 1.45 |
| HDC16188 |  |  | 16188 | Unknown | 2.32 | 1.06 | 0.25 | 0.93 |
| HDC06346 |  |  | 6346 | Unknown | 2.32 | 1.08 | -0.16 | 0.85 |
| HDC02979 |  |  | 2979 | Unknown | 2.32 | 1.13 | -0.91 | 0.86 |
| Src42A | 4603 | 7873 |  | Signaling | 2.30 | 1.25 | 4.31 | 1.35 |
| insv | 31434 | 3227 |  | Unknown | 2.29 | 1.20 | 2.26 | 1.14 |
| CG9522 | 30587 | 9522 |  | Metabolism | 2.29 | 1.20 | 1.25 | 1.10 |
| CG5861 | 15338 | 5861 |  | Unknown | 2.29 | 1.36 | 0.16 | 1.02 |
| CG10481 | 32827 | 10481 |  | Signaling | 2.29 | 1.11 | 0.23 | 1.01 |
| CG32564 | 52564 | 32564 |  | Unknown | 2.29 | 1.14 | 1.21 | 1.03 |
| CG12169 | 35143 | 12169 |  | Signaling | 2.29 | 1.26 | 1.84 | 1.22 |
| CG31666 | 51666 | 31666 |  | Unknown | 2.28 | 1.35 | -1.29 | 1.05 |
| Rpt4 | 28685 | 3455 |  | Proteolysis | 2.28 | 1.62 | 2.28 | 1.58 |
| HDC09475 |  |  | 9475 | Unknown | 2.27 | 1.10 | 0.25 | 0.95 |
| dco | 2413 | 2048 |  | Signaling | 2.27 | 1.24 | 0.39 | 0.95 |
| CG14998 | 35500 | 14998 |  | Unknown | 2.27 | 1.17 | 2.31 | 1.15 |
| AP-2sigma | 43012 | 6056 |  | Other | 2.26 | 1.39 | -1.70 | 0.97 |
| CG10440 | 34636 | 10440 |  | Other | 2.26 | 1.17 | 1.32 | 1.05 |
| CG32354 | 52354 | 32354 |  | Unknown | 2.24 | 1.47 | -0.63 | 1.03 |
| Osi13 | 37422 | 15595 |  | Unknown | 2.24 | 1.04 | 1.14 | 1.00 |
| CG11505 | 35424 | 11505 |  | RNA binding | 2.24 | 1.12 | 1.86 | 1.21 |
| CG4119 | 28474 | 4119 |  | RNA binding | 2.24 | 1.23 | 3.62 | 1.43 |
| CG3960 | 29876 | 3960 |  | Cytoskeletal | 2.23 | 1.43 | 0.75 | 1.10 |
| l(1)G0022 | 30681 | 8231 |  | Other | 2.23 | 1.07 | 0.45 | 1.05 |
| RpS5 | 2590 | 8922 |  | Translation | 2.23 | 0.98 | 1.13 | 1.02 |
| CG3940 | 37788 | 3940 |  | Metabolism | 2.23 | 1.26 | 0.73 | 0.98 |
| CG32033 | 52033 | 32033 |  | Unknown | 2.23 | 1.19 | 0.58 | 0.96 |
| CG30085 | 50085 | 30085 |  | Unknown | 2.23 | 1.08 | 0.77 | 0.86 |
| CG5888 | 28523 | 5888 |  | Unknown | 2.22 | 1.50 | -0.93 | 0.95 |
| CG33251 | 53251 | 33251 |  | Unknown | 2.21 | 1.12 | 0.79 | 1.00 |
| CG32373 | 52373 | 32373 |  | Unknown | 2.21 | 1.47 | -1.46 | 0.98 |
| CG10752 | 36325 | 10752 |  | Unknown | 2.20 | 1.06 | 2.41 | 1.07 |
| Trf2 | 26758 | 18009 |  | DNA binding | 2.18 | 1.20 | 3.64 | 1.29 |
| CG12972 | 37076 | 12972 |  | DNA binding | 2.17 | 1.15 | 1.21 | 1.07 |
| CSN6 | 28837 | 6932 |  | Unknown | 2.16 | 1.05 | 1.17 | 1.06 |
| CG5948 | 39386 | 5948 |  | Metabolism | 2.16 | 1.46 | -0.15 | 0.96 |
| CG6735 | 36472 | 6735 |  | Cytoskeletal | 2.16 | 1.26 | 3.40 | 1.71 |
| CG5326 | 38983 | 5326 |  | Metabolism | 2.15 | 1.41 | -0.44 | 0.99 |
| smg | 16070 | 5263 |  | RNA binding | 2.15 | 1.06 | 2.43 | 1.16 |
| HDC19535 |  |  | 19535 | Unknown | 2.15 | 1.05 | 0.75 | 0.99 |
| CG31030 | 51030 | 31030 |  | Metabolism | 2.15 | 1.14 | 0.34 | 0.98 |
| CG11790 | 39265 | 11790 |  | Other | 2.15 | 1.13 | 1.10 | 1.06 |
| Ccp84Ag | 4777 | 2342 |  | Other | 2.14 | 1.15 | -0.19 | 1.03 |
| CG2901 | 29679 | 2901 |  | Signaling | 2.14 | 1.03 | 1.74 | 0.99 |
| SelR | 37847 | 6584 |  | Other | 2.14 | 1.11 | 1.61 | 1.06 |
| CG9576 | 31091 | 9576 |  | Unknown | 2.13 | 0.95 | -0.81 | 0.78 |
| HP1e | 37675 | 8120 |  | DNA binding | 2.12 | 1.08 | 1.97 | 1.34 |
| Smg5 | 19890 | 8954 |  | RNA binding | 2.12 | 1.25 | 4.59 | 1.80 |
| CG5284 | 36566 | 5284 |  | Other | 2.12 | 1.08 | 1.21 | 0.96 |
| CG5804 | 35926 | 5804 |  | Other | 2.12 | 1.14 | 1.17 | 1.02 |
| CG4404 | 30432 | 4404 |  | Unknown | 2.12 | 1.08 | 0.86 | 1.00 |
| pita | 34878 | 3941 |  | DNA binding | 2.12 | 1.34 | 0.55 | 0.93 |
| CG13749 | 33353 | 13749 |  | Unknown | 2.11 | 1.07 | 0.89 | 1.08 |
| TfIIFα | 10282 | 10281 |  | DNA binding | 2.11 | 1.21 | 0.47 | 0.93 |
| Ef1α48D | 556 | 8280 |  | Translation | 2.11 | 1.23 | -0.20 | 0.99 |
| tun | 34046 | 8253 |  | Unknown | 2.11 | 0.99 | 1.01 | 0.92 |
| CG5946 | 36211 | 5946 |  | Metabolism | 2.11 | 1.31 | 0.95 | 1.07 |
| CG11666 | 40648 | 11666 |  | Unknown | 2.11 | 1.08 | 1.57 | 1.04 |
| HDC03637 |  |  | 3637 | Unknown | 2.10 | 1.06 | 0.21 | 0.88 |
| CG3597 | 31417 | 3597 |  | Metabolism | 2.10 | 1.22 | -0.07 | 0.89 |
| TepIII | 41181 | 7068 |  | Unknown | 2.10 | 1.08 | 0.45 | 0.96 |
| Trip1 | 15834 | 8882 |  | Translation | 2.10 | 1.03 | 0.99 | 1.07 |
| CG31525 | 51525 | 31525 |  | Unknown | 2.10 | 1.10 | 1.43 | 1.03 |
| HDC06322 |  |  | 6322 | Unknown | 2.10 | 1.14 | 2.78 | 1.23 |
| Syx8 | 36643 | 4109 |  | Other | 2.10 | 1.08 | 1.53 | 1.02 |
| CG15211 | 30234 | 15211 |  | Unknown | 2.09 | 1.13 | 1.07 | 0.96 |
| CG14638 | 37223 | 14638 |  | Unknown | 2.09 | 1.04 | 0.89 | 1.06 |
| CG9727 | 37445 | 9727 |  | DNA binding | 2.09 | 1.05 | 0.39 | 0.94 |
| Orc4 | 23181 | 2917 |  | Other | 2.09 | 1.07 | 1.01 | 0.91 |
| Asph | 34075 | 8421 |  | Unknown | 2.09 | 1.24 | -1.50 | 0.83 |
| HDC19532 |  |  | 19532 | Unknown | 2.08 | 1.02 | 0.79 | 1.06 |
| CG12000 | 37314 | 12000 |  | Proteolysis | 2.08 | 1.15 | 1.11 | 1.10 |
| CG11727 | 30299 | 11727 |  | Signaling | 2.08 | 1.06 | 0.50 | 1.06 |
| CG3590 | 38467 | 3590 |  | Metabolism | 2.08 | 1.03 | 0.76 | 0.94 |
| CG9598 | 36424 | 9598 |  | Unknown | 2.07 | 0.93 | 1.69 | 1.09 |
| dj | 19828 | 1980 |  | Unknown | 2.07 | 1.14 | -1.11 | 0.96 |
| CG6016 | 33844 | 6016 |  | Metabolism | 2.07 | 1.43 | 0.00 | 1.14 |
| eca | 53104 | 33104 |  | Other | 2.07 | 0.97 | 0.63 | 1.05 |
| HDC10149 |  |  | 10149 | Unknown | 2.05 | 1.14 | 4.24 | 1.29 |
| CG6194 | 38325 | 6194 |  | Proteolysis | 2.05 | 1.33 | 0.33 | 1.08 |
| kto | 1324 | 8491 |  | DNA binding | 2.05 | 1.19 | 4.23 | 1.28 |
| bun | 10460 | 5461 |  | DNA binding | 2.05 | 1.20 | 5.75 | 1.54 |
| CG17737 | 35423 | 17737 |  | Translation | 2.05 | 1.10 | 0.51 | 0.99 |
| put | 3169 | 7904 |  | Signaling | 2.04 | 1.12 | 0.23 | 0.87 |
| CG5116 | 39339 | 5116 |  | Unknown | 2.04 | 1.21 | 1.20 | 1.04 |
| Sry-δ | 3512 | 17958 |  | DNA binding | 2.04 | 1.16 | 1.45 | 1.12 |
| CG18358 | 30782 | 18358 |  | Unknown | 2.04 | 1.17 | 0.49 | 1.01 |
| maf-S | 34534 | 9954 |  | DNA binding | 2.03 | 1.10 | 3.38 | 1.43 |
| CG8740 | 27585 | 8740 |  | Unknown | 2.03 | 1.10 | 0.40 | 1.00 |
| CG31738 | 51738 | 31738 |  | Cytoskeletal | 2.03 | 1.04 | 1.37 | 1.02 |
| sgg | 3371 | 2621 |  | Signaling | 2.03 | 1.12 | 0.43 | 0.96 |
| CG3622 | 34778 | 3622 |  | Proteolysis | 2.03 | 1.28 | 1.26 | 1.16 |
| HDC01229 |  |  | 1229 | Unknown | 2.02 | 1.12 | -0.11 | 0.92 |
| CG8603 | 33923 | 8603 |  | Unknown | 2.02 | 1.18 | 0.65 | 1.05 |
| orb | 4882 | 10868 |  | RNA binding | 2.02 | 1.12 | 0.64 | 0.85 |
| CG2614 | 32873 | 2614 |  | Unknown | 2.02 | 1.05 | 1.03 | 1.00 |
| dpr12 | 33044 | 14469 |  | Unknown | 2.02 | 1.06 | 2.00 | 1.12 |
| HDC03347 |  |  | 3347 | Unknown | 2.02 | 1.13 | 0.18 | 0.95 |
| hdc | 10113 | 15532 |  | Unknown | 2.02 | 1.35 | 0.60 | 1.08 |
| Optix | 25360 | 18455 |  | DNA binding | 2.01 | 1.15 | 1.76 | 1.14 |
| HDC06257 |  |  | 6257 | Unknown | 2.01 | 1.07 | 0.58 | 0.93 |
| CG13047 | 36594 | 13047 |  | Unknown | 2.01 | 1.14 | 2.59 | 1.08 |
| ksr | 15402 | 2899 |  | Signaling | 2.01 | 1.05 | 2.38 | 1.11 |
| CG11245 | 30388 | 11245 |  | Unknown | 2.01 | 0.93 | 3.15 | 1.14 |
| HDC03621 |  |  | 3621 | Unknown | 2.00 | 1.04 | 0.41 | 0.96 |
| CG6769 | 30878 | 6769 |  | Unknown | 2.00 | 1.12 | 0.45 | 1.09 |
| CG14741 | 37989 | 14741 |  | Metabolism | 2.00 | 1.22 | 1.77 | 1.12 |
| dia | 11202 | 1768 |  | Cytoskeletal | 2.00 | 1.09 | 1.54 | 1.01 |
| CG3474 | 28871 | 3474 |  | Other | 1.99 | 1.05 | 1.46 | 1.03 |
| γCop | 28968 | 1528 |  | Other | 1.99 | 1.16 | -0.55 | 0.78 |
| HDC16120 |  |  | 16120 | Unknown | 1.99 | 1.03 | 0.95 | 1.00 |
| CG2191 | 39873 | 2191 |  | Other | 1.99 | 1.12 | -0.03 | 0.89 |
| CG17570 | 32948 | 17570 |  | Unknown | 1.99 | 1.08 | 0.54 | 0.95 |
| Cyp28d1 | 31689 | 10833 |  | Metabolism | 1.99 | 1.03 | 0.99 | 0.90 |
| cas | 4878 | 2102 |  | DNA binding | 1.99 | 1.13 | 1.37 | 1.00 |
| RpL1 | 3279 | 5502 |  | Translation | 1.98 | 0.99 | 2.99 | 1.31 |
| CG3259 | 38221 | 3259 |  | Unknown | 1.98 | 0.99 | 1.83 | 1.21 |
| CG10795 | 34626 | 10795 |  | Unknown | 1.98 | 1.30 | 0.35 | 1.06 |
| Pka-R1 | 275 | 3263 |  | Signaling | 1.98 | 1.18 | 1.36 | 1.06 |
| NHP2 | 29148 | 5258 |  | RNA binding | 1.97 | 1.09 | 2.46 | 1.14 |
| HDC17098 |  |  | 17098 | Unknown | 1.97 | 1.01 | 1.76 | 1.02 |
| Parg | 23216 | 2864 |  | Metabolism | 1.97 | 1.09 | 0.97 | 1.04 |
| Nmdar1 | 10399 | 2902 |  | Signaling | 1.97 | 1.01 | 0.65 | 0.96 |
| CG15778 | 29788 | 15778 |  | Unknown | 1.97 | 1.05 | 0.87 | 0.97 |
| MED17 | 38578 | 7957 |  | DNA binding | 1.97 | 1.19 | 1.43 | 1.26 |
| CG3077 | 31457 | 3077 |  | Unknown | 1.96 | 1.36 | 0.58 | 1.19 |
| debcl | 29131 | 33134 |  | Signaling | 1.96 | 1.37 | -0.28 | 1.09 |
| CG12112 | 30048 | 12112 |  | Unknown | 1.96 | 1.02 | 0.90 | 0.99 |
